# Dithiane-directed Rh(iii)-catalyzed amidation of unactivated C(sp^3^)–H bonds†
†Electronic supplementary information (ESI) available: Experimental protocol and spectra data. See DOI: 10.1039/c8sc05225e


**DOI:** 10.1039/c8sc05225e

**Published:** 2019-02-18

**Authors:** Heyao Shi, Darren J. Dixon

**Affiliations:** a Chemistry Research Laboratory , 12 Mansfield Road , Oxford , OX1 3TA , UK . Email: darren.dixon@chem.ox.ac.uk

## Abstract

An oxidant-free Rh(iii)-catalyzed direct amidation of alkyl dithianes *via* C(sp^3^)–H bond activation utilizing diverse and robust dioxazolone reagents is reported. The reaction hinges on use of a Cp*Rh(iii) complex in combination with an essential amino-carboxylate additive to generate usefully protected 1,3-aminoaldehyde derivatives. The scalability of the reaction was demonstrated as was a series of downstream product functionalizations, including dithiane deprotection, anion alkylation and reductive desulfurization, highlighting the general applicability of this transformation in the synthesis of novel scaffolds and building blocks.

## Abstract 2

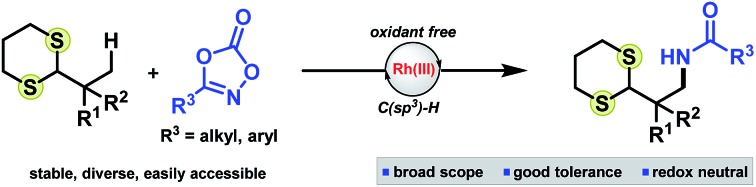
A Rh(iii) catalysed dithiane directed C(sp^3^)–H amidation for the synthesis of usefully protected β-aminoaldehyde derivatives is described.

## 

Dithianes are versatile building blocks in synthesis, acting as stable carbonyl and masked methylene surrogates as well as unique umpolung C1 acyl anion synthons as first pioneered by Corey and Seebach.^1^ Their synthetic accessibility combined with impressive and unique reactivity has led to widespread adoption of dithianes in complex target synthesis for this purpose.^2^ Alongside the very well-established lithium anion umpolung reactivity, discovery of new applications for this functional group has been the focus of much interest over the past decade^3^–^6^.

In parallel, transition metal catalyzed direct C–H functionalization reactions are a powerful strategy for step- and atom-economic synthesis,^7^ and Rh(iii)-catalysis has emerged in recent years as a powerful tool in the activation of various C(sp^2^)–H bonds. This area has been extensively reviewed,^8^ with a wide range of oxygen and nitrogen containing directing groups being found to be suitable in chelation-assisted activation of such bonds. However the use of sulfur-containing groups has been much less developed;^9^ the high Lewis basicity of sulfur and its strong coordination to metal centres is generally problematic for catalysis due to potential formation of less reactive, thermodynamically stable cyclometallated species.

Extension of Rh(iii)-catalysis to the much more challenging C(sp^3^)–H bond activation is an attractive, but difficult synthetic proposition which is still largely unrealized. Only a handful of examples exist for the activation of reactive C(sp^3^)–H bonds such as at acidic, allylic or benzylic positions.^10^ Regarding unactivated C(sp^3^)–H bonds, the high C–H bond strength, steric hinderance and low reactivity of the resulting Rh–C(alkyl) species are significant challenges which have only recently been reduced to practice by the seminal reports of You and Li.^11^ Both these reports have made a significant advancement to the field,^12^ but their use of strongly coordinating pyridyl and ketoxime functionality also highlights the need for identification of alternative directing groups which can be easily installed, cleaved, and enable synthetically useful downstream functionalization.

To this end, we decided to investigate dithiane functionality as a potential directing group for C–H activation of unreactive C(sp^3^)–H bonds.^13^ Dithianes have only previously been used once in catalytic C–H bond activation by Miura,^14^ enabling *ortho* C(sp^2^)–H alkenylation of benzylic dithianes with electron deficient olefins. Activation of alkyl substituted dithianes would therefore represent a powerful advancement, expanding the utility of this versatile functional group and enabling synthesis of sp^3^-rich scaffolds and building blocks bearing a useful umpolung acyl anion equivalent. Herein, we wish to report our findings (Scheme 1).

**Scheme 1:**
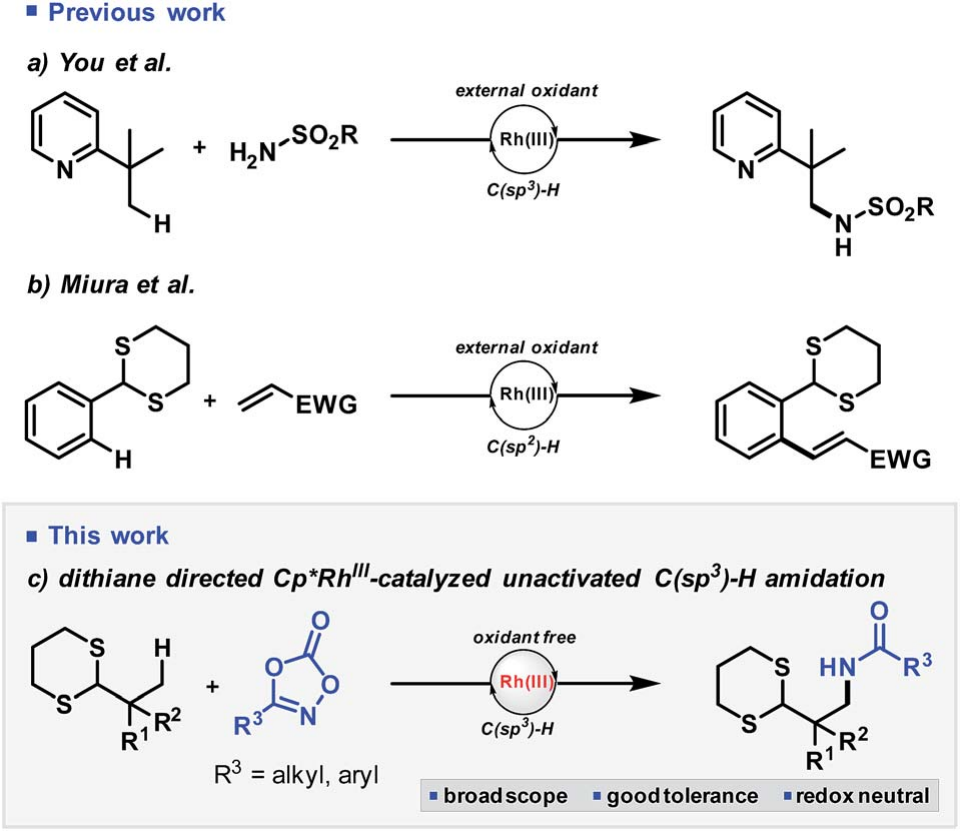
(a) You's seminal report on Rh(iii)-catalyzed functionalization of unactivated C(sp^3^)–H bonds. (b) Miura's oxidative C(sp^2^)–H *ortho* alkenylation of benzylic dithianes. (c) This work.

We began our studies by assessing Cp*Rh(iii) catalysis applied to pivaldehyde-derived dithiane **1a** and dioxazolone **2a** as model substrates. Pleasingly, treatment of **1a** with catalytic [Cp*Rh(MeCN)_3_][(SbF_6_)_2_] and sodium benzoate in dichloroethane (DCE) at 80 °C led to formation of amidodithiane **3a** in 16% yield (entry 1, Table 1). Remarkably, the reaction profile was extremely clean, with starting material conversion reflecting yield of product, and no bis-amidated was observed. Encouraged by this significant lead result establishing proof of concept, optimization studies were performed. Initial focus was placed on the solvent, and DCE proved optimal (entry 1), with toluene, dioxane and chloroform (entries 2–4) all completely suppressing reactivity. Next, a varied range of carboxylic acid salts^15^ – which were formed *in situ* by neutralisation of the acid with a slight excess of sodium *tert*-butoxide – were evaluated as additives to promote the reaction. Sterically bulky adamantyl and pivalic acid salts (entries 5 and 6), which are commonly used in C–H activation, provided only slightly improved yields of 23% and 18% respectively. Accordingly, we then turned towards amino acid salts as potential additives for the reaction (Table 1, entry 7 and Scheme 2). A range of differentially protected α-amino acid derivatives (**4a–o**) were screened, and interestingly Fmoc-protected sodium prolinate (**4h**) was found to be the most effective. Studies found 30 mol% of additive was optimal (entries 8–10) and use of sodium as the carboxylate counterion was critically important, with potassium, lithium and silver salts all displaying no reactivity whatsoever (entries 11–13). Temperature variations were also made, with the reaction yield decreasing when the temperature was both lowered to 70 °C (entry 14) or increased to 90 °C (entry 15). Finally, after extensive studies of all other reaction parameters, increasing the Rh catalyst loading from 10 to 15 mol% improved the yield to 71% (entry 16).

**Table 1:** Optimisation of reaction parameters

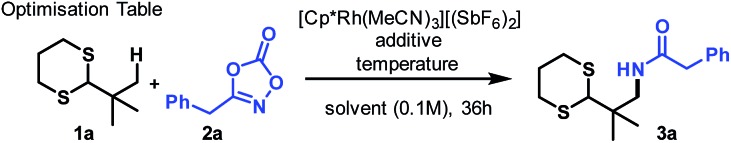					
Entry^*a*^	Solvent	Additive [mol%]	*T* [°C]	Cat. loading [mol %]	Yield^*b*^ [%]
1	DCE	PhCO_2_ Na (40)	80	10	16
2	Toluene	PhCO_2_ Na (40)	80	10	0
3	Dioxane	PhCO_2_ Na (40)	80	10	Trace
4	CHCI_3_	PhCO_2_ Na (40)	80	10	Trace
5	DCE	AdCO_2_ Na (40)	80	10	23
6	DCE	PivCO_2_ Na (40)	80	10	18
7	DCE	Amino acid (40)	80	10	Scheme 2
8	DCE	Fmoc-Pro-ONa (40)	80	10	55
9	DCE	Fmoc-Pro-ONa (30)	80	10	62
10	DCE	Fmoc-Pro-ONa (20)	80	10	57
11	DCE	Fmoc-Pro-OK (30)	80	10	0
12	DCE	Fmoc-Pro-OLi (30)	80	10	0
13	DCE	Fmoc-Pro-OAg (30)	80	10	0
14	DCE	Fmoc-Pro-ONa (30)	70	10	45
15	DCE	Fmoc-Pro-ONa (30)	90	10	47
**16**	**DCE**	**Fmoc-Pro-ONa (30)**	**80**	**15**	**73(71)**

^*a*^Reaction conditions: **1a** (0.05 mmol), **2a** (0.1 mmol), catalyst, additive, solvent (0.5 mL), temperature, 36 h.

^*b*^Yield calculated by ^1^H NMR with methyl *para*-nitrobenzoate as internal standard; isolated yield of product in parentheses.

**Scheme 2:**
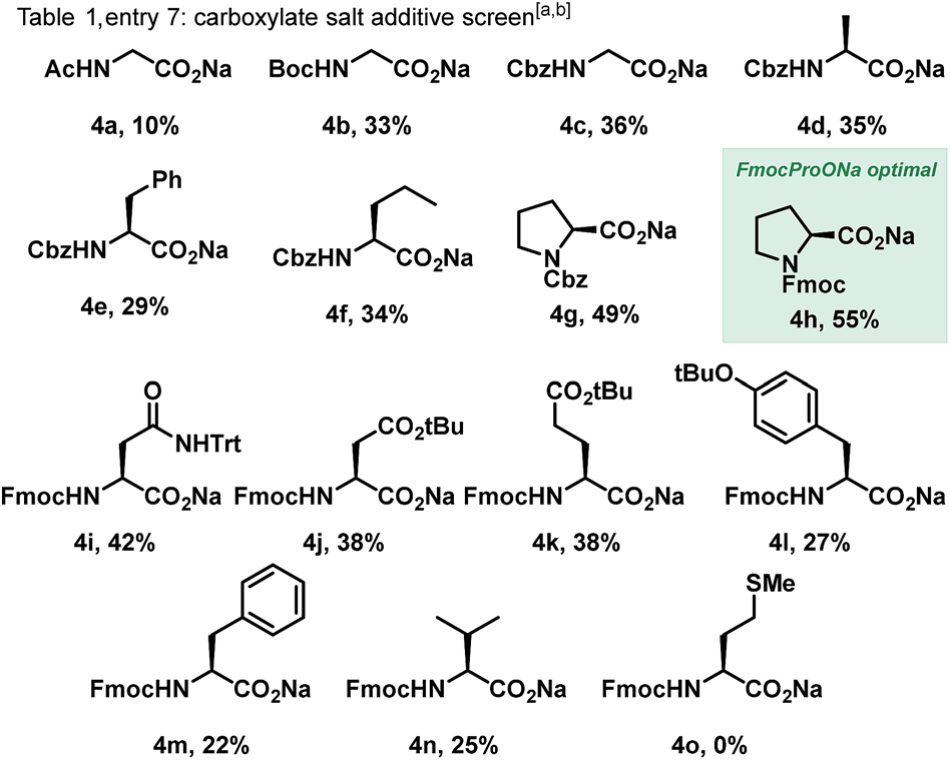
Evaluation of amino acid additives. ^*a*^Reaction conditions: **1a** (0.05 mmol), **2a** (0.1 mmol), [Cp*Rh(MeCN)_3_][(SbF_6_)_2_] (10 mol%), Fmoc-Pro-ONa (40 mol%), solvent (0.5 mL), temperature, 36 h. ^*b*^Yield calculated by ^1^H NMR with methyl *para*-nitrobenzoate as internal standard.

With optimal reaction conditions in hand, we explored the scope of the reaction, initially focusing on the dioxazolone amidating reagent. The reaction was found to be tolerant of a wide range of functionalized dioxazolones, with *p*-F, *p*-NO_2_ and *m*-OMe substituted phenylacetyl dioxazolones providing the amidated products in 74%, 40% and 67% yield respectively (Scheme 3, **3b–d**). Use of aromatic substituted dioxazolones was also tolerated, although a slight decrease of yield to moderate levels was observed. Phenyl, *p*-tolyl and bis-*meta*-methoxy substituted dioxazolones afforded good yields of the corresponding products (**3e–g**). Furan-bearing dithiane **3h** could also be synthesized in 46% yield. We postulate the reduced yield with aromatic dioxazolones was due to increased steric hinderance about the reaction centre, impeding reactivity. Despite this, a diverse range of acyclic alkyl substituted dioxazolones (**2i–o**) was shown to be successful, thus demonstrating applicability of this method towards the synthesis of sp^3^ rich scaffolds (**3i–o**). Finally, dioxazolones bearing medicinal chemistry-relevant small rings were also evaluated,^16^ with cyclopropyl (**2n**) and cyclobutyl (**2o**) bearing dithiane products (**3n**, **3o**) isolated in 45% and 52% yield respectively.

**Scheme 3:**
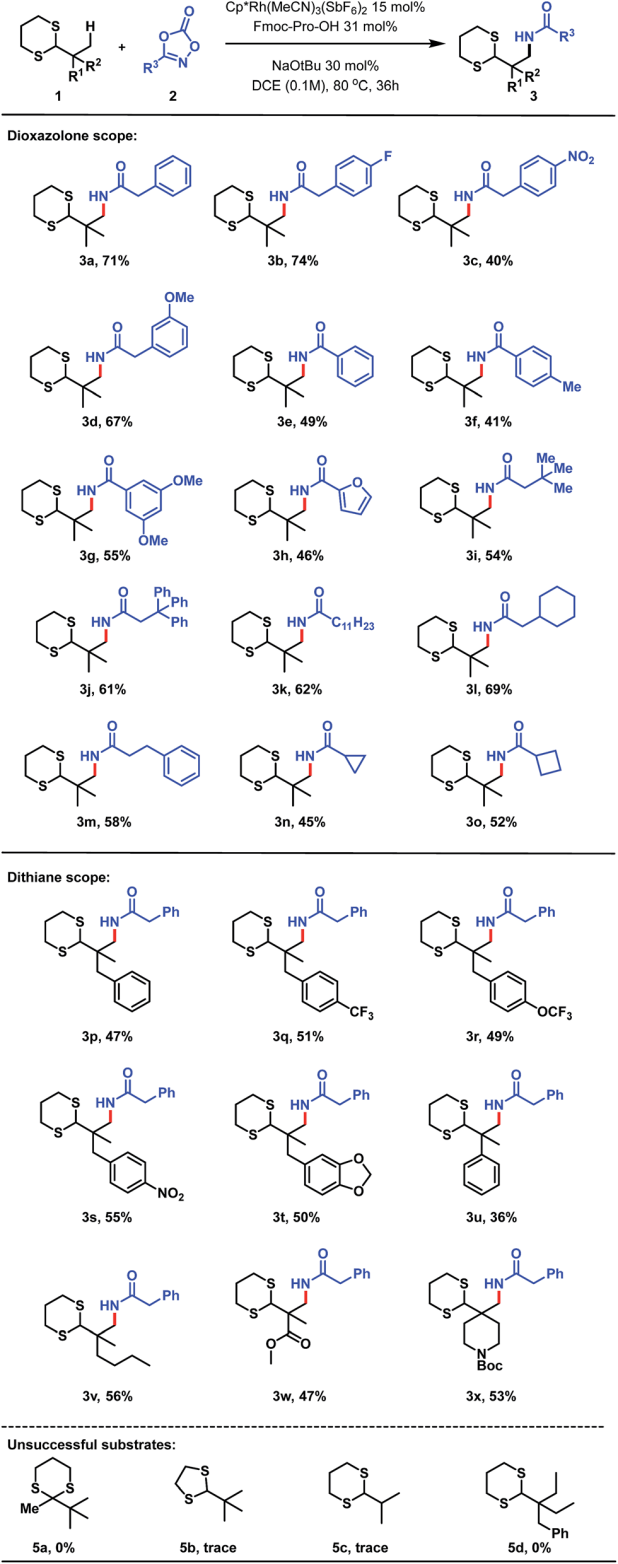
C(sp^3^)–H amidation of a range of dithianes **1** with diverse dioxazolones **2**.

The scope with respect to the dithiane partner was also found to be broad. Pleasingly, phenyl bearing dithiane (**1p**) delivered the corresponding amidated product in 47% yield.^17^ Trifluoromethyl (**1q**) and trifluoromethoxy (**1r**) substitution on the aromatic ring was also successfully accommodated, giving amidated products (**3q**, **3r**) in 51% and 49% yield respectively. The reaction was also tolerant of electron deficient *para*-nitro substituted dithiane (**3s**, 55%) as well as electron rich benzodioxole bearing dithiane (**3t**, 50%). Phenyl substitution directly at the quaternary centre delivered amidated product **3u**, albeit in low yield. Extension of the alkyl chain to a four-carbon subunit (**1v**) displayed a 56% yield for the corresponding amidated product **3v**. Pleasingly, methyl ester derived dithiane **1w** could also be activated, delivering amidoester product **3w** in 47% yield; demonstrating notable functional group tolerance for this methodology. Finally, *N*-Boc protected piperidine derived dithiane was utilized to afford **3x** in 53% yield. Control reactions using modified substrates were also performed. Interestingly, ketone-derived dithiane **5a** was completely unreactive to the standard reaction conditions, and dithiolane **5b** delivered only trace amounts of amidated product, highlighting the significance of the 6-membered ring aldehyde-derived dithiane for this transformation. The lack of reactivity in the case of **5c** demonstrates the requirement for an alpha-quaternary carbon. Ethyl-bearing dithiane **5d** was found to be completely unreactive, showing the unique selectivity of this method for methyl C–H bonds.

To investigate the practicality and efficiency of this transformation, a 56-fold scale up to 2.8 mmol (500 mg of **1a**) was performed using standard reaction conditions (Scheme 4a) and pleasingly **3a** was afforded in good yield. To demonstrate synthetic utility and versatility of the dithiane moiety, product derivatization was explored. Deprotection of dithiane **3a** to the corresponding aldehyde **6** was efficiently performed in 5 minutes with diacetoxyiodobenzene in a TFA/acetonitrile solvent mixture (Scheme 4b). Importantly, amido-dithiane **3e** was demonstrated to be successful in the Corey-Seebach alkylation with allyl bromide, providing allylated derivative **7** in 61% (Scheme 4c). Finally, desulfurization with RANEY® nickel to reveal a masked quaternary methyl group was performed on dithiane **3x**, affording **8** in 88% yield (Scheme 4d). This method provides an attractive method towards synthesis of such quaternary methyl bearing building blocks^18^ and structures such as **6–8** are attractive building blocks which could be of value within pharmaceutical drug discovery programmes.

**Scheme 4:**
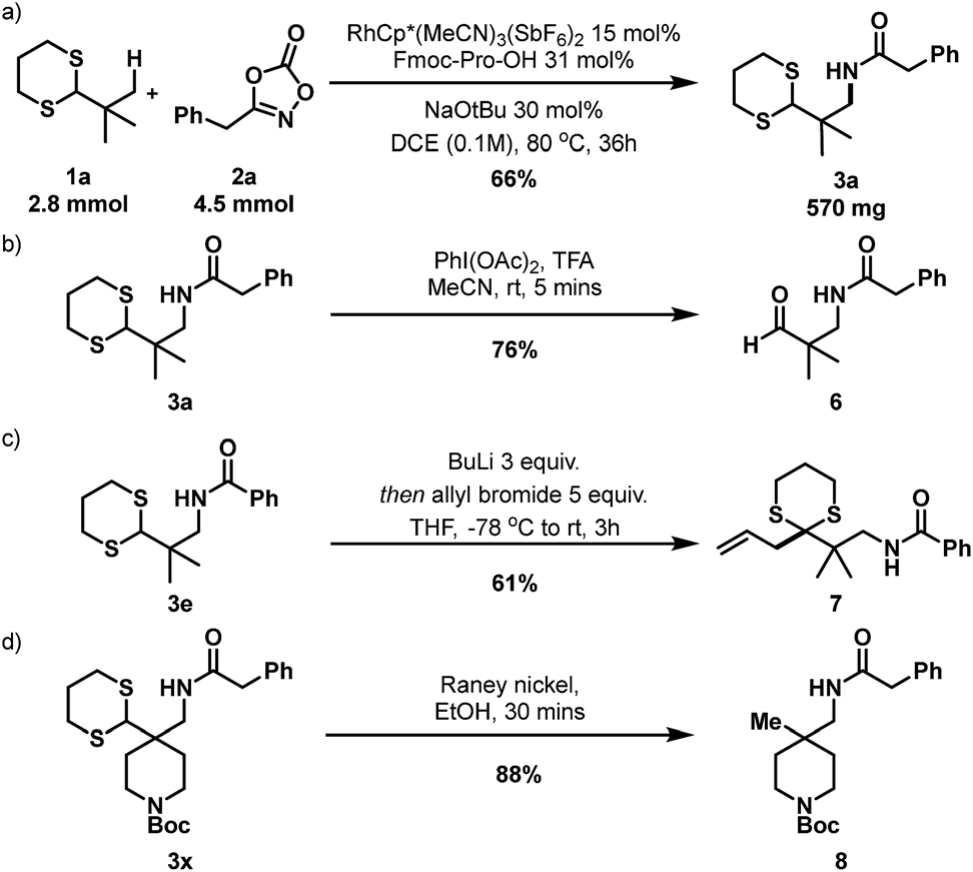
(a) 56-fold scale-up reaction. (b) Oxidative deprotection of dithiane. (c) Corey–Seebach alkylation with allyl bromide. (d) Reductive desulfurization.

## Conclusions

We have discovered and developed a Cp*Rh(iii) catalyzed C–H amidation of unactivated C(sp^3^)–H bonds utilizing the synthetically versatile dithiane as a directing group. The method is tolerant of a diverse range of aryl and alkyl dioxazolones as well as modifications on the dithiane partner. The work constitutes a rare example of Rh(iii) catalyzed C(sp^3^)–H activation of unreactive C–H bonds, and first use of dithianes for such alkyl C–H bonds. Varied modifications of the products exploiting the versatility of the dithiane group were also performed, demonstrating the high synthetic value of this method for use within industry and academia.

## Conflicts of interest

The authors declare no competing interests.

## Supplementary Material

Supplementary information
