# Supplementary material for: Dithiane-directed Rh(iii)-catalyzed amidation of unactivated C(sp3)–H bonds
Source: Chem Sci. 2019 Feb 18;10(13):3733–7. doi: 10.1039/c8sc05225e (PMC6457195; doi:10.1039/c8sc05225e)
Supplement: Supplementary file 1 [file SC-010-C8SC05225E-s001.pdf]

Electronic Supplementary Material (ESI) for Chemical Science.
This journal is © The Royal Society of Chemistry 2019

Supporting Information

Dithiane-Directed Rh(III)-Catalyzed Amidation of Unactivated C(sp^3)-H Bonds

Heyao Shi, Darren J. Dixon*

Department of Chemistry

Chemistry Research Laboratory

University of Oxford

Mansfield Road

Oxford, OX1 3TA

Email: darren.dixon@chem.ox.ac.uk

Contents

1. General Methods and Materials 6

2. Appendix 7

2.1. Time Studies 7

2.2. Unsuccessful Electrophile Examples 7

2.3. Poorly Reactive Substrate Examples 7

Experimental Procedures 8

1.1. General Procedure A: Synthesis of Dioxazolones from Acid Chlorides 8

1.1.1. Compound 2a 8

1.1.2. Compound 2b 8

1.1.3. Compound 2c 8

1.1.4. Compound 2d 8

1.1.5. Compound 2e 9

1.1.6. Compound 2f 9

1.1.7. Compound 2g 9

1.1.8. Compound 2h 9

1.1.9. Compound 2i 10

1.1.10. Compound 2j 10

1.1.11. Compound 2k 10

1.1.12. Compound 2l 10

1.1.13. Compound 2m 11

1.1.14. Compound 2n 11

1.1.15. Compound 2o 11

1.2. General Procedure C: Synthesis of Dithianes 11

1.2.1. Compound 1a 11

1.2.2. Compound 1b 12

1.2.3. Compound 1c 12

1.2.4. Compound 1d 12

1.2.5. Compound 1e 13

1.2.6. Compound 1f 13

1.2.7. Compound 1g 13

1.2.8. Compound 1h 13

1.2.9. Compound 1i 14

1.2.10. Compound 1j 14
1.3. General Procedure C: Synthesis of Amidated Dithianes 3 14
1.3.1. Compound 3a 15
1.3.2. Compound 3b 15
1.3.3. Compound 3c 15
1.3.4. Compound 3d 16
1.3.5. Compound 3e 16
1.3.6. Compound 3f 16
1.3.7. Compound 3g 17
1.3.8. Compound 3h 17
1.3.9. Compound 3i 17
1.3.10. Compound 3j 18
1.3.11. Compound 3k 18
1.3.12. Compound 3l 18
1.3.13. Compound 3m 19
1.3.14. Compound 3n 19
1.3.15. Compound 3o 19
1.3.16. Compound 3p 20
1.3.17. Compound 3q 20
1.3.18. Compound 3r 20
1.3.19. Compound 3s 21
1.3.20. Compound 3t 21
1.3.21. Compound 3u 21
1.3.22. Compound 3v 22
1.3.23. Compound 3w 22
1.3.24. Compound 3x 22
1.4. Derivatisation 23
1.4.1. Compound 6 23
1.4.2. Compound 7 23
1.4.3. Compound 8 24
3. NMR Spectra 25
3.1. Compound 2b 25
3.2. Compound 2c 28
3.3. Compound 3g 30
3.4. Compound 2g 32

3.5. Compound 2i 34
3.6. Compound 2j 36
3.7. Compound 2k 38
3.8. Compound 2l 40
3.9. Compound 2m 42
3.10. Compound 2n 44
3.11. Compound 2o 46
3.12. Compound 2b 48
3.13. Compound 1c 50
3.14. Compound 1d 53
3.15. Compound 1e 56
3.16. Compound 1f 58
3.17. Compound 1g 60
3.18. Compound 1h 62
3.19. Compound 1i 64
3.20. Compound 1j 66
3.21. Compound 3a 68
3.22. Compound 3b 70
3.23. Compound 3c 73
3.24. Compound 3d 75
3.25. Compound 3e 77
3.26. Compound 3f 79
3.27. Compound 3g 81
3.28. Compound 3h 83
3.29. Compound 3i 85
3.30. Compound 3j 87
3.31. Compound 3k 89
3.32. Compound 3l 91
3.33. Compound 3m 93
3.34. Compound 3n 95
3.35. Compound 3o 97
3.36. Compound 3p 99
3.37. Compound 3q 101
3.38. Compound 3r 104
3.39. Compound 3s 107

3.40. Compound 3u 111
3.41. Compound 3w 115
3.42. Compound 3x 117
3.43. Compound 6 119
3.44. Compound 7 121
3.45. Compound 8 123

1. General Methods and Materials

Reactions were carried out under a nitrogen atmosphere in oven-dried glassware at room temperature (rt = 20 - 25 ^{o}C) unless stated otherwise. Standard inert atmosphere techniques were used in handling all air and moisture sensitive reagents. Thin-layer chromatography (TLC) was performed using Merck aluminium backed sheets coated with Merck Kieselgel 60 F254 (230-400 mesh) fluorescent treated silica, which were visualised under UV light (λmax= 254 or 365 nm). Flash column chromatography was performed using Merck Kieselgel (230-400 mesh). All 1H and 13C NMR spectra were recorded using a Bruker 500 MHz and Bruker 400 MHz spectrometers and are quoted in ppm for measurement against a tetramethylsilane (TMS) or residual solvent peak internal standard. Coupling constants (J) are reported in hertz (Hz), and rounded to the nearest 0.5Hz. Two-dimensional spectroscopy (COSY, HSQC and HMBC) was used to assist in the assignment and the data is not reported. IR spectra were recorded on a Bruker Tensor 27 FT-IR spectrometer deposited as a thin film. Melting points were recorded using a Leica Galen III hot-stage microscope apparatus and are reported uncorrected in degrees Celsius (oC). Low resolution mass spectra were recorded on a Waters LCT premier XE Micromass spectrometer (ESI). High resolution mass spectra (ESI) were recorded on a Bruker MicroTof mass spectrometer. Optical rotations were recorded using a Perkin Elmer 341 polarimeter; [α]D T values are reported in 10-1 deg·cm2 ·g-1 ; concentrations (c) are quoted in g/100 mL; D refers to the D-line of sodium (589 nm); temperatures (T) are given in degrees Celsius (°C). (+) and (–) compound number prefixes indicate the sign of the optical rotation. The enantiomeric excesses were determined by HPLC analysis on an Agilent 1200 Series instrument or by GC analysis on an Agilent 7820A instrument employing a chiral stationary phase column specified in the individual experiment and by comparing the samples with the appropriate racemic mixtures. Commercial grade reagents and solvents were used without further purification. Anhydrous toluene, tetrahydrofuran and dichloromethane were obtained by filtration through activated alumina (powder ~150 mesh, pore size 58Å, basic, Sigma-Aldrich) columns. Other solvents were used directly as received from commercial suppliers.

2. Appendix

2.1. Time Studies

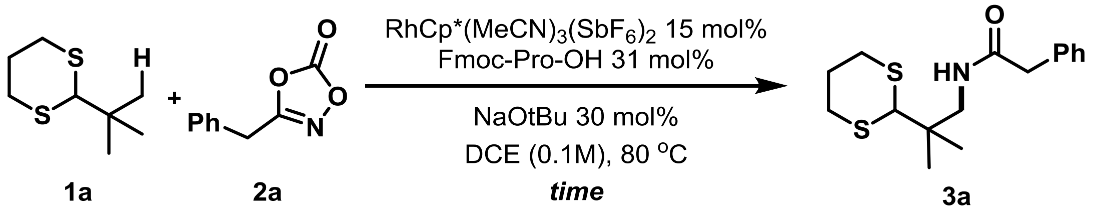

Entry	Time (h)	Yield (%)
1	16	63
2	24	66
3	36	73(71)

Table 1: Reaction time optimisation.

2.2. Unsuccessful Electrophile Examples

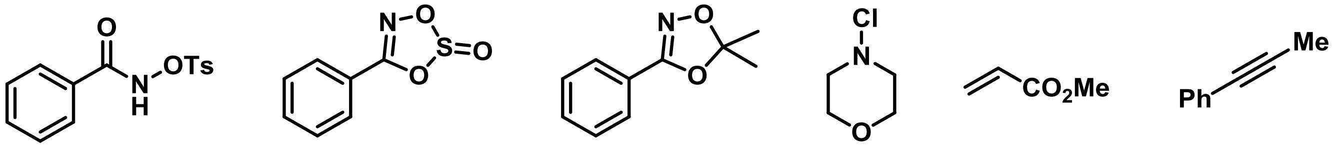

Figure 1: Unsuccessful electrophiles with optimised C-H functionalisation conditions

2.3. Poorly Reactive Substrate Examples

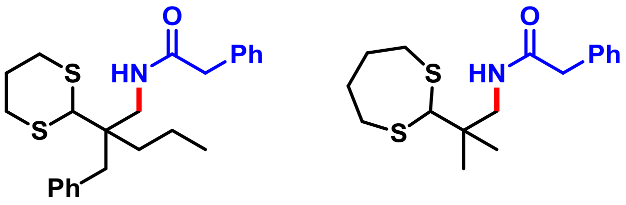

3y, 27% **3z, 16%**

Figure 3: Poorly reactive substrate examples.

Experimental Procedures

1.1. General Procedure A: Synthesis of Dioxazolones from Acid Chlorides

A flask was charged with hydroxylamine hydrochloride (1 equivalent), potassium carbonate (1 equivalent) and solvent (diethyl ether/water; 7.5:1). The reaction was cooled to 0 ^{o}C with vigourous stirring before addition of acid chloride (1 equivalent) dropwise. The reaction was stirred overnight, diluted with water and extracted with ethyl acetate. The combined organic layers were washed with brine, dried over anhydrous sodium sulfate and concentrated *in vacuo*. The crude hydroxamic acid product was purified by trituration with pentane/diethyl ether if necessary.

To a slurry of hydroxamic acid (1 equivalent) in dichloromethane was added carbonyl diimidazole (CDI, 1.5 equivalents). The reaction was stirred for 30 minutes, diluted with 1M aqueous HCl and extracted with dichloromethane. The combined organic layers were dried over sodium sulfate, and concentrated *in vacuo* to give dioxazolone **2**. Purification by dissolving in toluene and filtration was performed in necessary.

1.1.1. Compound 2a

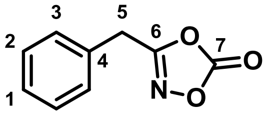

^{1}H NMR ($CDCl_3$, 400 MHz) δ_H 7.42 – 7.33 (m, 3H, $H^{1,2}$), 7.32 – 7.28 (m, 2H, H^3), 3.93 (s, 2H, H^5)
^{13}C NMR ($CDCl_3$, 100 MHz) δ_C 165.4 (C^6), 154.1 (C^7), 130.6 (C^4), 129.4 (C^3), 129.1 (C^2), 128.6 (C^1), 31.4 (C^5).

Data matches previous literature report. V. Bizet, L. Buglioni, C. Bolm, *Angew. Chem. Int. Ed.* **2014**, *53*, 5639.

1.1.2. Compound 2b

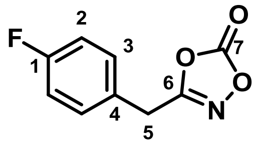

mp 39-40 ^{o}C
IR (film)/cm^{-1} ν_{max} 2917 (C-H), 2849 (C-H), 1861 (C=O), 1828 (C=N).
^{1}H NMR ($CDCl_3$, 400 MHz) δ_H 7.33 – 7.23 (m, 2H, H^2), 7.11 – 7.05 (m, 2H, H^3), 3.91 (s, 2H, H^5).
^{13}C NMR ($CDCl_3$, 100 MHz) δ_C 165.3 (C^7), 162.9 (d, J = 248 Hz, C^1), 153.9 (C^6), 130.9 (d, J = 8.3 Hz, C^3), 126.2 (d, J = 3.5 Hz, C^4), 116.3 (d, J = 21.9 Hz, C^2), 30.6 (C^5).
^{19}F NMR ($CDCl_3$, 400 MHz) δ_F -113.1.
HRMS (EI^+) exact mass calculated for [M]+ ($C_9H_6FNO_3$) requires m/z 195.0326, found m/z 195.0330.

1.1.3. Compound 2c

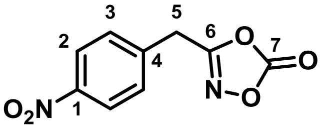

mp 89-94 ^{o}C.
IR (film)/cm^{-1} ν_{max} 2981 (C-H stretch), 1866 (C=O stretch), 1837 (C=N stretch), 1520 (N-O stretch).
^{1}H NMR ($CDCl_3$, 400 MHz) δ_H 8.27 (d, *J* = 8.7 Hz, 1H, H^2), 7.53 – 7.49 (m, 2H, H^3), 4.07 (s, 2H, H^5).
^{13}C NMR ($CDCl_3$, 100 MHz) δ_C 164.2 (C^6), 153.5 (C^7), 148.2 (C^1), 137.5 (C^4), 130.3 (C^2), 124.6 (C^3), 31.1 (C^5).
HRMS (EI^+) exact mass calculated for [M+Na]+ ($C_9H_6N_2O_5$) requires m/z 222.0271, found m/z 222.0278.

1.1.4. Compound 2d

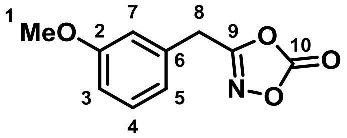

IR (film)/cm^{-1} $\mathbf{\nu_{max}}$ 2972 (C-H stretch), 1860 (C=O stretch), 1828 (C=N stretch), 1600 (C=C stretch).
^{1}H NMR ($CDCl_3$, 400 MHz) δ_H 7.30 (dd, *J* = 8.3, 7.6 Hz, 1H, H^4), 6.92 – 6.84 (m, 1H, $H^{3, 5}$), 6.82 (t, *J* = 2.1 Hz, 1H, H^7), 3.90 (s, 2H, H^8), 3.82 (s, 3H, H^1).
^{13}C NMR ($CDCl_3$, 100 MHz) δ_C 165.4 (C^9), 160.3 (C^{10}), 154.0 (C^2), 131.9 (C^6), 130.5 (C^4), 121.3 (C^5), 115.0 (C^7), 113.9 (C^3), 55.5 (C^1), 31.4 (C^8).
HRMS (EI^+) exact mass calculated for [M]+ ($C_{10}H_9NO_4$) requires m/z 207.0526, found m/z 207.0531.

1.1.5. Compound 2e

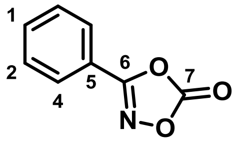

^{1}H NMR ($CDCl_3$, 400 MHz) δ_H 7.90 – 7.83 (m, 2H, H^4), 7.69 – 7.61 (m, 1H, H^1), 7.59 – 7.50 (m, 1H, H^2).
^{13}C NMR ($CDCl_3$, 100 MHz) δ_C 163.7 (C^6), 154.0 (C^7), 133.9 (C^1), 129.5 (C^4), 126.8 (C^2), 120.3 (C^5).

Matches literature report: Bolm *et al. Angew. Chem. Int. Ed.* **2014**, *53*, 5639 –5642

1.1.6. Compound 2f

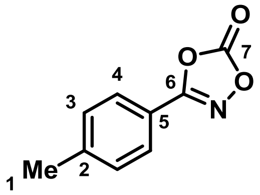

^{1}H NMR ($CDCl_3$, 400 MHz) δ_H 7.77 – 7.70 (m, 2H, H^4), 7.34 (d, *J* = 8.1 Hz, 2H, H^3), 2.45 (s, 3H, H^1).
^{13}C NMR ($CDCl_3$, 100 MHz) δ_C δ 163.8 (C^6), 154.1 (C^7), 145.0 (C^2), 130.2(C^3), 126.7(C^4), 117.4 (C^5), 21.9 (C^1).

Matches literature report: Bolm *et al. Angew. Chem. Int. Ed.* **2014**, *53*, 5639 –5642.

1.1.7. Compound 2g

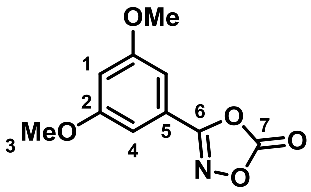

mp 89-92 °C.
IR (film)/cm^{-1} $\mathbf{\nu_{max}}$ 2981 (C-H stretch), 1824 (C=O stretch), 1585 (C=C stretch).
^{1}H NMR ($CDCl_3$, 400 MHz) δ_H 6.96 (d, *J* = 2.3 Hz, 2H, H^4), 6.69 (t, *J* = 2.3 Hz, 1H, H^1), 3.84 (s, 6H, H^3).
^{13}C NMR ($CDCl_3$, 100 MHz) δ_C 163.7 (C^6), 161.5 (C^7), 153.9 (C^2), 121.7 (C^5), 106.3 (C^1), 104.5 (C^4), 55.9 (C^3).
HRMS (EI^+) exact mass calculated for [M]+ ($C_{10}H_9NO_5$) requires m/z 223.0475, found m/z 223.0481.

1.1.8. Compound 2h

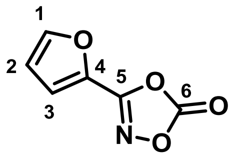

^{1}**H NMR** ($CDCl_3$, 400 MHz) δ_H 7.72 (dd, *J* = 1.8, 0.8 Hz, 1H, H^{3}), 7.19 (dd, *J* = 3.6, 0.8 Hz, 1H, H^{1}), 6.66 (dd, *J* = 3.6, 1.8 Hz, 1H, H^{2}).
^{13}**C NMR** ($CDCl_3$, 100 MHz) δ_C 156.7 (C^{5}), 153.2 (C^{6}), 147.9 (C^{3}), 135.2 (C^{4}), 117.9 (C^{1}), 112.7 (C^{2}).

Matches literature report: Bolm *et al. Angew. Chem. Int. Ed.* **2014**, *53*, 5639 –5642.

1.1.9. Compound 2i

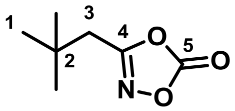

IR (film)/cm^{-1} $\boldsymbol{\nu}_{\mathbf{max}}$ 2964 (C-H stretch), 1878 (C=O stretch), 1832 (C=N stretch), 1630 (N-O) stretch.
^{1}**H NMR** ($CDCl_3$, 400 MHz) δ_H 2.50 (s, 2H, H^{3}), 1.07 (s, 9H, H^{1}).
^{13}**C NMR** ($CDCl_3$, 100 MHz) δ_C 165.7 (C^{4}), 154.4 (C^{5}), 38.5 (C^{3}), 31.4 (C^{2}), 29.5 (C^{1}).

1.1.10. Compound 2j

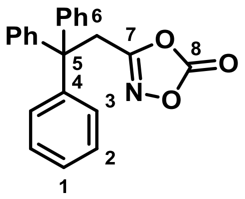

mp 98-101 ^{o}C
IR (film)/cm^{-1} $\boldsymbol{\nu}_{\mathbf{max}}$ 1867 (C=O stretch), 1828 (C=N stretch), 1627 (C=C stretch).
^{1}**H NMR** ($CDCl_3$, 400 MHz) δ_H 7.38 – 7.15 (m, 15H, $H^{1, 2, 3, 4}$), 3.97 (s, 2H, H^{6}).
^{13}**C NMR** ($CDCl_3$, 100 MHz) δ_C 165.0 (C^{7}), 153.8 (C^{8}), 144.8 (C^{4}), 128.9 (C^{3}), 128.5 (C^{2}), 127.2 (C^{1}), 56.3 (C^{5}), 37.1 (C^{6}).
HRMS (EI^{+}) exact mass calculated for [M]+ ($C_{22}H_{17}NO_3$) requires m/z 343.1203, found m/z 343.1209.

1.1.11. Compound 2k

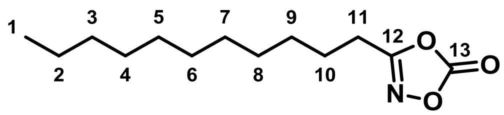

IR (film)/cm^{-1} $\boldsymbol{\nu}_{\mathbf{max}}$ 2924 (C-H stretch), 1869 (C=O stretch), 1829 (C=N stretch), 1637 (N-O stretch).
^{1}**H NMR** ($CDCl_3$, 400 MHz) δ_H 2.64 – 2.58 (m, 2H, H^{11}), 1.79 – 1.66 (m, 2H, H^{10}), 1.46 – 1.22 (m, 16H, $H^{2, 3, 4, 5, 6, 7, 8, 9}$), 0.93 – 0.83 (m, 3H, H^{1}).
^{13}**C NMR** ($CDCl_3$, 100 MHz) δ_C 166.9 (C^{12}), 154.4 (C^{13}), 32.0 ($C^{2/3/4/5/6/7/8/9}$), 29.7 ($C^{2/3/4/5/6/7/8/9}$), 29.7 ($C^{2/3/4/5/6/7/8/9}$), 29.6 ($C^{2/3/4/5/6/7/8/9}$), 29.4 ($C^{2/3/4/5/6/7/8/9}$), 29.1 ($C^{2/3/4/5/6/7/8/9}$), 28.8 ($C^{2/3/4/5/6/7/8/9}$), 24.9 (C^{11}), 24.7 (C^{10}), 22.8 ($C^{2/3/4/5/6/7/8/9}$), 14.2 (C^{1}).

1.1.12. Compound 2l

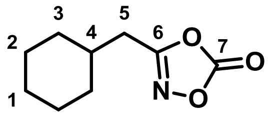

IR (film)/cm^{-1} $\boldsymbol{\nu}_{\mathbf{max}}$ 2926 (C-H stretch), 1859 (C=O stretch), 1826 (C=N stretch), 1633 (N-O stretch).
^{1}**H NMR** ($CDCl_3$, 400 MHz) δ_H 2.50 (d, *J* = 6.7 Hz, 2H, H^{5}), 1.83 – 1.64 (m, 6H, $H^{4, 1a, 2a, 3a}$), 1.36 – 1.10 (m, 3H, $H^{2b, 3b}$), 1.05 (qd, *J* = 11.3, 10.1, 5.6 Hz, 1H, H^{1b}).

^{13}C NMR ($CDCl_3$, 100 MHz) δ_C 166.1 (C^6), 154.4 (C^7), 34.7 (C^4), 32.8 (C^3), 32.2 (C^5), 25.9 (C^1), 25.8 (C^2).

1.1.13. Compound 2m

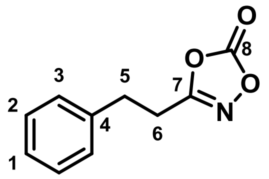

IR (film)/cm^{-1} $\mathbf{\nu_{max}}$ 2981 (C-H stretch), 1867 (C=O stretch), 1827 (C=N stretch).
^{1}H NMR ($CDCl_3$, 400 MHz) δ_H 7.38 – 7.32 (m, 2H, H^2), 7.31 – 7.25 (m, 1H, H^1), 7.24 – 7.20 (m, 2H, H^3), 3.09 – 3.02 (m, 2H, H^6), 2.98 – 2.92 (m, 2H, H^5)
^{13}C NMR ($CDCl_3$, 100 MHz) δ_C 166.0 (C^7), 154.1 (C^8), 138.1 (C^4), 129.0 (C^2), 128.3 (C^3), 127.3 (C^1), 30.6 (C^6), 26.8 (C^5).
HRMS (EI^+) exact mass calculated for [M]+ ($C_{10}H_9NO_3$) requires m/z 191.0577, found m/z 191.0578.

1.1.14. Compound 2n

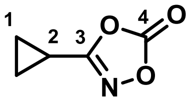

IR (film)/cm^{-1} $\mathbf{\nu_{max}}$ 2917 (C-H stretch), 1881 (C=O stretch), 1827 (C=N stretch), 1630 (N-O stretch).
^{1}H NMR ($CDCl_3$, 400 MHz) δ_H 1.90 (tt, J = 8.3, 5.1 Hz, 1H, H^2), 1.22 – 1.12 (m, 4H, H^1)
^{13}C NMR ($CDCl_3$, 100 MHz) δ_C 168.0 (C^3), 154.1 (C^4), 7.4 (C^1), 5.5 (C^2).
HRMS (EI^+) exact mass calculated for [M]+ ($C_5H_5NO_3$) requires m/z 127.0264, found m/z 127.0264.

1.1.15. Compound 2o

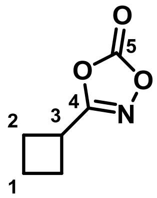

IR (film)/cm^{-1} $\mathbf{\nu_{max}}$ 2954 (C-H stretch), 1871 (C=O stretch), 1827 (C=N stretch), 1626 (N-O stretch).
^{1}H NMR ($CDCl_3$, 400 MHz) δ_H 3.47 (pd, J = 8.4, 1.1 Hz, 1H, H^3), 2.43 – 2.35 (m, 4H, H^2), 2.21 – 2.00 (m, 2H, H^1)
^{13}C NMR ($CDCl_3$, 100 MHz) δ_C 168.4 (C^4), 154.5 (C^5), 29.5 (C^3), 25.2 (C^2), 19.0 (C^1).
HRMS (EI^+) exact mass calculated for [M]+ ($C_6H_7NO_3$) requires m/z 141.0420, found m/z 141.0419.

1.2. General Procedure C: Synthesis of Dithianes

To a solution of aldehyde (1 equivalent) in dichloromethane cooled to 0 ^{o}C, was added potassium tert-butoxide (1.3 equivalents), followed by dropwise addition of electrophile (2 equivalents). The reaction was warmed to room temperature and stirred overnight, followed by addition of 1M HCl. The reaction was extracted with dichloromethane, dried over anhydrous sodium sulfate and concentrated in vacuo. The crude product was purified by flash column chromatography to α-methylated aldehyde.
To a solution of α-methylated aldehyde in toluene, was pTSA.H2O (0.05 equivalents) and propane dithiol (1.3 equivalents) sequentially. The reaction was purged with nitrogen and heated to 80 ^{o}C until full consumption of the aldehyde. The reaction was diluted with diethyl ether and washed twice with 1M aqueous KOH. The organic layers were dried over anhydrous sodium sulfate and concentrated in vacuo. The crude product was purified by flash column chromatography to give desired dithiane.

1.2.1. Compound 1a

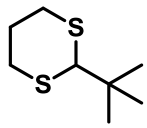

Prepared according to an analogous procedure to General Procedure C. To a solution of trimethylacetaldehyde (3 mL, 27.62 mmol) in toluene (50 mL) was added para-toluenesulfonic acid monohydrate (263 mg, 0.05 eq.), followed by 1,3-propanedithiol (3.61 mL, 1.3 eq.). The reaction was purged with nitrogen and heated to 80 ^{o}C overnight. The reaction mixture was diluted with diethyl ether (100 mL) and washed three times with 1M aqueous KOH. The organic layers were washed with brine, dried over anhydrous sodium sulfate and concentrated in vacuo. The crude oil was seeded with a crystal of product, and the crystals were collected and washed with ice methanol to give **1a** as a clear crystalline solid, 4.70g, 96%.

1.2.2. Compound 1b

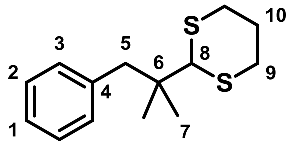

Clear crystalline solid, 23%.
mp 36-40 ^{o}C.
IR (film)/cm^{-1} $\mathbf{\nu_{max}}$ 2964 (C-H stretch), 1493 (C-S stretch), 1422 (C=C stretch).
^{1}H NMR ($CDCl_3$, 400 MHz) δ_H 7.31 – 7.23 (m, 5H, $H^{1,2,3}$), 3.93 (s, 1H, H^8), 2.96 – 2.82 (m, 4H, H^9), 2.81 (s, 2H, H^5), 2.08 (dtt, *J* = 13.6, 3.8, 2.8 Hz, 1H, H^{10a}), 1.84 (dtt, *J* = 14.0, 11.2, 4.6 Hz, 1H, H^{10b}), 1.09 (s, 6H, H^7).
^{13}C NMR ($CDCl_3$, 100 MHz) δ_C 138.4 (C^4), 130.8 (C^3), 128.0 (C^2), 126.3 (C^1), 60.4 (C^8), 45.7 (C^5), 39.6 (C^6), 31.5 (C^9), 26.3 (C^{10}), 25.5 (C^7).

1.2.3. Compound 1c

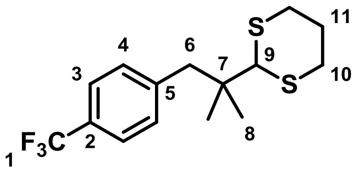

Clear crystalline solid, 19%.
mp 58-59 ^{o}C.
IR (film)/cm^{-1} $\mathbf{\nu_{max}}$ 2964 (C-H stretch), 2935 (C-H stretch), 2899 (C-H stretch), 1324.
^{1}H NMR ($CDCl_3$, 400 MHz) δ_H 7.54 (d, *J* = 8.0 Hz, 2H, H^3), 7.40 – 7.37 (m, 2H, H^4), 3.88 (s, 1H, H^9), 2.95 – 2.82 (m, 6H, $H^{6, 10}$), 2.09 (dtt, *J* = 13.7, 3.9, 2.8 Hz, 1H, H^{11a}), 1.84 (dtt, *J* = 14.0, 11.6, 4.1 Hz, 1H, H^{11b}), 1.09 (s, 6H, H^8).
^{13}C NMR ($CDCl_3$, 100 MHz) δ_C 142.6 (C^5), 131.0 (C^4), 128.9 (C^2), 125.9 (C^1), 124.9 (C^3), 60.1 (C^9), 45.5 (C^{10}), 39.7 (C^7), 31.4 (C^6), 26.2 (C^{11}), 25.5 (C^8).
^{19}F NMR ($CDCl_3$, 400 MHz) δ_F -62.34.
HRMS (APCI$^+$) exact mass calculated for [M+H]+ ($C_{15}H_{20}F_3{}^{32}S_2$) requires m/z 321.0953, found m/z 321.0957.

1.2.4. Compound 1d

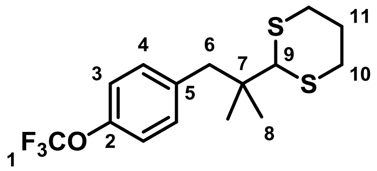

Oil, 17%.
IR (film)/cm^{-1} $\mathbf{\nu_{max}}$ 2968 (C-H stretch), 2899 (C-H stretch), 1508 (C-S stretch).

^{1}H NMR ($CDCl_3$, 400 MHz) δ_H 7.30 – 7.27 (m, 2H, H^4), 7.12 (dq, *J* = 7.6, 1.0 Hz, 2H, H^3), 3.88 (s, 1H, H^9), 2.94 – 2.81 (m, 4H, H^{10}), 2.81 (s, 2H, H^6), 2.09 (dtt, *J* = 13.6, 3.9, 2.8 Hz, 1H, H^{11a}), 1.83 (dtt, *J* = 14.0, 11.4, 4.4 Hz, 1H, H^{11b}), 1.08 (s, 6H, H^8).
^{13}C NMR ($CDCl_3$, 100 MHz) δ_C 148.00 (q, *J* = 2.0 Hz, C^2), 137.09 (C^5), 132.0 (C^4), 120.7 (q, J = 257 Hz, C^1), 120.4 (C^3), 60.1 (C^9), 45.0 (C^6), 39.6 (C^7), 31.4 (C^{10}), 26.2 (C^{11}), 25.5 (C^8).
^{19}F NMR ($CDCl_3$, 400 MHz) δ_F -57.82.
HRMS ($APCI^+$) exact mass calculated for [M+H]+ ($C_{15}H_{20}OF_3{}^{32}S_2$) requires m/z 337.0902, found m/z 337.0906.

1.2.5. Compound 1e

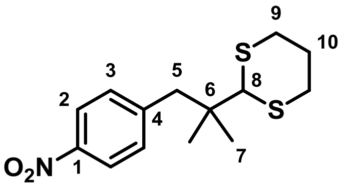

Off-white solid, 15%.
mp 73-77 ^{o}C.
IR (film)/cm^{-1} ν_{max} 2963 (C-H stretch), 2931 (C-H stretch), 1517 (C-S stretch), 1345 (N-O stretch).
^{1}H NMR ($CDCl_3$, 400 MHz) δ_H 8.17 – 8.12 (m, 2H, H^2), 7.46 – 7.42 (m, 2H, H^3), 3.84 (s, 1H, H^8), 2.95 – 2.81 (m, 6H, $H^{5,\,9}$), 2.10 (dtt, *J* = 13.7, 4.0, 2.7 Hz, 1H, H^{10a}), 1.83 (dtt, *J* = 14.0, 11.9, 3.8 Hz, 1H, H^{10b}), 1.10 (s, 6H, H^7).
^{13}C NMR ($CDCl_3$, 100 MHz) δ_C 146.9 (C^1), 146.4 (C^4), 131.5 (C^3), 123.2 (C^2), 60.0 (C^8), 45.5 (C^5), 39.9 (C^9), 31.4 (C^6), 26.1 (C^{10}), 25.6 (C^7).
HRMS ($APCI^+$) exact mass calculated for [M+H]+ ($C_{14}H_{20}O_2N^{32}S_2$) requires m/z 298.0930, found m/z 298.0934.

1.2.6. Compound 1f

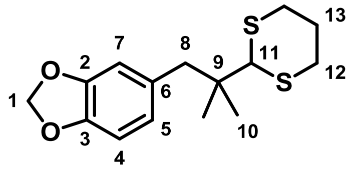

Oil, 21%.
IR (film)/cm^{-1} ν_{max} 2961 (C-H stretch), 2895 (C-H stretch), 1501 (C-S stretch), 1488 (C=C stretch).
^{1}H NMR ($CDCl_3$, 400 MHz) δ_H 6.80 – 6.77 (m, 1H, H^7), 6.76 – 6.68 (m, 2H, $H^{4,\,5}$), 5.93 (s, 2H, H^1), 3.91 (s, 1H, H^{11}), 2.93 – 2.81 (m, 4H, H^{12}), 2.72 (s, 2H, H^8), 2.14 – 2.02 (m, 1H, H^{13a}), 1.82 (dtt, *J* = 14.0, 10.9, 4.8 Hz, 1H, H^{13b}), 1.07 (s, 6H, H^{10}).
^{13}C NMR ($CDCl_3$, 100 MHz) δ_C 147.3 (C^2), 146.1 (C^3), 132.0 (C^6), 123.7 (C^5), 111.1 (C^7), 107.8 (C^4), 100.9 (C^1), 60.2 (C^{11}), 45.4 (C^8), 39.6 (C^9), 31.4 (C^{12}), 26.2 (C^{13}), 25.5 (C^{10}).

1.2.7. Compound 1g

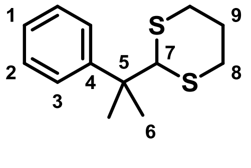

Oil, 52%.
IR (film)/cm^{-1} ν_{max} 2971 (C-H stretch), 2893 (C-H stretch), 1496 (C-S stretch).
^{1}H NMR ($CDCl_3$, 400 MHz) δ_H 7.46 – 7.38 (m, 2H, H^3), 7.35 (ddd, *J* = 7.8, 6.8, 1.2 Hz, 2H, H^2), 7.30 – 7.21 (m, 1H, H^1), 4.38 (s, 1H, H^7), 2.91 – 2.77 (m, 4H, H^8), 2.08 – 1.9f7 (m, 1H, H^{9a}), 1.87 – 1.63 (m, 1H, H^{9b}), 1.55 (s, 6H, H^6).
^{13}C NMR ($CDCl_3$, 100 MHz) δ_C 146.7 (C^4), 128.2 (C^2), 126.7 (C^3), 126.3 (C^1), 61.9 (C^7), 42.8 (C^5), 31.6 (C^8), 26.4 (C^9), 26.0 (C^6).
HRMS (ES^+) exact mass calculated for [M+H]+ ($C_{13}H_{19}{}^{32}S_2$) requires m/z 239.0923, found m/z 239.0925.

1.2.8. Compound 1h

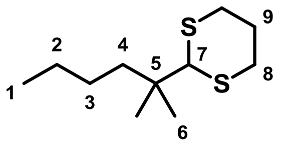

Oil, 27%.
IR (film)/cm^{-1} ν_{max} 2966 (C-H stretch), 1422.
^{1}H NMR ($CDCl_3$, 400 MHz) δ_H 4.02 (s, 1H, H^7), 2.94 – 2.81 (m, 4H, H^8), 2.11 – 2.00 (m, 1H, H^{9a}), 1.89 – 1.73 (m, 1H, H^{9b}), 1.48 – 1.40 (m, 2H, H^4), 1.36 – 1.20 (m, 4H, $H^{2,3}$), 1.07 (s, 6H, H^6), 0.91 (t, *J* = 6.9 Hz, 3H, H^1).
^{13}C NMR ($CDCl_3$, 100 MHz) δ_C 61.2 (C^7), 40.3 (C^4), 38.3 (C^5), 31.6 (C^8), 26.4 (C^9), 26.1 (C^3), 25.6 (C^6), 23.5 (C^2), 14.3 (C^1).
HRMS (ES^+) exact mass calculated for [M+Na]+ ($C_{19}H_{30}ON^{32}S_2$) requires m/z 352.1763, found m/z 352.1757.

1.2.9. Compound 1i

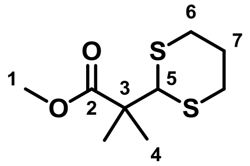

Oil, 95% (from aldehyde).
IR (film)/cm^{-1} ν_{max} 2981 (C-H stretch), 2948 (C-H stretch), 1731 (C=O stretch), 1464 (C-S stretch).
^{1}H NMR ($CDCl_3$, 400 MHz) δ_H 4.44 (s, 1H, H^5), 3.71 (s, 3H, H^1), 2.98 – 2.82 (m, 4H, H^6), 2.09 (dtt, *J* = 14.0, 4.2, 2.7 Hz, 1H, H^{7a}), 1.83 (dtt, *J* = 14.0, 11.7, 3.8 Hz, 1H, H^{7b}), 1.34 (s, 6H, H^4)
^{13}C NMR ($CDCl_3$, 100 MHz) δ_C 175.9 (C^2), 57.7 (C^5), 52.4 (C^1), 47.9 (C^3), 31.4 (C^6), 26.1 (C^7), 22.8 (C^4).
HRMS (ES^+) exact mass calculated for [M+Na]+ ($C_9H_{16}O_2{}^{23}Na^{32}S_2$) requires m/z 243.0484, found m/z 243.0484.

1.2.10. Compound 1j

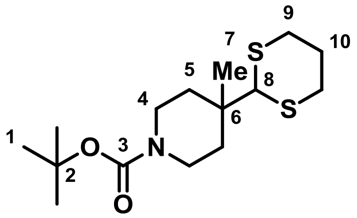

White solid, 47%.
mp 59-61 °C.
IR (film)/cm^{-1} ν_{max} 2966 (C-H stretch), 1688 (C=O stretch), 1453 (C-S stretch).
^{1}H NMR ($CDCl_3$, 400 MHz) δ_H 4.10 (s, 1H, H^8), 3.66 – 3.56 (m, 2H, H^{4a}), 3.17 (ddd, *J* = 13.5, 9.5, 3.6 Hz, 2H, H^{4b}), 2.92 – 2.83 (m, 4H, H^9), 2.13 – 2.05 (m, 1H, H^{10a}), 1.88 – 1.76 (m, 1H, H^{10b}), 1.71 (ddd, *J* = 13.8, 9.5, 4.3 Hz, 2H, H^{5a}), 1.55 – 1.48 (m, 2H, H^{5b}), 1.45 (s, 9H, H^1), 1.14 (s, 3H, H^7)
^{13}C NMR ($CDCl_3$, 100 MHz) δ_C 155.1 (C^3), 79.5 (C^2), 60.4 (C^8), 39.7 (C^4), 36.8 (C^6), 35.4 (C^5), 31.4 (C^9), 28.6 (C^7), 26.4 (C^{10}), 20.7 (C^1).

1.3. General Procedure C: Synthesis of Amidated Dithianes 3.

A 14 mL oven dried vial is charged with dithiane (0.05 mmol, 1 equivalent), $[Cp^*Rh(MeCN)_3][(SbF_6)_2]$ (0.15 equivalents), Fmoc-Pro-OH (0.31 equivalents), NaOtBu (0.30 equivalents) and dioxazolone (2 equivalents). The vial is sealed, dichloroethane (0.5 mL, 0.1 M) was added, the atmosphere replaced with nitrogen and heated to 80 °C. The reaction was left to stir for 36 hours, and the crude reaction mixture was loaded directly and purified by flash column chromatography to give amidodithianes.

1.3.1. Compound 3a

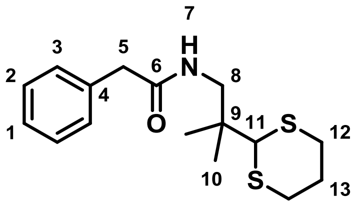

Off-white solid, 71%.
mp 111-113 °C.
IR (film)/cm^{-1} ν_{max} 3305 (N-H stretch), 2929 (C-H stretch), 1647 (C=O stretch), 1527.
^{1}H NMR ($CDCl_3$, 400 MHz) δ_H 7.41 – 7.35 (m, 2H, H^2), 7.33 – 7.28 (m, 3H, $H^{1,3}$), 5.80 (br s, 1H, H^7), 3.65 (s, 1H, H^{11}), 3.62 (s, 2H, H^5), 3.26 (d, *J* = 6.6 Hz, 2H, H^8), 2.76 (dt, *J* = 14.5, 3.7 Hz, 2H, H^{12a}), 2.64 (ddd, *J* = 14.5, 12.5, 2.5 Hz, 2H, H^{12b}), 2.01 (dtt, *J* = 13.6, 4.2, 2.5 Hz, 1H, H^{13a}), 1.70 (dtt, *J* = 13.9, 12.4, 3.3 Hz, 1H, H^{13b}), 1.00 (s, 6H, H^{10}).
^{13}C NMR ($CDCl_3$, 100 MHz) δ_C 171.3 (C^6), 135.4 (C^4), 129.8 (C^3), 129.2 (C^2), 127.4 (C^1), 58.7 (C^{11}), 47.5 (C^8), 44.3 (C^5), 40.1 (C^9), 31.4 (C^{12}), 26.0 (C^{13}), 23.9 (C^{10}).
HRMS (ES^+) exact mass calculated for [M+Na]+ ($C_{16}H_{23}ON^{23}Na^{32}S_2$) requires m/z 332.1113, found m/z 332.1109.

1.3.2. Compound 3b

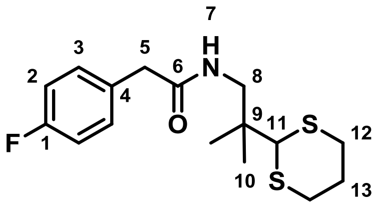

Off-white solid, 74%.
mp 104-105 °C.
IR (film)/cm^{-1} ν_{max} 3307 (N-H stretch), 2971 (C-H stretch), 1648 (C=O stretch), 1508 (C=C stretch).
^{1}H NMR ($CDCl_3$, 500 MHz) δ_H 7.30 – 7.25 (m, 2H, H^2), 7.09 – 7.04 (m, 2H, H^3), 5.84 (s, 1H, H^7), 3.74 (s, 1H, H^{11}), 3.58 (s, 2H, H^5), 3.27 (d, *J* = 6.6 Hz, 2H, H^8), 2.85 – 2.77 (m, 2H, H^{12a}), 2.70 (ddd, *J* = 14.6, 12.4, 2.4 Hz, 2H, H^{12b}), 2.05 (dtt, *J* = 13.6, 4.1, 2.5 Hz, 1H, H^{13a}), 1.72 (dtt, *J* = 14.1, 12.5, 3.3 Hz, 1H, H^{13b}), 1.02 (s, 6H, H^{10})
^{13}C NMR ($CDCl_3$, 125 MHz) δ_C 170.90 (C^6), 162.27 (d, *J* = 246.0 Hz, C^1), 131.38 (d, *J* = 7.9 Hz, C^3) 131.11 (d, *J* = 3.3 Hz, C^4), 116.01 (d, *J* = 21.4 Hz, C^2) , 58.9 (C^{11}), 47.5 (C^8), 43.4 (C^5), 40.1 (C^9), 31.5 (C^{12}), 26.0 (C^{13}), 24.2 (C^{10}).
^{19}F NMR ($CDCl_3$, 400 MHz) δ_F -115.3.
HRMS (ES^+) exact mass calculated for [M+H]+ ($C_{16}H_{23}ONF^{32}S_2$) requires m/z 328.1200, found m/z 328.1201.

1.3.3. Compound 3c

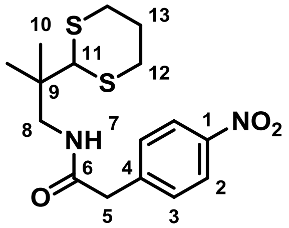

Yellow solid, 40%.
mp 96-101 °C.
IR (film)/cm^{-1} ν_{max} 3291 (N-H stretch), 3081 (C-H stretch), 1650 (C=O stretch), 1519 (N-O stretch).
^{1}H NMR ($CDCl_3$, 500 MHz) δ_H 8.23 – 8.20 (m, 2H, H^2), 7.52 – 7.48 (m, 2H, H^3), 6.01 (s, 1H, H^7), 3.88 (s, 1H, H^{11}), 3.68 (s, 2H, H^5), 3.31 (d, *J* = 6.5 Hz, 2H, H^8), 2.88 – 2.71 (m, 4H, H^{12}), 2.10 – 2.04 (m, 1H, H^{13a}), 1.72 (dtt, *J* = 14.1, 12.2, 3.7 Hz, 1H, H^{13b}), 1.05 (s, 6H, H^{10})
^{13}C NMR ($CDCl_3$, 125 MHz) δ_C 169.3 (C^6), 147.4 (C^1), 142.7 (C^4), 130.6 (C^3), 124.1 (C^2), 59.1 (C^{11}), 47.7 (C^8), 43.8 (C^5), 40.1 (C^9), 31.6 (C^{12}), 26.0 (C^{13}), 24.5 (C^{10}).
HRMS (ES^+) exact mass calculated for [M+Na]+ ($C_{16}H_{22}O_3N_2{}^{23}Na^{32}S_2$) requires m/z 377.0964, found m/z 377.0964.

1.3.4. Compound 3d

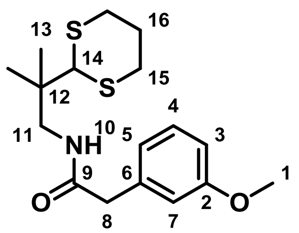

Off-white solid, 67%.
mp 96-99 °C.
IR (film)/cm^{-1} ν_{max} 3304 (N-H stretch), 2980 (C-H stretch), 1649 (N-H stretch), 1532 (C=C stretch).
^{1}**H NMR** ($CDCl_3$, 500 MHz) δ_H 7.30 (td, *J* = 7.5, 1.0 Hz, 1H, H^4), 6.90 – 6.82 (m, 3H, $H^{3,5,7}$), 5.84 (s, 1H, H^{10}), 3.81 (s, 3H, H^1), 3.66 (s, 1H, H^{14}), 3.59 (s, 2H, H^8), 3.26 (d, *J* = 6.6 Hz, 2H, H^{11}), 2.77 (dt, *J* = 14.2, 3.7 Hz, 2H, H^{15a}), 2.66 (ddd, *J* = 14.4, 12.4, 2.5 Hz, 2H, H^{15b}), 2.02 (dtt, *J* = 13.6, 4.2, 2.5 Hz, 1H, H^{16a}), 1.71 (dtt, *J* = 14.2, 12.5, 3.4 Hz, 1H, H^{16b}), 1.01 (s, 6H, H^{13}).
^{13}**C NMR** ($CDCl_3$, 100 MHz) δ_C 171.0 (C^9), 160.3 (C^2), 136.9 (C^6), 130.3 (C^4), 122.1 (C^3), 115.1 (C^7), 113.2 (C^5), 58.7 (C^{14}), 55.4 (C^1), 47.4 (C^{11}), 44.4 (C^8), 40.2 (C^{12}), 31.4 (C^{15}), 26.1 (C^{16}), 24.0 (C^{13}).
HRMS (ES^+) exact mass calculated for [M+Na]+ ($C_{17}H_{25}O_2N^{23}Na^{32}S_2$) requires m/z 362.1219, found m/z 362.1213.

1.3.5. Compound 3e

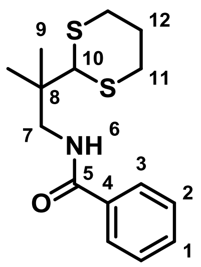

White solid, 49%.
mp 89-92 °C.
IR (film)/cm^{-1} ν_{max} 3328 (N-H stretch), 2971 (C-H stretch), 1643 (C=O stretch), 1537 (C=C stretch).
^{1}**H NMR** ($CDCl_3$, 400 MHz) 7.86 – 7.81 (m, 1H, H^3), 7.53 – 7.48 (m, 1H, H^1), 7.48 – 7.42 (m, 2H, H^2), 6.75 (s, 1H, H^6), 4.12 (s, 1H, H^{10}), 3.56 (d, *J* = 6.6 Hz, 2H, H^7), 3.05 – 2.86 (m, 4H, H^{11}), 2.17 – 2.09 (m, 1H, H^{12a}), 1.94 – 1.80 (m, 1H, H^{12b}), 1.18 (s, 6H, H^9).
^{13}**C NMR** ($CDCl_3$, 100 MHz) δ_C 167.6 (C^5), 134.9 (C^4), 131.5 (C^1), 128.7 (C^3), 127.1 (C^2), 59.5 (C^{10}), 47.9 (C^7), 40.4 (C^8), 31.7 (C^{11}), 26.2 (C^{12}), 24.6 (C^9).
HRMS (ES^+) exact mass calculated for [M+Na]+ ($C_{15}H_{21}ON^{23}Na^{32}S_2$) requires m/z 318.0957, found m/z 318.0958.

1.3.6. Compound 3f

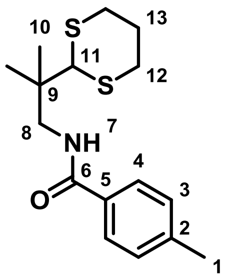

White solid, 41%.
mp 167-168 °C.
IR (film)/cm^{-1} ν_{max} 3348 (N-H stretch), 2980 (C-H stretch), 1642 (C=O stretch), 1503 (C=C stretch).
^{1}**H NMR** ($CDCl_3$, 500 MHz) δ_H 7.72 (d, *J* = 8.2 Hz, 2H, H^4), 7.25 (m, 2H, H^3), 6.69 (s, 1H, H^7), 4.11 (s, 1H, H^{11}), 3.55 (d, *J* = 6.6 Hz, 2H, H^8), 2.97 – 2.85 (m, 4H, H^{12}), 2.40 (s, 3H, H^1), 2.13 (app dq, *J* = 14.0, 3.2 Hz, 1H, H^{13a}), 1.91 – 1.81 (m, 1H, H^{13b}), 1.17 (s, 6H).
^{13}**C NMR** ($CDCl_3$, 125 MHz) δ_C 167.5 (C^6), 141.9 (C^2), 132.0 (C^5), 129.4 (C^3), 127.1 (C^4), 59.5 (C^{11}), 47.8 (C^8), 40.4 (C^9), 31.7 (C^{12}), 26.2 (C^{13}), 24.5 (C^{10}), 21.6 (C^1).

HRMS (ES^+) exact mass calculated for [M+Na]+ ($C_{16}H_{23}ON^{23}Na^{32}S_2$) requires m/z 332.1113, found m/z 332.1112.

1.3.7. Compound 3g

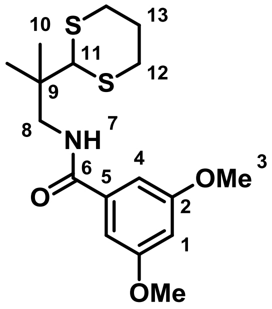

White solid, 55%.
mp 103-108 °C.
IR (film)/cm^{-1} ν_{max} 3332 (N-H stretch), 2933 (C-H stretch), 1649 (C=O stretch), 1593 (C=C stretch).
^{1}H NMR ($CDCl_3$, 400 MHz) δ_H 6.97 (d, J = 2.3 Hz, 2H, H^4), 6.67 (t, J = 6.9 Hz, 1H, H^7), 6.58 (t, J = 2.3 Hz, 1H, H^1), 4.11 (s, 1H, H^{11}), 3.83 (s, 6H, H^3), 3.53 (d, J = 6.6 Hz, 2H, H^8), 2.99 – 2.88 (m, 4H, H^{12}), 2.16 – 2.08 (m, 1H, H^{13a}), 1.93 – 1.77 (m, 1H, H^{13b}), 1.17 (s, 6H, H^{10}).
^{13}C NMR ($CDCl_3$, 100 MHz) δ_C 167.3 (C^6), 161.0 (C^2), 137.2 (C^5), 105.1 (C^4), 103.6 (C^1), 59.4 (C^{11}), 55.7 (C^3), 47.9 (C^8), 40.4 (C^9), 31.7 (C^{12}), 26.2 (C^{13}), 24.5 (C^{10}).
HRMS (ES^+) exact mass calculated for [M+H]+ ($C_{17}H_{26}O_3N^{32}S_2$) requires m/z 356.1349, found m/z 356.1343.

1.3.8. Compound 3h

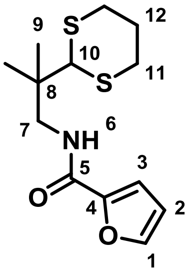

Pale yellow solid, 46%.
mp 137-139 °C.
IR (film)/cm^{-1} ν_{max} 3267 (N-H stretch), 2827 (C-H stretch), 1653 (C=O stretch), 1524 (C=C stretch).
^{1}H NMR ($CDCl_3$, 400 MHz) δ_H 7.48 – 7.44 (m, 1H, H^3), 7.11 (d, J = 3.5 Hz, 1H, H^1), 6.77 (s, 1H, H^6), 6.50 (dd, J = 3.5, 1.8 Hz, 1H, H^2), 4.11 (s, 1H, H^{10}), 3.51 (d, J = 6.8 Hz, 2H, H^7), 2.98 – 2.88 (m, 4H, H^{11}), 2.12 (dp, J = 13.6, 3.3 Hz, 1H, H^{12a}), 1.86 (dtt, J = 15.6, 8.8, 6.9 Hz, 1H, H^{12b}), 1.15 (s, 6H, H^9).
^{13}C NMR ($CDCl_3$, 100 MHz) δ_C 158.7 (C^5), 148.3 (C^4), 144.0 (C^3), 114.4 (C^1), 112.3 (C^2), 59.0 (C^{10}), 47.0 (C^7), 40.4 (C^8), 31.6 (C^{11}), 26.2 (C^{12}), 24.1 (C^9).
HRMS (ES^+) exact mass calculated for [M+Na]+ ($C_{13}H_{19}O_2N^{23}Na^{32}S_2$) requires m/z 308.0749, found m/z 308.0748.

1.3.9. Compound 3i

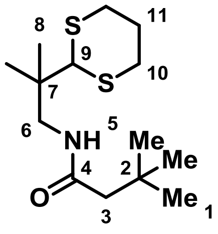

Off-white solid, 54%.
mp 136-139 °C.
IR (film)/cm^{-1} ν_{max} 3309 (N-H stretch), 2962 (C-H stretch), 1645 (C=O stretch), 1556 (C=C stretch).
^{1}H NMR ($CDCl_3$, 400 MHz) δ_H 5.81 (s, 1H, H^5), 4.03 (s, 1H, H^9), 3.33 (d, J = 6.7 Hz, 2H, H^6), 2.95 – 2.81 (m, 4H, H^{10}), 2.15 – 2.03 (m, 3H, $H^{3, 11a}$), 1.89 – 1.77 (m, 1H, H^{11b}), 1.10 (s, 6H, H^8), 1.06 (s, 9H, H^1).

^{13}C NMR ($CDCl_3$, 100 MHz) δ_C 171.9 (C^4), 59.3 (C^9), 51.2 (C^3), 47.4 (C^6), 40.0 (C^7), 31.7 (C^{10}), 31.0 (C^2), 30.1 (C^1), 26.2 (C^{11}), 24.3 (C^8).
HRMS (ES^+) exact mass calculated for [M+H]+ ($C_{14}H_{28}ON^{32}S_2$) requires m/z 290.1607, found m/z 290.1607.

1.3.10. Compound 3j

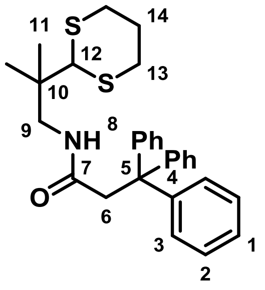

Off-white solid, 61%.
IR (film)/cm^{-1} $\mathbf{\nu_{max}}$ 3337 (N-H stretch), 2964 (C-H stretch), 1653 (C=O stretch), 1522 (C=C stretch).
^{1}H NMR ($CDCl_3$, 400 MHz) δ_H 7.33 – 7.20 (m, 15H, $H^{1,2,3,4}$), 5.27 (t, J = 5.9 Hz, 1H, H^8), 3.65 (s, 2H, H^6), 3.59 (s, 1H, H^{12}), 3.04 (d, J = 6.4 Hz, 2H, H^9), 2.86 – 2.68 (m, 4H, H^{13}), 2.08 – 2.00 (m, 1H, H^{14a}), 1.75 (dtt, J = 13.9, 12.0, 3.6 Hz, 1H, H^{14b}), 0.83 (s, 6H, H^{11}).
^{13}C NMR ($CDCl_3$, 100 MHz) δ_C 170.8 (C^7), 146.5 (C^4), 129.4 (C^3), 128.3 (C^2), 126.7 (C^1), 58.1 (C^{12}), 56.1 (C^5), 49.1 (C^6), 47.7 (C^9), 39.6 (C^{10}), 31.2 (C^{13}), 26.1 (C^{14}), 23.6 (C^{11}).
HRMS (ES^+) exact mass calculated for [M+H]+ ($C_{29}H_{34}ON^{32}S_2$) requires m/z 476.2087, found m/z 476.2075.

1.3.11. Compound 3k

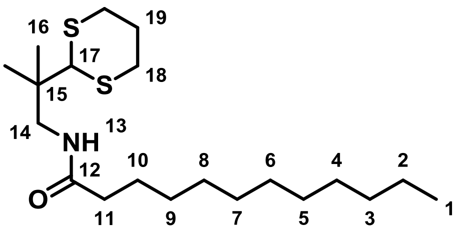

Off-white solid, 62%.
mp 64-66 ^{o}C.
IR (film)/cm^{-1} $\mathbf{\nu_{max}}$ 3279 (N-H stretch), 2924 (C-H stretch), 1649 (C=O stretch), 1559 C=C stretch).
^{1}H NMR ($CDCl_3$, 400 MHz) δ_H 5.89 (s, 1H, H^{13}), 4.03 (s, 1H, H^{17}), 3.34 (d, J = 6.6 Hz, 2H, H^{14}), 3.00 – 2.84 (m, 4H, H^{18}), 2.23 – 2.18 (m, 2H, H^{11}), 2.15 – 2.07 (m, 1H, H^{19a}), 1.91 – 1.77 (m, 1H, H^{19b}), 1.64 (p, J = 7.4 Hz, 2H, H^{10}), 1.36 – 1.22 (m, 16H, $H^{2, 3, 4, 5, 6, 7, 8, 9}$), 1.10 (s, 6H, H^{16}), 0.91 – 0.85 (m, 3H, H^1).
^{13}C NMR ($CDCl_3$, 100 MHz) δ_C 173.3 (C^{12}), 59.3 (C^{17}), 47.3 (C^{14}), 40.1 (C^{15}), 37.3 (C^{11}), 32.1 (C^3), 31.7 (C^{18}), 29.8 ($C^{4/5/6/7/8/9}$), 29.8 ($C^{4/5/6/7/8/9}$), 29.8 ($C^{4/5/6/7/8/9}$), 29.7 ($C^{4/5/6/7/8/9}$), 29.5 ($C^{4/5/6/7/8/9}$), 29.5 ($C^{4/5/6/7/8/9}$), 26.2 (C^{19}), 26.0 (C^{10}), 24.3 (C^{16}), 22.8 ($C^{4/5/6/7/8/9}$), 14.3 (C^1).
HRMS (ES^+) exact mass calculated for [M+H]+ ($C_{20}H_{40}ON^{32}S_2$) requires m/z 374.2546, found m/z 374.2540.

1.3.12. Compound 3l

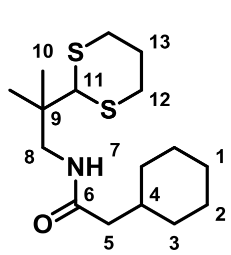

Off-white solid, 69%.
mp 98-99 ^{o}C.
IR (film)/cm^{-1} $\mathbf{\nu_{max}}$ 3304 (N-H stretch), 2922 (C-H stretch), 1642 (C=O stretch), 1546 (C=C stretch).

^{1}H NMR ($CDCl_3$, 400 MHz) δ_H 5.88 (s, 1H, H^7), 4.02 (s, 1H, H^{11}), 3.33 (d, *J* = 6.7 Hz, 2H, H^8), 2.94 – 2.83 (m, 4H, H^{12}), 2.15 – 2.04 (m, 3H, $H^{5, 13a}$), 1.89 – 1.60 (m, 8H, H, $H^{1a, 2a, 3a, 4, 13b}$), 1.28 (qt, *J* = 13.3, 3.4 Hz, 2H, H^{3b}), 1.20 – 1.10 (m, 1H, H^{1b}), 1.09 (s, 6H, H^{10}), 1.02 – 0.90 (m, 2H, H^{2b})
^{13}C NMR ($CDCl_3$, 100 MHz) δ_C 172.6 (C^6), 59.3 (C^{11}), 47.3 (C^8), 45.4 (C^5), 40.0 (C^9), 35.4 (C^4), 33.3 (C^2), 31.7 (C^{12}), 26.4 (C^1), 26.2 (C^3), 26.2 (C^{13}), 24.3 (C^{10}).
HRMS (ES^+) exact mass calculated for [M+Na]+ ($C_{16}H_{29}ON^{23}Na^{32}S_2$) requires m/z 338.1583, found m/z 338.1578.

1.3.13. Compound 3m

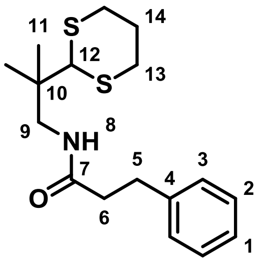

Off-white solid, 58%.
mp 96-97 °C.
IR (film)/cm^{-1} ν_{max} 3308 (N-H stretch), 2929 (C-H stretch), 1647 (C=O stretch), 1545 (C=C stretch).
^{1}H NMR ($CDCl_3$, 400 MHz) δ_H 7.33 – 7.18 (m, 5H, $H^{1,2,3}$), 5.79 (br s, 1H, H^8), 3.82 (s, 1H, H^{12}), 3.29 (d, *J* = 6.7 Hz, 2H, H^9), 2.99 (t, *J* = 7.6 Hz, 2H, H^6), 2.89 – 2.77 (m, 4H, H^{13}), 2.53 (t, *J* = 7.6 Hz, 2H, H^5), 2.12 – 2.04 (m, 1H, H^{14a}), 1.85 – 1.73 (m, 1H, H^{14b}), 1.02 (s, 6H, H^{11}).
^{13}C NMR ($CDCl_3$, 100 MHz) δ_C 172.2 (C^7), 141.0 (C^4), 128.7 (C^2), 128.5 (C^3), 126.4 (C^1), 58.8 (C^{12}), 47.4 (C^9), 40.0 (C^{10}), 38.8 (C^5), 31.8 (C^6), 31.5 (C^{13}), 26.1 (C^{14}), 24.1 (C^{11}).
HRMS (ES^+) exact mass calculated for [M+Na]+ ($C_{17}H_{25}ON^{23}Na^{32}S_2$) requires m/z 346.1270, found m/z 346.1267.

1.3.14. Compound 3n

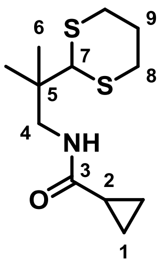

Clear oil, 45%.
IR (film)/cm^{-1} ν_{max} 3365 (N-H stretch), 2927 (C-H stretch), 1629 (C=O stretch), 1459 (C-S stretch).
^{1}H NMR (MeOH-d_4, 500 MHz) δ_H 4.24 (s, 1H, H^7), 3.01 (ddd, *J* = 14.8, 12.5, 2.5 Hz, 2H, H^{8a}), 2.89 (ddd, *J* = 14.6, 4.2, 3.2 Hz, 2H, H^{8b}), 2.11 (dtd, *J* = 13.6, 4.3, 2.1 Hz, 1H, H^{9a}), 1.78 (dtt, *J* = 13.9, 12.5, 3.2 Hz, 1H, H^{9b}), 1.70 (tt, *J* = 7.9, 4.6 Hz, 1H, H^2), 1.07 (s, 6H, H^6), 0.89 – 0.84 (m, 2H, H^{1a}), 0.80 – 0.75 (m, 2H, H^{1b}).
^{13}C NMR (MeOH-d_4, 500 MHz) δ_C 177.8 (C^3), 60.6 (C^7), 41.8 (C^5), 33.0 (C^8), 28.2 (C^9), 24.6 (C^6), 15.6 (C^2), 8.1 (C^1).
HRMS (ES^+) exact mass calculated for [M+Na]+ ($C_{12}H_{21}ON^{23}Na^{32}S_2$) requires m/z 282.0957, found m/z 282.0957.

1.3.15. Compound 3o

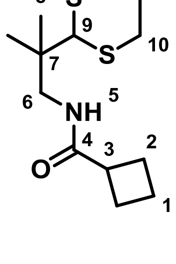

Pale brown solid, 52%.
mp 89-93 °C.
IR (film)/cm^{-1} ν_{max} 3303 (N-H stretch), 2971 (C-H stretch), 1646 (C=O stretch), 1543 (C-S stretch).

^{1}H NMR ($CDCl_3$, 400 MHz) δ_H 5.82 (s, 1H, H^{5}), 4.02 (s, 1H, H^{9}), 3.33 (d, *J* = 6.7 Hz, 2H, H^{6}), 3.03 (pd, *J* = 8.6, 1.0 Hz, 1H, H^{3}), 2.94 – 2.83 (m, 4H, H^{10}), 2.34 – 2.24 (m, 2H, H^{2a}), 2.22 – 2.07 (m, 3H, $H^{2b, 11a}$), 2.02 – 1.76 (m, 3H, $H^{1, 11b}$), 1.09 (s, 6H, H^{8}).
^{13}C NMR ($CDCl_3$, 400 MHz) δ_C 175.2 (C^{4}), 59.4 (C^{9}), 47.2 (C^{6}), 40.4 (C^{3}), 40.2 (C^{7}), 31.7 (C^{10}), 26.2 (C^{11}), 25.6 (C^{2}), 24.3 (C^{8}), 18.4 (C^{1}).
HRMS (ES^{+}) exact mass calculated for [M+Na]+ ($C_{13}H_{23}ON^{23}Na^{32}S_2$) requires m/z 296.1113, found m/z 296.1113.

1.3.16. Compound 3p

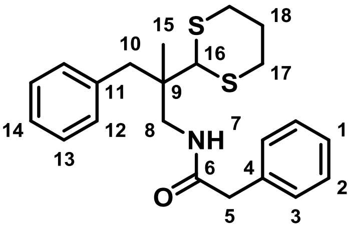

Off-white solid, 47%.
mp 146-148 °C.
IR (film)/cm^{-1} **ν_{max}** 3313 (N-H stretch), 2927 (C-H stretch), 1647 (C=O stretch), 1529 (C-S stretch), 1495 (C=C stretch).
^{1}H NMR ($CDCl_3$, 400 MHz) δ_H 7.39 – 7.34 (m, 2H, $H^{2/13}$), 7.32 – 7.21 (m, 8H, $H^{1, 2/13, 3, 12, 14}$), 5.92 (s, 1H, H^{7}), 3.74 (s, 1H, H^{16}), 3.58 (s, 2H, H^{5}), 3.40 – 3.27 (m, 2H, H^{8}), 2.85 (d, *J* = 13.5 Hz, 1H, H^{10a}), 2.78 (dtdd, *J* = 11.8, 4.8, 3.8, 2.0 Hz, 2H, H^{17a}), 2.71 – 2.60 (m, 3H, $H^{10b, 17b}$), 2.07 – 1.99 (m, 1H, H^{18a}), 1.72 (dtt, *J* = 13.9, 12.4, 3.3 Hz, 1H, H^{18b}), 0.95 – 0.92 (s, 3H, H^{15}).
^{13}C NMR ($CDCl_3$, 100 MHz) δ_C 171.0 (C^{6}), 137.2 (C^{4}), 135.3 (C^{11}), 130.8 (C^{3}), 129.9 (C^{12}), 129.1 (C^{2}), 128.2 (C^{13}), 127.4 (C^{1}), 126.7 (C^{14}), 57.6 (C^{16}), 45.9 (C^{8}), 44.3 (C^{5}), 43.3 (C^{9}), 42.0 (C^{10}), 31.6 (C^{17a}), 31.4 (C^{17b}), 26.0 (C^{18}), 21.6 (C^{15}).
HRMS (ES^{+}) exact mass calculated for [M+H]+ ($C_{22}H_{28}ON^{32}S_2$) requires m/z 386.1607, found m/z 386.1614.

1.3.17. Compound 3q

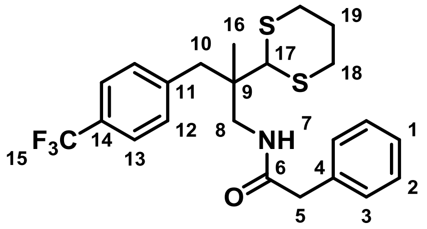

Oil, 51%.
IR (film)/cm^{-1} **ν_{max}** 3304 (N-H stretch), 2971 (C-H stretch), 1647 (C=O stretch), 1540 (C-S stretch).
^{1}H NMR ($CDCl_3$, 500 MHz) δ_H 7.52 (d, *J* = 8.0 Hz, 2H, H^{13}), 7.44 – 7.34 (m, 4H, $H^{3, 12}$), 7.33 – 7.25 (m, 3H, $H^{1, 2}$), 5.97 (t, *J* = 6.6 Hz, 1H, H^{7}), 3.66 (s, 1H, H^{17}), 3.60 (s, 2H, H^{5}), 3.40 (dd, *J* = 14.2, 6.6 Hz, 1H, H^{8a}), 3.25 (dd, *J* = 14.2, 6.6 Hz, 1H, H^{8b}), 2.92 (d, *J* = 13.4 Hz, 1H, H^{10a}), 2.79 (dt, *J* = 14.4, 3.6 Hz, 2H, H^{18a}), 2.74 – 2.57 (m, 3H, $H^{10b, 18b}$), 2.04 (dtt, *J* = 10.7, 4.2, 2.2 Hz, 1H, H^{19a}), 1.79 – 1.65 (m, 1H, H^{19b}), 0.91 (s, 3H, H^{16}).
^{13}C NMR ($CDCl_3$, 100 MHz) δ_C 171.2 (C^{6}), 141.4 (C^{11}), 135.2 (C^{4}), 131.1 (C^{3}), 129.9 (C^{2}), 129.2 (C^{12}), 129.0 (q, *J* = 32.3 Hz, C^{13}), 127.4 (C^{1}), 125.07 (q, *J* = 3.8 Hz, H^{13}), 123.3 (d, *J* = 272.8 Hz, H^{15}), 57.4 (C^{17}), 45.6 (C^{8}), 44.3 (C^{5}), 43.4 (C^{9}), 41.5 (C^{10}), 31.6 (C^{18a}), 31.4 (C^{18b}), 25.9 (C^{19}), 21.6 (C^{16}).
^{19}F NMR ($CDCl_3$, 400 MHz) δ_F -62.40.
HRMS (ES^{+}) exact mass calculated for [M+Na]+ ($C_{23}H_{26}F_3N^{32}S_2Na$) requires m/z 476.2087, found m/z 476.2075.

1.3.18. Compound 3r

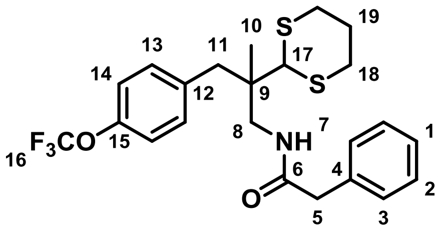

Oil, 49%.

IR (film)/cm^{-1} ν_{max} 3308 (N-H stretch), 2931 (C-H stretch), 1647 (C=O stretch), 1509 (C=C stretch).
^{1}H NMR ($CDCl_3$, 400 MHz) δ_H 7.41 – 7.24 (m, 7H, $H^{1,2,3,13}$), 7.11 (dt, J = 7.6, 1.1 Hz, 2H, H^{14}), 5.96 (t, J = 6.6 Hz, 1H, H^7), 3.68 (s, 1H, H^{17}), 3.60 (s, 1H, H^{11a}), 2.82 – 2.76 (m, 2H, H^{18a}), 2.71 – 2.57 (m, 3H, $H^{11b, 18b}$), 2.09 – 1.99 (m, 1H, H^{19a}), 1.72 (dtt, J = 13.9, 12.4, 3.2 Hz, 1H, H^{19b}), 0.91 (s, 3H, H^{10}).
^{13}C NMR ($CDCl_3$, 100 MHz) δ_C 171.1 (C^6), 148.2 (C^{15}), 135.9 (C^4), 135.2 (C^3), 132.1 (C^2), 129.9 (C^{13}), 129.2 (C^{16}), 128.8 (C^{12}), 127.4 (C^1), 120.6 (C^{14}), 57.4 (C^{17}), 45.7 (C^8), 44.3 (C^5), 43.3 (C^9), 41.0 (C^{11}), 31.6 (C^{18a}), 31.4 (C^{18b}), 26.0 (C^{19}), 21.6 (C^{10}).
^{19}F NMR ($CDCl_3$, 400 MHz) δ_F -57.79.
HRMS (ES^+) exact mass calculated for [M+H]+ ($C_{23}H_{27}ONF_3{}^{32}S_2$) requires m/z 454.1481, found m/z 454.1475.

1.3.19. Compound 3s

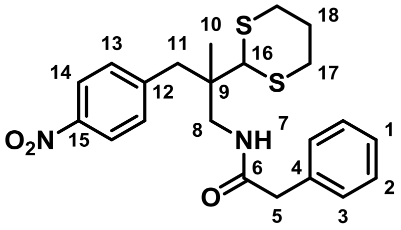

Cream solid, 55%.
mp 97-98 °C
IR (film)/cm^{-1} ν_{max} 3305 (N-H stretch), 2969 (C-H stretch), 1648 (C=O stretch), 1520 (C-S stretch), 1454 (N-O stretch).
^{1}H NMR ($CDCl_3$, 400 MHz) δ_H 8.13 (d, J = 8.7 Hz, 2H, H^{14}), 7.47 (d, J = 8.7 Hz, 2H, H^{13}), 7.44 – 7.28 (m, 5H, $H^{1, 2, 3}$), 5.97 (s, 1H, H^7), 3.64 (s, 1H, H^{16}), 3.62 (s, 2H, H^5), 3.44 (dd, J = 14.2, 6.9 Hz, 1H, H^{8a}), 3.22 (dd, J = 14.3, 6.4 Hz, 1H, H^{8b}), 2.99 (d, J = 13.3 Hz, 1H, H^{11a}), 2.80 (dt, J = 14.3, 3.7 Hz, 2H, H^{17a}), 2.73 – 2.57 (m, 3H, H^{17b}), 2.12 – 2.00 (m, 1H, H^{18a}), 1.81 – 1.66 (m, 1H, H^{18b}), 0.91 (s, 3H, H^{10}).

^{13}C NMR ($CDCl_3$, 100 MHz) δ_C 171.3 (C^6), 147.0 (C^{15}), 145.2 (C^{12}), 135.1 (C^4), 131.7 (C^{13}), 129.9 (C^2), 129.3 (C^3), , 127.5 (C^1), 123.3 (C^{14}), 57.5 (C^{16}), 45.5 (C^8), 44.3 (C^5), 43.6 (C^9), 41.3 (C^{11}), 31.6 (C^{17a}), 31.4 (C^{17b}), 25.9 (C^{18}), 21.6 (C^{10}).

HRMS (ES^+) exact mass calculated for [M+Na]+ ($C_{22}H_{26}O_3N_2{}^{23}Na^{32}S_2$) requires m/z 453.1277, found m/z 453.1268.

1.3.20. Compound 3t

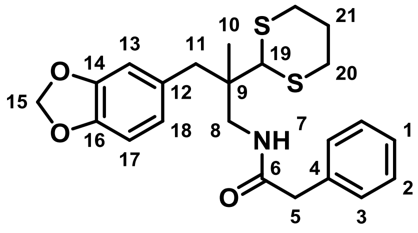

Oil, 50%.
IR (film)/cm^{-1} ν_{max} 3309 (N-H stretch), 2929 (C-H stretch), 1648 (C=O stretch), 1528 (C-S stretch).
^{1}H NMR ($CDCl_3$, 400 MHz) 7.43 – 7.34 (m, 2H, H^2), 7.32 – 7.27 (m, 3H, $H^{1, 3}$), 6.75 (d, J = 1.6 Hz, 1H, H^{13}), 6.71 (d, J = 7.9 Hz, 1H, H^{18}), 6.67 (dd, J = 8.0, 1.6 Hz, 1H, H^{17}), 5.96 – 5.90 (m, 3H, $H^{7, 15}$), 3.72 (s, 1H, H^{19}), 3.59 (s, 2H, H^5), 3.31 (d, J = 6.5 Hz, 2H, H^8), 2.85 – 2.71 (m, 3H, $H^{11a, 20a}$), 2.65 (dddd, J = 13.9, 12.4, 2.6, 1.1 Hz, 2H, H^{20b}), 2.53 (d, J = 13.7 Hz, 1H, H^{11b}), 2.07 – 1.98 (m, 1H, H^{21a}), 1.71 (dtt, J = 13.7, 12.4, 3.3 Hz, 1H, H^{21b}), 0.92 (s, 3H, H^{10}).
^{13}C NMR ($CDCl_3$, 100 MHz) δ_C 171.0 (C^6), 147.5 (C^{14}), 146.4 (C^{16}), 135.3 (C^4), 130.7 (C^{12}), 129.9 (C^1), 129.1 (C^2), 127.4 (C^3), 123.8 (C^{18}), 111.1 (C^{13}), 108.1 (C^{17}), 101.0 (C^{15}), 57.6 (C^{19}), 45.9 (C^8), 44.4 (C^5), 43.4 (C^9), 41.7 (C^{11}), 31.6 (C^{20a}), 31.4 (C^{20b}), 26.0 (C^{21}), 21.6 (C^{10}).
HRMS (ES^+) exact mass calculated for [M+Na]+ ($C_{23}H_{27}O_3N^{23}Na^{32}S_2$) requires m/z 452.1325, found m/z 452.1324.

1.3.21. Compound 3u

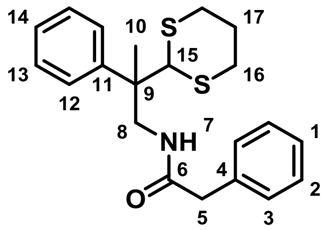

Oil, 36%.

IR (film)/cm^{-1} $\mathbf{v_{max}}$ 3308 (N-H stretch), 2980 (C-H stretch), 1649 (C=O stretch), 1529 (C-S stretch).

^{1}H NMR ($CDCl_3$, 500 MHz) δ_H 7.31 – 7.19 (m, 8H, $H^{1, 2, 3, 13, 14}$), 7.08 – 7.00 (m, 2H, H^{12}), 5.29 (s, 1H, H^{7}), 4.26 (s, 1H, H^{15}), 3.87 (dd, *J* = 13.8, 7.1 Hz, 1H, H^{8a}), 3.55 (dd, *J* = 13.8, 5.1 Hz, 1H, H^{8b}), 3.49 (d, *J* = 16.0 Hz, 1H, H^{5a}), 3.45 (d, *J* = 16.1 Hz, 1H, H^{5b}), 2.86 – 2.77 (m, 2H, H^{16a}), 2.74 – 2.65 (m, 2H, H^{16b}), 2.08 – 1.95 (m, 1H, H^{17a}), 1.74 (ddtd, *J* = 15.6, 9.5, 7.9, 6.3 Hz, 1H, H^{17b}), 1.43 (s, 3H, H^{10}).

^{13}C NMR ($CDCl_3$, 126 MHz) δ_C 170.8 (C^{6}), 142.0 (C^{11}), 134.7 (C^{4}), 129.4 (C^{3}), 129.0 (C^{2}), 128.5 (C^{13}), 127.2 (C^{1}), 127.2 (C^{14}), 126.5 (C^{12}), 59.2 (C^{15}), 47.8 (C^{8}), 46.8 (C^{9}), 44.0 (C5), 31.7 (C^{16a}), 31.4 (C^{16b}), 25.9 (C^{17}), 20.3 (C^{10}).

HRMS (ES^+) exact mass calculated for [M+H]+ ($C_{21}H_{26}ON^{32}S_2$) requires m/z 372.1450, found m/z 372.1448.

1.3.22. Compound 3v

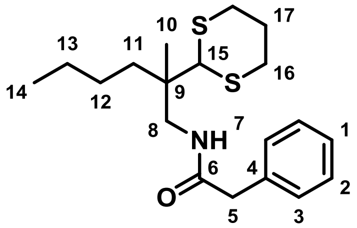

Oil, 56%.

IR (film)/cm^{-1} $\mathbf{v_{max}}$ 3309 (N-H stretch), 2931 (C-H stretch), 1648 (C=O stretch), 1544 (C-S stretch).

^{1}H NMR ($CDCl_3$, 400 MHz) 7.41 – 7.35 (m, 2H, H^{2}), 7.32 – 7.27 (m, 3H, $H^{1, 3}$), 5.88 (s, 1H, H^{15}), 3.74 (s, 1H, H^{15}), 3.60 (s, 2H, H^{5}), 3.36 – 3.22 (m, 2H, H^{8}), 2.82 – 2.72 (m, 2H, H^{16a}), 2.71 – 2.59 (m, 2H, H^{16b}), 2.02 (dtt, *J* = 13.5, 4.1, 2.5 Hz, 1H, H^{17a}), 1.70 (dtt, *J* = 14.0, 12.3, 3.4 Hz, 1H, H^{17b}), 1.53 – 1.37 (m, 1H, H^{11a}), 1.34 – 1.15 (m, 5H, $H^{11b, 12, 13}$), 0.96 (s, 3H, H^{10}), 0.93 – 0.84 (m, 3H, H^{14}).

^{13}C NMR ($CDCl_3$, 100 MHz) δ_C 171.0 (C^{6}), 135.5 (C^{4}), 129.9 (C^{2}), 129.2 (C^{3}), 127.4 (C^{1}), 58.6 (C^{15}), 45.6 (C^{8}), 44.4 (C^{5}), 42.3 (C^{9}), 36.2 (C^{11}), 31.6 (C^{16a}), 31.6 (C^{16b}), 26.2 (C^{17}), 25.7 (C^{12}), 23.5 (C^{13}), 21.5 (C^{10}), 14.2 (C^{14}).

HRMS (ES^+) exact mass calculated for [M+H]+ ($C_{19}H_{30}ON^{32}S_2$) requires m/z 352.1763, found m/z 352.1757.

1.3.23. Compound 3w

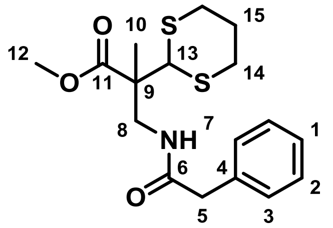

Oil, 47%.

IR (film)/cm^{-1} $\mathbf{v_{max}}$ 3308 (N-H stretch), 2940 (C-H stretch), 1732 (C=O ester stretch), 1654 (C=O amide stretch), 1546 (C-S stretch).

^{1}H NMR ($CDCl_3$, 400 MHz) δ_H 7.40 – 7.27 (m, 5H, $H^{1,2,3}$), 5.87 (s, 1H, H^{7}), 4.29 (s, 1H, H^{13}), 3.70 (dd, *J* = 14.1, 7.3 Hz, 1H, H^{8a}), 3.63 (s, 3H, H^{12}), 3.57 (d, *J* = 1.7 Hz, 2H, H^{5}), 3.42 (dd, *J* = 14.0, 5.7 Hz, 1H, H^{8b}), 2.90 – 2.68 (m, 4H, H^{14}), 2.11 – 2.01 (m, 1H, H^{15a}), 1.85 – 1.71 (m, 1H, H^{15b}), 1.24 (s, 3H, H^{10}).

^{13}C NMR ($CDCl_3$, 100 MHz) δ_C 174.0 (C^{11}), 171.0 (C^{6}), 135.2 (C^{4}), 129.7 (C^{2}), 129.2 (C^{3}), 127.5 (C^{1}), 54.8 (C^{13}), 52.6 (C^{12}), 51.8 (C^{9}), 44.2 (C^{5}), 44.1 (C^{8}), 31.6 (C^{14a}), 31.4 (C^{14b}), 26.0 (C^{15}), 18.2 (C^{10}).

HRMS (ES^+) exact mass calculated for [M+Na]+ ($C_{17}H_{23}O_3N^{23}Na^{32}S_2$) requires m/z 376.1012, found m/z 376.1006.

1.3.24. Compound 3x

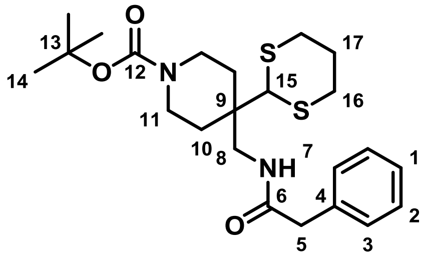

Clear crystalline solid, 53%.
mp 24-25 °C.
IR (film)/cm^{-1} ν_{max} 3291 (N-H stretch), 2980 (C-H stretch), 1685 (C=O carbamate stretch), 1652 (C=O amide stretch), 1525 (C-S stretch).
^{1}H NMR ($CDCl_3$, 500 MHz) 7.39 – 7.35 (m, 2H, H^2), 7.32 – 7.27 (m, 3H, $H^{1,3}$), 6.08 (t, *J* = 6.7 Hz, 1H, H^7), 3.90 (s, 1H, H^{15}), 3.60 (s, 2H, H^5), 3.59 – 3.23 (m, 6H, $H^{8, 11}$), 2.77 (dt, *J* = 14.1, 3.7 Hz, 2H, H^{16a}), 2.67 (t, *J* = 13.2 Hz, 2H, H^{16b}), 2.04 (dtt, *J* = 13.5, 4.1, 2.5 Hz, 1H, H^{17a}), 1.79 – 1.61 (m, 3H, $H^{10a, 17b}$), 1.44 (s, 11H, $H^{10b, 14}$).
^{13}C NMR ($CDCl_3$, 125 MHz) δ_C 171.2 (C^6), 155.0 (C^{12}), 135.2 (C^4), 129.9 (C^3), 129.1 (C^2), 127.4 (C^1), 79.6 (C^{13}), 57.4 (C^{15}), 44.2 (C^5), 42.4 (C^8), 41.0 (C^9), 40.0 (C^{11a}), 39.1 (C^{11b}), 31.6 (C^{16}), 31.3 (C^{10}), 28.6 (C^{14}), 26.2 (C^{17}).
HRMS (ES^+) exact mass calculated for [M+Na]+ ($C_{23}H_{34}O_3N_2{}^{23}Na^{32}S_2$) requires m/z 473.1903, found m/z 473.1898.

1.4. Derivatisation

1.4.1. Compound 6

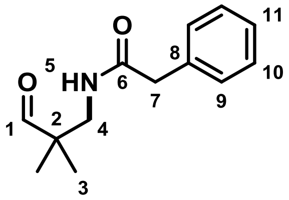

To a solution of **3a** (30.9 mg, 0.1 mmol) in MeCN (3 mL) was added TFA (77 µL, 1 mmol) followed by $PhI(OAc)_2$ (97 mg, 0.3 mmol). The reaction was stirred for 10 minutes or until full disappearance of the starting material by TLC. The reaction mixture was diluted with water, extracted with diethyl ether and the combined organic layers dried over magnesium sulfate and concentrated *in vacuo*. The crude product was purified by flash column chromatography to give **6** as an oil (16.8 mg, 0.077 mmol, 77%).

IR (film)/cm^{-1} ν_{max} 3301 (N-H stretch), 2980 (C-H stretch), 2929 (C-H stretch), 1723 (C=O aldehyde stretch), 1651 (C=O amide stretch), 1544 (C-S stretch).
^{1}H NMR ($CDCl_3$, 400 MHz) δ_H 9.36 (s, 1H, H^1), 7.36 (ddt, *J* = 8.1, 6.6, 1.1 Hz, 2H, H^{10}), 7.32 – 7.27 (m, 1H, H^{11}), 7.25 – 7.20 (m, 2H, H^9), 5.73 (s, 1H, H^5), 3.55 (s, 2H, H^7), 3.32 (d, *J* = 6.5 Hz, 2H, H^4), 1.01 (s, 6H, H^3).
^{13}C NMR ($CDCl_3$, 100 MHz) δ_C 205.3 (C^1), 171.2 (C^6), 134.9 (C^8), 129.5 (C^{10}), 129.2 (C^9), 127.6 (C^{11}), 47.6 (C^2), 44.4 (C^4), 44.0 (C^7), 19.7 (C^3).
HRMS (ES^+) exact mass calculated for [M+H]+ ($C_{13}H_{18}O_2N$) requires m/z 220.1332, found m/z 220.1335.

1.4.2. Compound 7

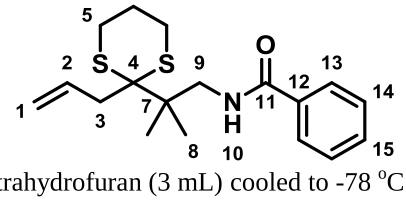

A solution of **3e** (20 mg, 0.068 mmol) in tetrahydrofuran (3 mL) cooled to -78 °C was added n-BuLi (0.13 mL, 1.6M in hexane, 0.20 mmol). The reaction was stirred for 15 minutes, before addition of allyl bromide (29 µL, 0.34 mmol). The reaction was warmed slowly to room temperature and stirred for a further 3 hours. The reaction was diluted with saturated ammonium chloride and extracted with ethyl acetate. The combined organic layers were washed with brine, dried over sodium sulfate and concentrated *in vacuo*. The crude product was purified by flash column chromatography to give **7** as an oil (14 mg, 61%).

IR (film)/cm^{-1} ν_{max} 3324 (N-H stretch), 2973 (C-H stretch), 2933 (C-H stretch), 1645 (C=O stretch), 1537 (C=C stretch).
^{1}H NMR ($CDCl_3$, 500 MHz) δ_H 7.86 (d, *J* = 7.3 Hz, 2H, H^{13}), 7.53 – 7.36 (m, 3H, $H^{14, 15}$), 7.30 (s, 1H, H^{10}), 6.13 (ddt, *J* = 17.0, 10.2, 6.8 Hz, 1H, H^2), 5.20 (dt, *J* = 17.1, 1.9 Hz, 1H, H^{1a}), 5.12 (dd, *J* = 10.2, 1.8 Hz, 1H, H^{1b}), 3.73 (d, *J* = 6.1 Hz, 2H, H^9), 3.11 – 2.99 (m, 4H, $H^{3, 5a}$), 2.76 (dt, *J* = 14.5, 4.2 Hz, 2H, H^{5b}), 2.10 (dp, *J* = 12.0, 3.9 Hz, 1H, H^{6a}), 1.92 – 1.78 (m, 1H, H^{6b}), 1.22 (s, 6H, H^8).
^{13}C NMR ($CDCl_3$, 100 MHz) δ_C 167.4 (C^{11}), 136.5 (C^2), 135.1 (C^{12}), 131.4 (C^{15}), 128.7 (C^{14}), 127.1 (C^{13}), 116.8 (C^1), 60.9 (C^4), 48.6 (C^9), 44.7 (C^7), 41.3 (C^3), 26.7 (C^5), 24.9 (C^6), 22.8 (C^8).
HRMS (ES^+) exact mass calculated for [M+H]+ ($C_{18}H_{26}ON^{32}S_2$) requires m/z 336.1450, found m/z 336.1450.

1.4.3. Compound 8

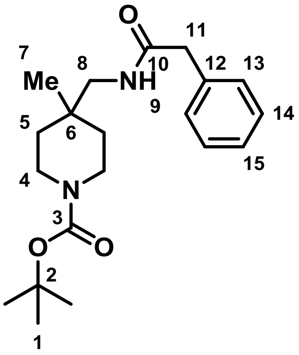

To a solution of **3x** (30 mg, 0.067 mmol) in ethanol (3 mL) was added Raney nickel (100 mg) and the reaction slurry stirred for 5 hours. The reaction was filtered through a pad of celite, taking care not to let the Raney nickel dry out. The filtrate was concentrated and the crude product was purified by flash column chromatography to give **8** as a clear oil (20.3 mg, 88%).

IR (film)/cm^{-1} ν_{max} 3311 (N-H stretch), 2980 (C-H stretch), 2926 (C-H stretch), 1691 (C=O carbamate stretch), 1652 (C=O amide stretch), 1552 (C=C stretch).
^{1}H NMR ($CDCl_3$, 500 MHz) δ_H 7.31 (t, *J* = 7.4 Hz, 2H, H^{12}), 7.31 (t, *J* = 7.4 Hz, 1H, H^{15}), 7.28 – 7.24 (m, 2H, H^{14}), 5.35 (s, 1H, H^9), 3.69 – 3.50 (m, 4H, $H^{11, 4a}$), 3.13 (ddd, *J* = 13.5, 9.3, 3.7 Hz, 2H, H^{4b}), 1.44 (s, 9H, H^1), 1.30 – 1.22 (m, 2H, H^{5a}), 1.16 (dt, *J* = 13.8, 4.0 Hz, 2H, H^{5b}), 0.80 (s, 3H, H^7).
^{13}C NMR ($CDCl_3$, 126 MHz) δ_C 171.3 (C^{10}), 155.0 (C^3), 135.0 (C^{12}), 129.6 (C^{13}), 129.3 (C^{14}), 127.7 (C^{15}), 79.5 (C^2), 48.7 (C^4), 44.1 (C^8), 34.4 (C^5), 33.4 (C^6), 28.6 (C^1), 22.0 (C^7).
HRMS (ES^+) exact mass calculated for [M+H]+ ($C_{20}H_{31}O_3N_2$) requires m/z 347.2329, found m/z 347.2326.

3. NMR Spectra

3.1. Compound 2b

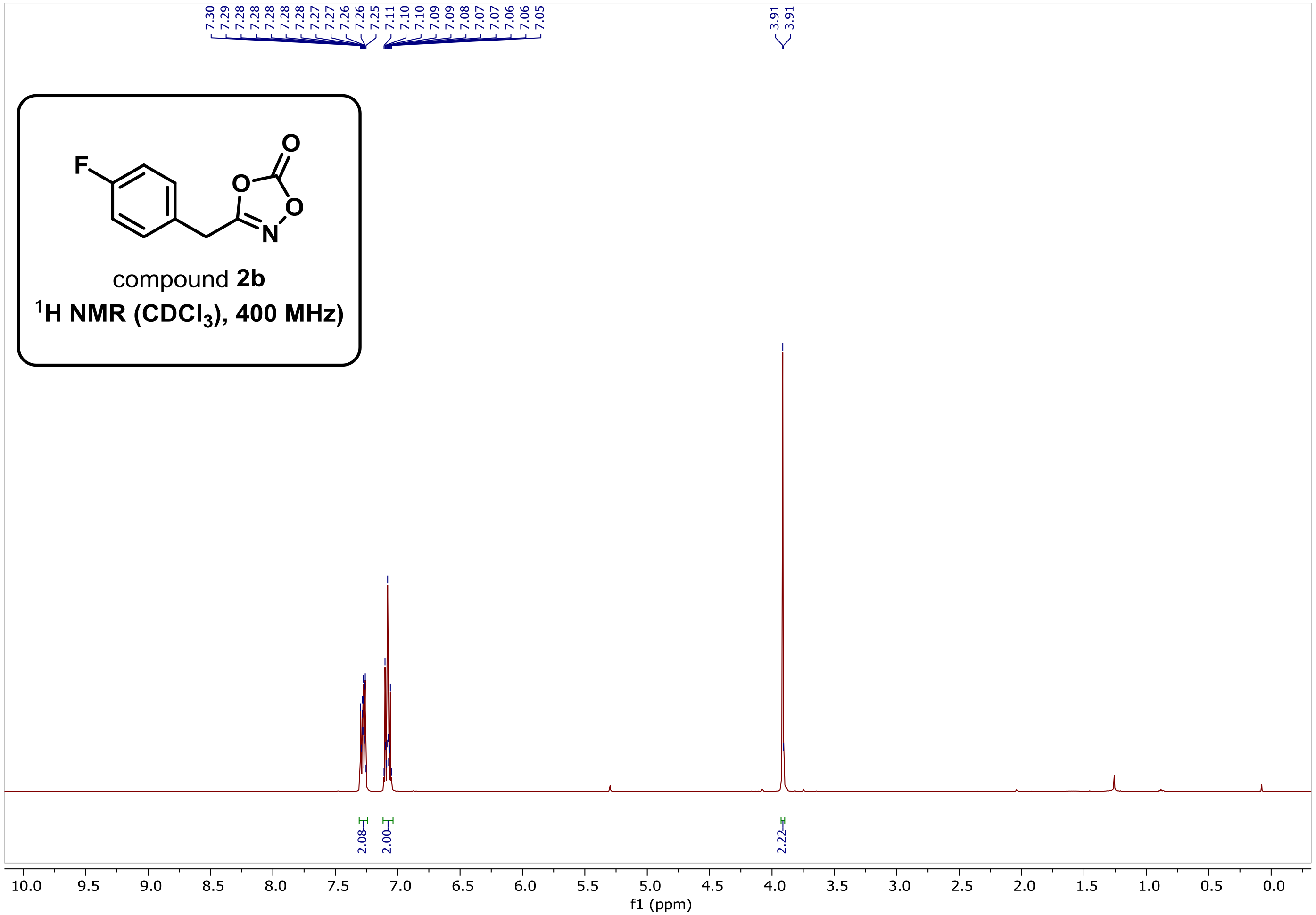

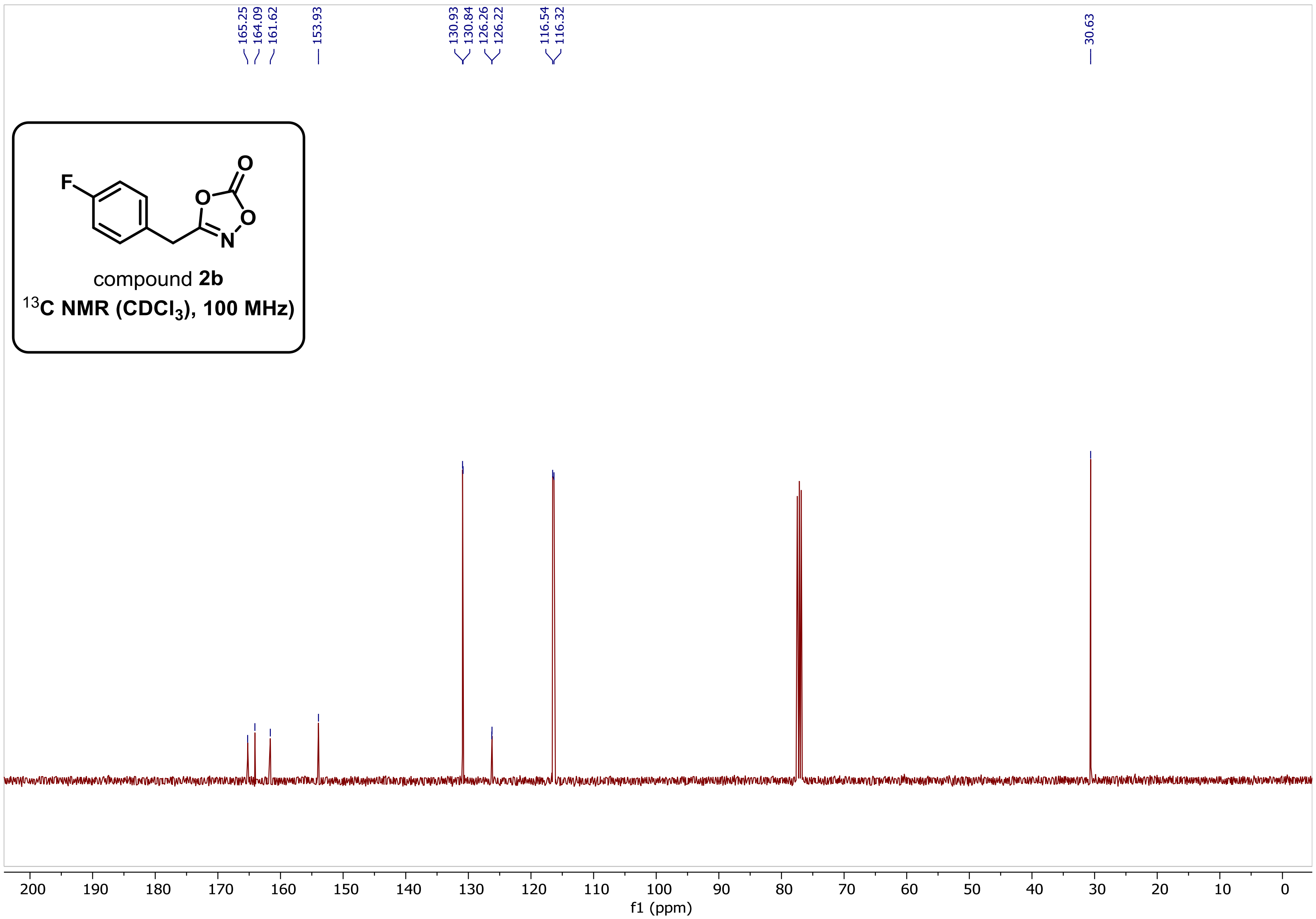
165.25
164.09
161.62
153.93
130.93
130.84
126.26
126.22
116.54
116.32
30.63
compound 2b
13C NMR (CDCl3), 100 MHz)
f1 (ppm)

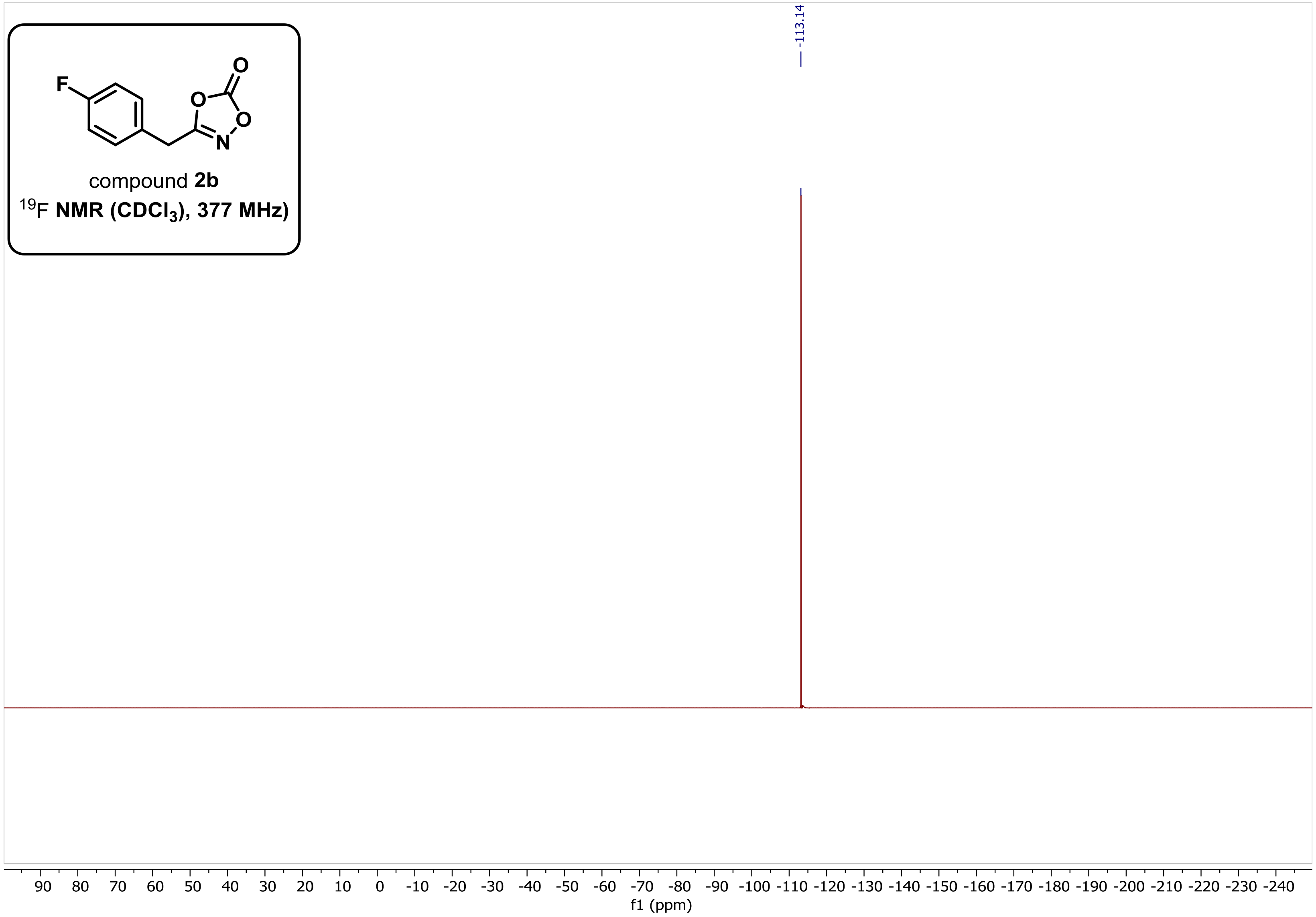

compound **2b**
^{19}F NMR ($CDCl_3$), 377 MHz)
-113.14
90 80 70 60 50 40 30 20 10 0 -10 -20 -30 -40 -50 -60 -70 -80 -90 -100 -110 -120 -130 -140 -150 -160 -170 -180 -190 -200 -210 -220 -230 -240
f1 (ppm)

3.2. Compound 2c

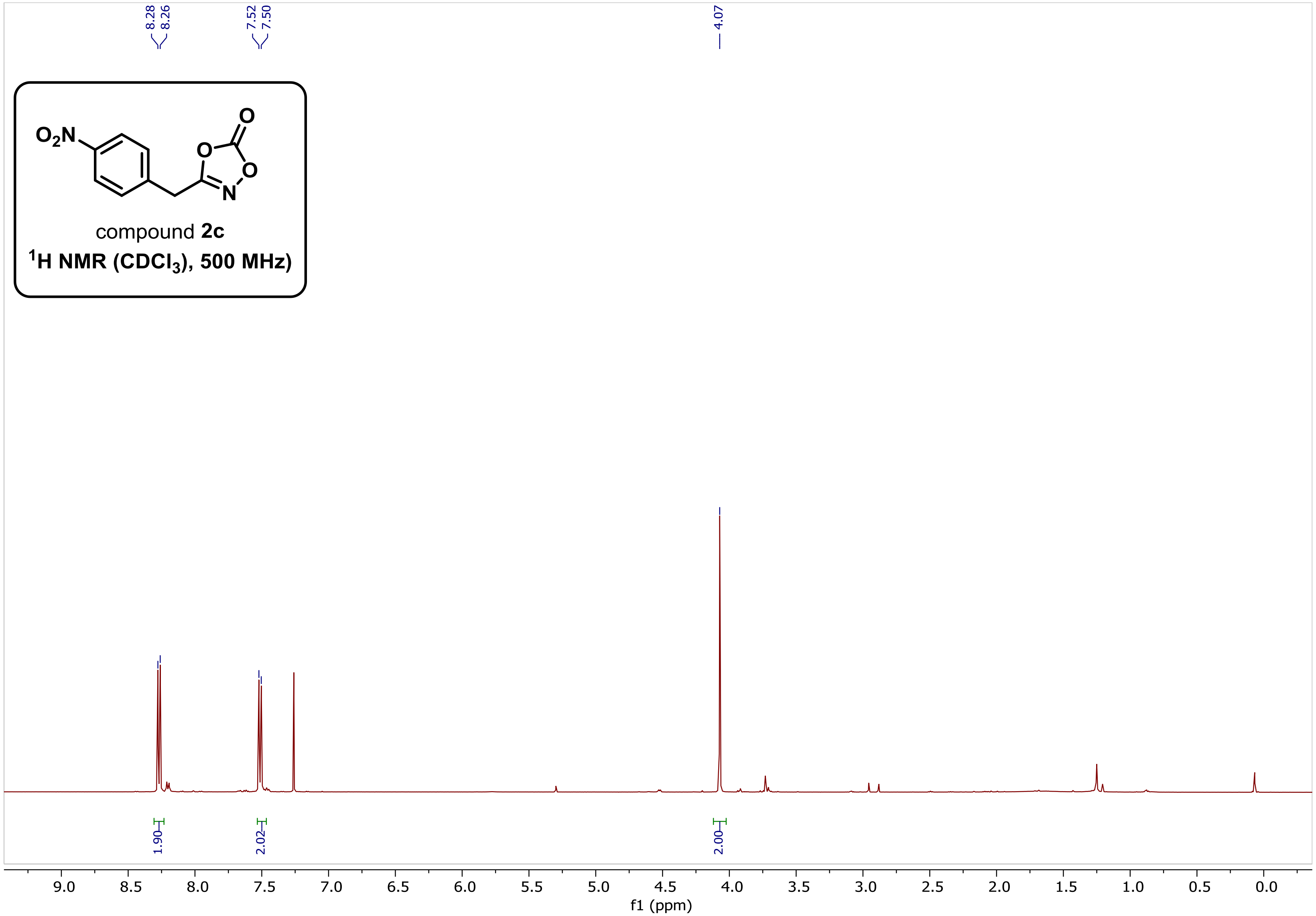

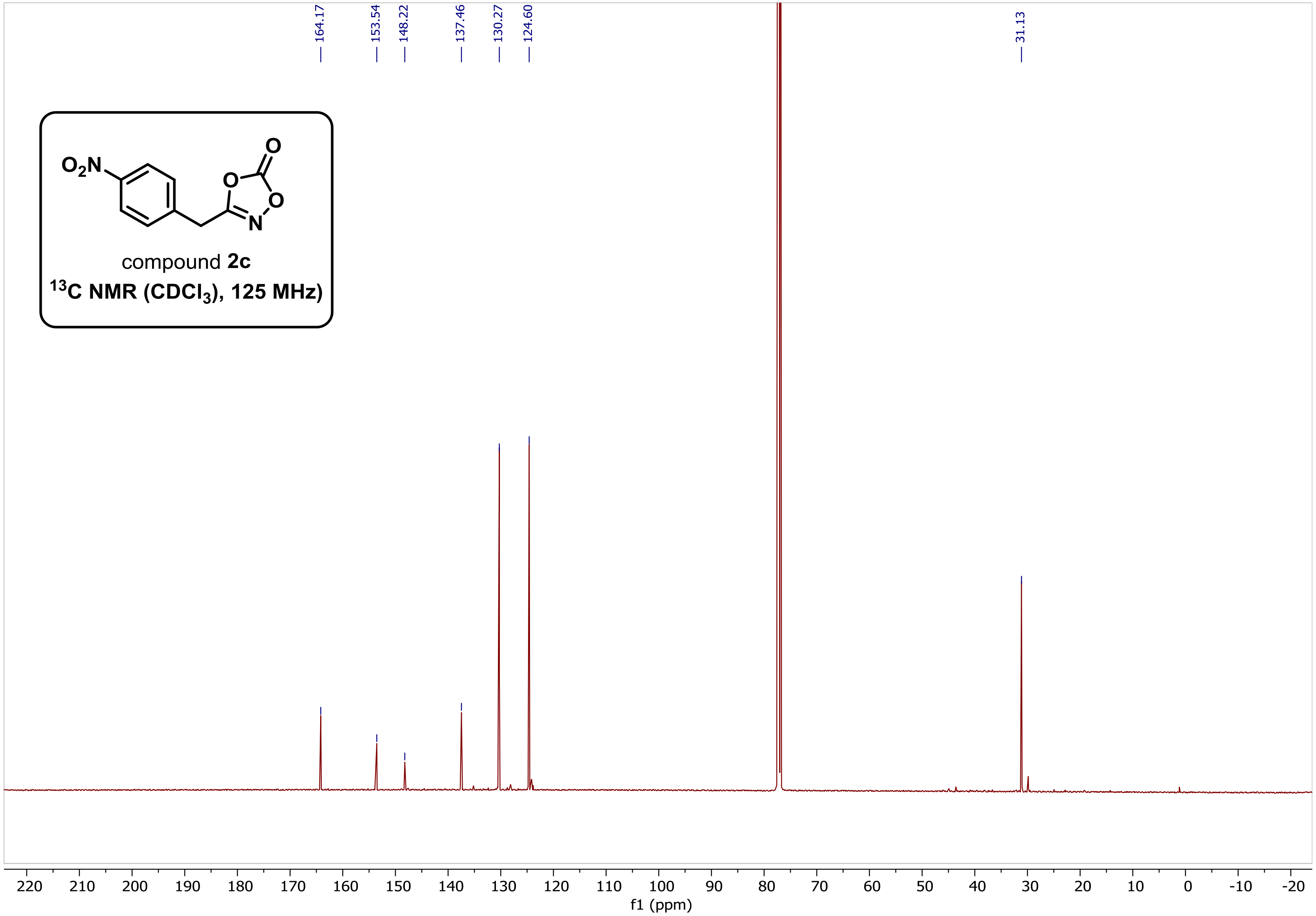

164.17
153.54
148.22
137.46
130.27
124.60
31.13
O_2N
O
N
compound **2c**
^{13}C NMR ($CDCl_3$), 125 MHz)
220 210 200 190 180 170 160 150 140 130 120 110 100 90 80 70 60 50 40 30 20 10 0 -10 -20
f1 (ppm)

3.3. Compound 3g

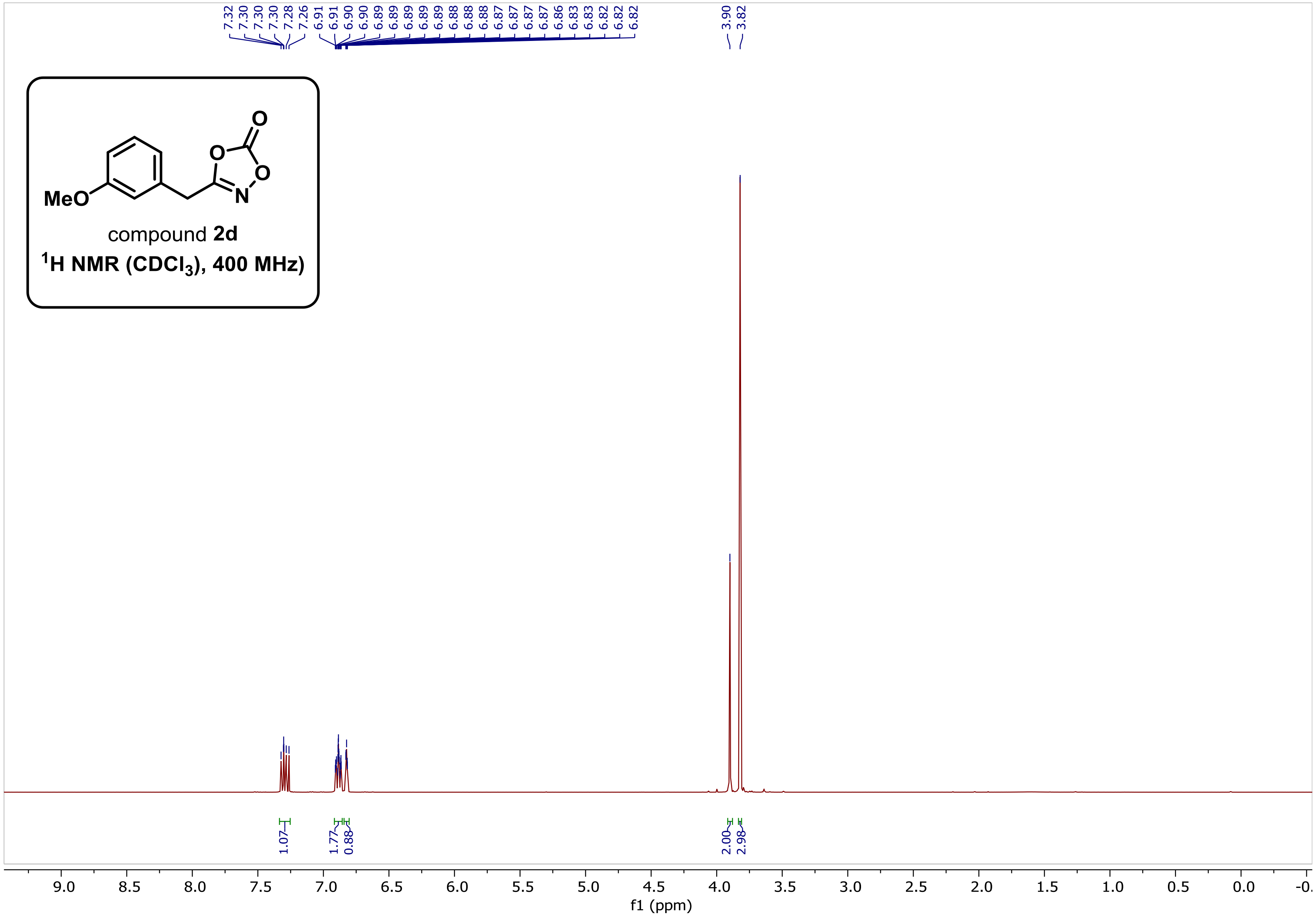

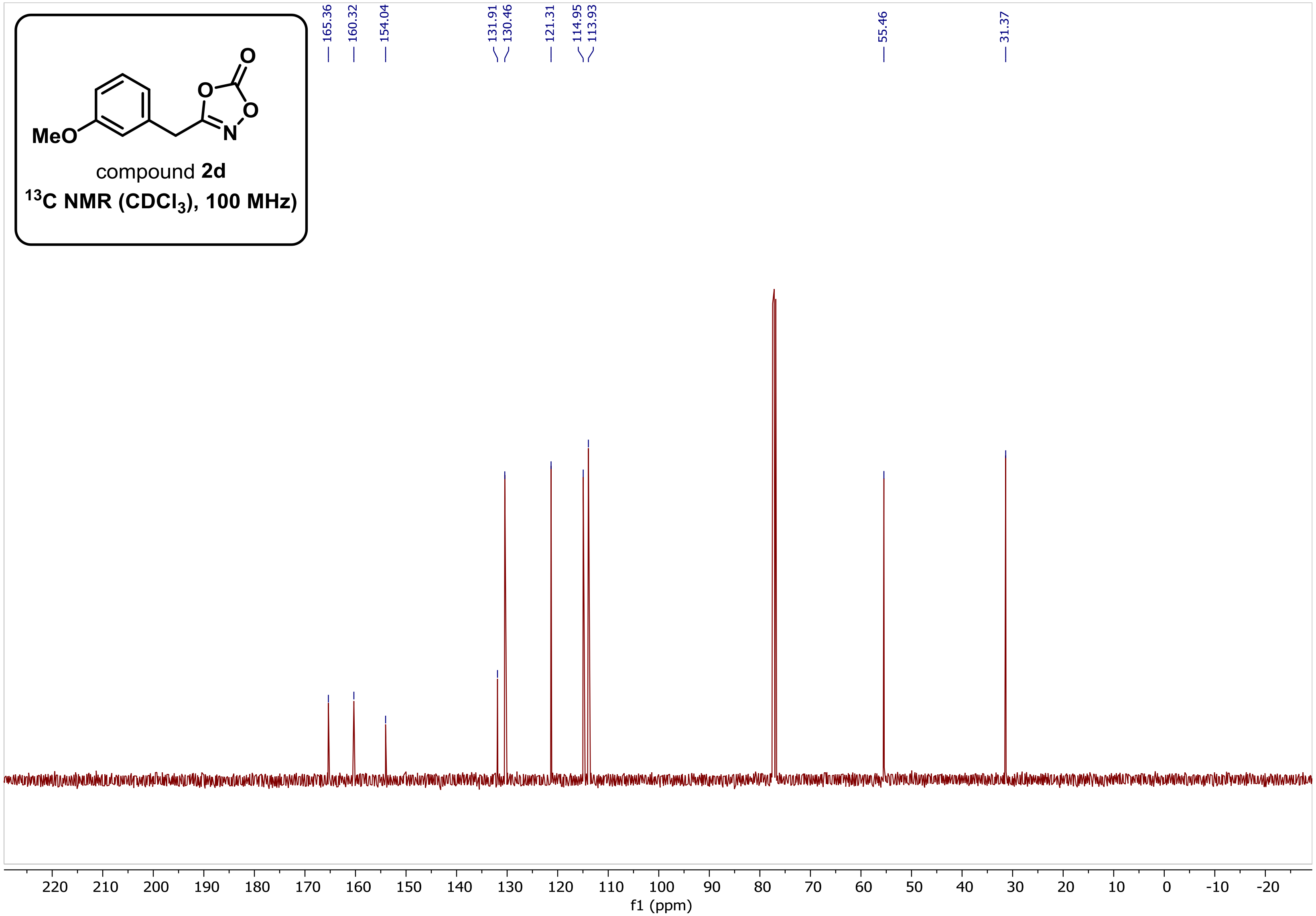
165.36
160.32
154.04
131.91
130.46
121.31
114.95
113.93
55.46
31.37
MeO
compound **2d**
^{13}C NMR ($CDCl_3$), 100 MHz)
220 210 200 190 180 170 160 150 140 130 120 110 100 90 80 70 60 50 40 30 20 10 0 -10 -20
f1 (ppm)

3.4. Compound 2g

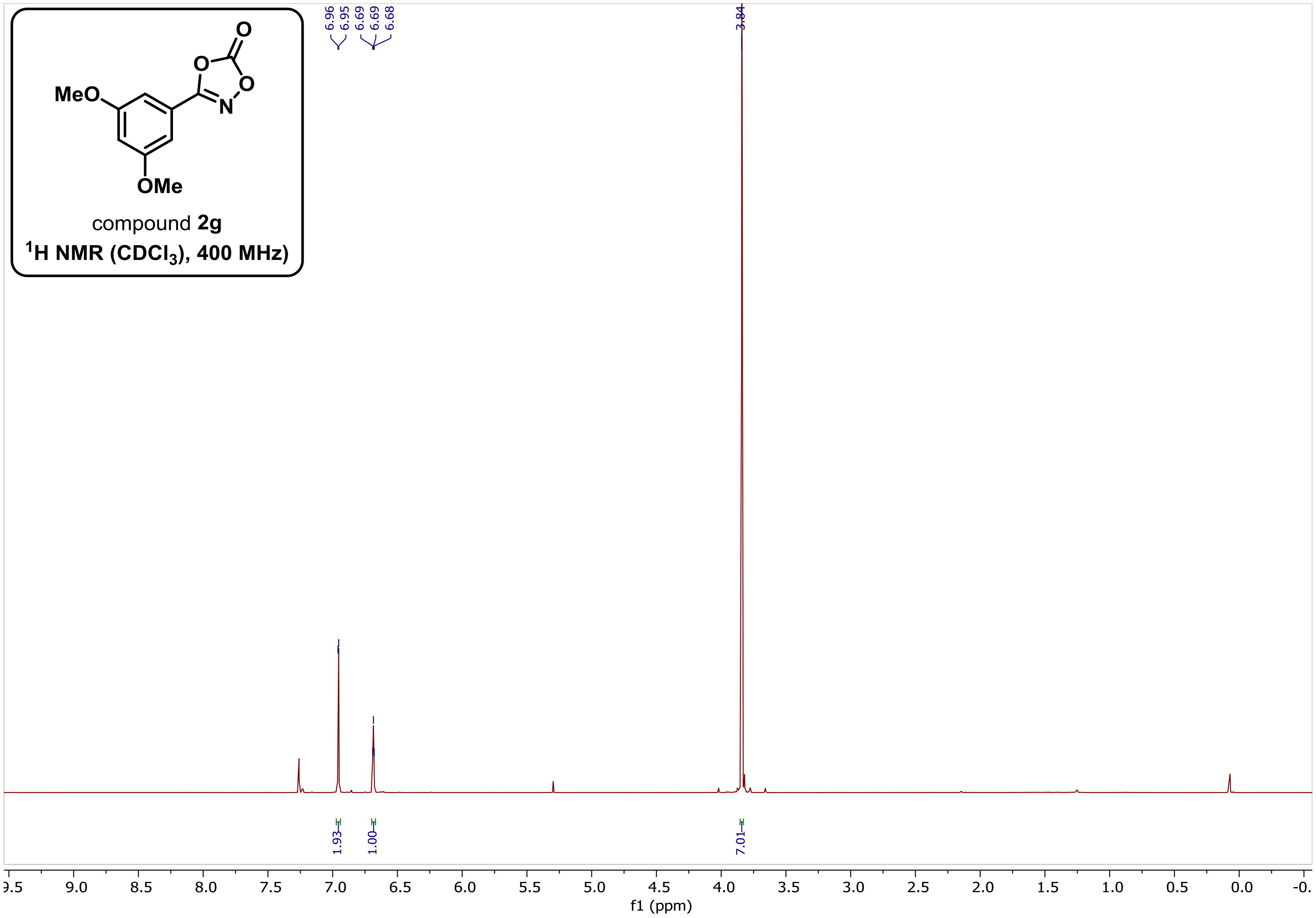

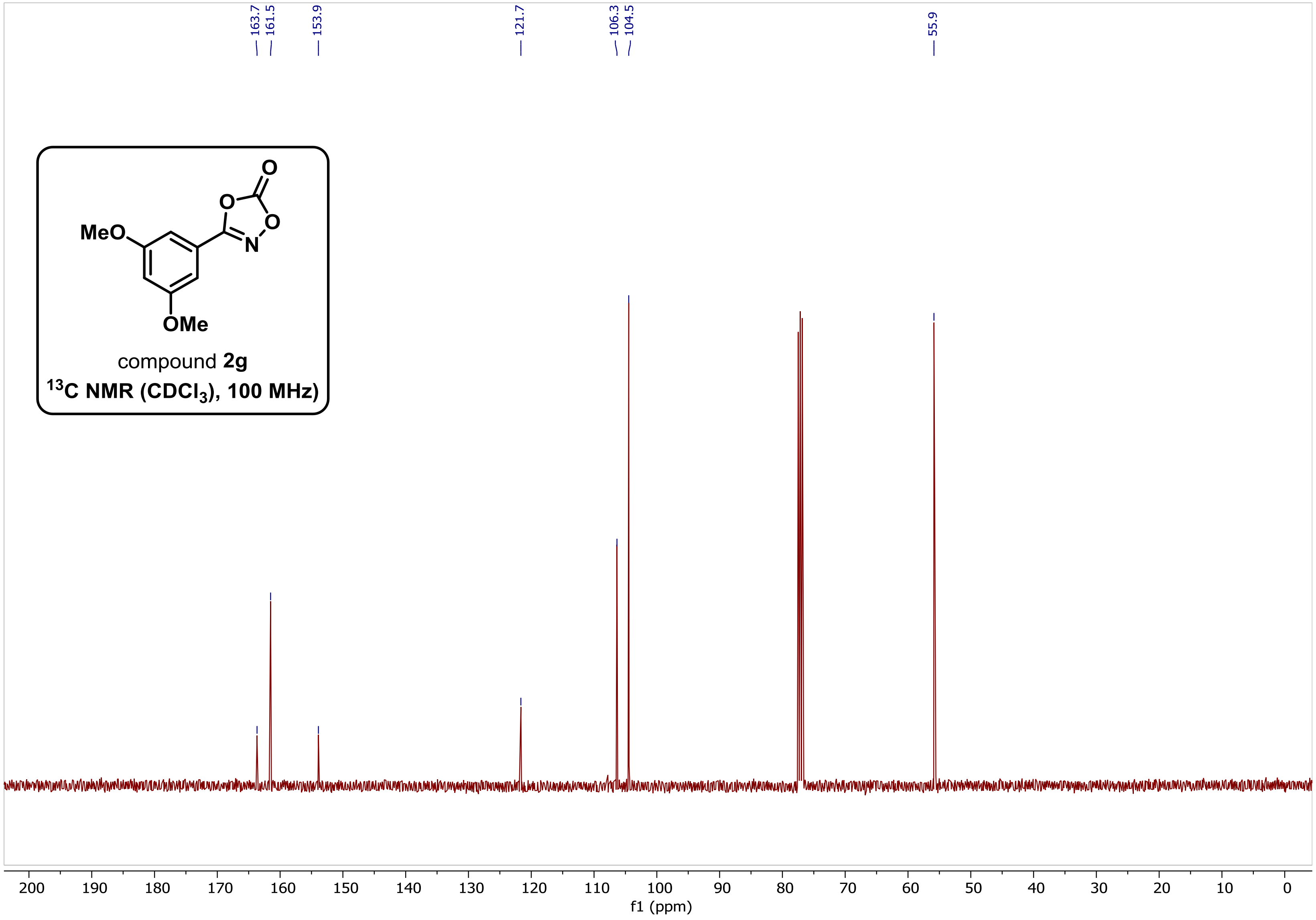

163.7
161.5
153.9
121.7
106.3
104.5
55.9
MeO
O
O
O
N
OMe
compound **2g**
^{13}C NMR ($CDCl_3$), 100 MHz)
200
190
180
170
160
150
140
130
120
110
100
90
80
70
60
50
40
30
20
10
0
f1 (ppm)

3.5. Compound 2i

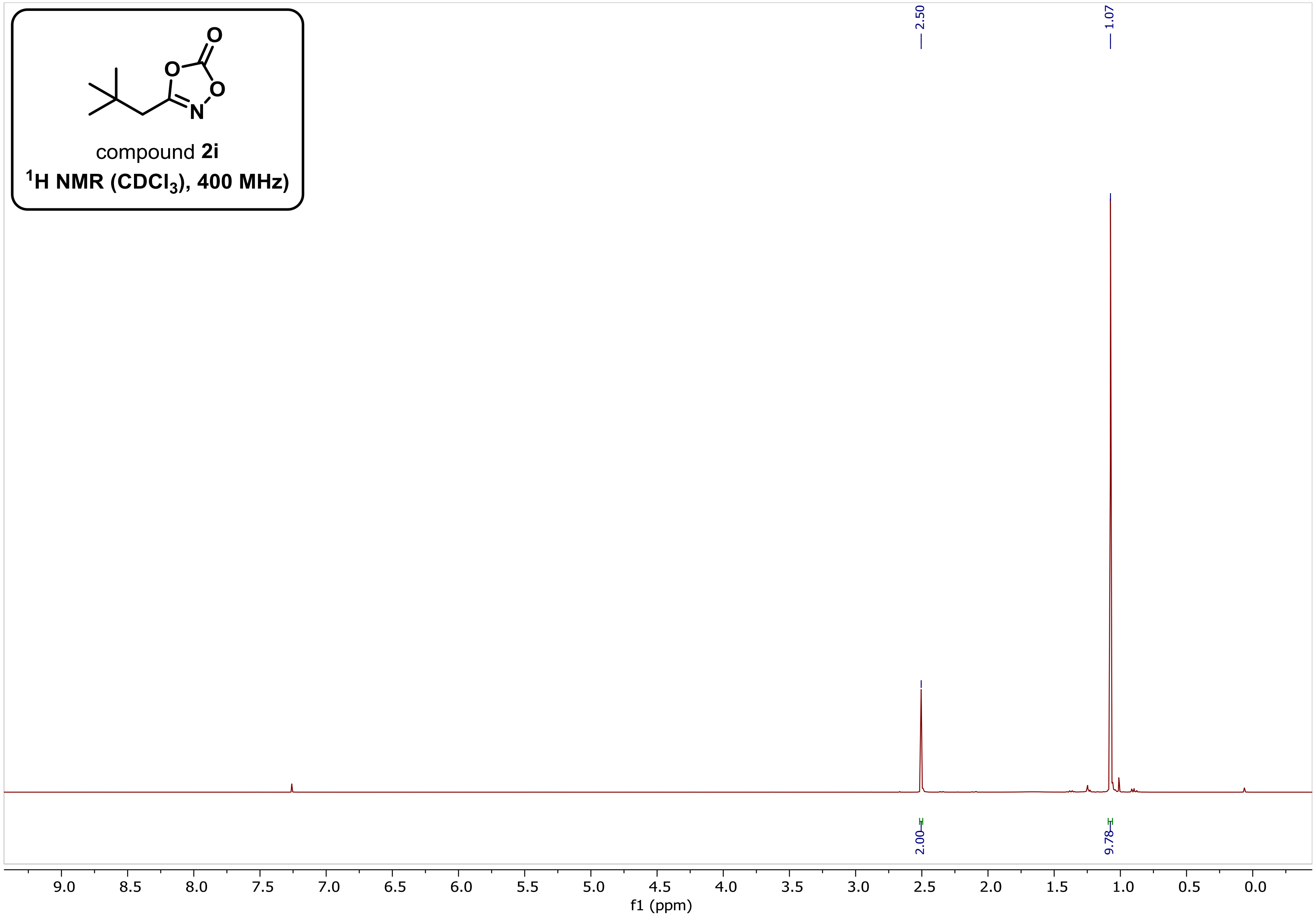

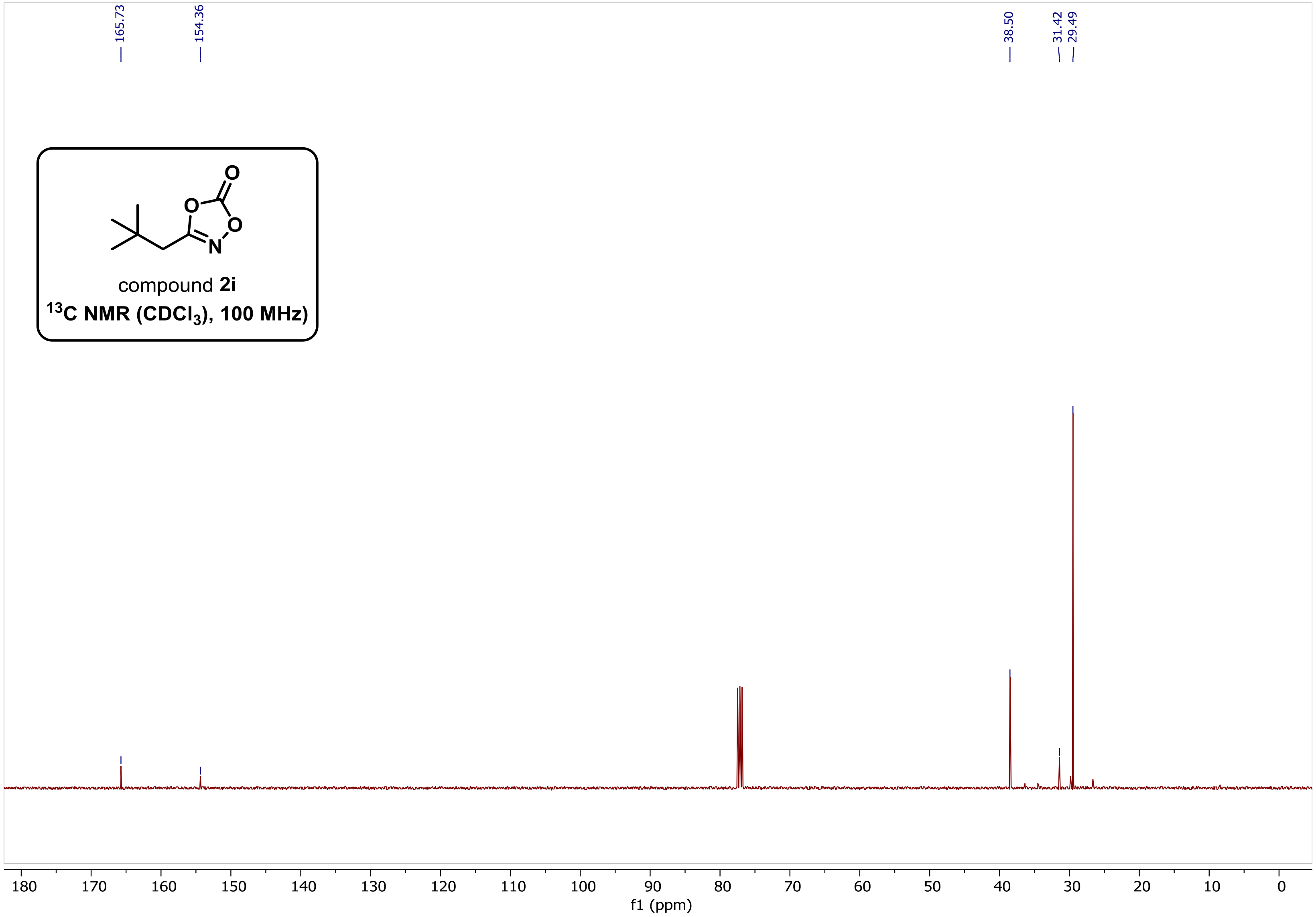

165.73
154.36
38.50
31.42
29.49
compound 2i
13C NMR (CDCl3), 100 MHz)
180 170 160 150 140 130 120 110 100 90 80 70 60 50 40 30 20 10 0
f1 (ppm)

3.6. Compound 2j

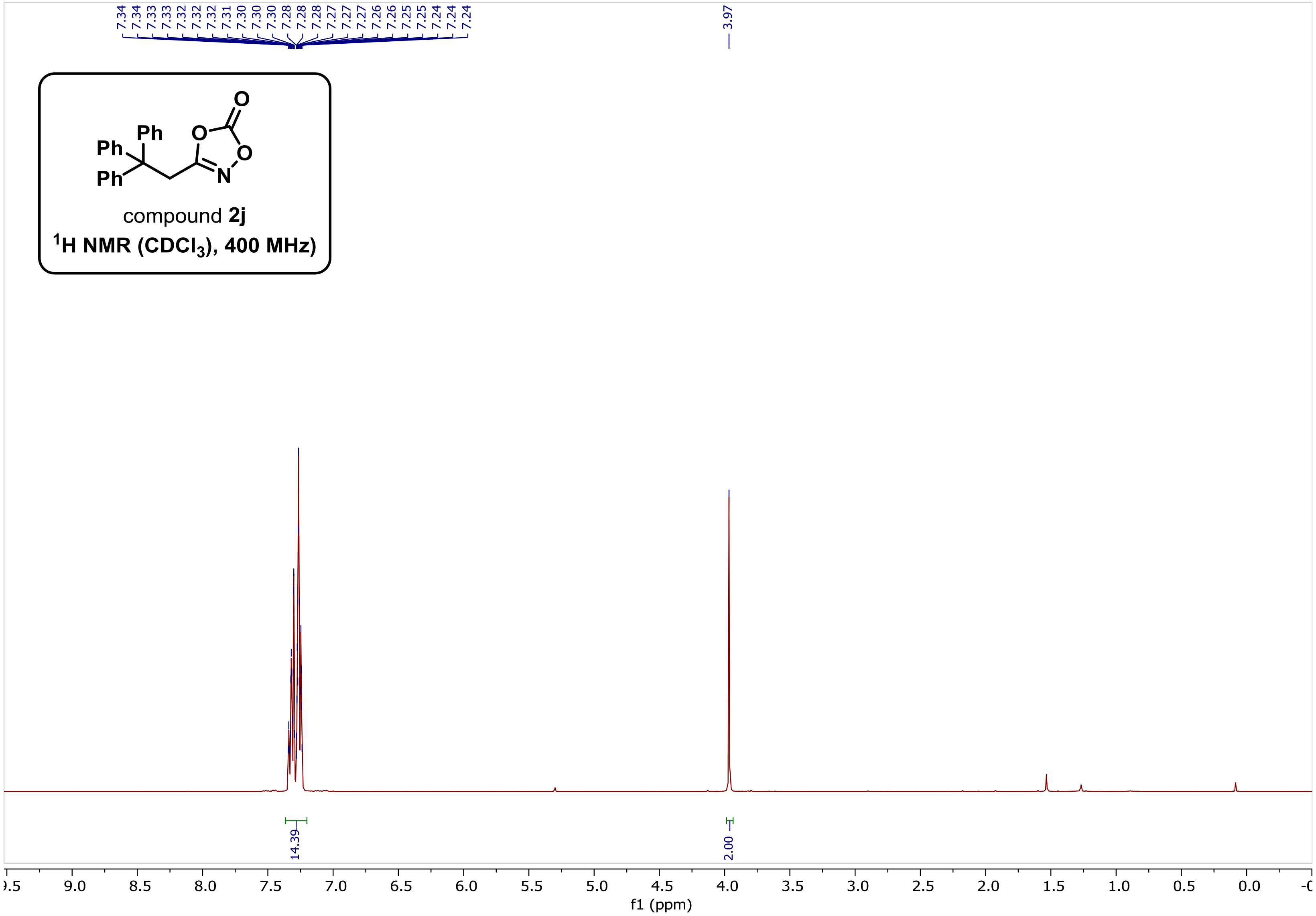

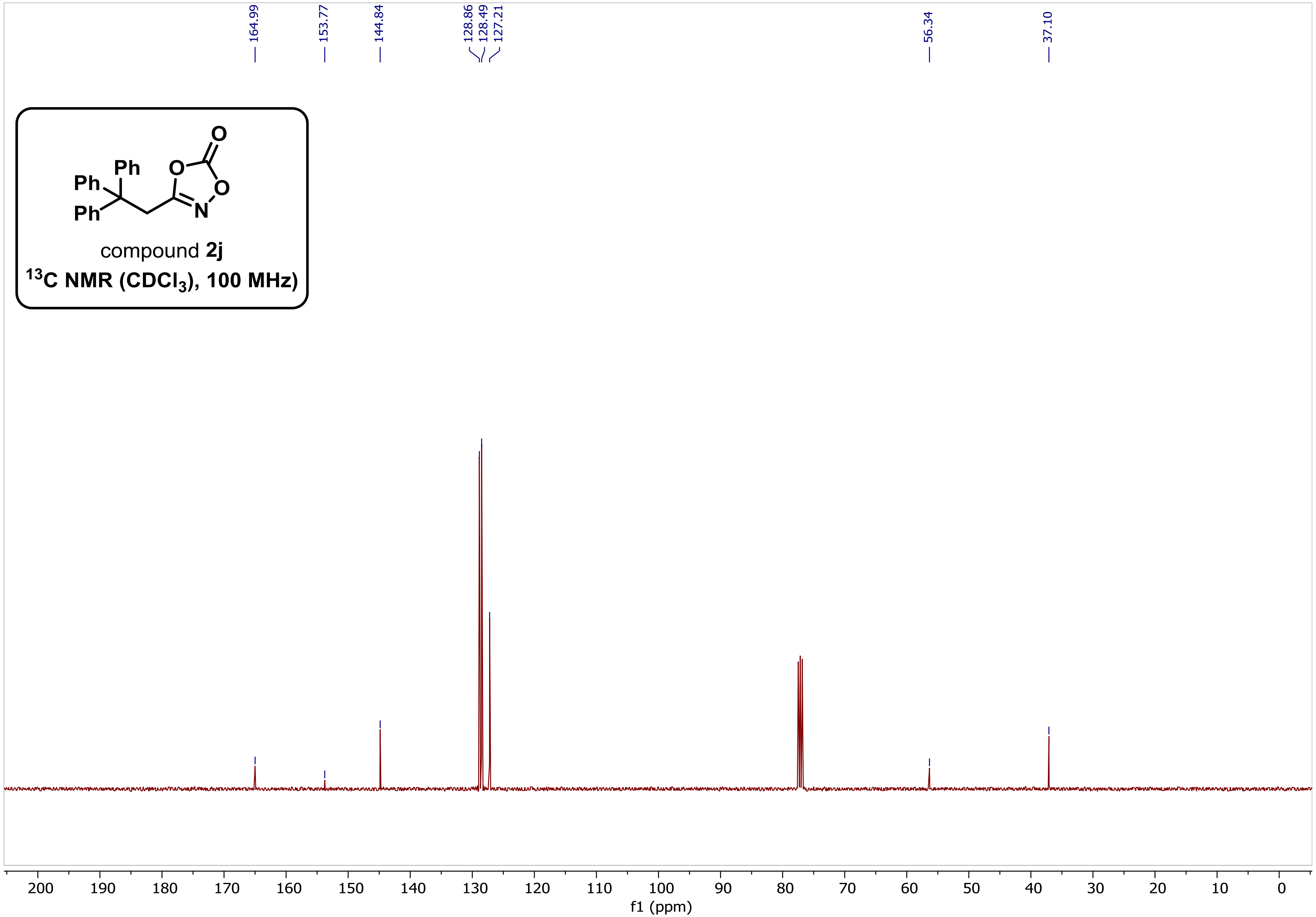
164.99
153.77
144.84
128.86
128.49
127.21
56.34
37.10
Ph
Ph
Ph
O
O
O
N
compound 2j
13C NMR (CDCl3), 100 MHz)
200
190
180
170
160
150
140
130
120
110
100
90
80
70
60
50
40
30
20
10
0
f1 (ppm)

3.7. Compound 2k

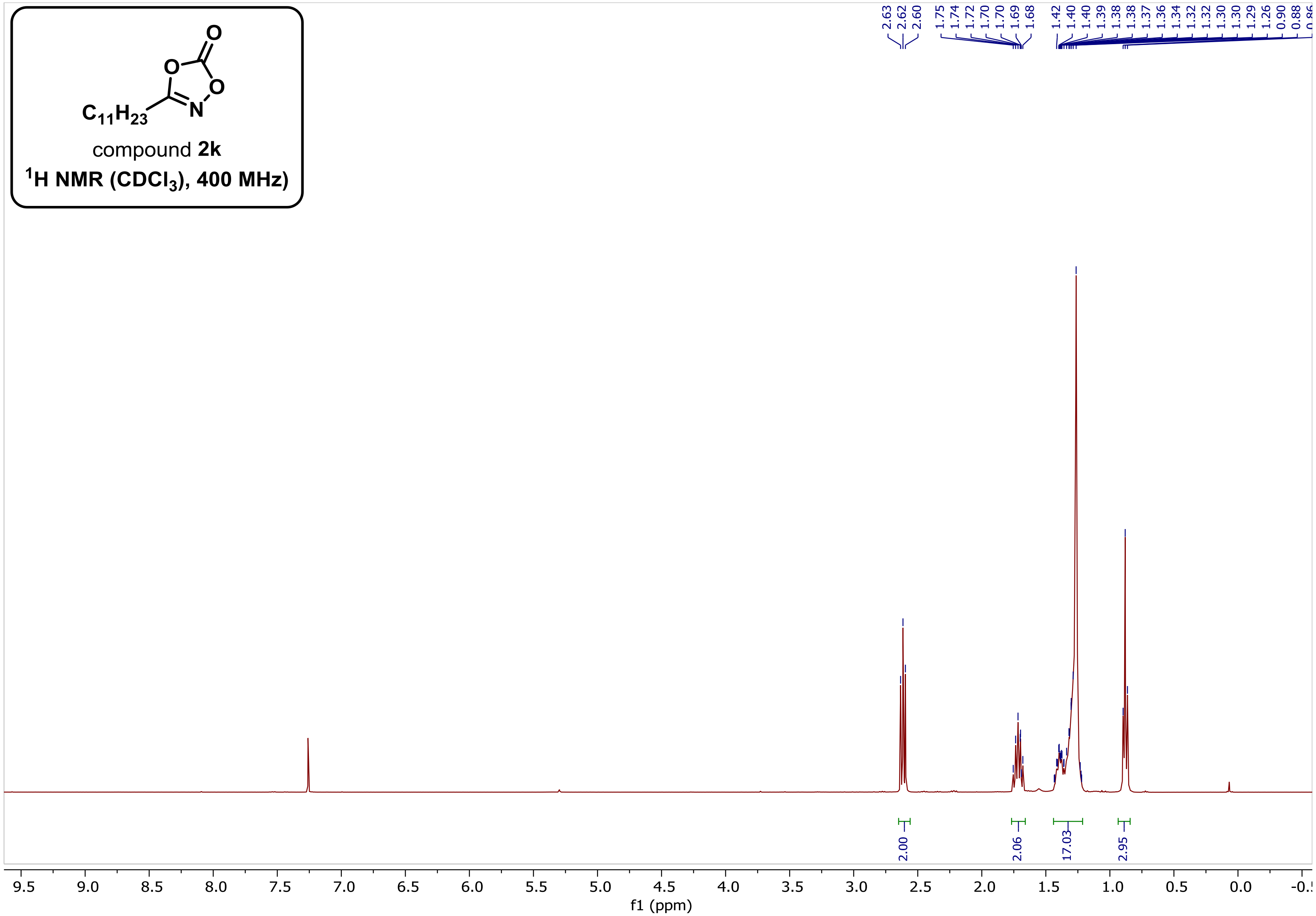

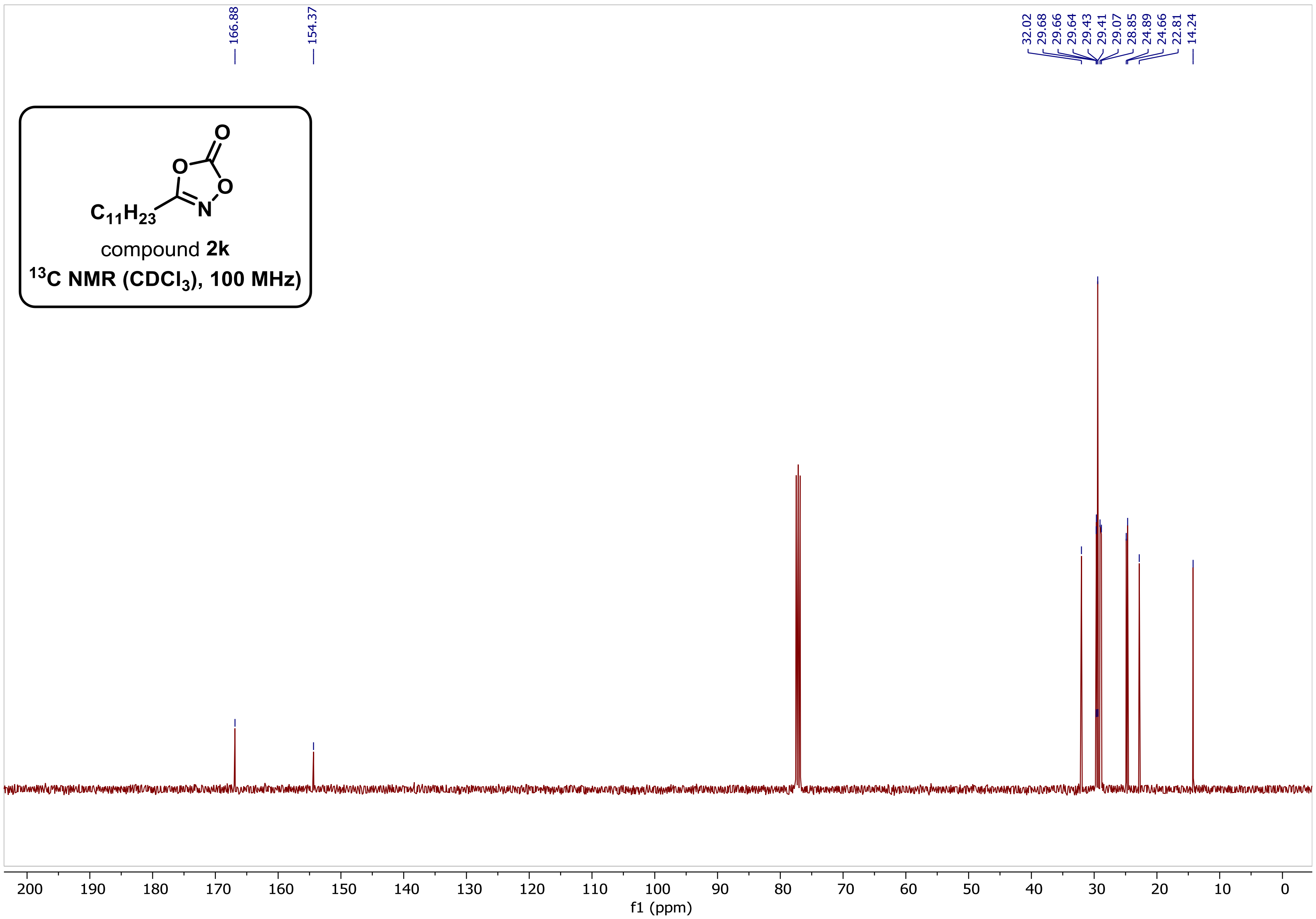

compound **2k**
^{13}C NMR ($CDCl_3$), 100 MHz)
166.88
154.37
32.02
29.68
29.66
29.64
29.43
29.41
29.07
28.85
24.89
24.66
22.81
14.24
f1 (ppm)

3.8. Compound 2l

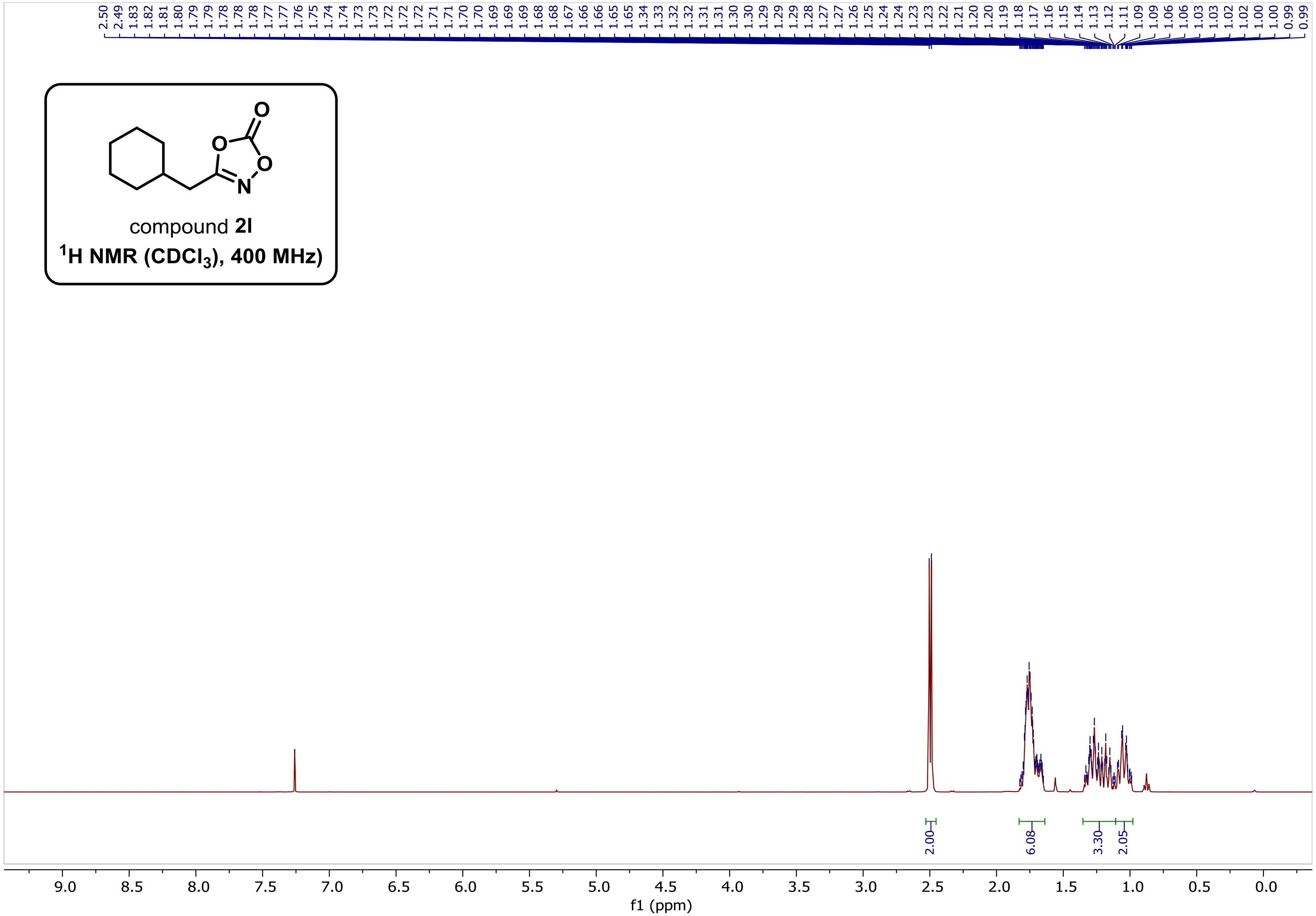

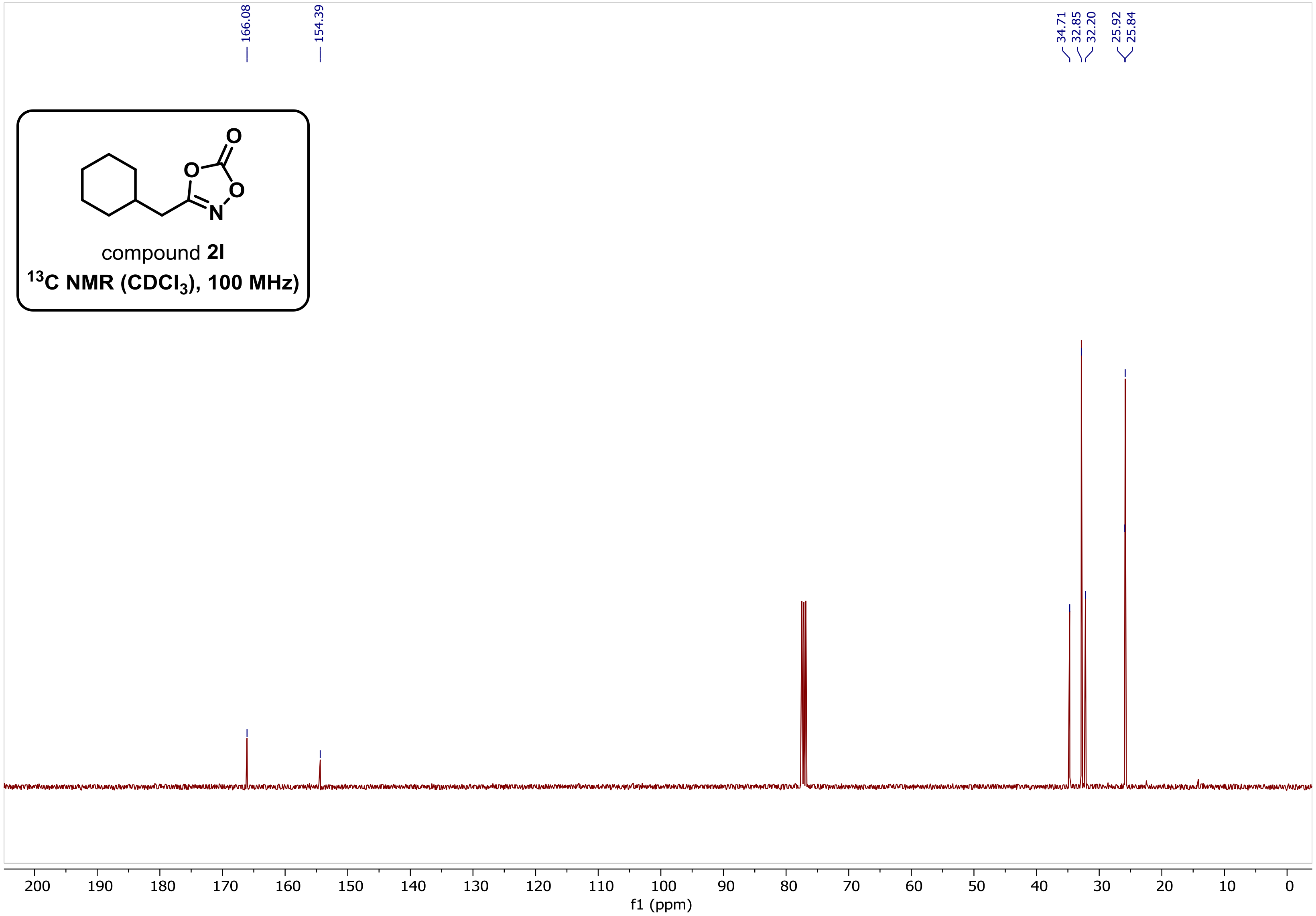
166.08
154.39
34.71
32.85
32.20
25.92
25.84
compound **2l**
^{13}C NMR (CDCl$_3$), 100 MHz)
200 190 180 170 160 150 140 130 120 110 100 90 80 70 60 50 40 30 20 10 0
f1 (ppm)

3.9. Compound 2m

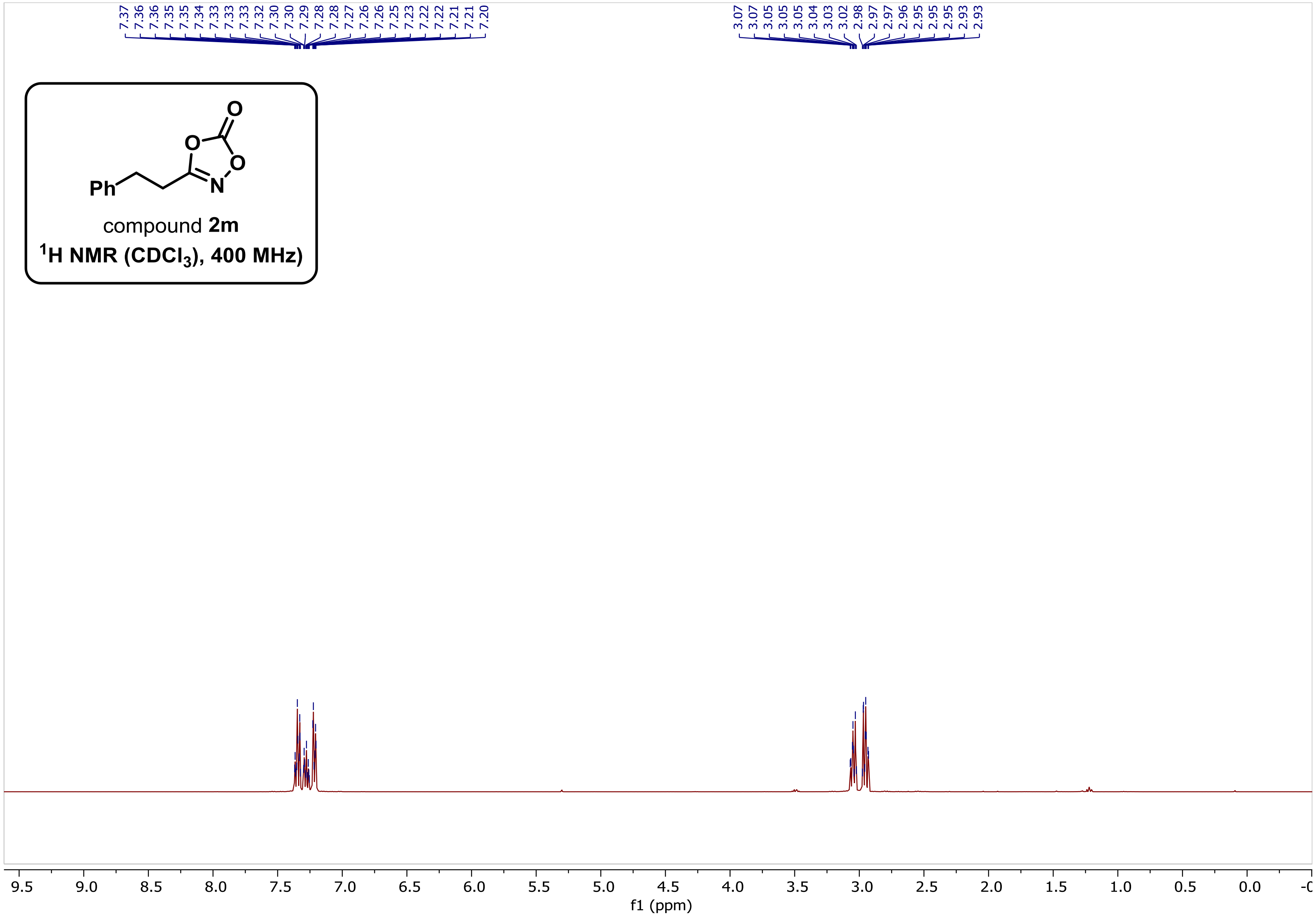

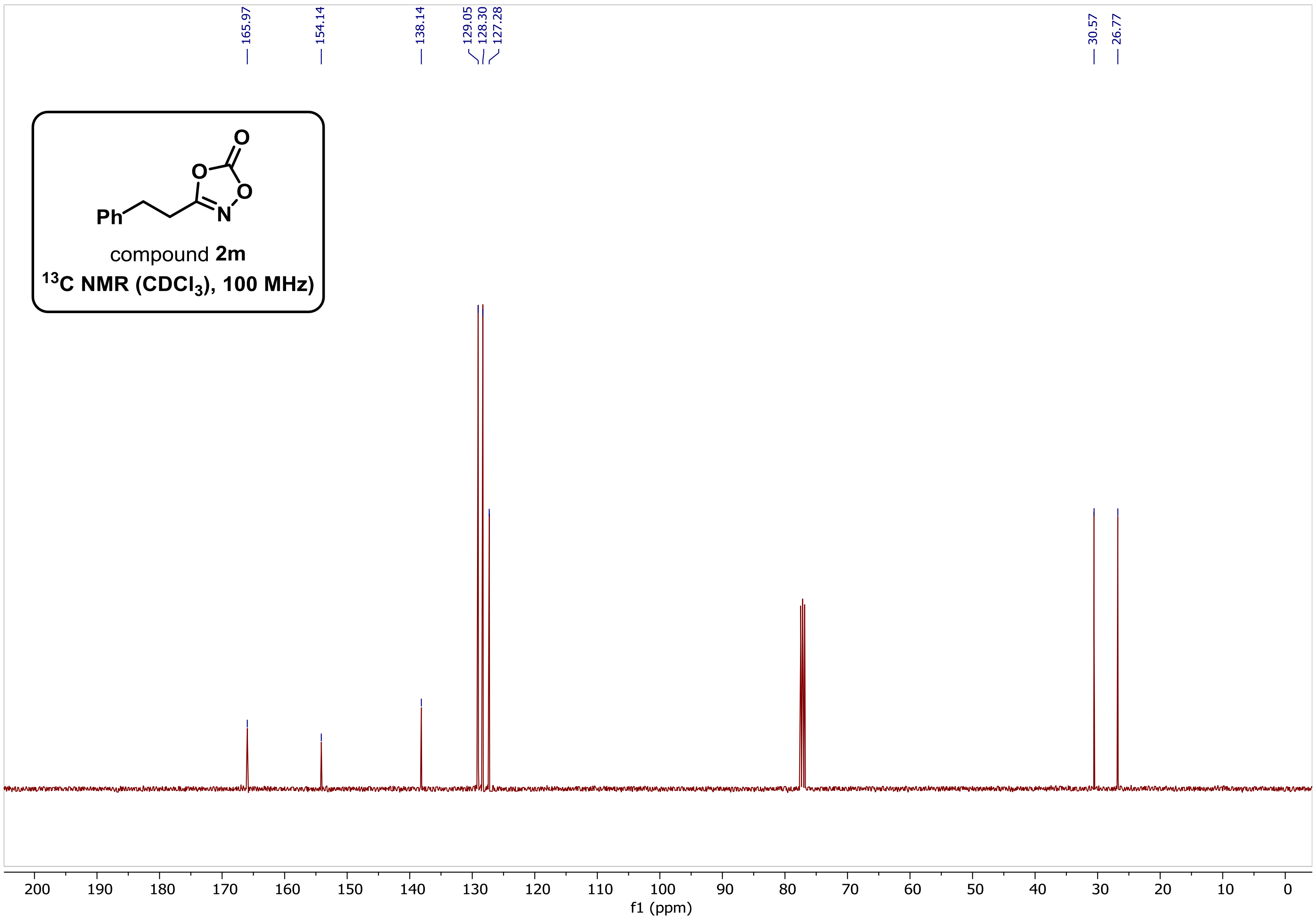

165.97
154.14
138.14
129.05
128.30
127.28
30.57
26.77
Ph
O
N
compound 2m
13C NMR (CDCl3), 100 MHz)
200
190
180
170
160
150
140
130
120
110
100
90
80
70
60
50
40
30
20
10
0
f1 (ppm)

3.10. **Compound 2n**

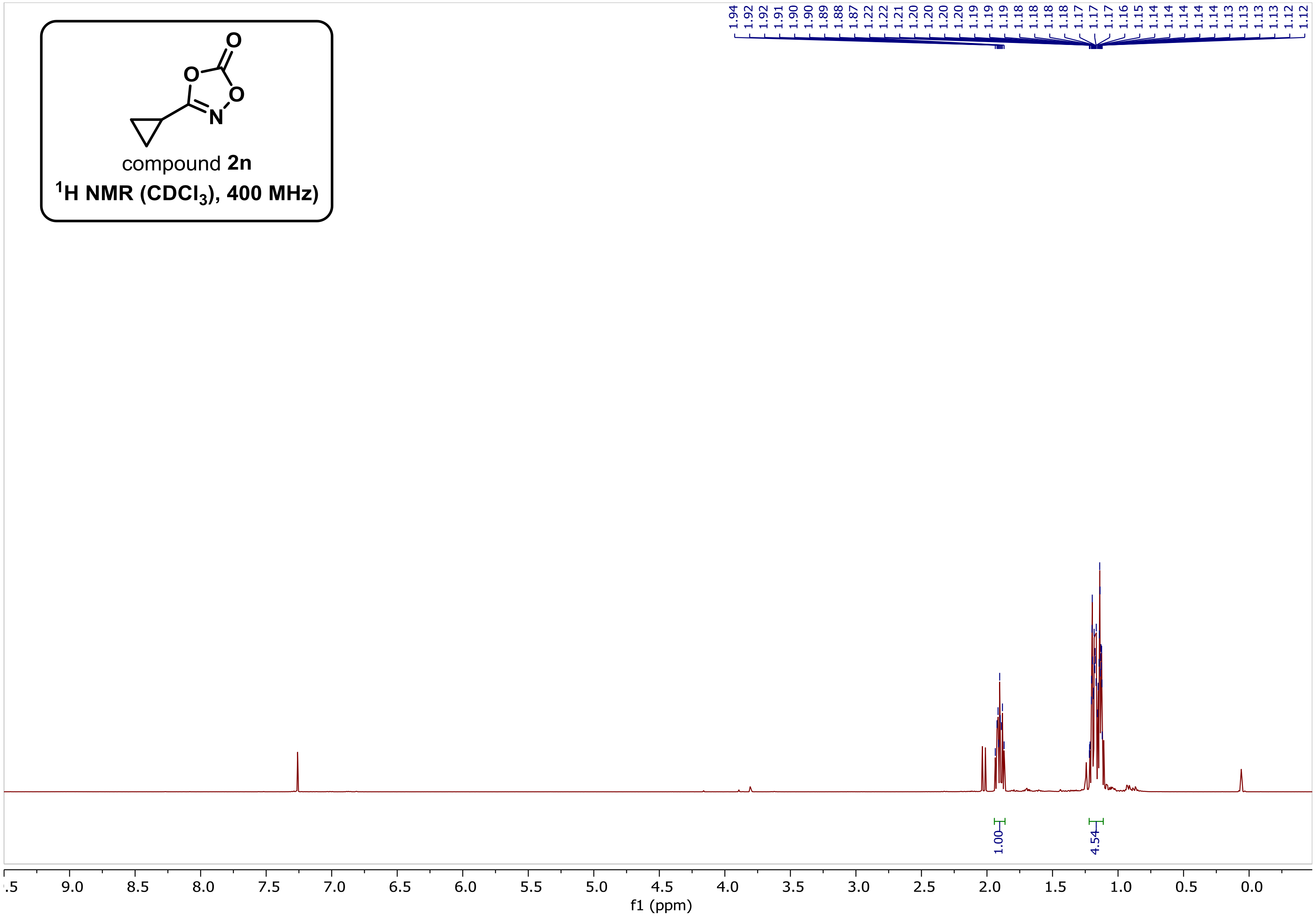

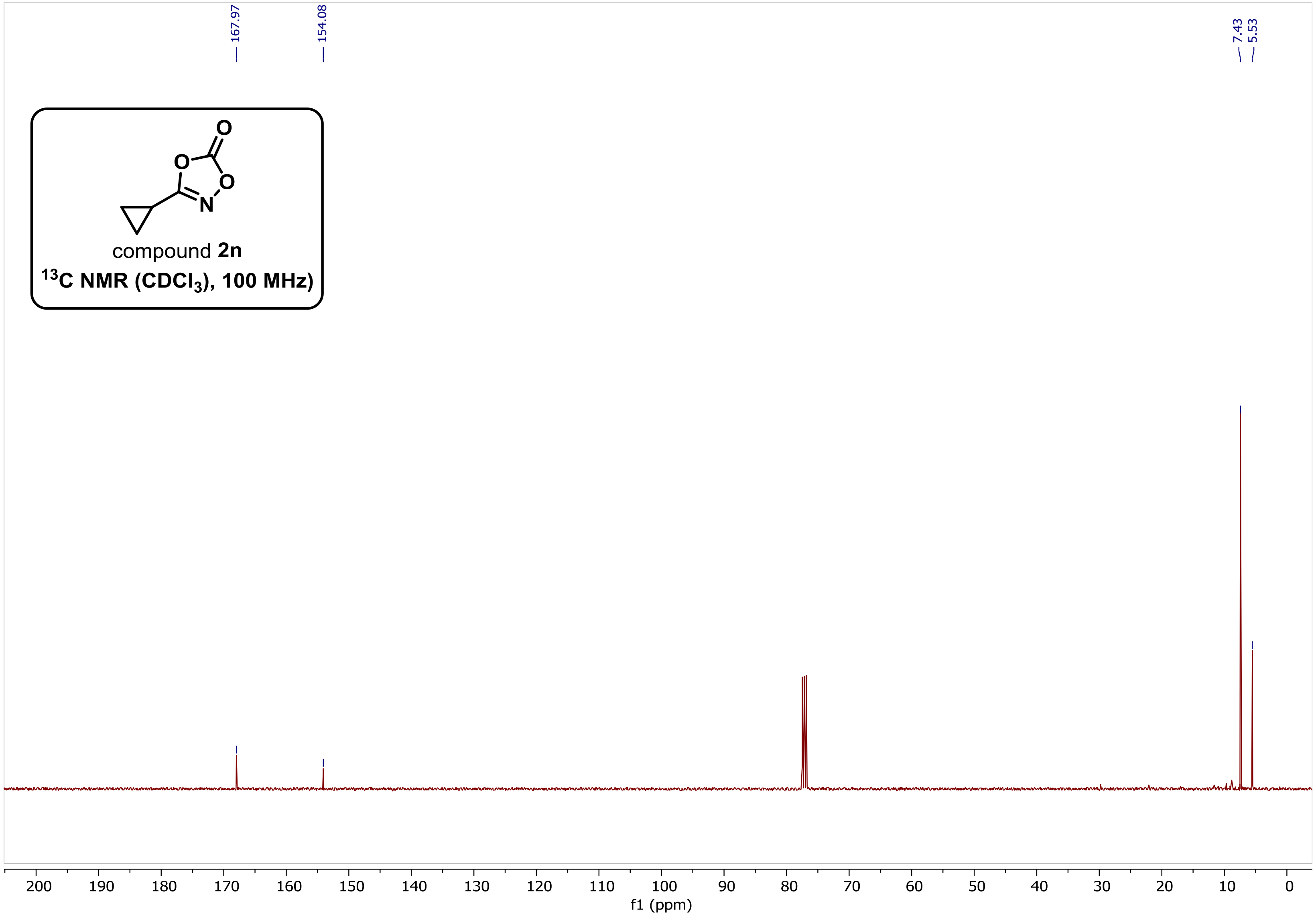

167.97
154.08
7.43
5.53
O
O
O
N
compound 2n
13C NMR (CDCl3), 100 MHz)
200
190
180
170
160
150
140
130
120
110
100
90
80
70
60
50
40
30
20
10
0
f1 (ppm)

3.11. Compound 2o

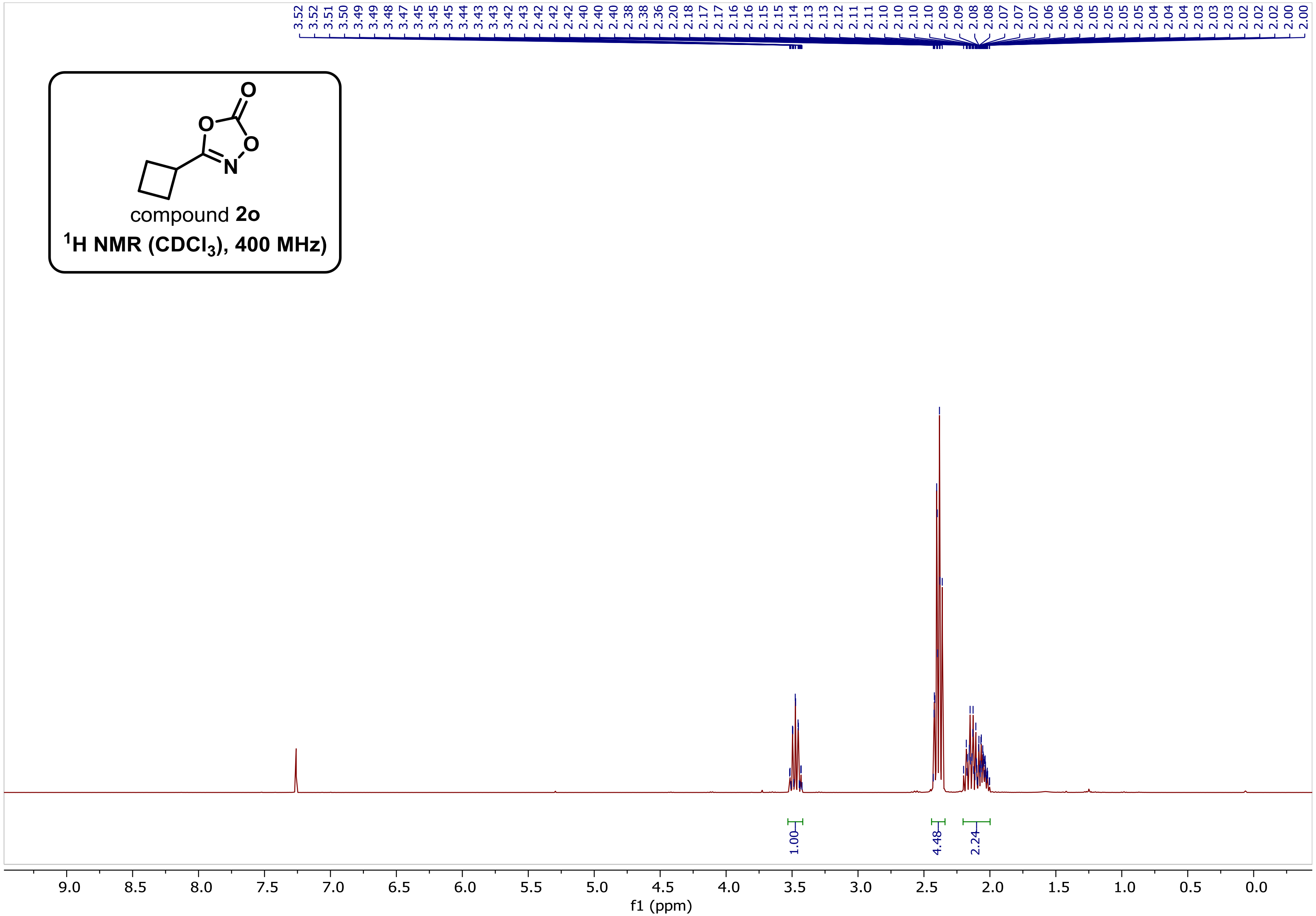

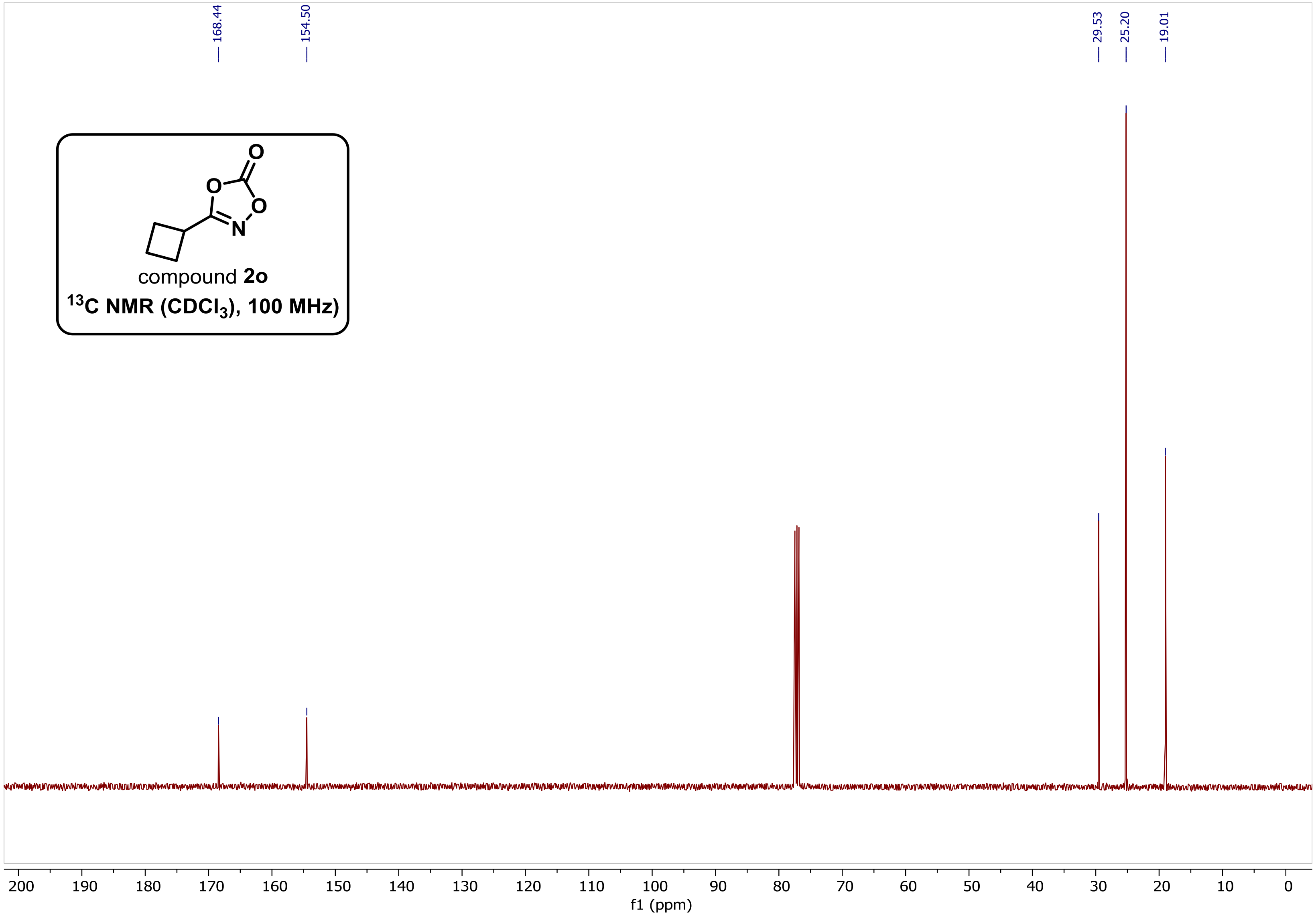

168.44
154.50
29.53
25.20
19.01
O
O
O
N
compound **2o**
^{13}C NMR (CDCl$_3$), 100 MHz)
200
190
180
170
160
150
140
130
120
110
100
90
80
70
60
50
40
30
20
10
0
f1 (ppm)

3.12. Compound 2b

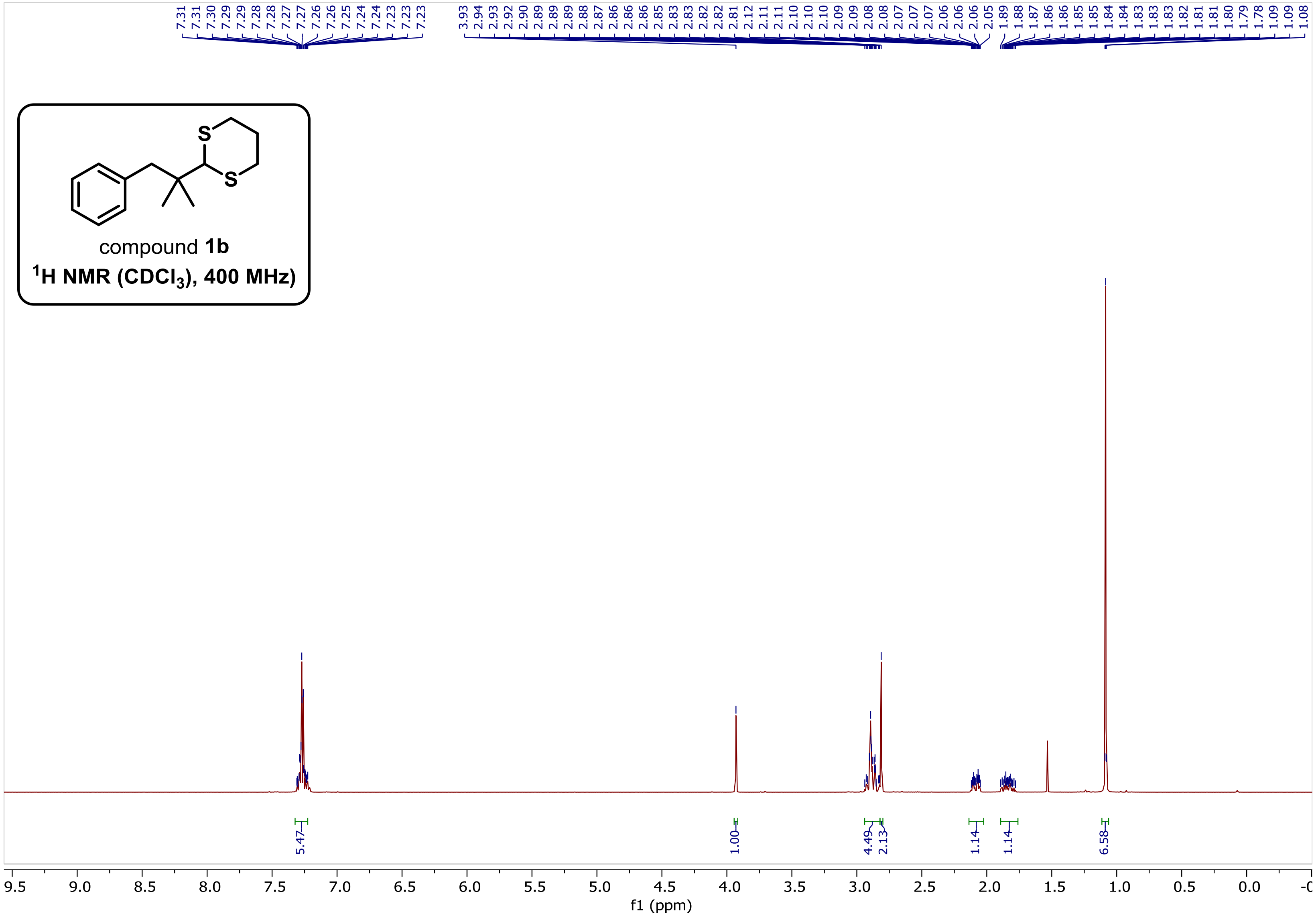

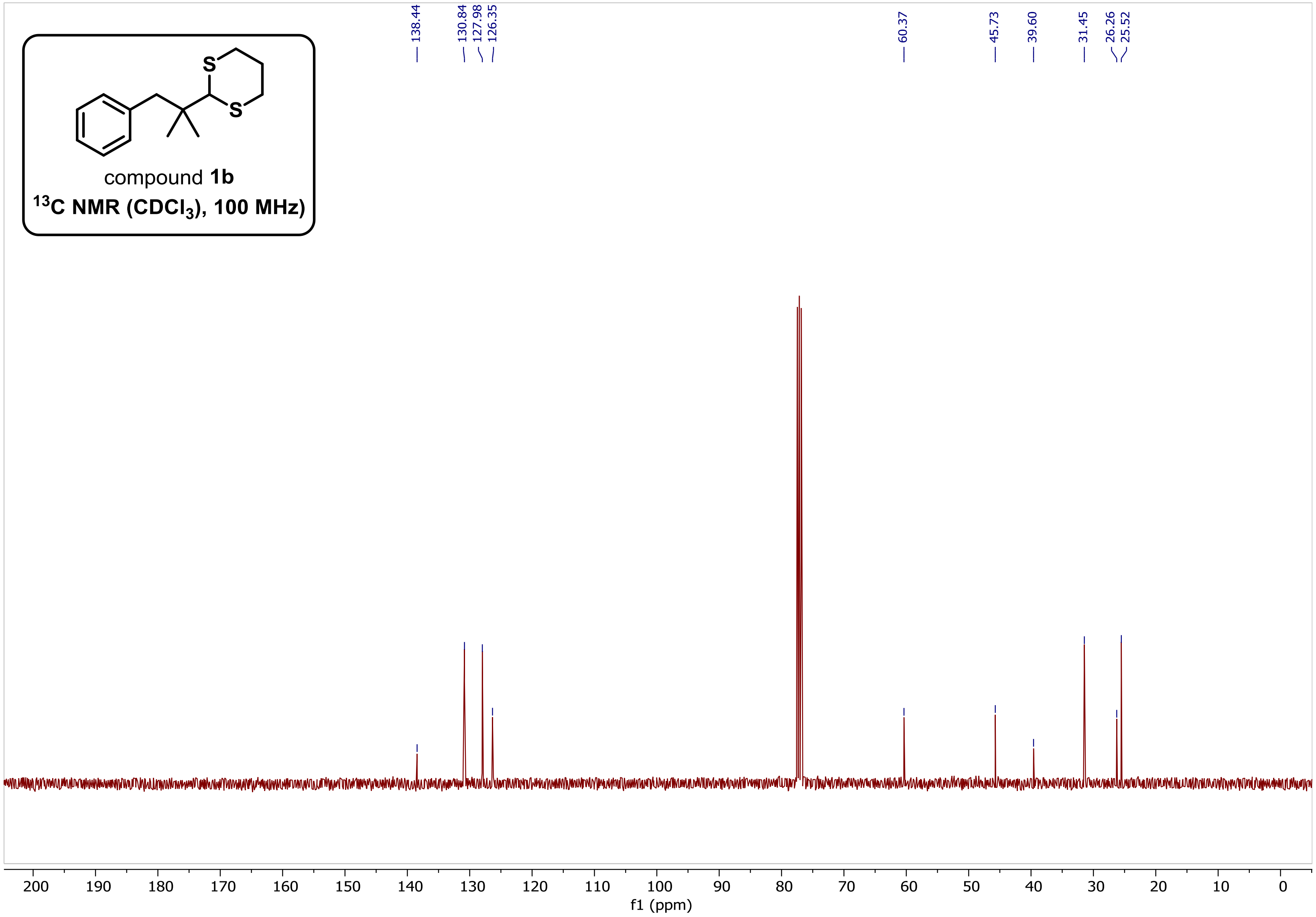

compound 1b
^{13}C NMR ($CDCl_3$), 100 MHz)
138.44
130.84
127.98
126.35
60.37
45.73
39.60
31.45
26.26
25.52
200 190 180 170 160 150 140 130 120 110 100 90 80 70 60 50 40 30 20 10 0
f1 (ppm)

3.13. Compound 1c

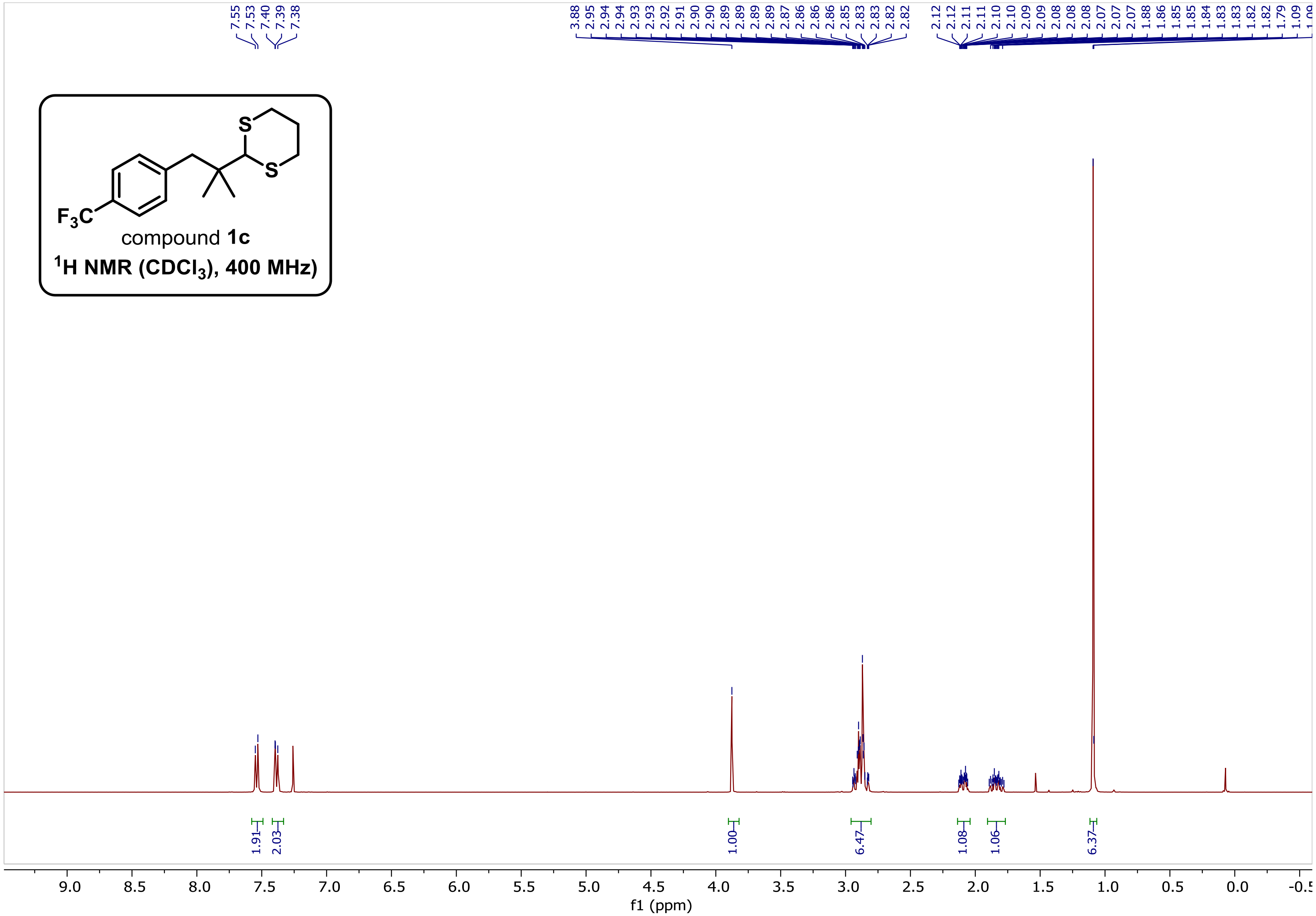

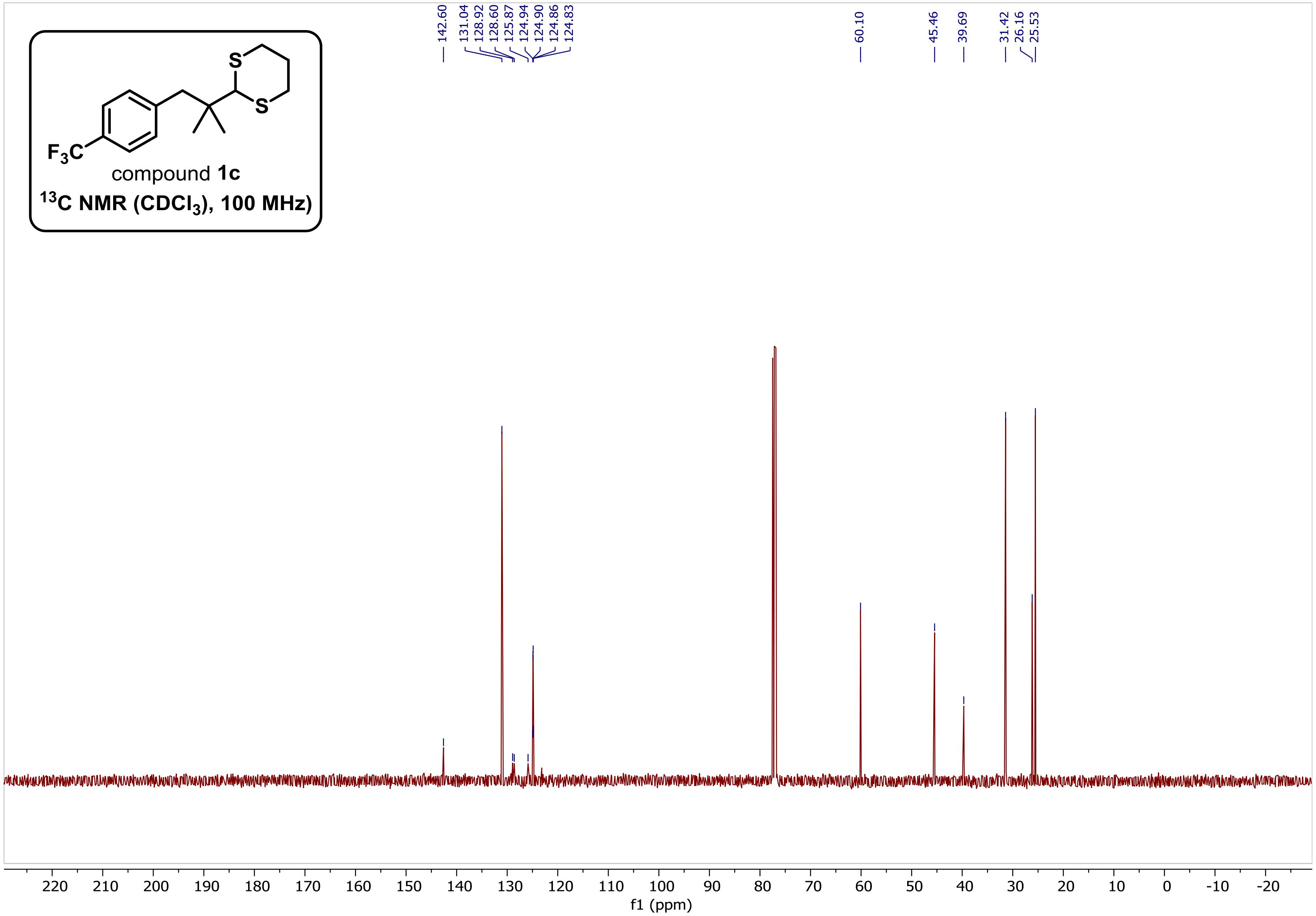

F3C
S
S
compound 1c
13C NMR (CDCl3), 100 MHz)
142.60
131.04
128.92
128.60
125.87
124.94
124.90
124.86
124.83
60.10
45.46
39.69
31.42
26.16
25.53
220 210 200 190 180 170 160 150 140 130 120 110 100 90 80 70 60 50 40 30 20 10 0 -10 -20
f1 (ppm)

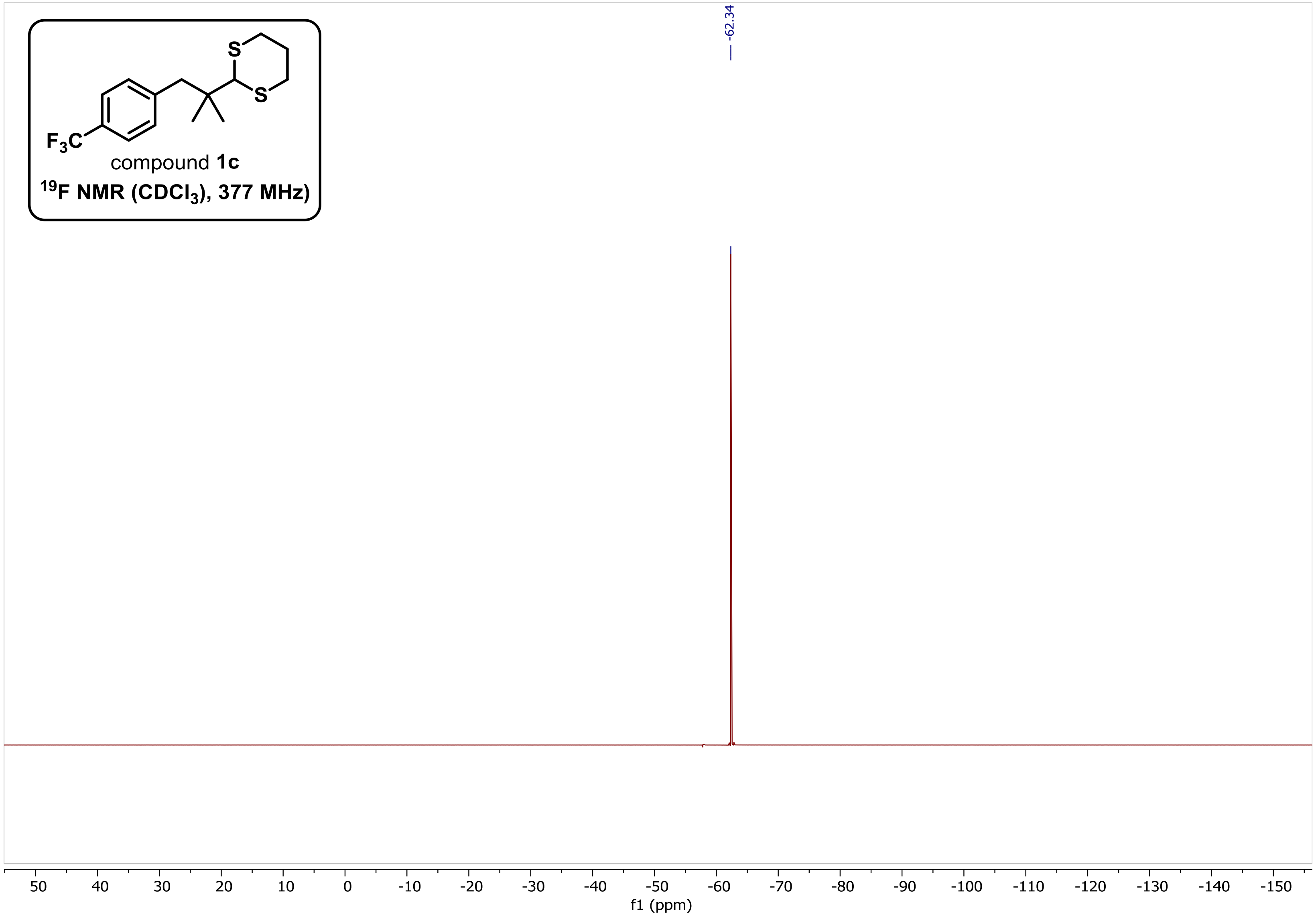
-62.34
F3C
S
S
compound 1c
19F NMR (CDCl3), 377 MHz)
50 40 30 20 10 0 -10 -20 -30 -40 -50 -60 -70 -80 -90 -100 -110 -120 -130 -140 -150
f1 (ppm)

3.14. Compound 1d

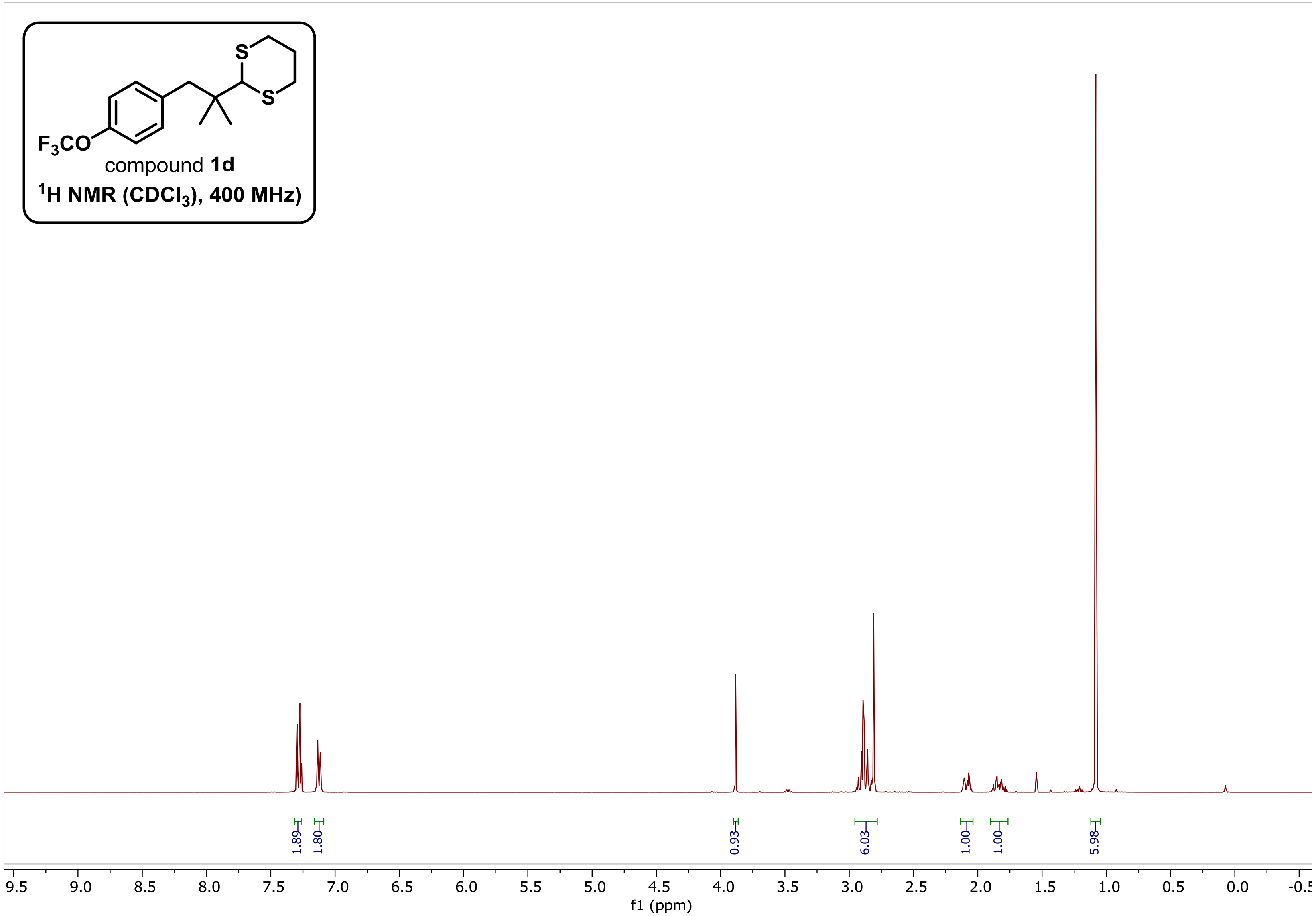

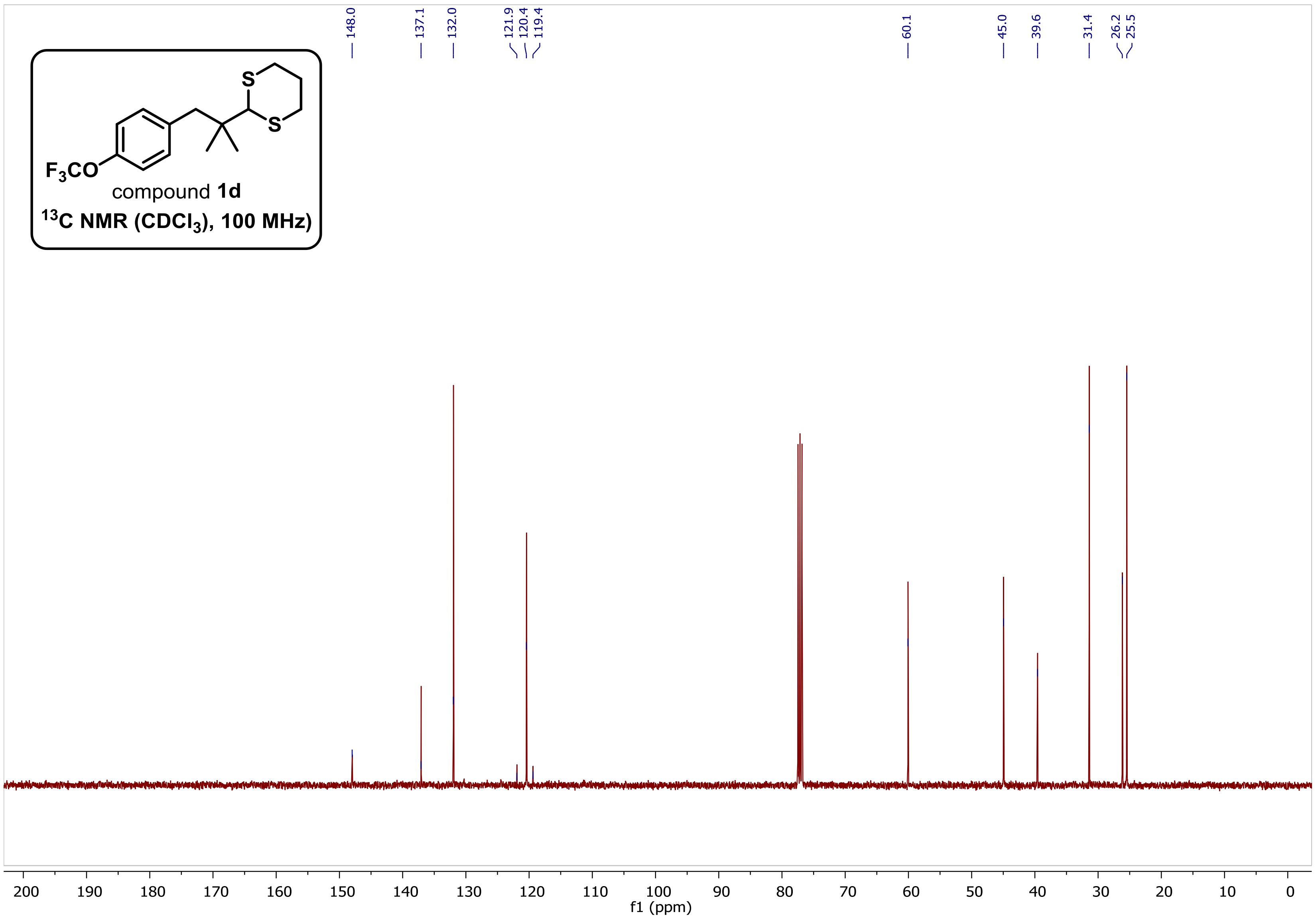
148.0
137.1
132.0
121.9
120.4
119.4
60.1
45.0
39.6
31.4
26.2
25.5
F3CO
S
S
compound 1d
13C NMR (CDCl3), 100 MHz)
200
190
180
170
160
150
140
130
120
110
100
90
80
70
60
50
40
30
20
10
0
f1 (ppm)

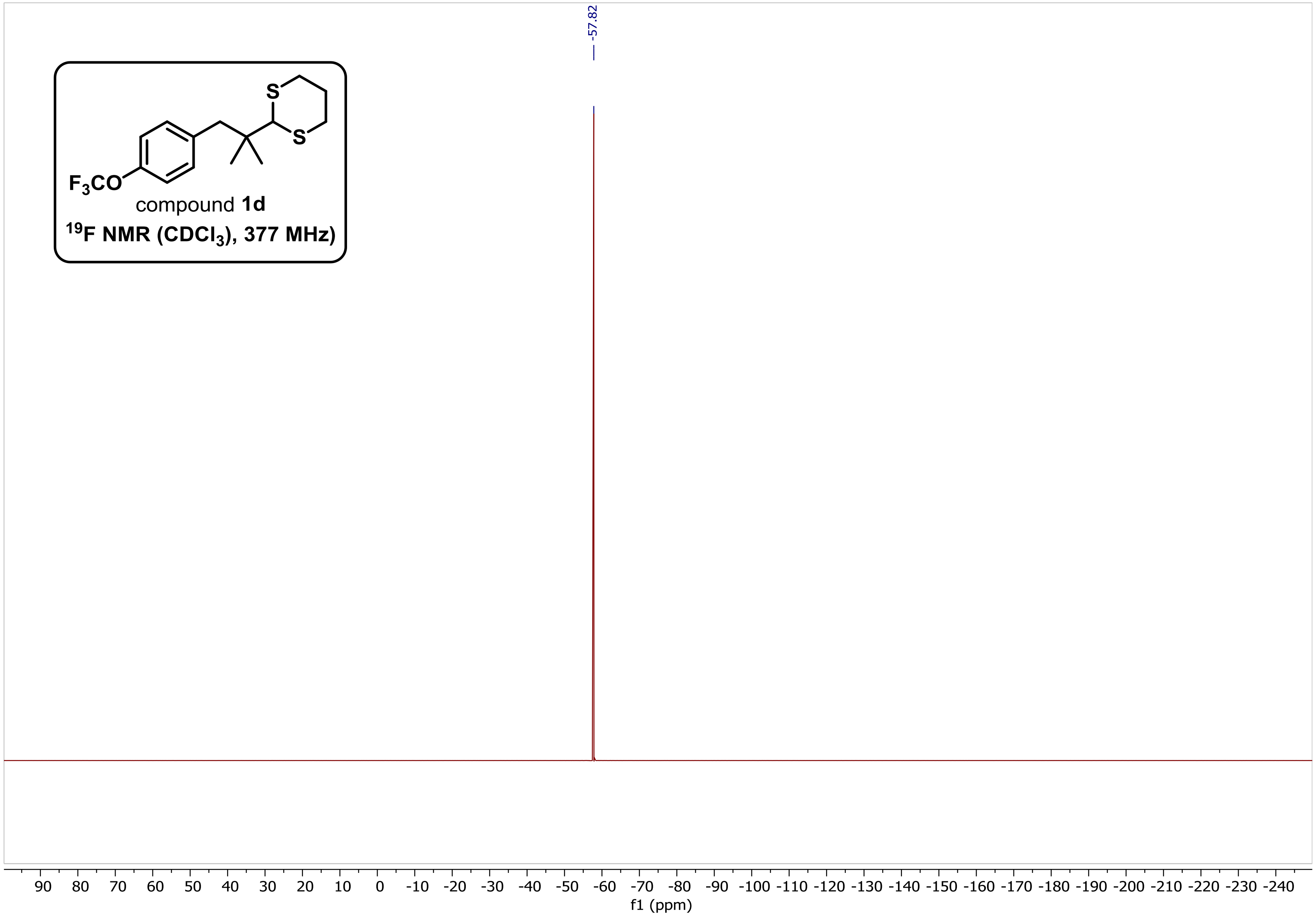
-57.82
F3CO
S
S
compound 1d
19F NMR (CDCl3), 377 MHz)
90 80 70 60 50 40 30 20 10 0 -10 -20 -30 -40 -50 -60 -70 -80 -90 -100 -110 -120 -130 -140 -150 -160 -170 -180 -190 -200 -210 -220 -230 -240
f1 (ppm)

3.15. Compound 1e

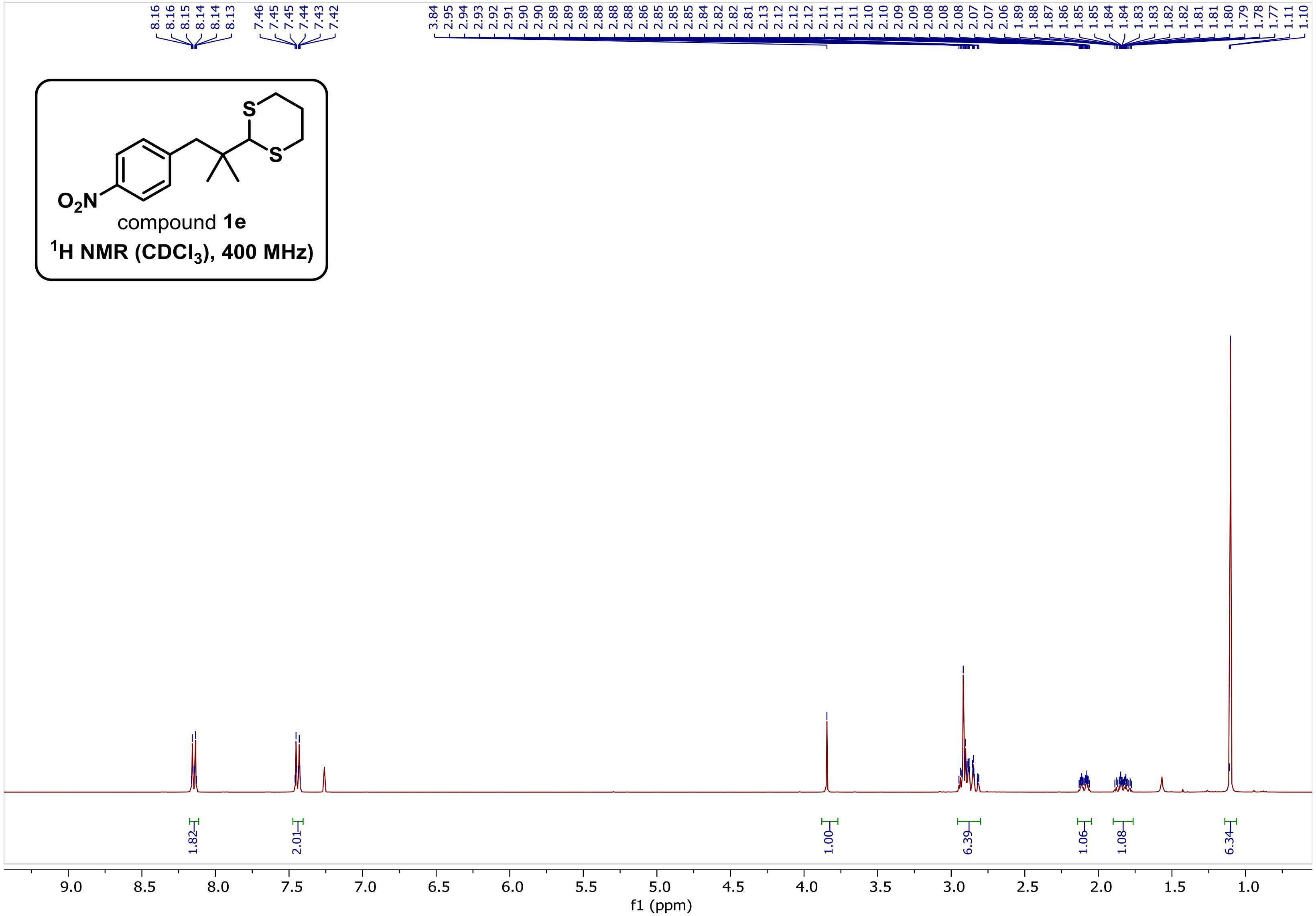

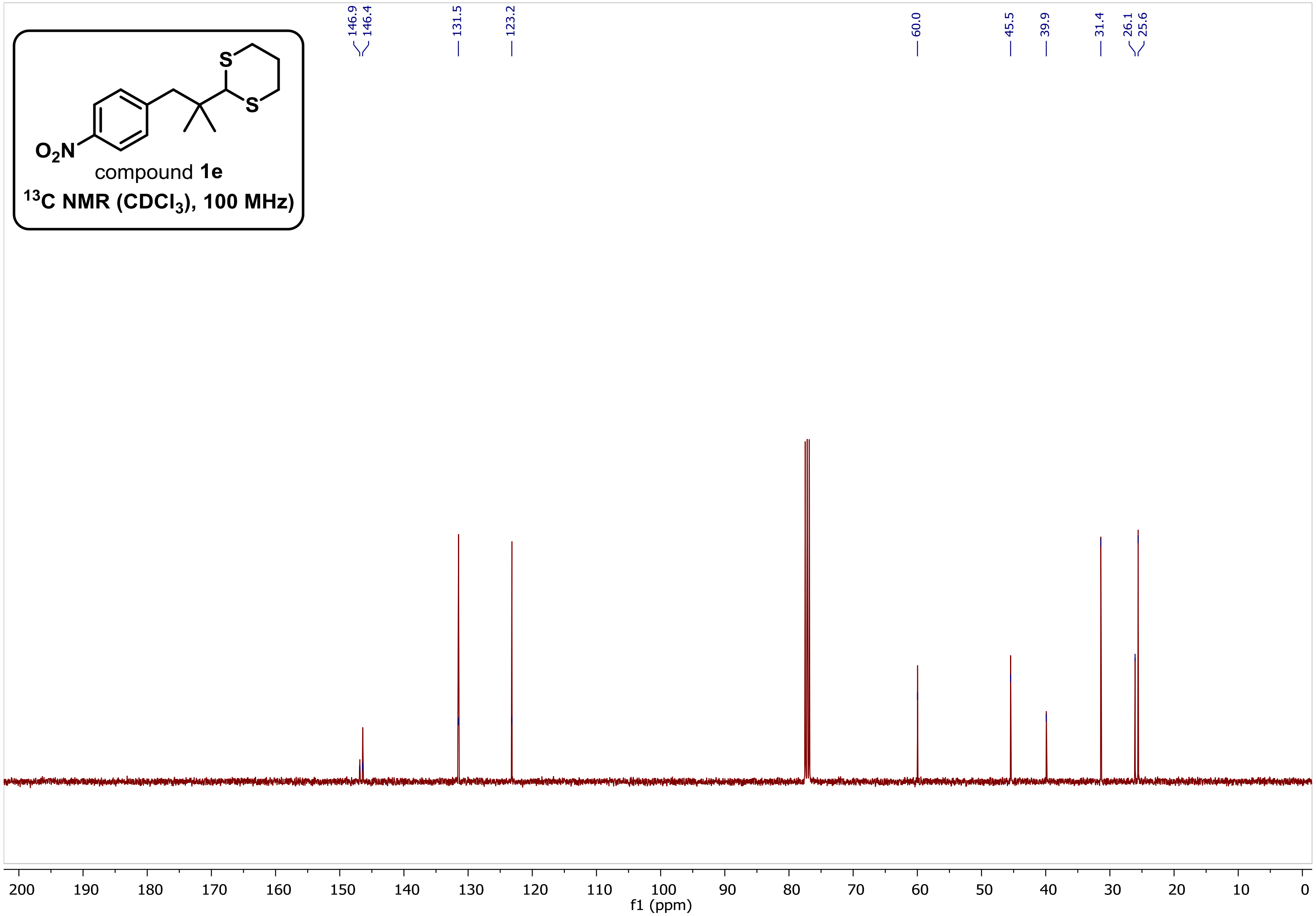

O2N
compound 1e
13C NMR (CDCl3), 100 MHz)
146.9
146.4
131.5
123.2
60.0
45.5
39.9
31.4
26.1
25.6
200 190 180 170 160 150 140 130 120 110 100 90 80 70 60 50 40 30 20 10 0
f1 (ppm)

3.16. Compound 1f

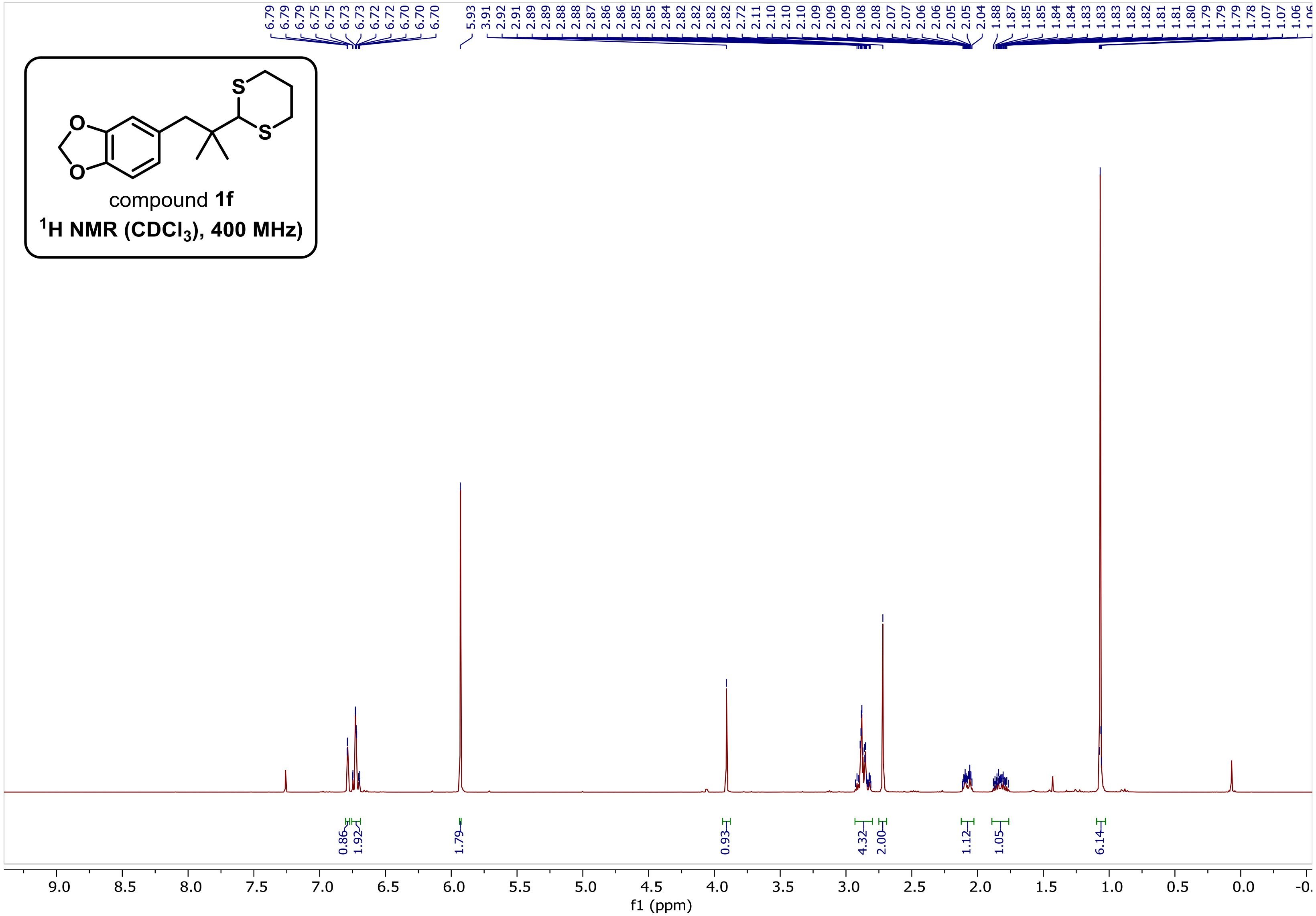

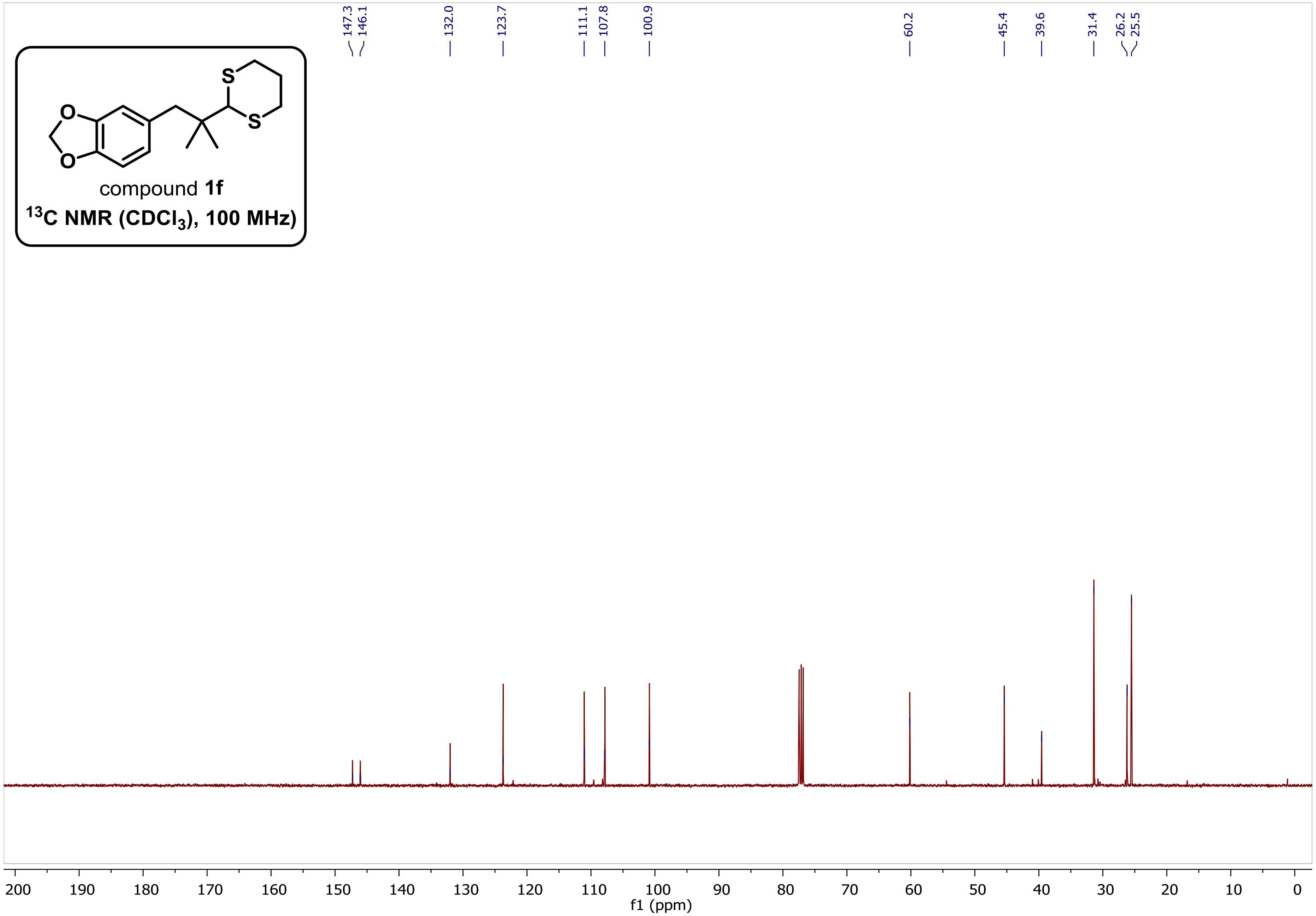
compound **1f**
^{13}C NMR ($CDCl_3$), 100 MHz)
147.3
146.1
132.0
123.7
111.1
107.8
100.9
60.2
45.4
39.6
31.4
26.2
25.5
200
190
180
170
160
150
140
130
120
110
100
90
80
70
60
50
40
30
20
10
0
f1 (ppm)

3.17. Compound 1g

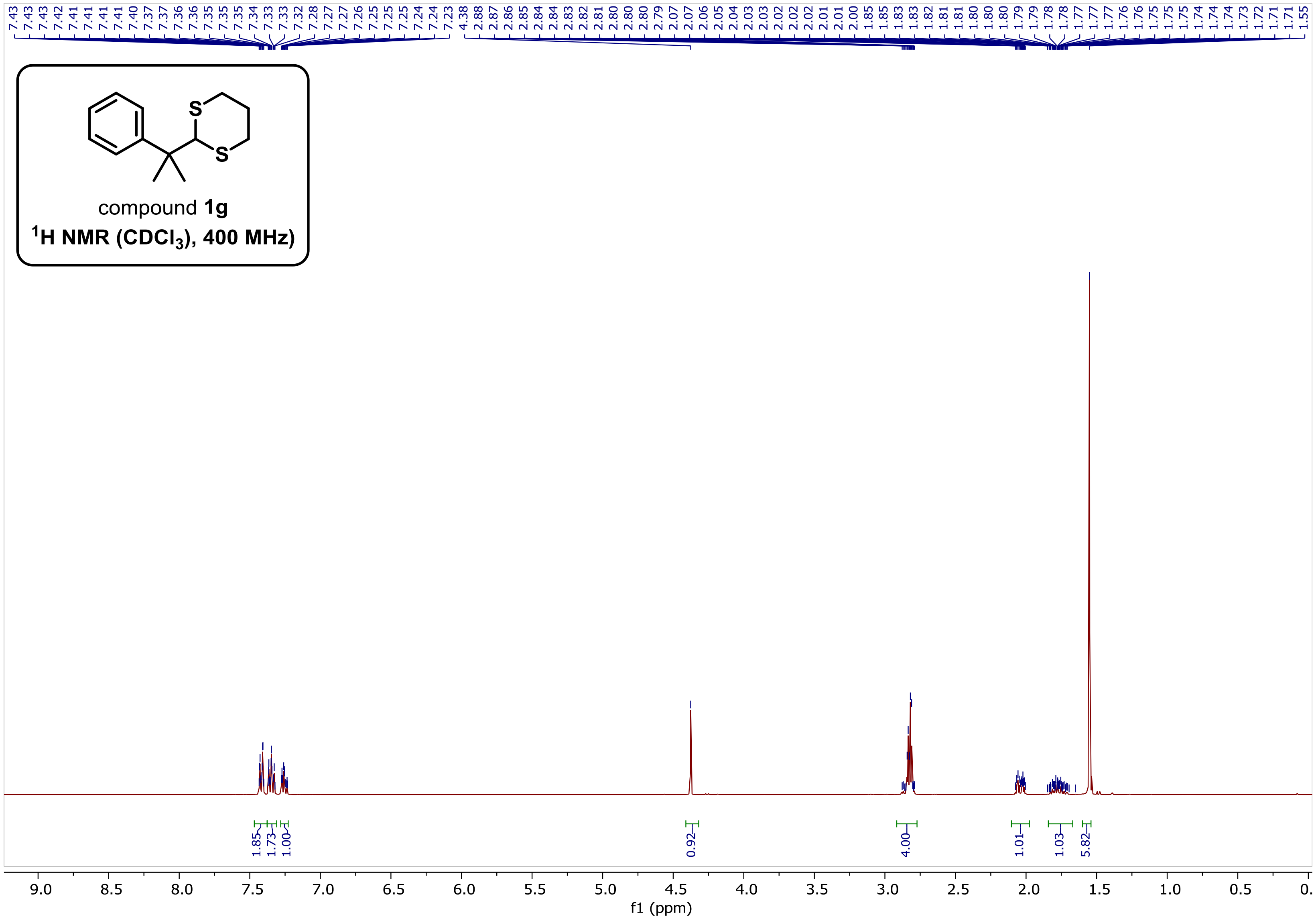

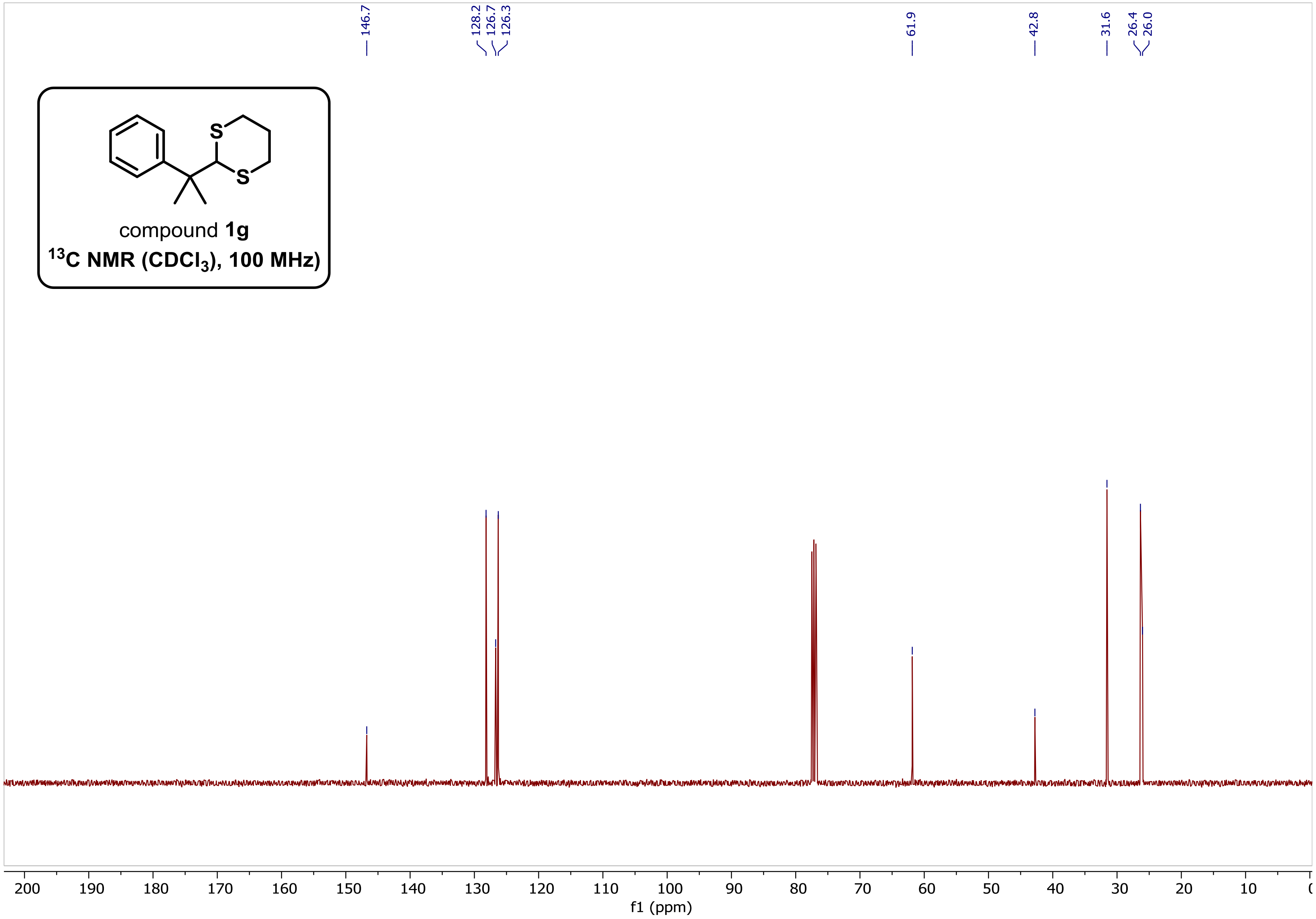
146.7
128.2
126.7
126.3
61.9
42.8
31.6
26.4
26.0
S
S
compound 1g
13C NMR (CDCl3), 100 MHz)
200
190
180
170
160
150
140
130
120
110
100
90
80
70
60
50
40
30
20
10
f1 (ppm)

3.18. Compound 1h

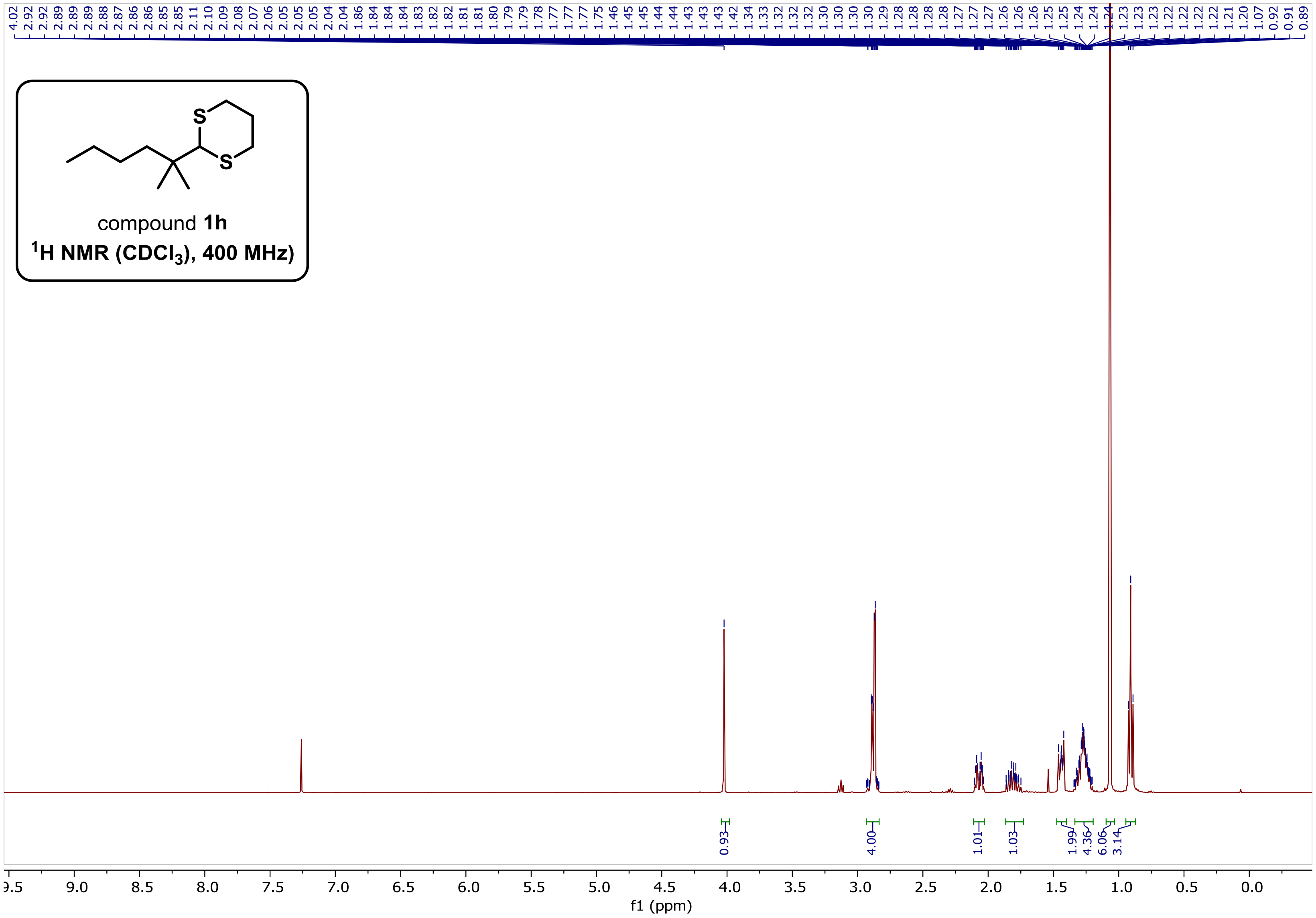

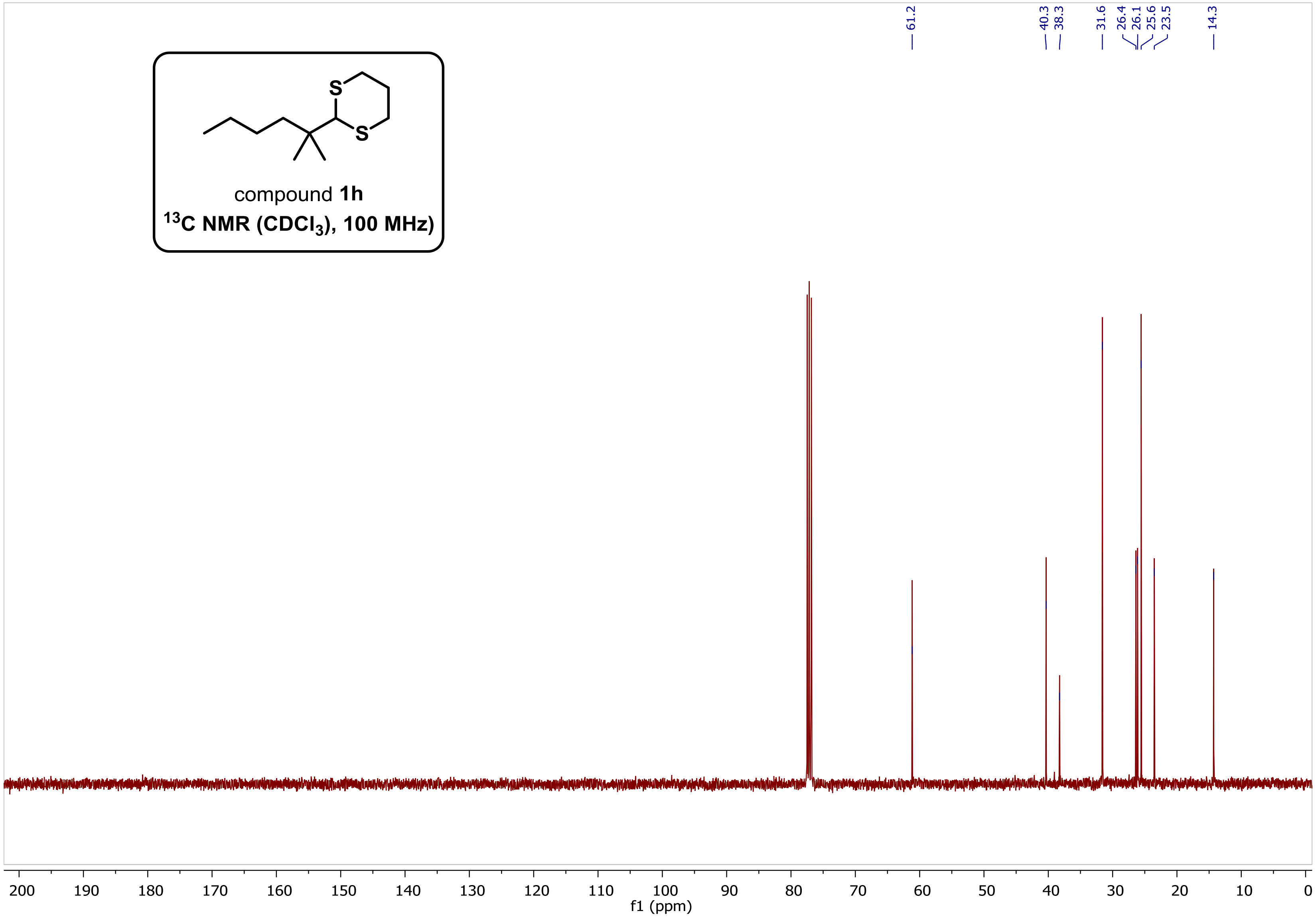
compound 1h
13C NMR (CDCl3), 100 MHz)
61.2
40.3
38.3
31.6
26.4
26.1
25.6
23.5
14.3
200 190 180 170 160 150 140 130 120 110 100 90 80 70 60 50 40 30 20 10 0
f1 (ppm)

3.19. Compound 1i

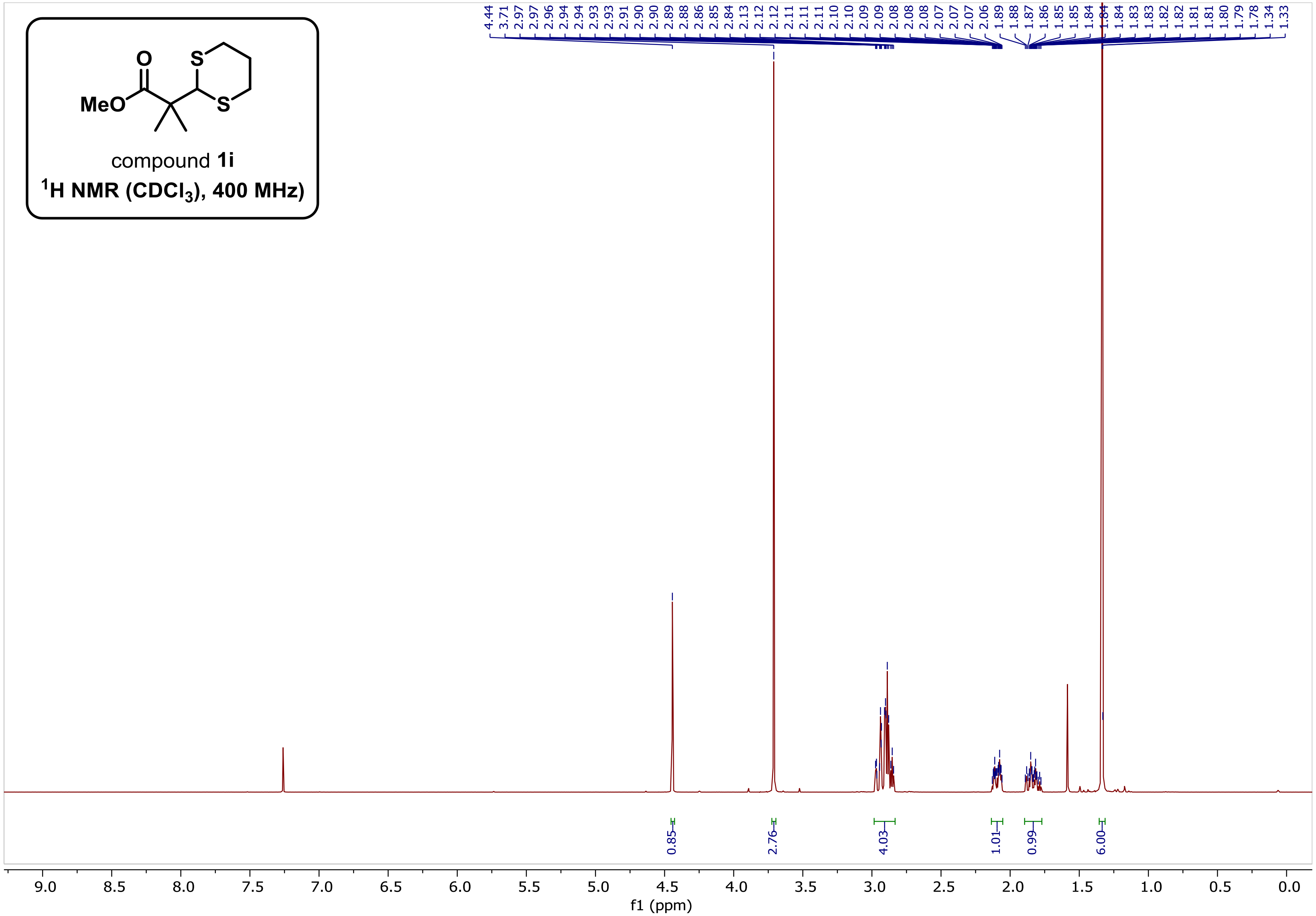

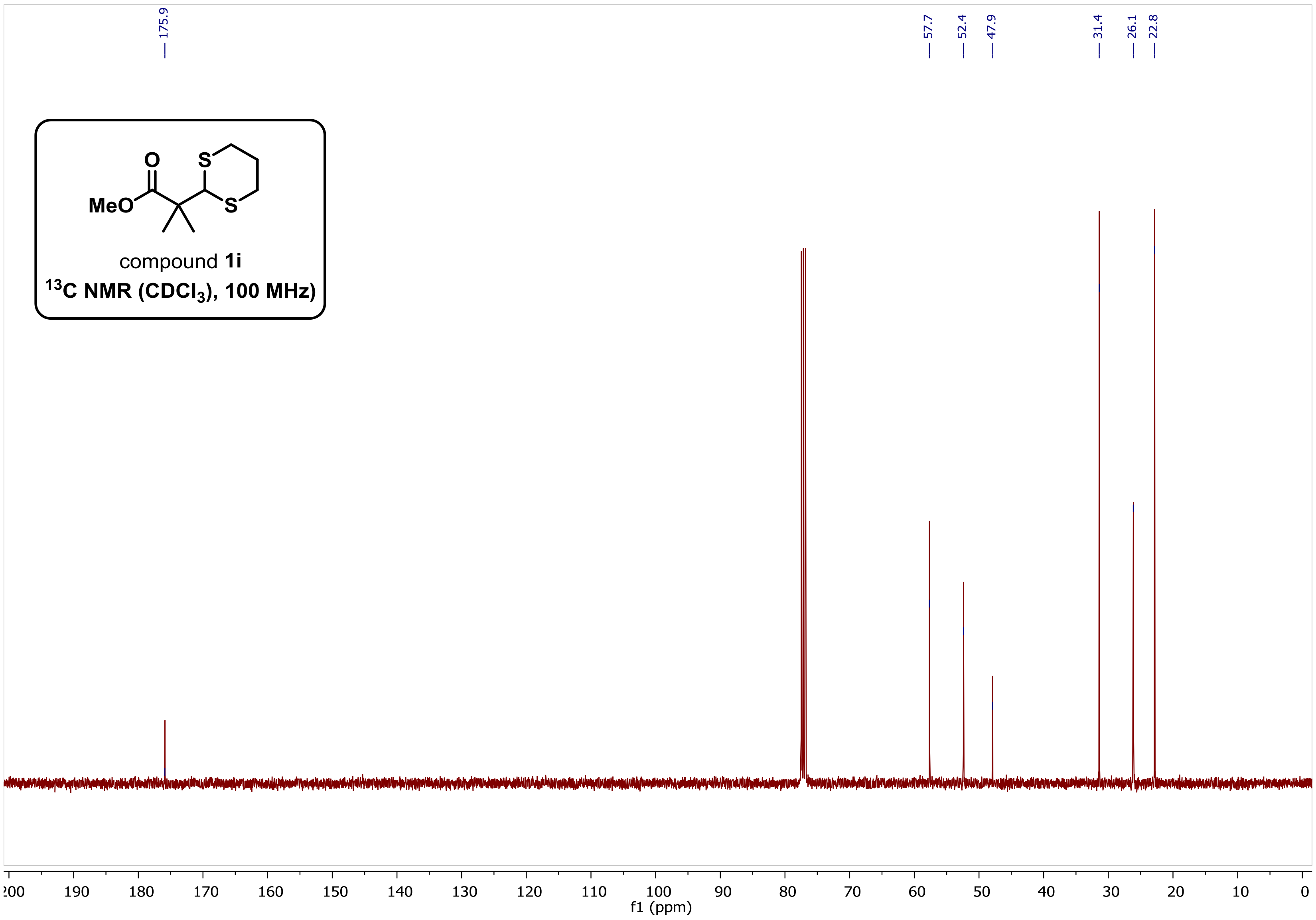
175.9
57.7
52.4
47.9
31.4
26.1
22.8
MeO
O
S
S
compound **1i**
^{13}C NMR ($CDCl_3$), 100 MHz)
200 190 180 170 160 150 140 130 120 110 100 90 80 70 60 50 40 30 20 10 0
f1 (ppm)

3.20. Compound 1j

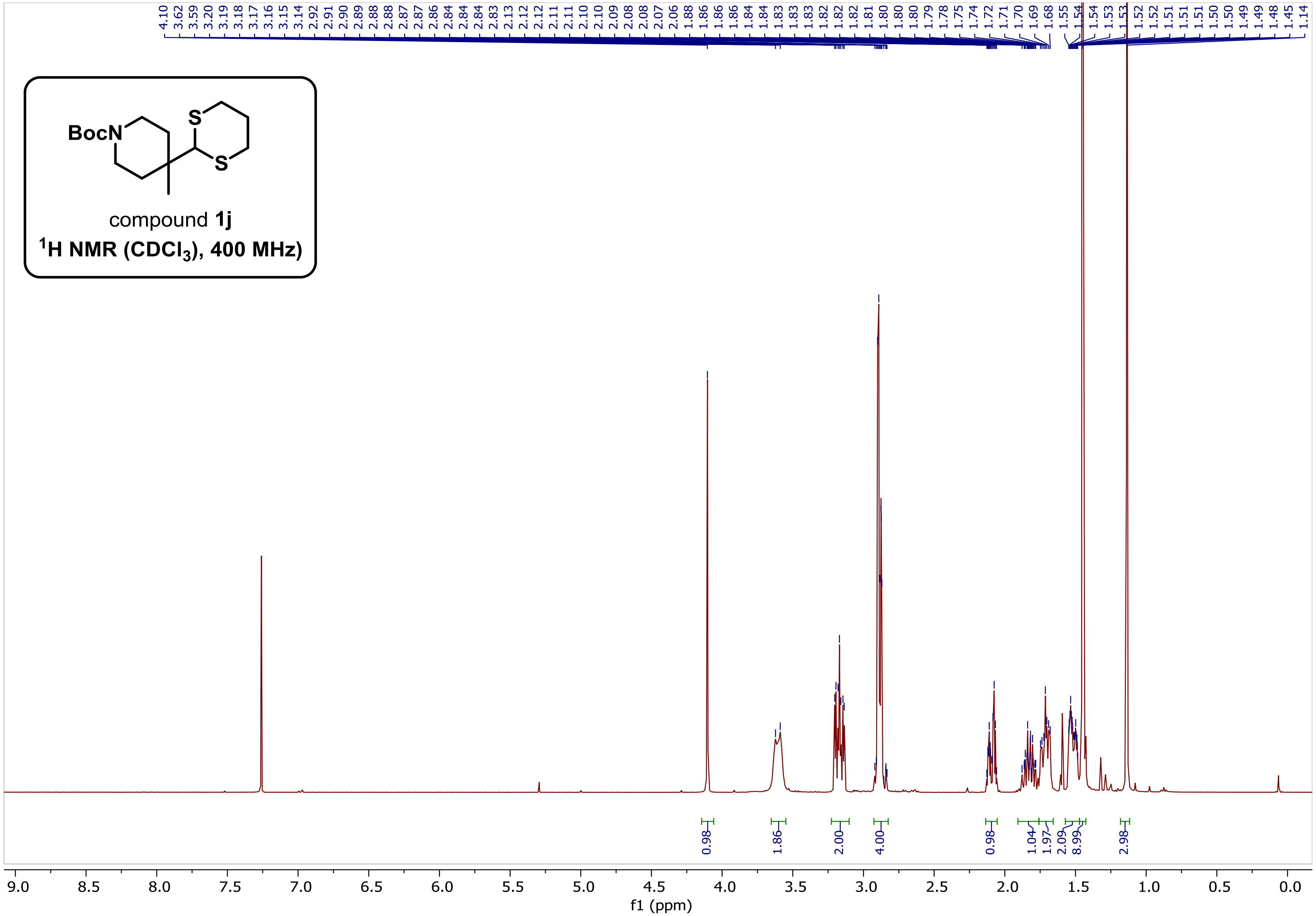

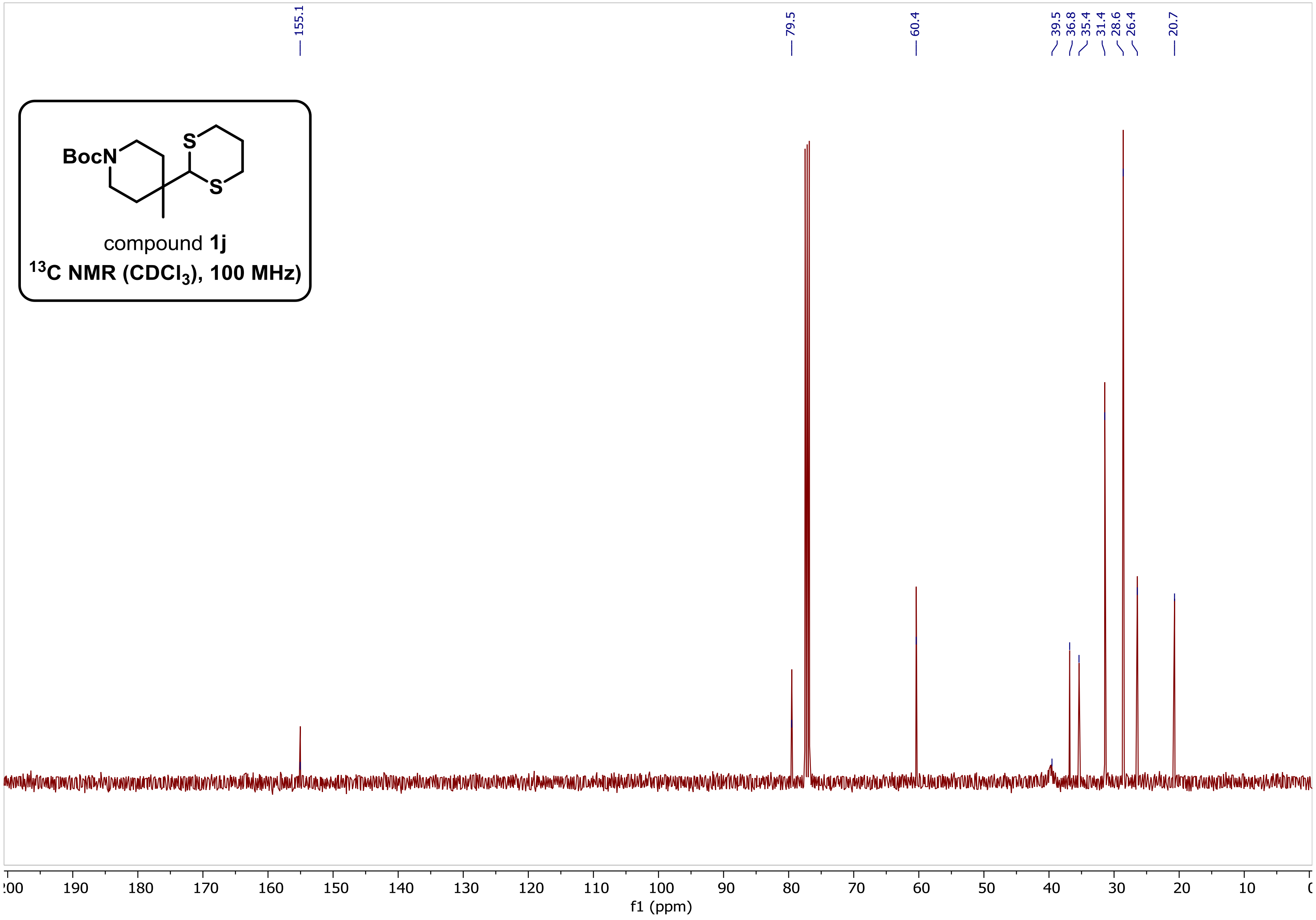
155.1
79.5
60.4
39.5
36.8
35.4
31.4
28.6
26.4
20.7
BocN
S
S
compound 1j
13C NMR (CDCl3), 100 MHz)
200
190
180
170
160
150
140
130
120
110
100
90
80
70
60
50
40
30
20
10
0
f1 (ppm)

3.21. Compound 3a

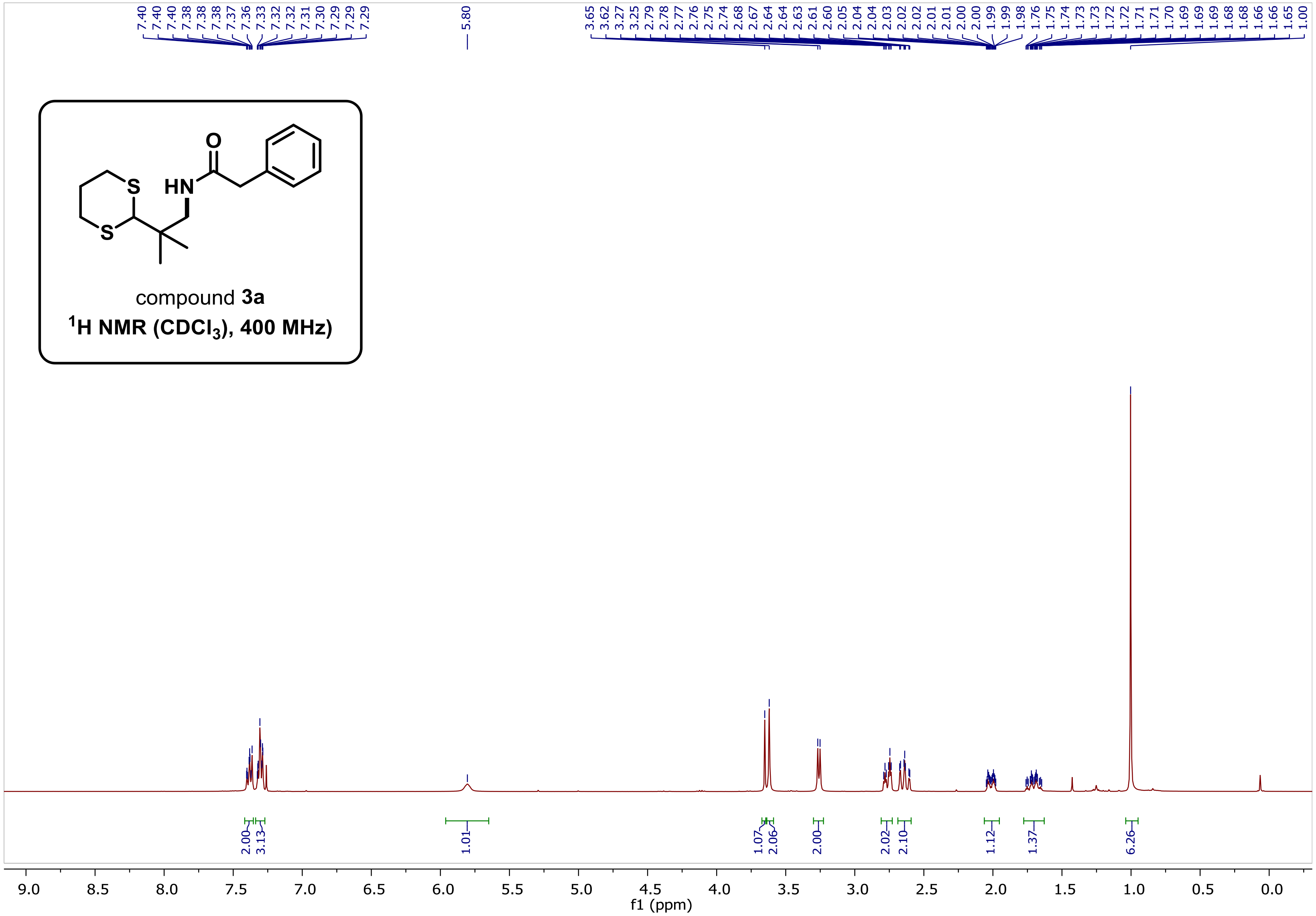

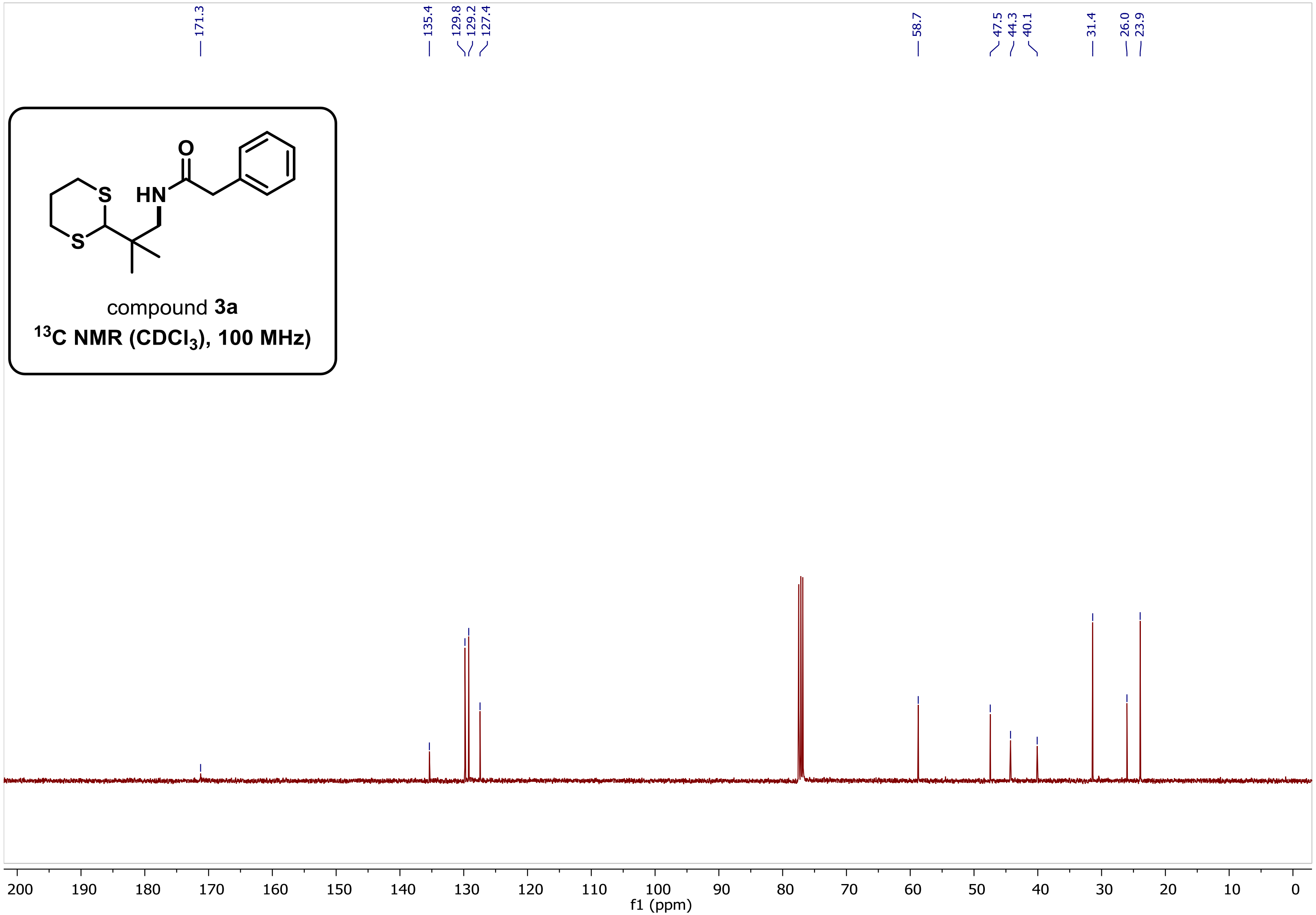

171.3
135.4
129.8
129.2
127.4
58.7
47.5
44.3
40.1
31.4
26.0
23.9
S
S
HN
O
compound 3a
13C NMR (CDCl3), 100 MHz)
200 190 180 170 160 150 140 130 120 110 100 90 80 70 60 50 40 30 20 10 0
f1 (ppm)

3.22. Compound 3b

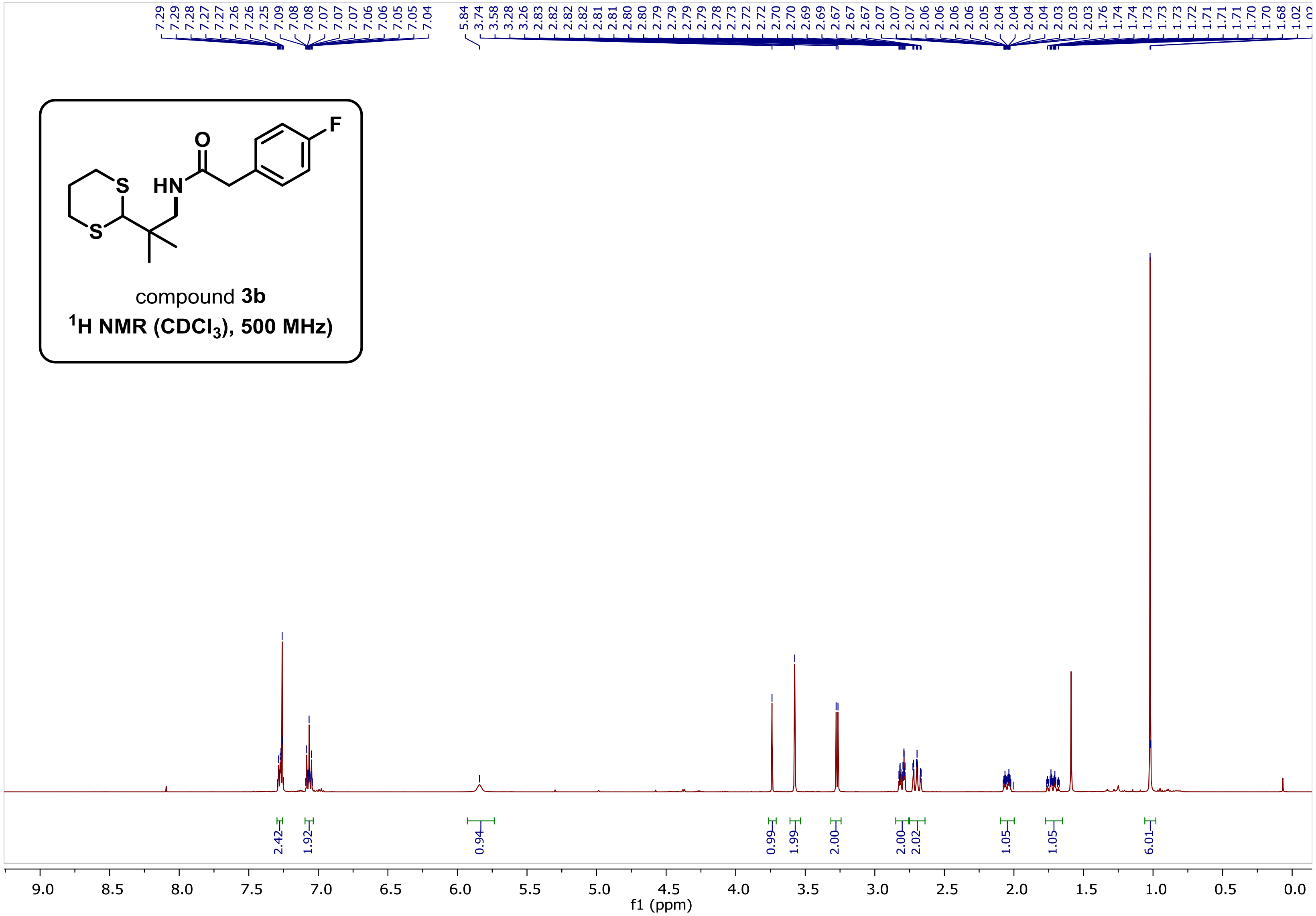

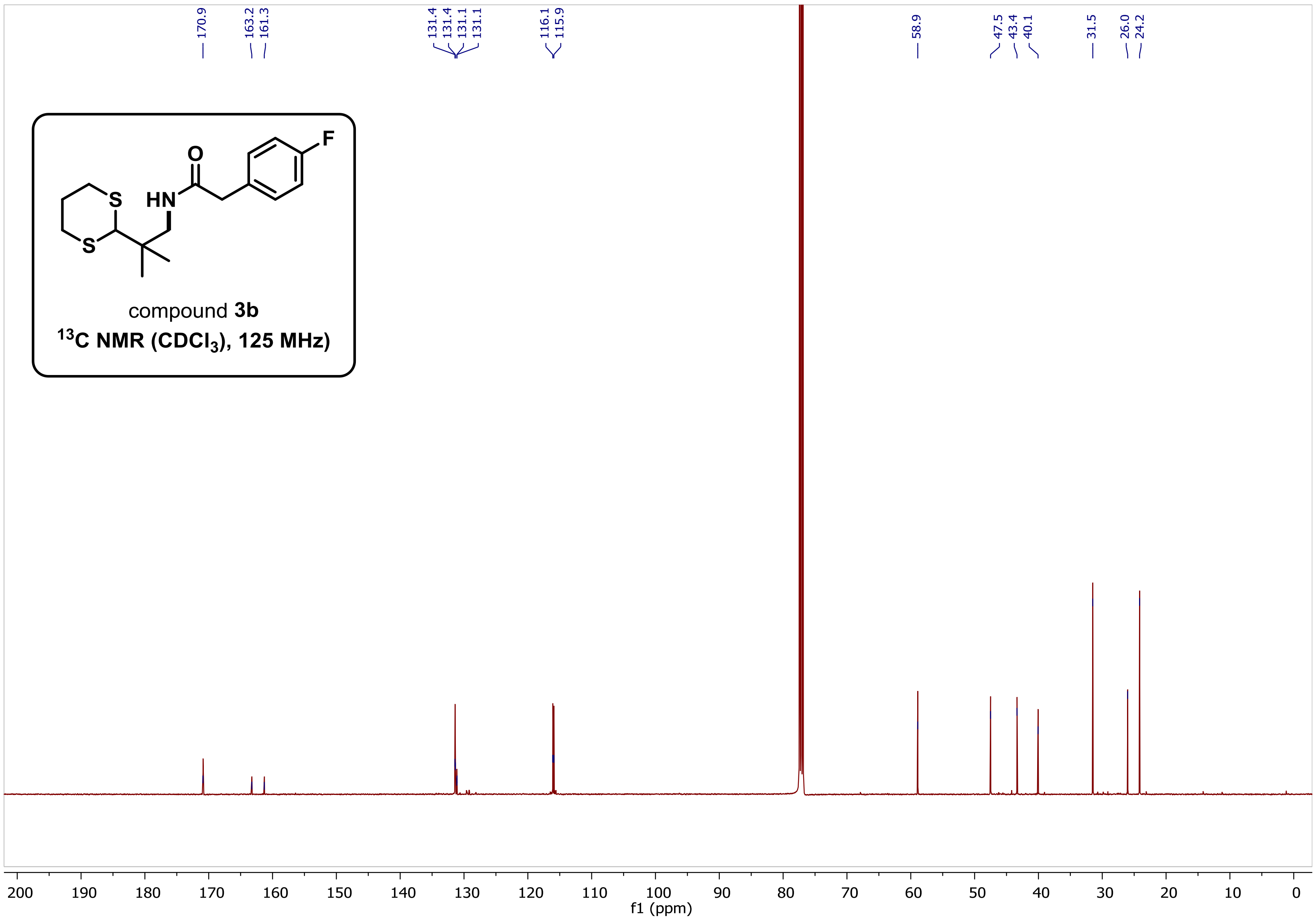

170.9
163.2
161.3
131.4
131.4
131.1
131.1
116.1
115.9
58.9
47.5
43.4
40.1
31.5
26.0
24.2
O
F
HN
S
S
compound 3b
^{13}C NMR ($CDCl_3$), 125 MHz)
200
190
180
170
160
150
140
130
120
110
100
90
80
70
60
50
40
30
20
10
0
f1 (ppm)

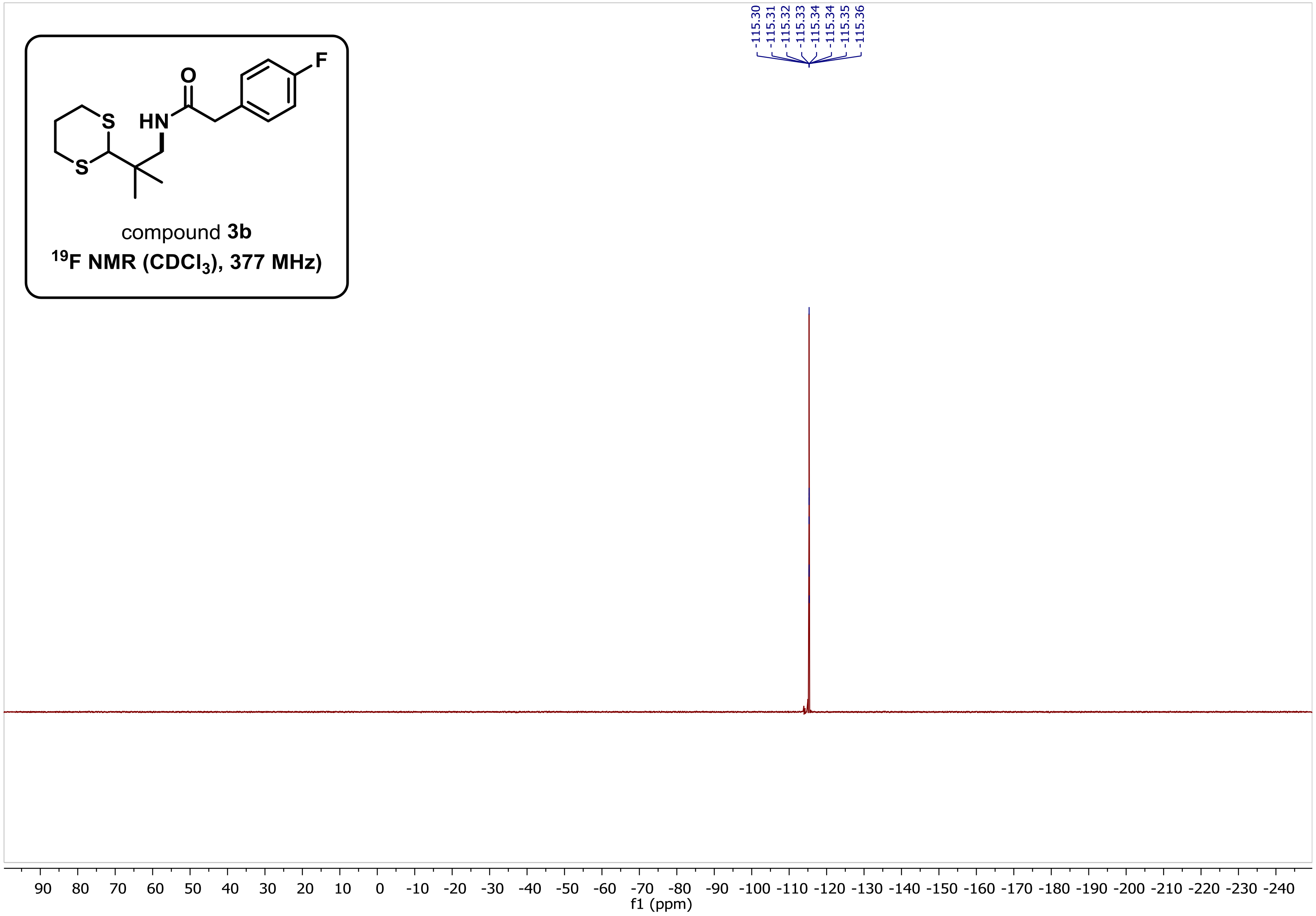

-115.30
-115.31
-115.32
-115.33
-115.34
-115.34
-115.35
-115.36
compound **3b**
^{19}F NMR (CDCl$_3$), 377 MHz)
90 80 70 60 50 40 30 20 10 0 -10 -20 -30 -40 -50 -60 -70 -80 -90 -100 -110 -120 -130 -140 -150 -160 -170 -180 -190 -200 -210 -220 -230 -240
f1 (ppm)

3.23. Compound 3c

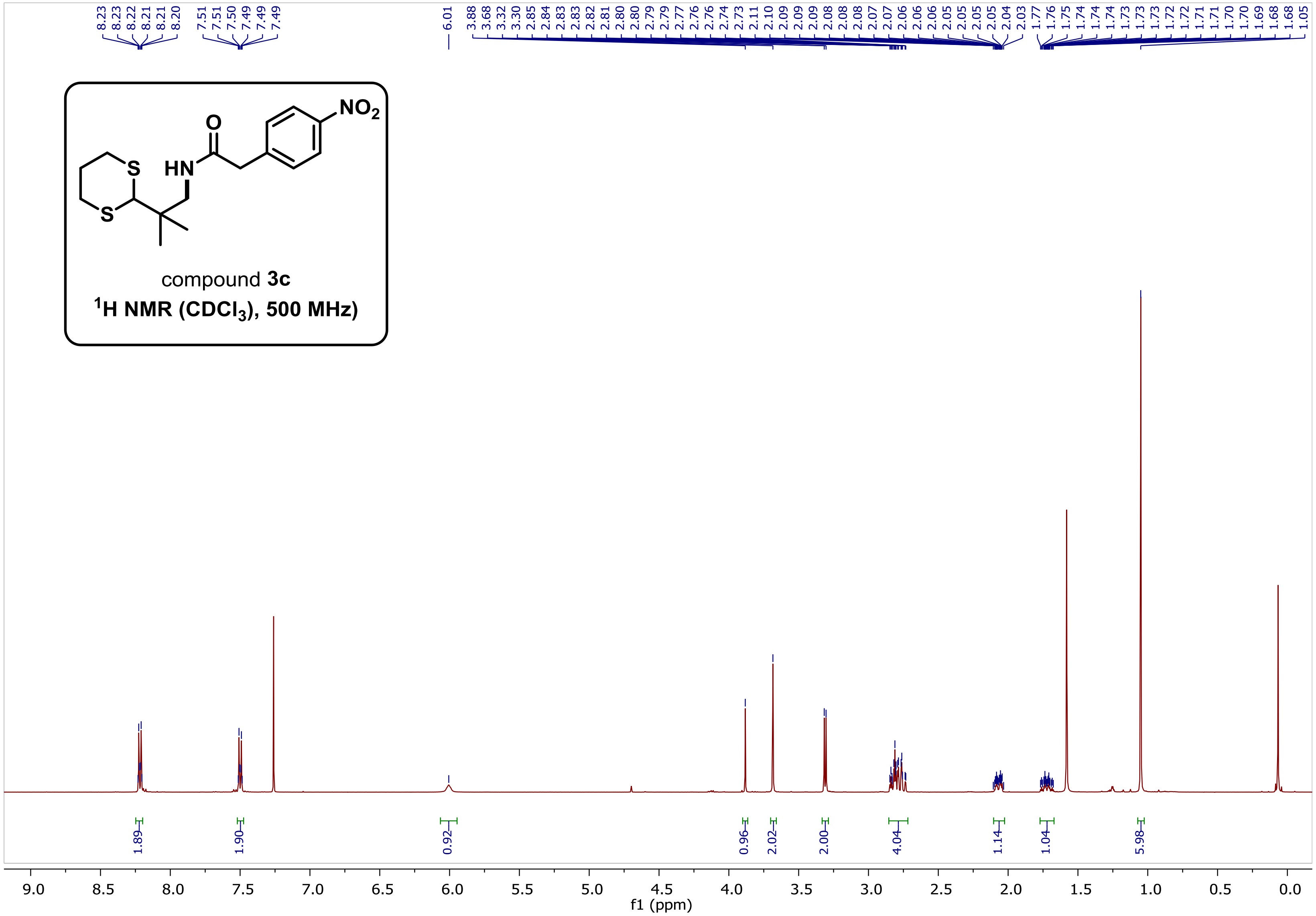

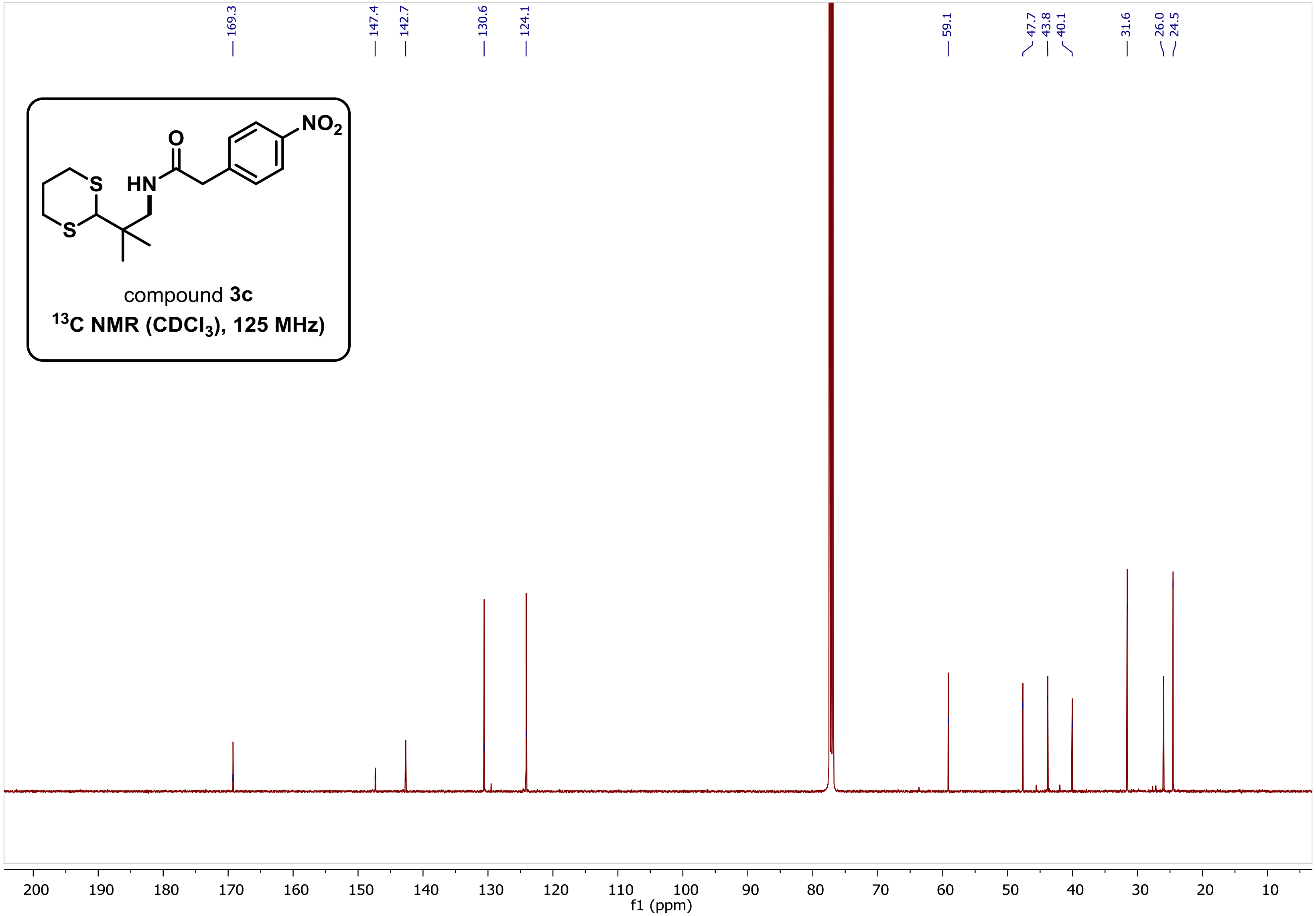
169.3
147.4
142.7
130.6
124.1
59.1
47.7
43.8
40.1
31.6
26.0
24.5
NO2
O
HN
S
S
compound 3c
13C NMR (CDCl3), 125 MHz)
200
190
180
170
160
150
140
130
120
110
100
90
80
70
60
50
40
30
20
10
f1 (ppm)

3.24. Compound 3d

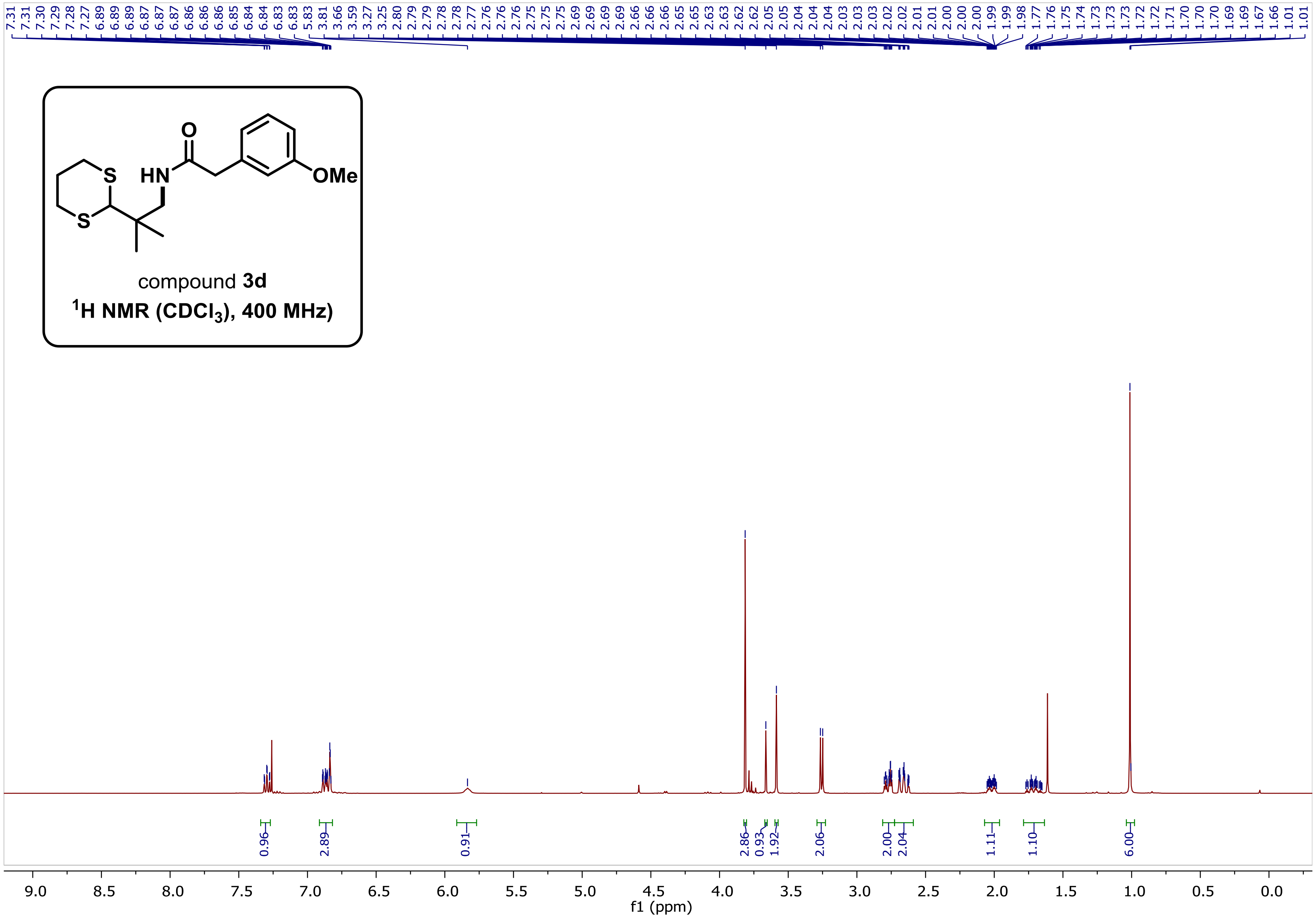

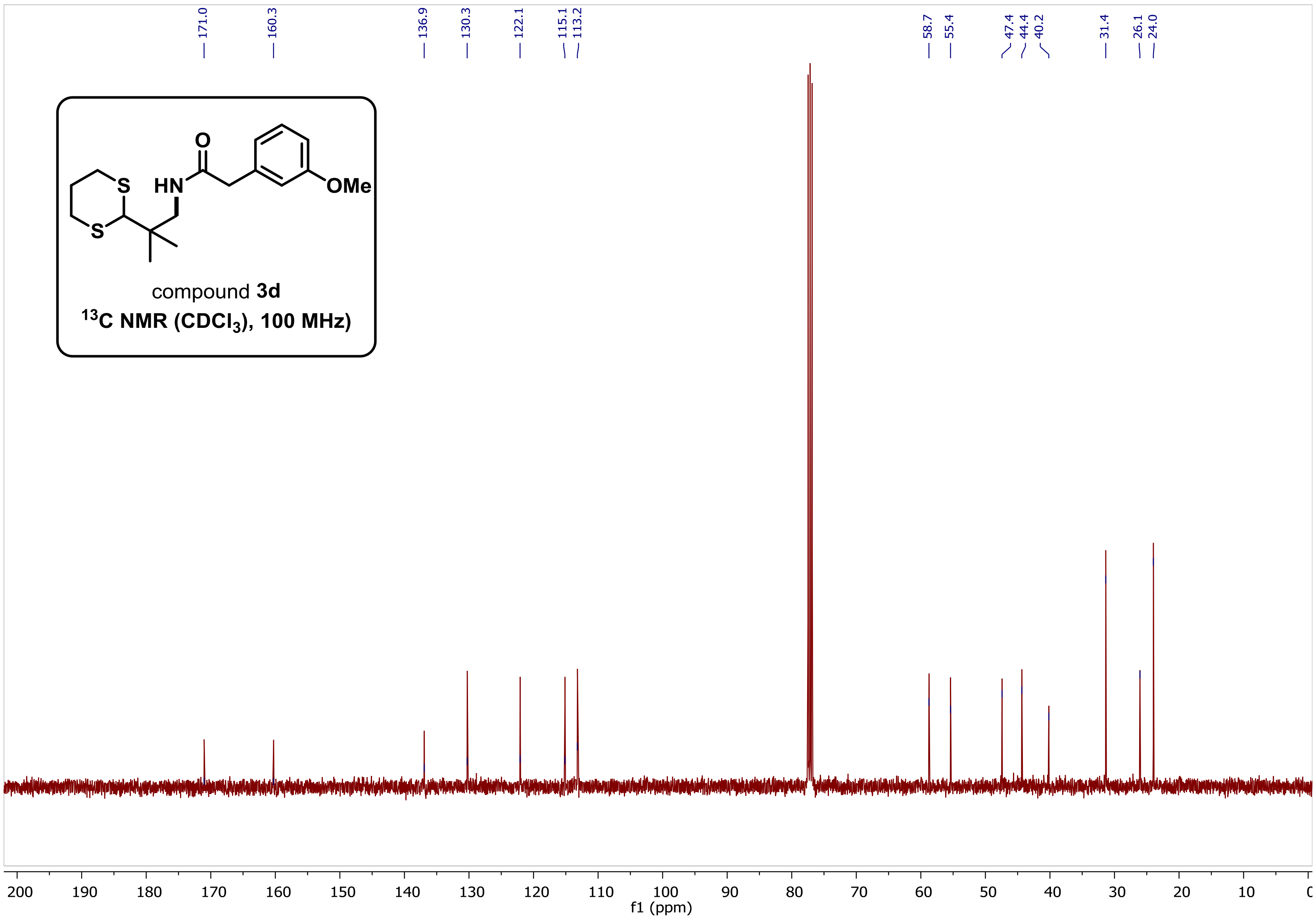
171.0
160.3
136.9
130.3
122.1
115.1
113.2
58.7
55.4
47.4
44.4
40.2
31.4
26.1
24.0
O
S
HN
OMe
S
compound **3d**
^{13}C NMR ($CDCl_3$), 100 MHz)
200 190 180 170 160 150 140 130 120 110 100 90 80 70 60 50 40 30 20 10 0
f1 (ppm)

3.25. Compound 3e

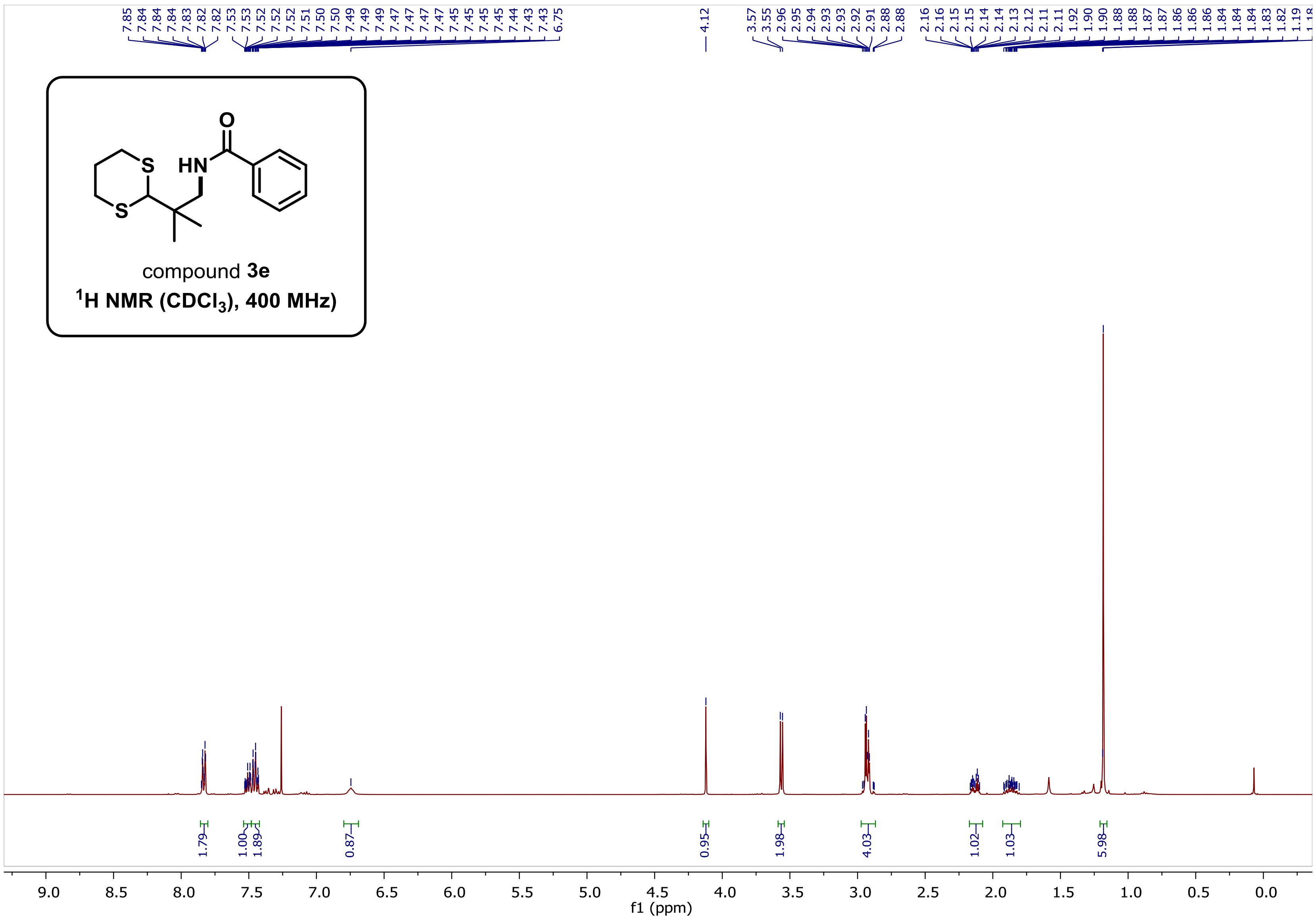

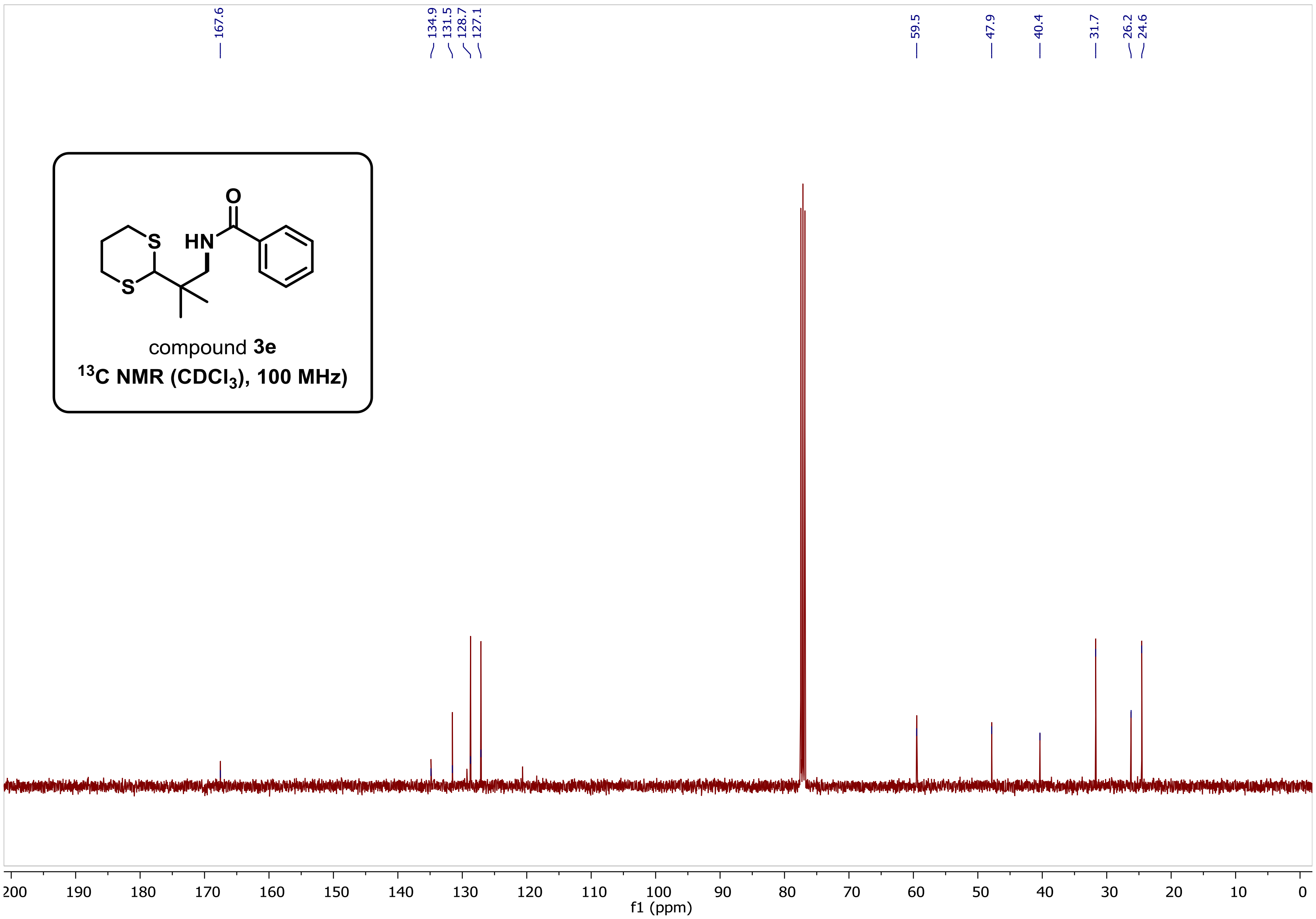

167.6
134.9
131.5
128.7
127.1
59.5
47.9
40.4
31.7
26.2
24.6
O
S
HN
S
compound 3e
13C NMR (CDCl3), 100 MHz)
200 190 180 170 160 150 140 130 120 110 100 90 80 70 60 50 40 30 20 10 0
f1 (ppm)

3.26. Compound 3f

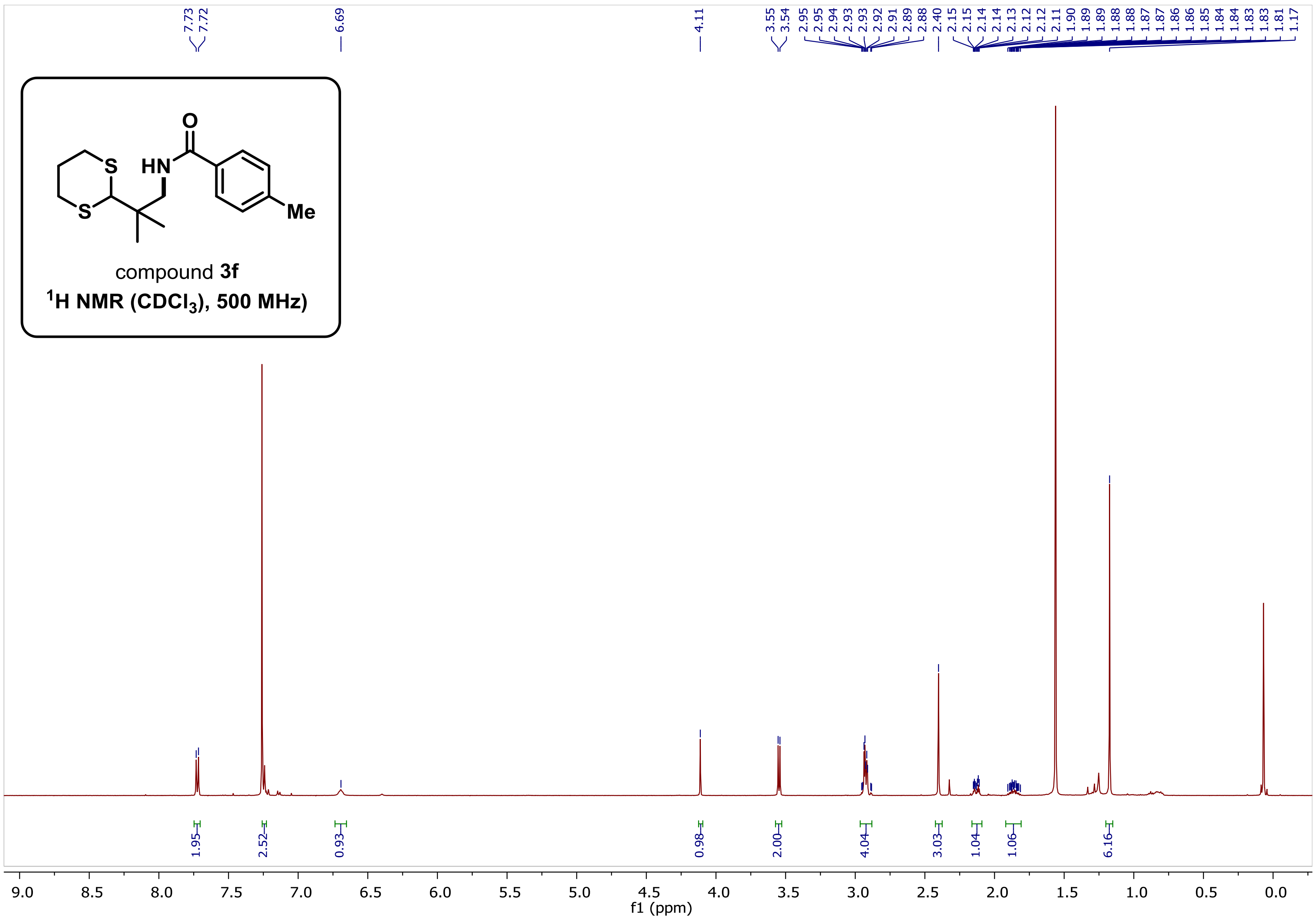

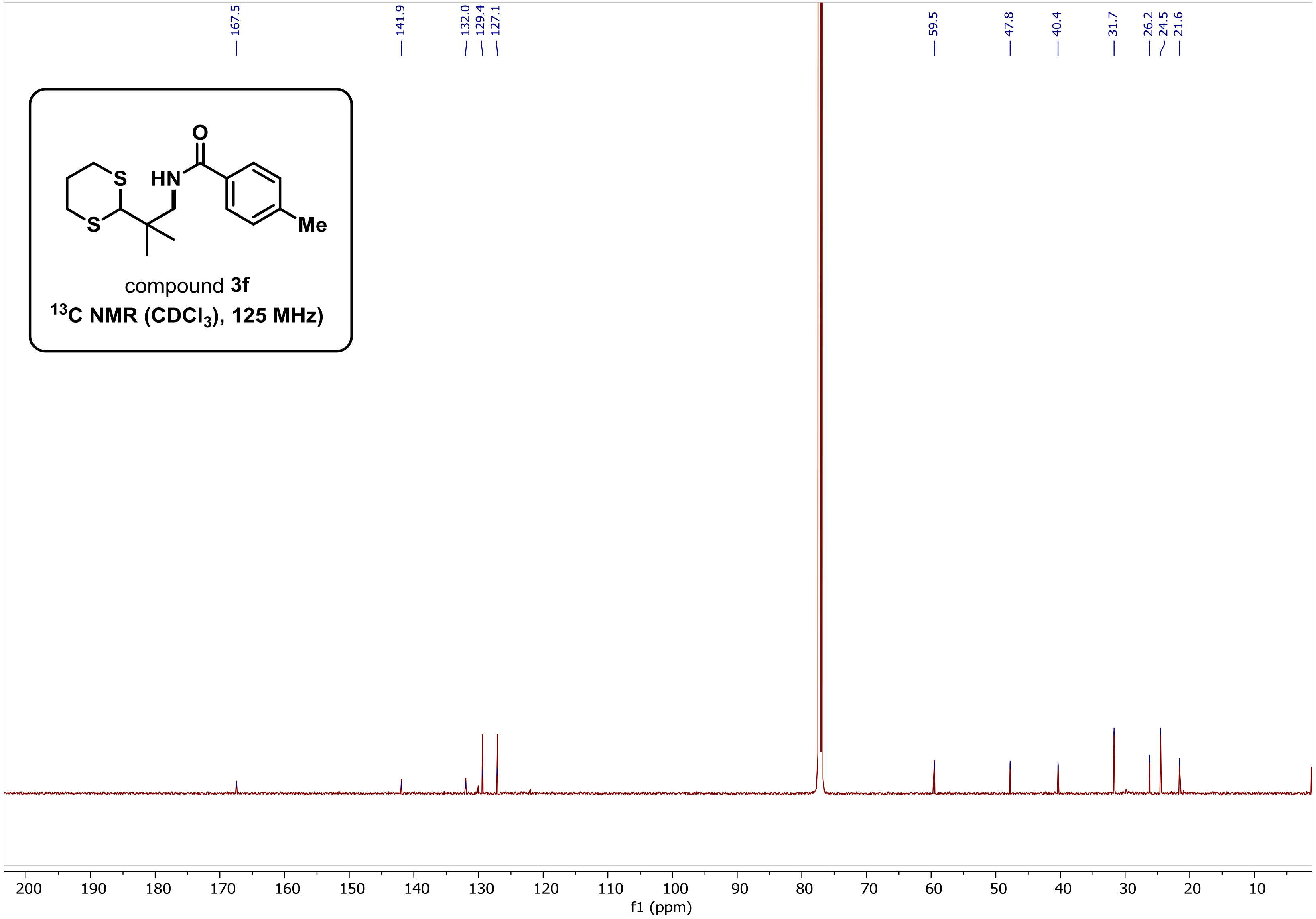

167.5
141.9
132.0
129.4
127.1
59.5
47.8
40.4
31.7
26.2
24.5
21.6
O
S
HN
S
Me
compound 3f
13C NMR (CDCl3), 125 MHz)
200 190 180 170 160 150 140 130 120 110 100 90 80 70 60 50 40 30 20 10
f1 (ppm)

3.27. Compound 3g

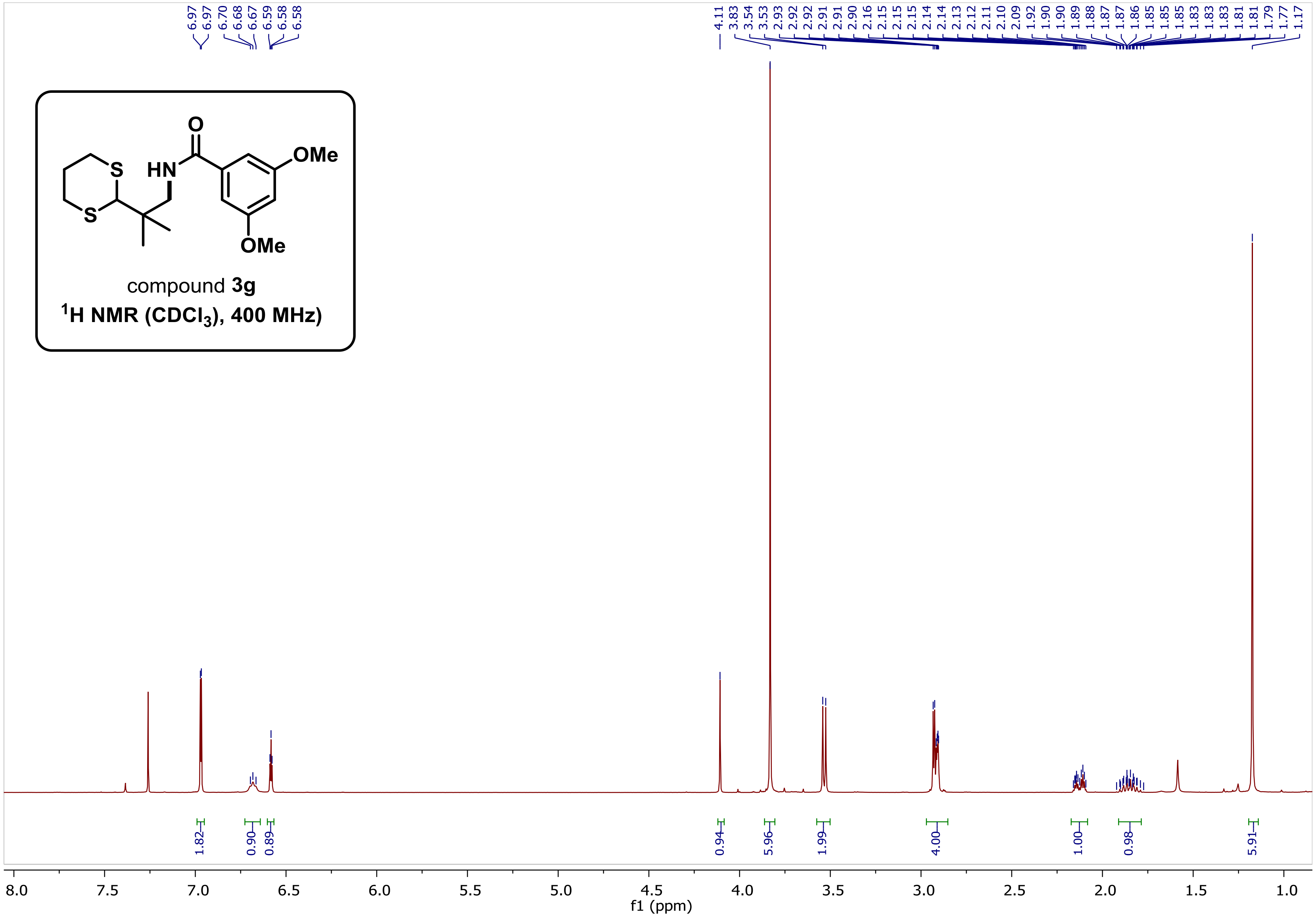

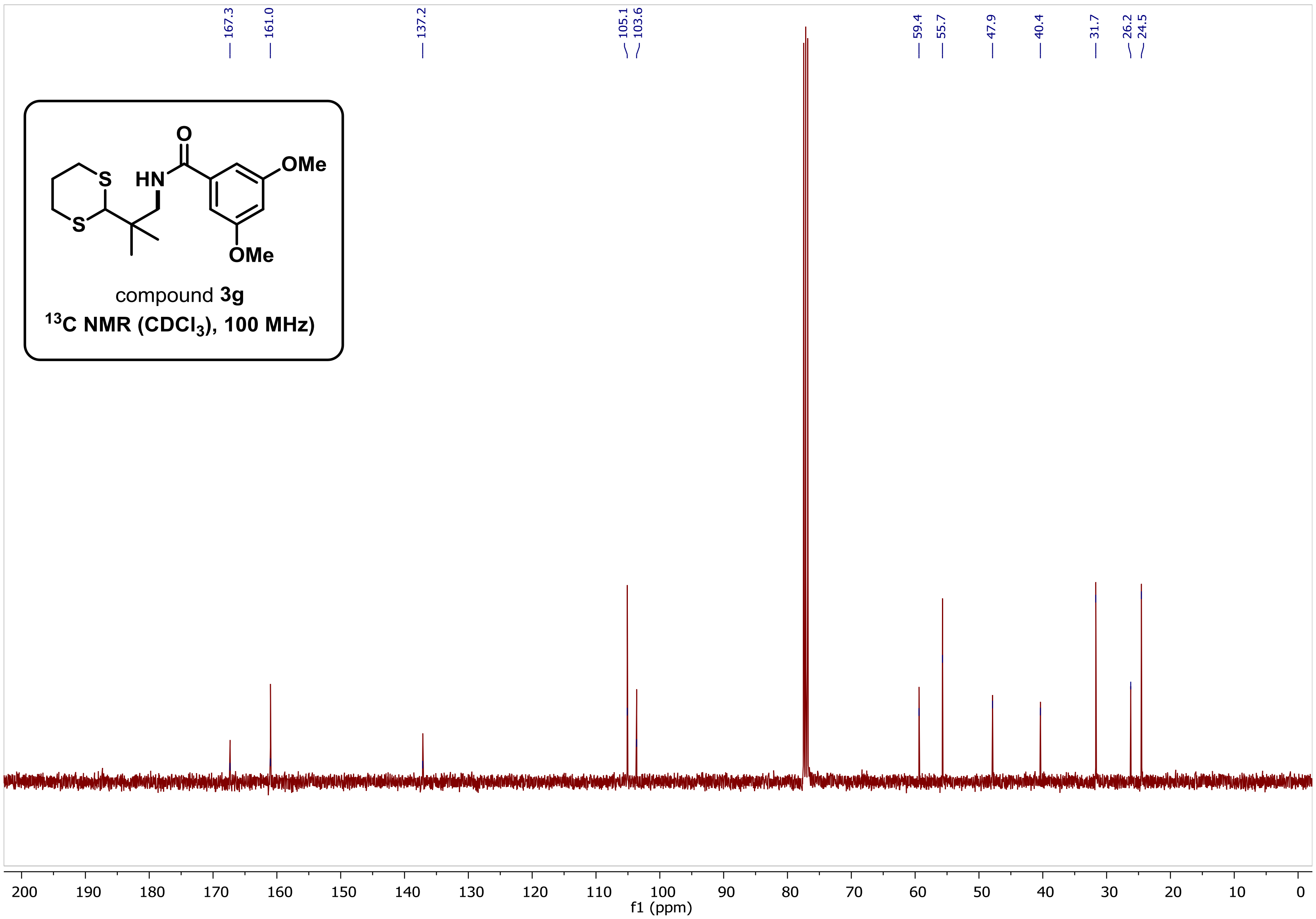

167.3
161.0
137.2
105.1
103.6
59.4
55.7
47.9
40.4
31.7
26.2
24.5
O
HN
S
S
OMe
OMe
compound 3g
13C NMR (CDCl3), 100 MHz)
200
190
180
170
160
150
140
130
120
110
100
90
80
70
60
50
40
30
20
10
0
f1 (ppm)

3.28. **Compound 3h**

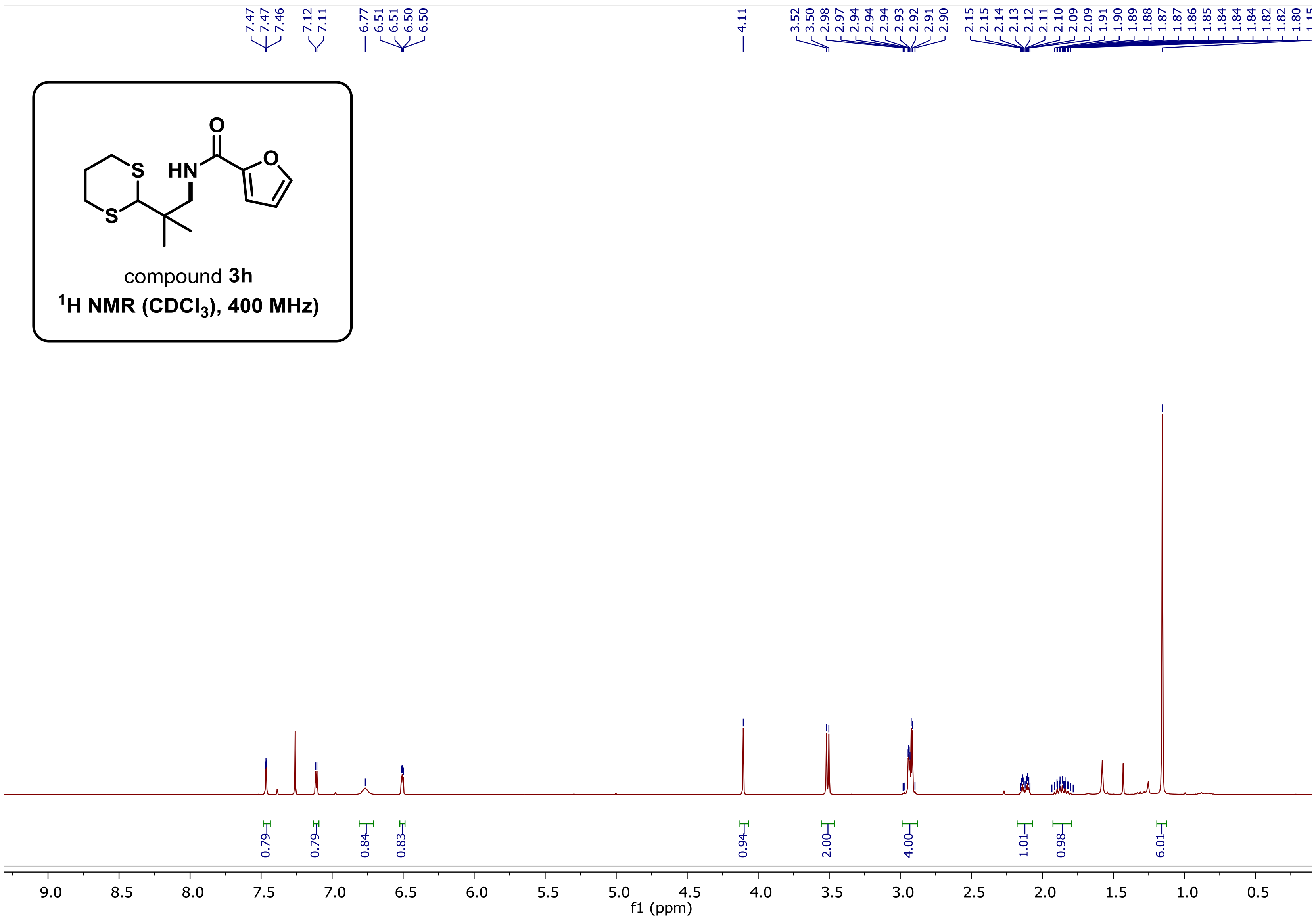

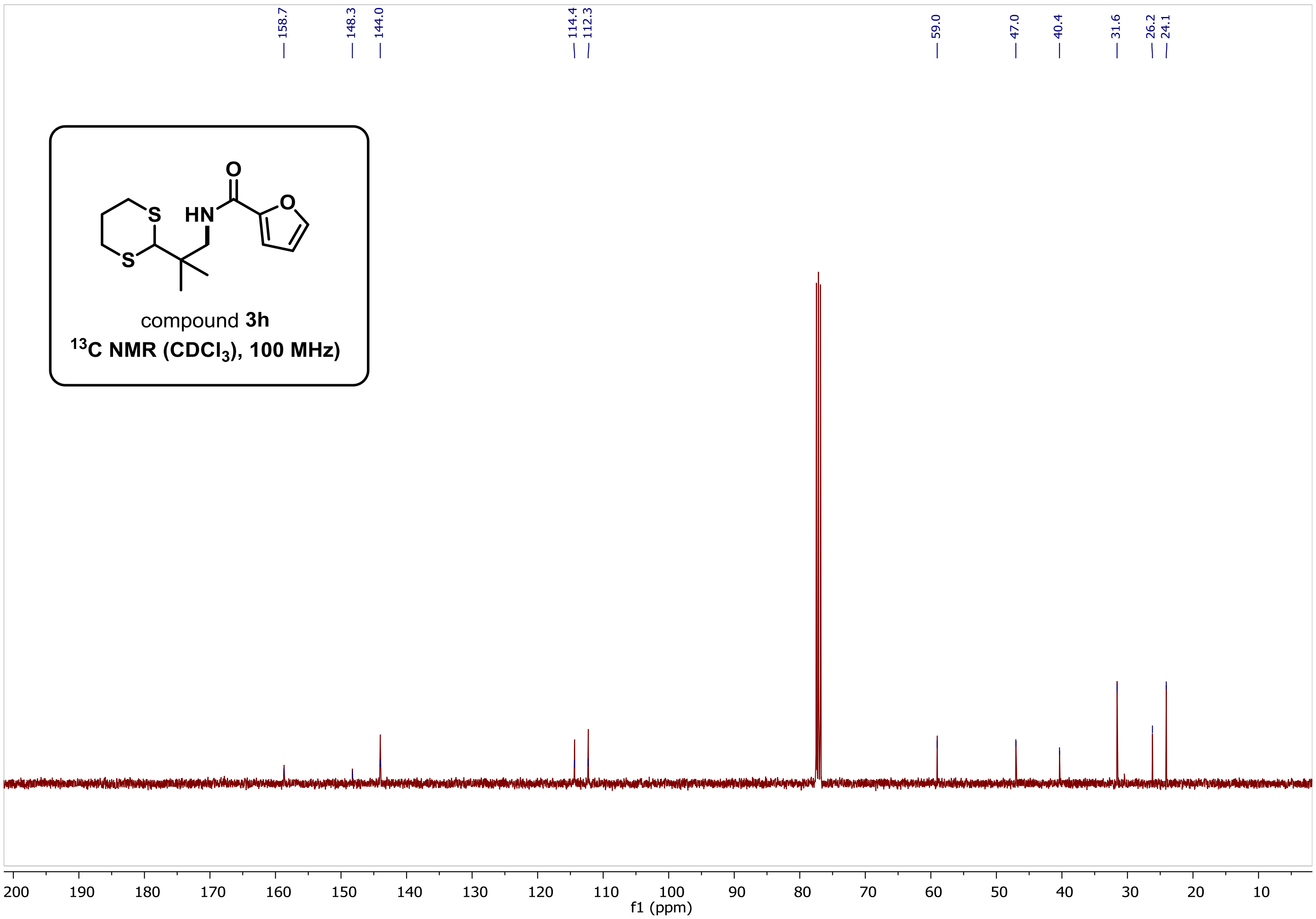
158.7
148.3
144.0
114.4
112.3
59.0
47.0
40.4
31.6
26.2
24.1
compound **3h**
^{13}C NMR ($CDCl_3$), 100 MHz)
f1 (ppm)

3.29. Compound 3i

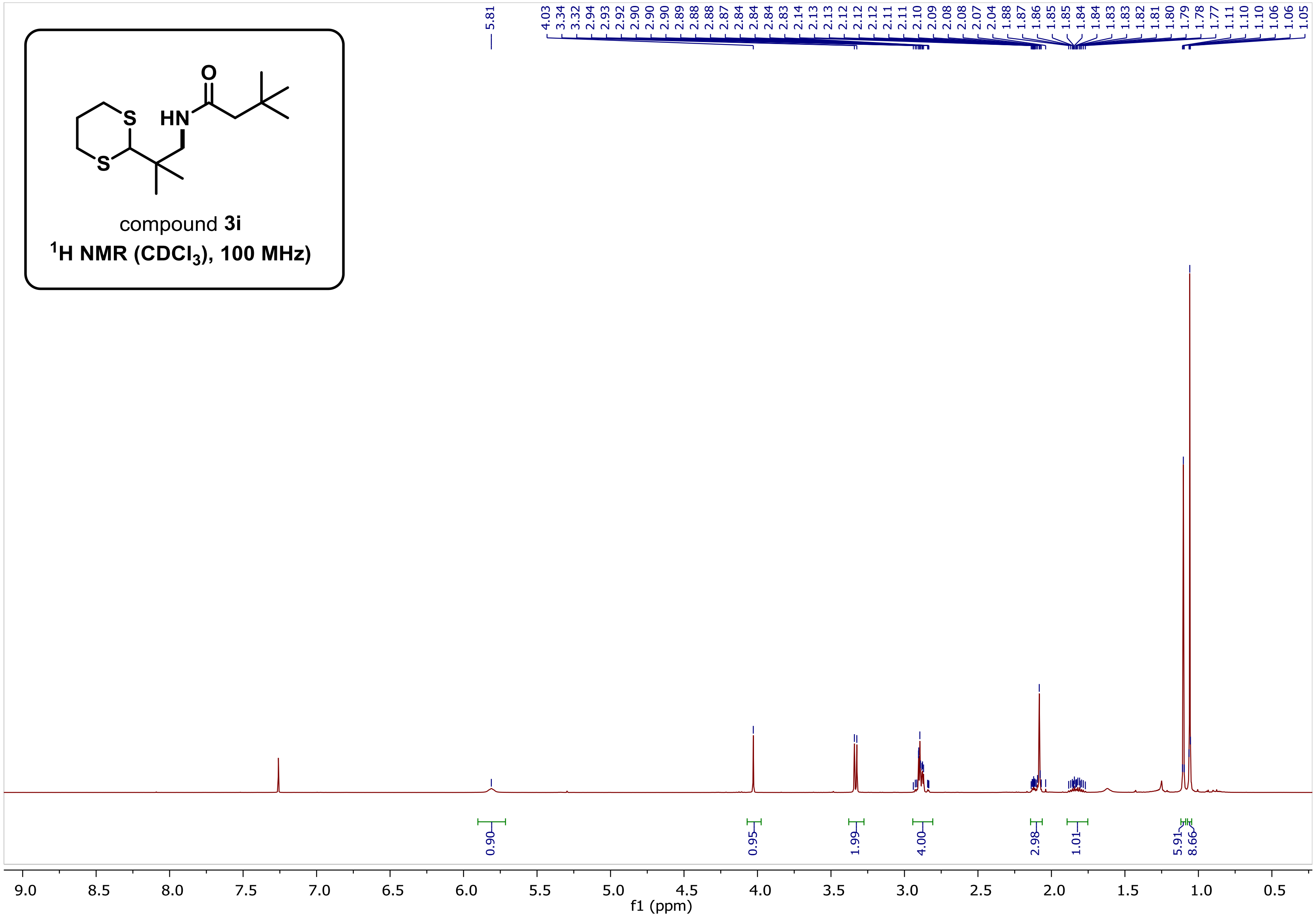

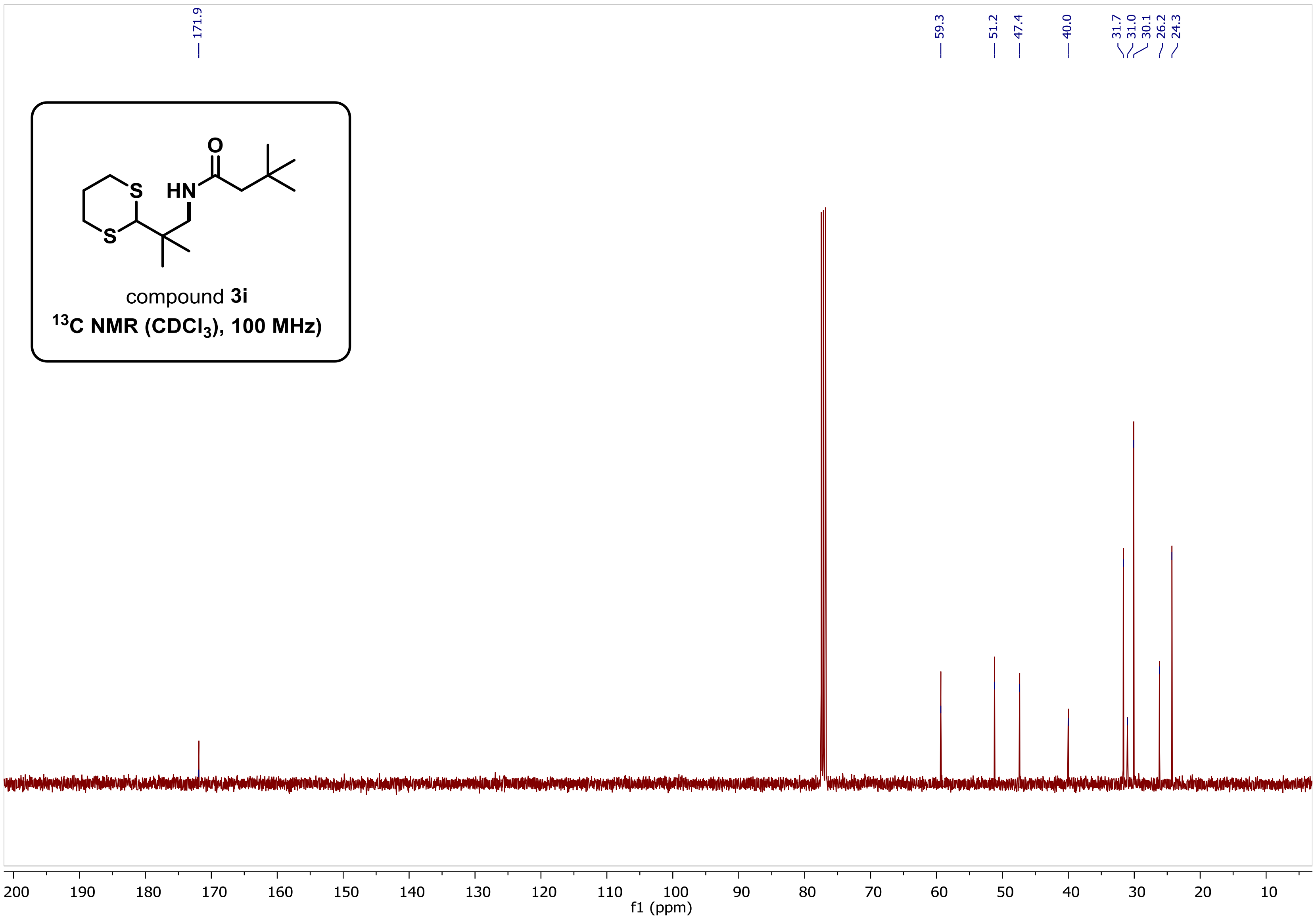

compound **3i**
^{13}C NMR ($CDCl_3$), 100 MHz
171.9
59.3
51.2
47.4
40.0
31.7
31.0
30.1
26.2
24.3
200
190
180
170
160
150
140
130
120
110
100
90
80
70
60
50
40
30
20
10
f1 (ppm)

3.30. Compound 3j

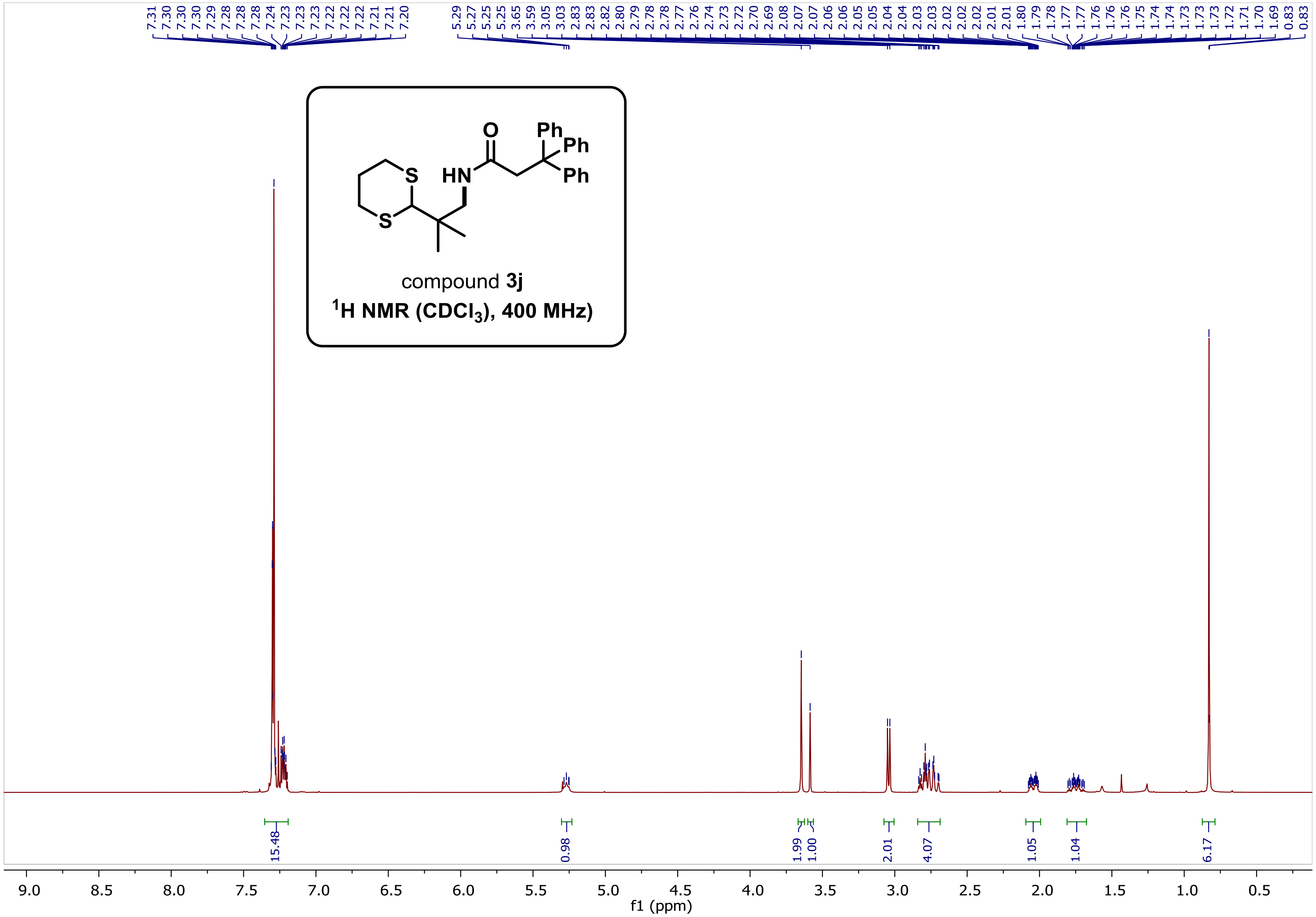

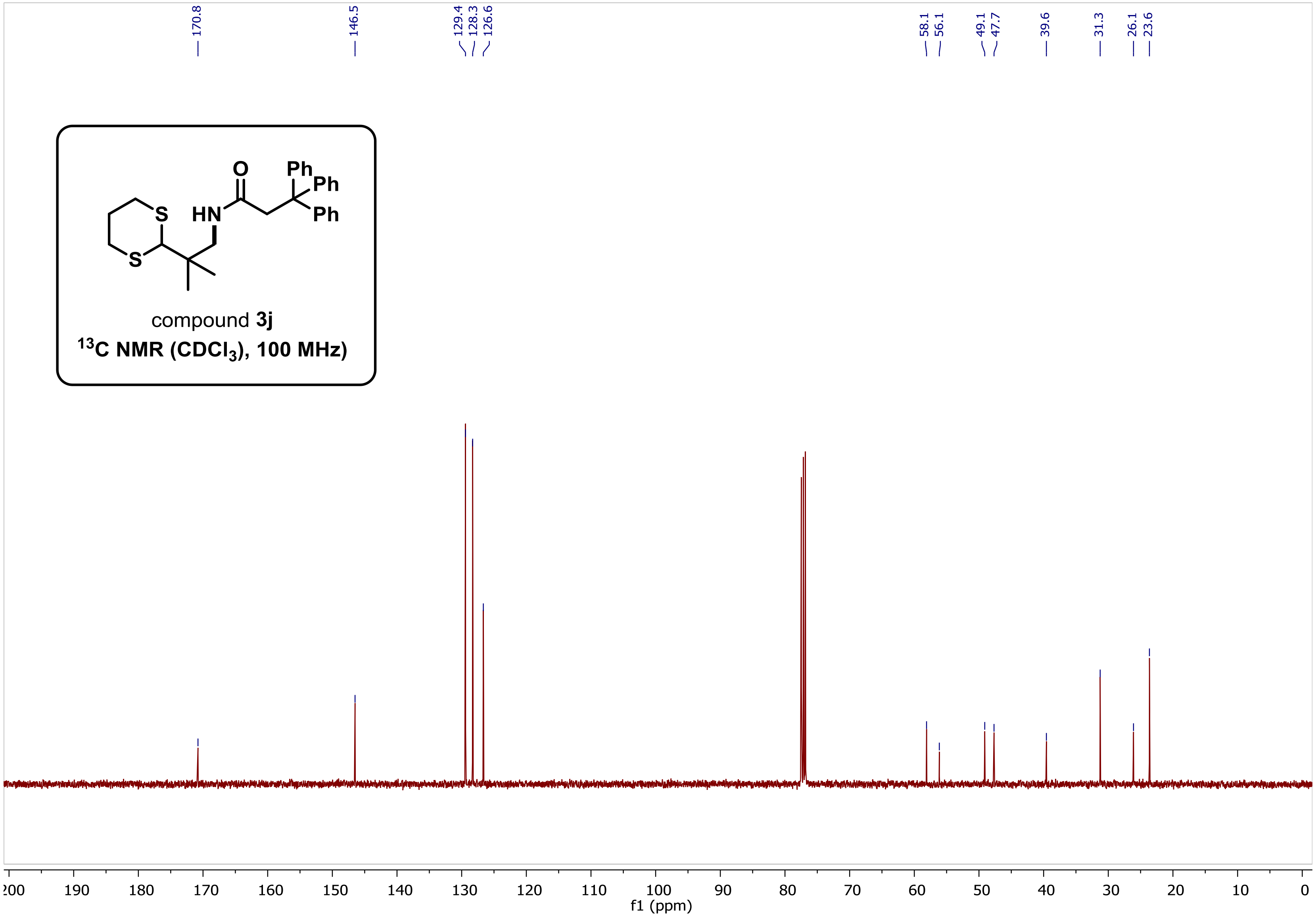

170.8
146.5
129.4
128.3
126.6
58.1
56.1
49.1
47.7
39.6
31.3
26.1
23.6
O
Ph
Ph
Ph
S
HN
S
compound 3j
13C NMR (CDCl3), 100 MHz)
200 190 180 170 160 150 140 130 120 110 100 90 80 70 60 50 40 30 20 10 0
f1 (ppm)

3.31. Compound 3k

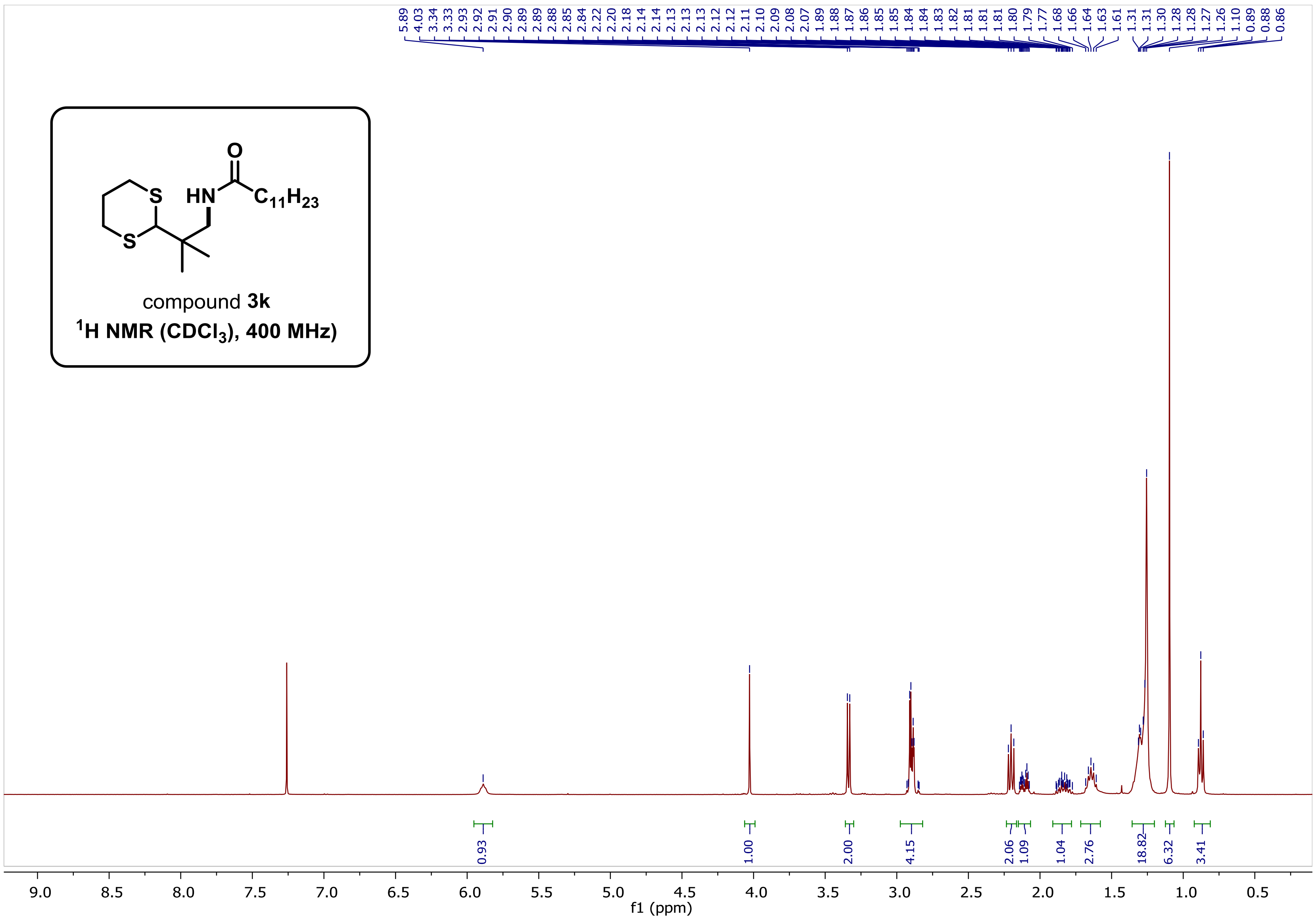

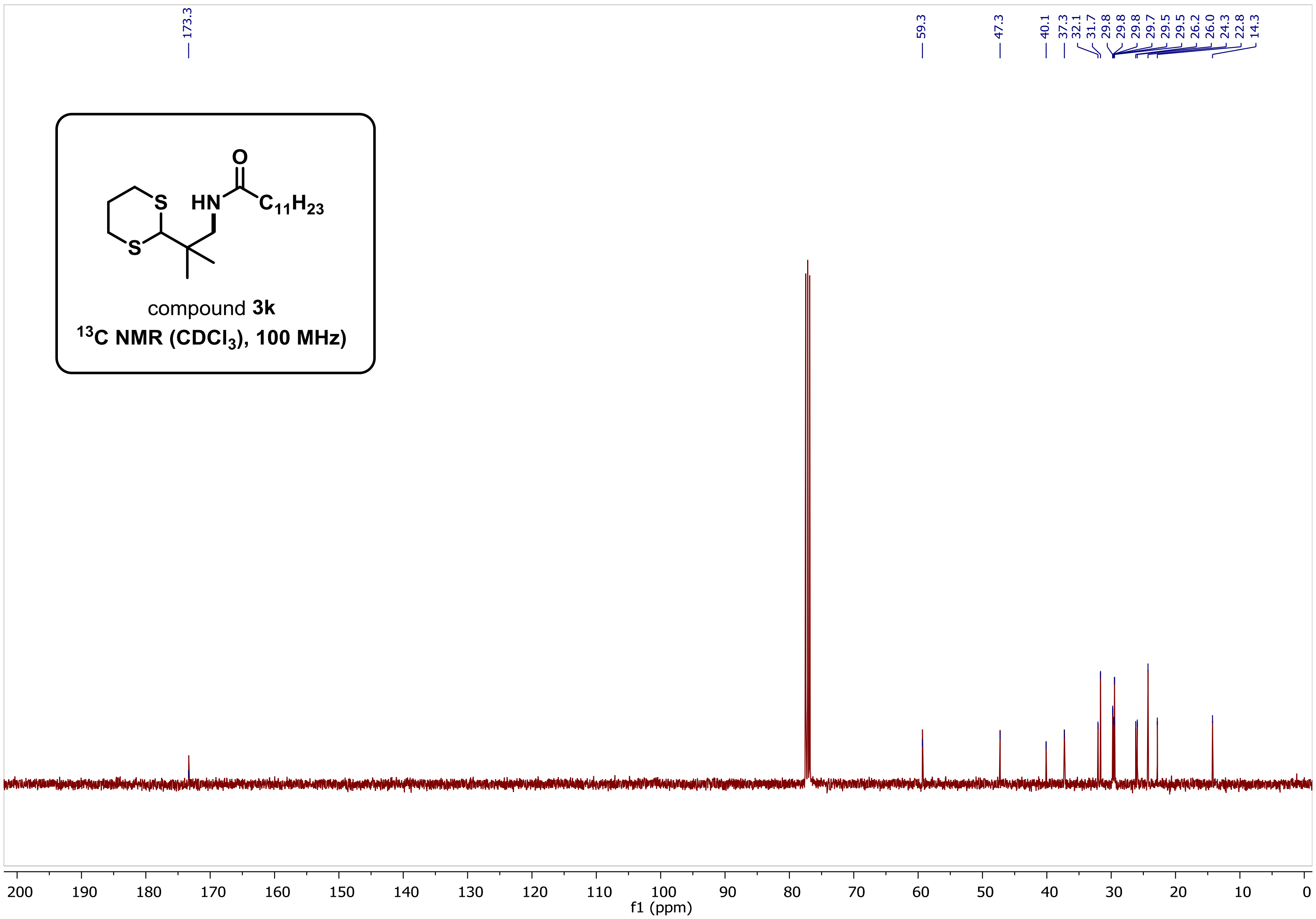
173.3
59.3
47.3
40.1
37.3
32.1
31.7
29.8
29.8
29.8
29.7
29.5
29.5
26.2
26.0
24.3
22.8
14.3
O
S
HN
$C_{11}H_{23}$
S
compound **3k**
^{13}C NMR ($CDCl_3$), 100 MHz
200 190 180 170 160 150 140 130 120 110 100 90 80 70 60 50 40 30 20 10 0
f1 (ppm)

3.32. Compound 3l

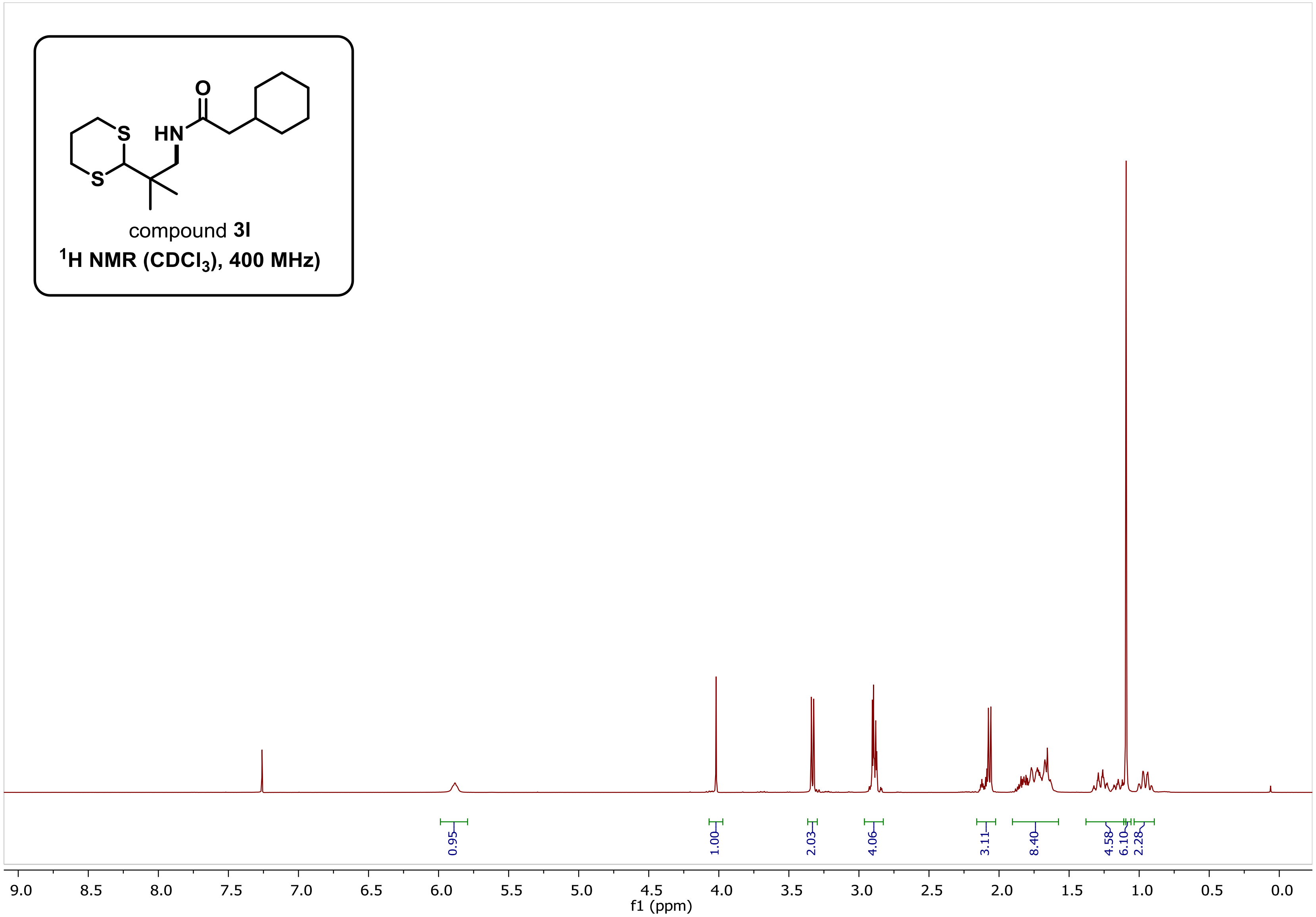

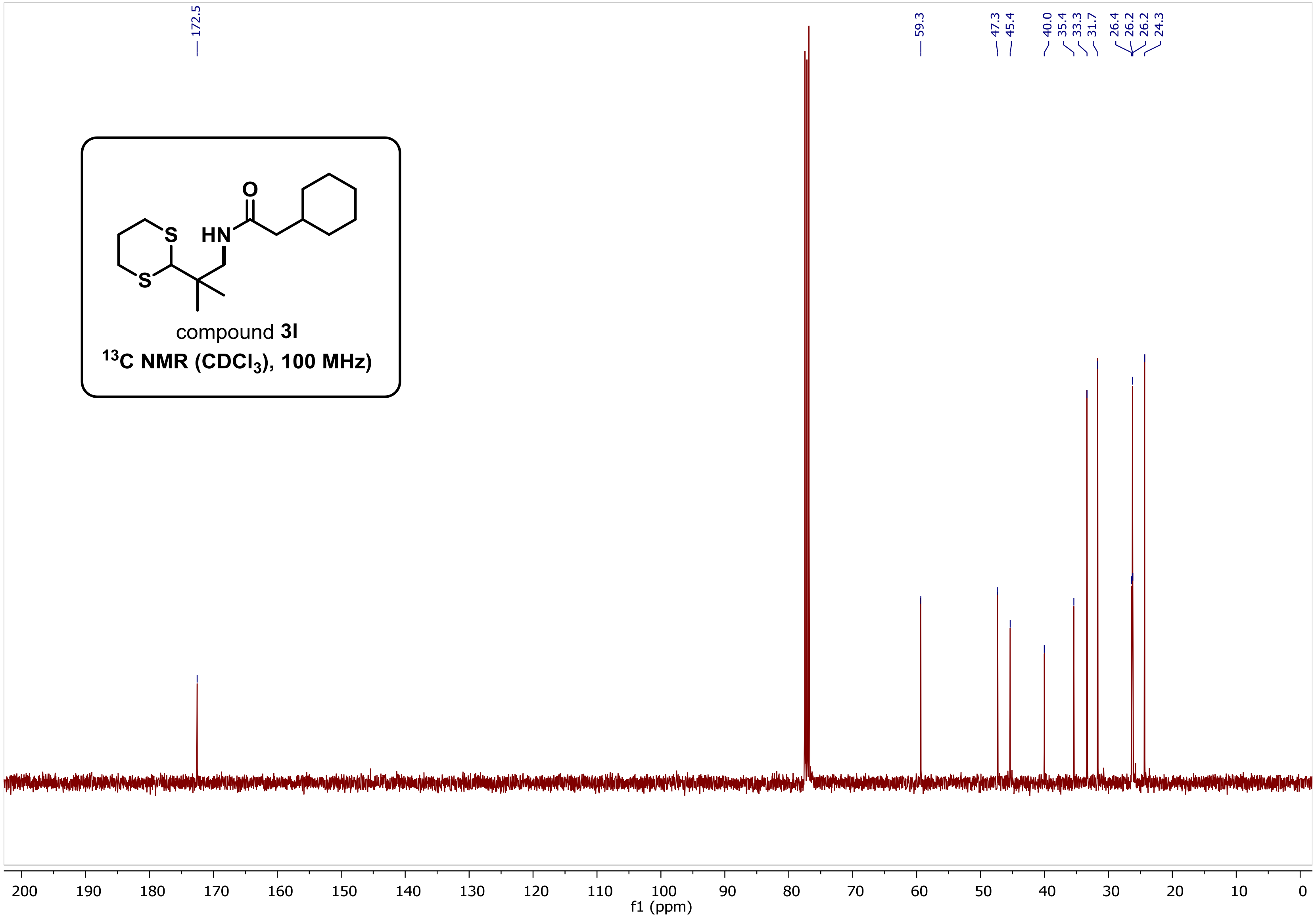
172.5
59.3
47.3
45.4
40.0
35.4
33.3
31.7
26.4
26.2
26.2
24.3
compound 3l
^{13}C NMR ($CDCl_3$), 100 MHz)
200
190
180
170
160
150
140
130
120
110
100
90
80
70
60
50
40
30
20
10
0
f1 (ppm)

3.33. Compound 3m

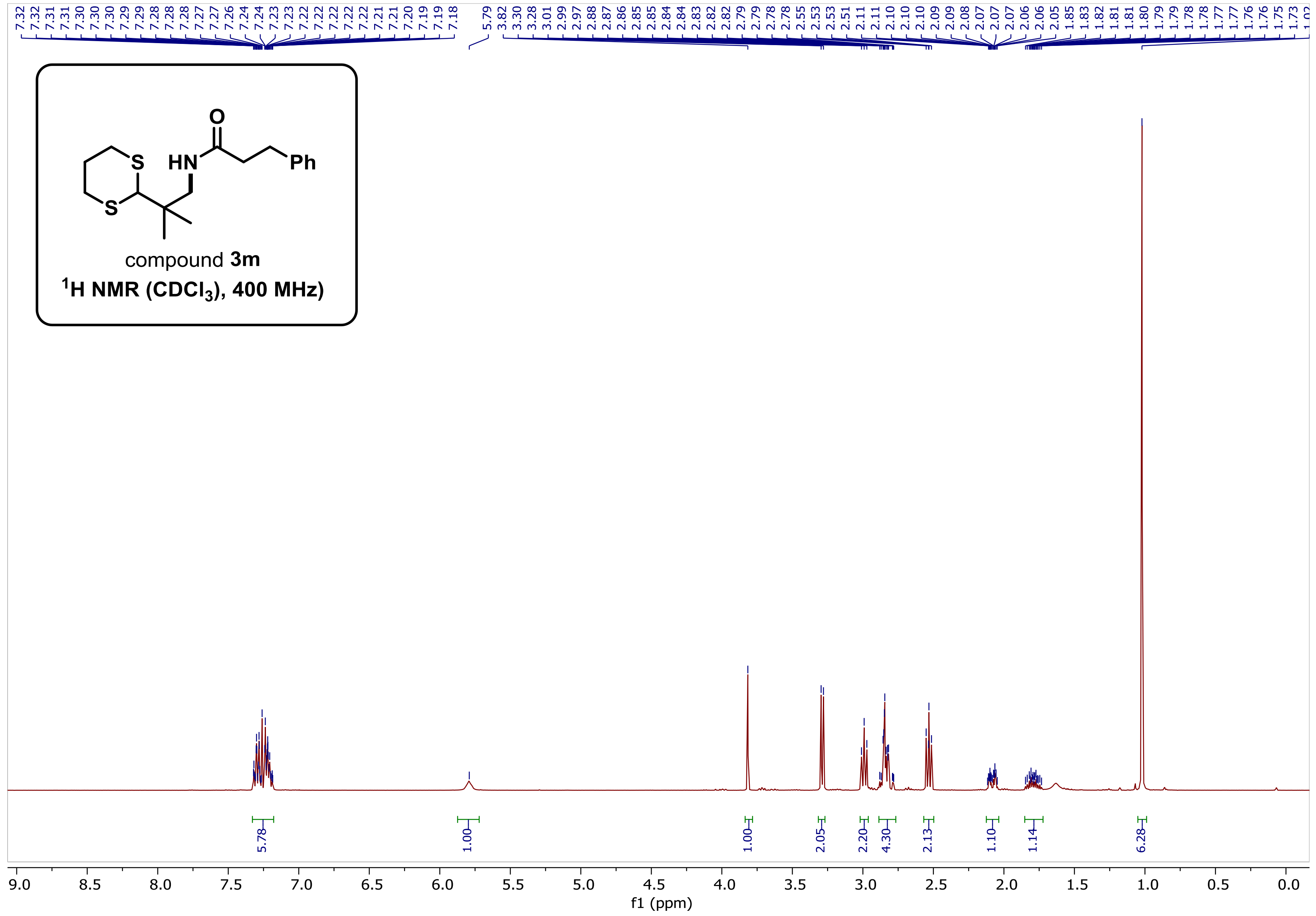

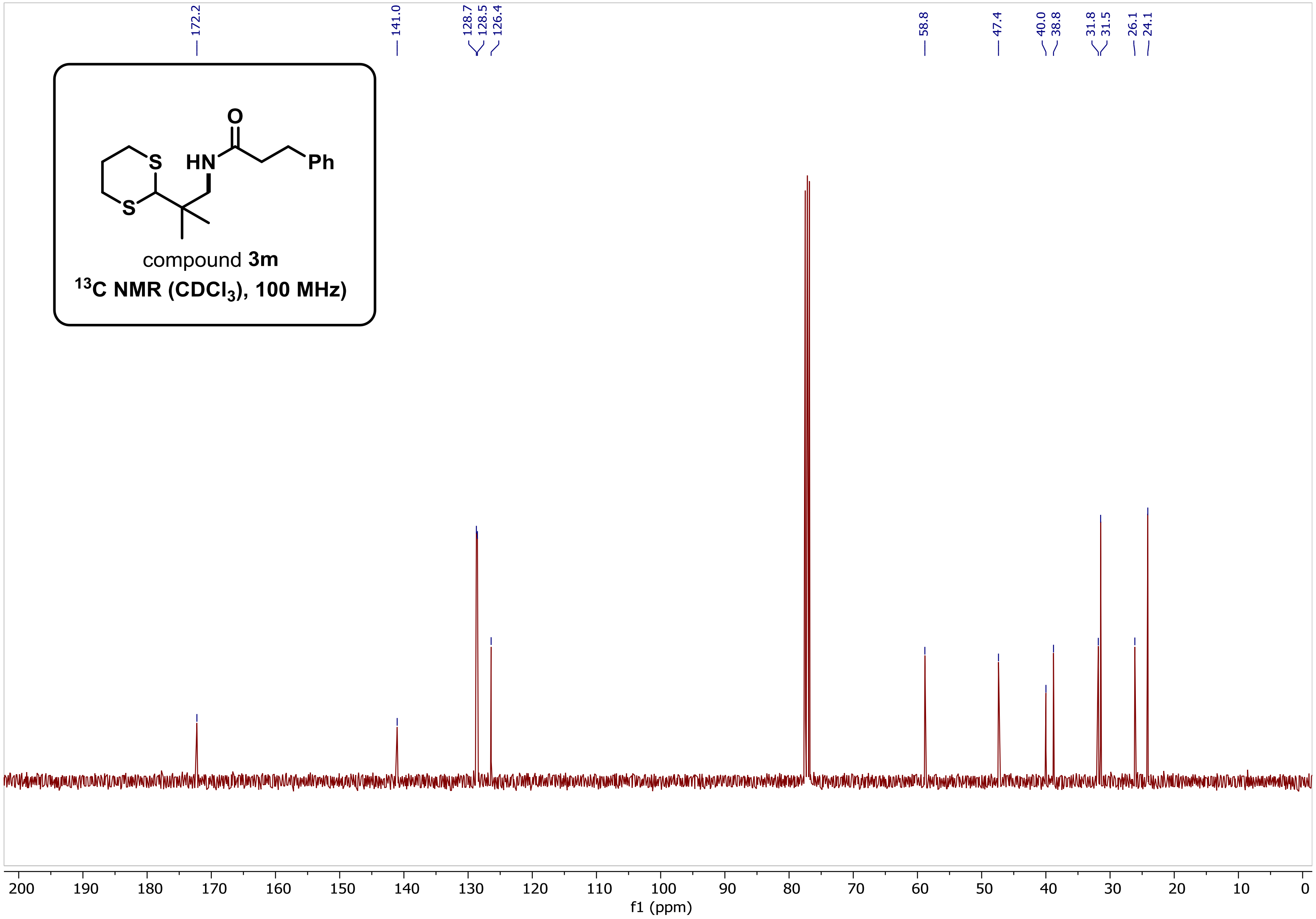

172.2
141.0
128.7
128.5
126.4
58.8
47.4
40.0
38.8
31.8
31.5
26.1
24.1
O
HN
S
S
Ph
compound 3m
^{13}C NMR ($CDCl_3$), 100 MHz)
200 190 180 170 160 150 140 130 120 110 100 90 80 70 60 50 40 30 20 10 0
f1 (ppm)

3.34. Compound 3n

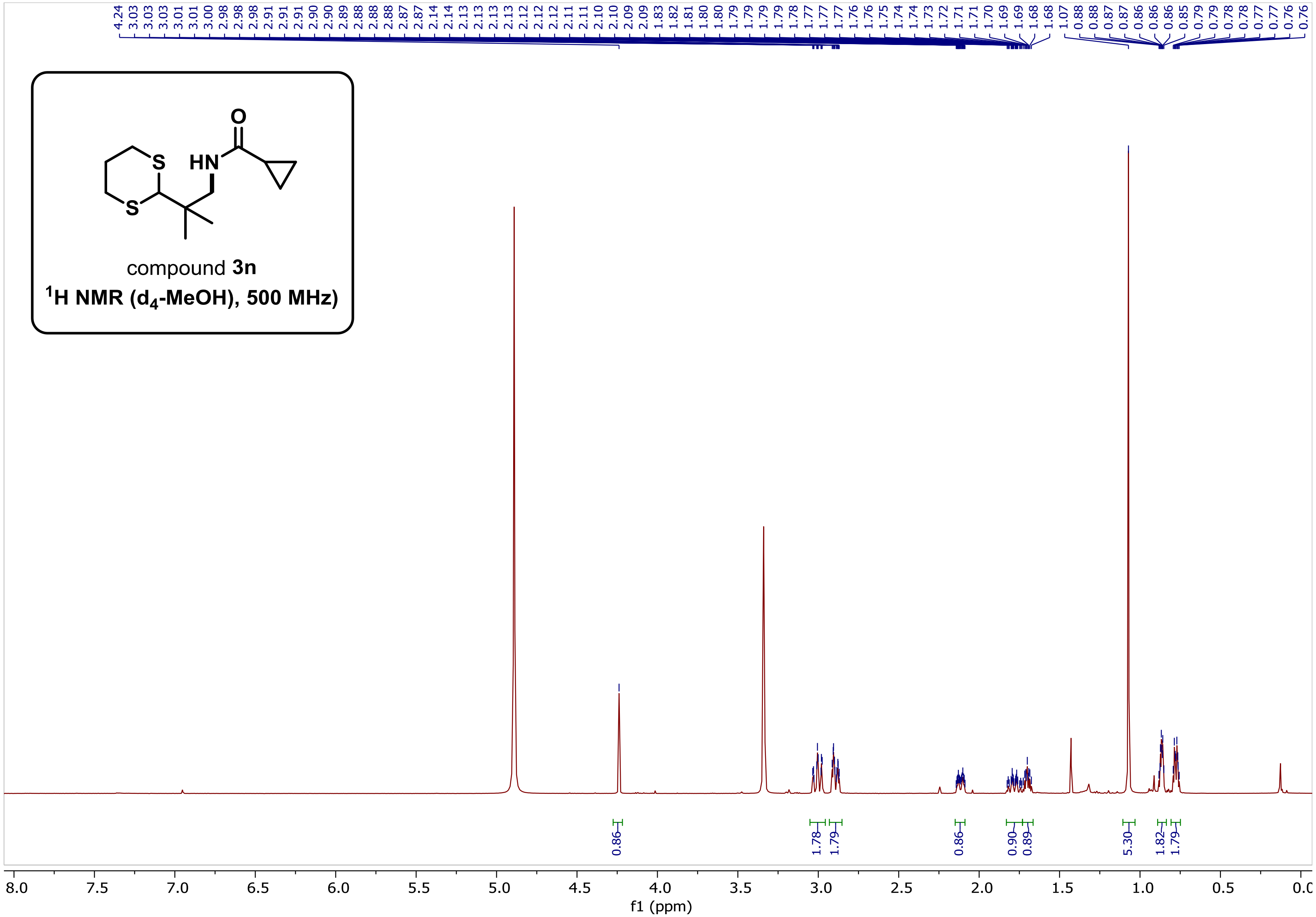

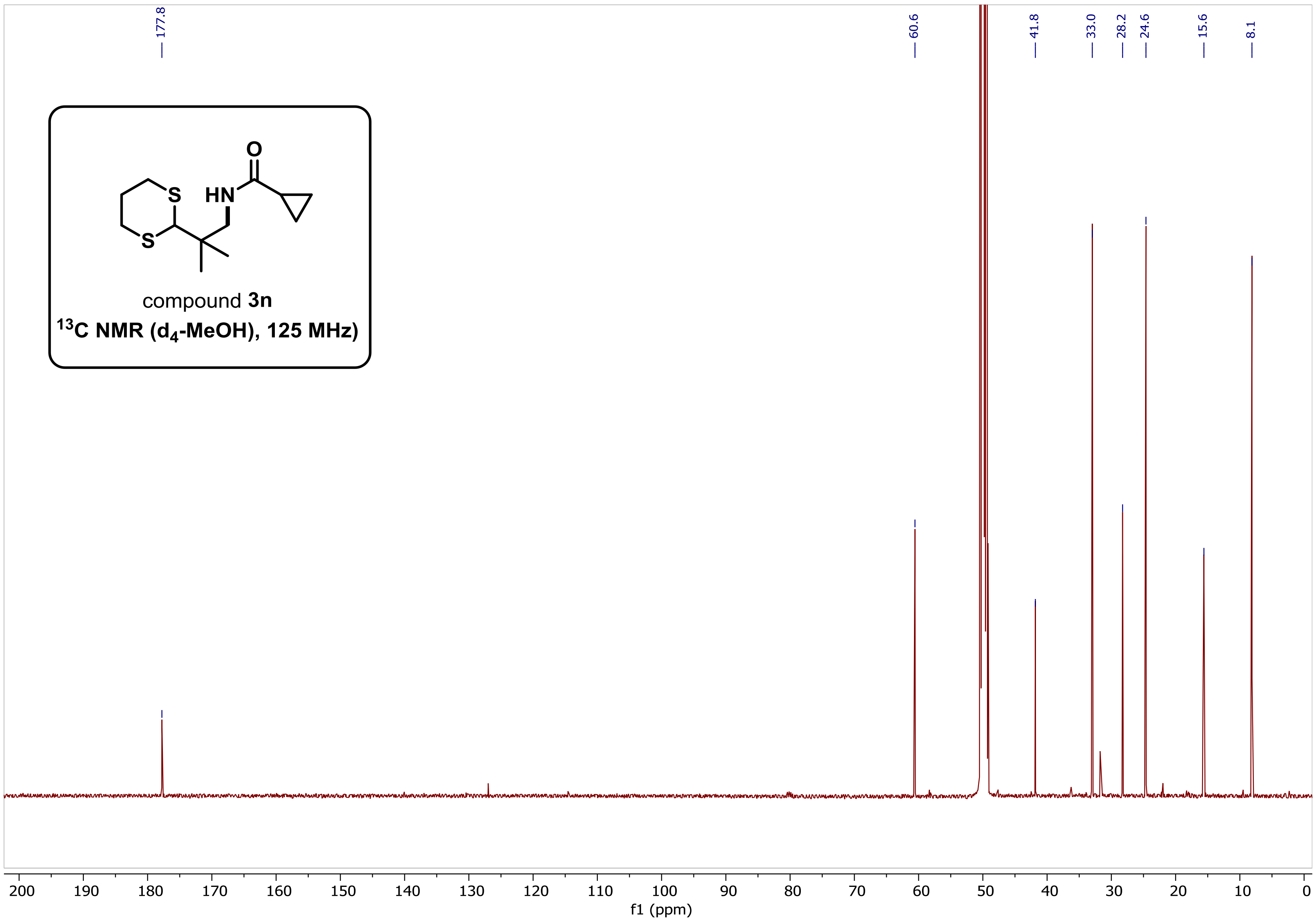

177.8
60.6
41.8
33.0
28.2
24.6
15.6
8.1
S
S
HN
O
compound 3n
13C NMR (d4-MeOH), 125 MHz)
200 190 180 170 160 150 140 130 120 110 100 90 80 70 60 50 40 30 20 10 0
f1 (ppm)

3.35. Compound 3o

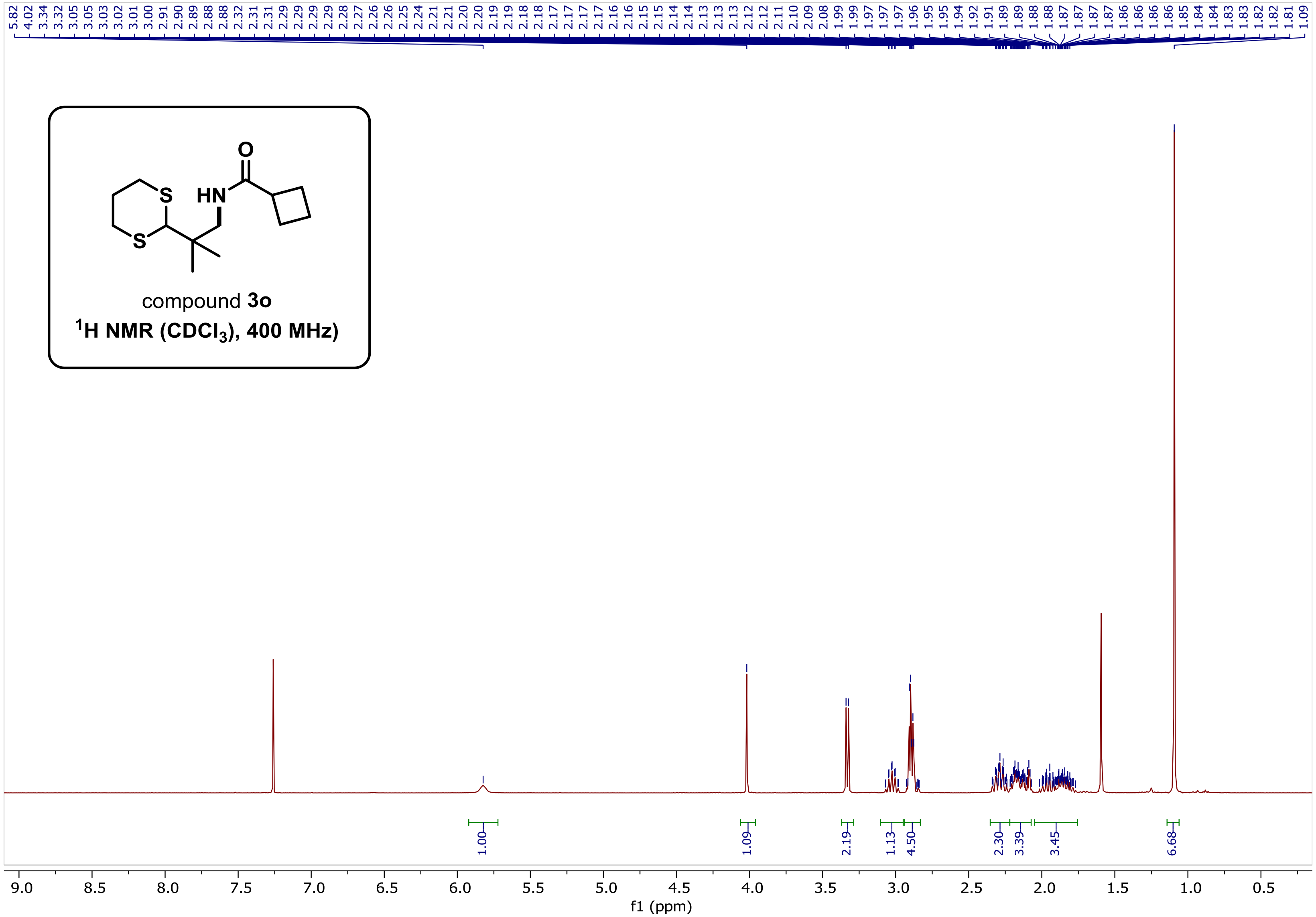

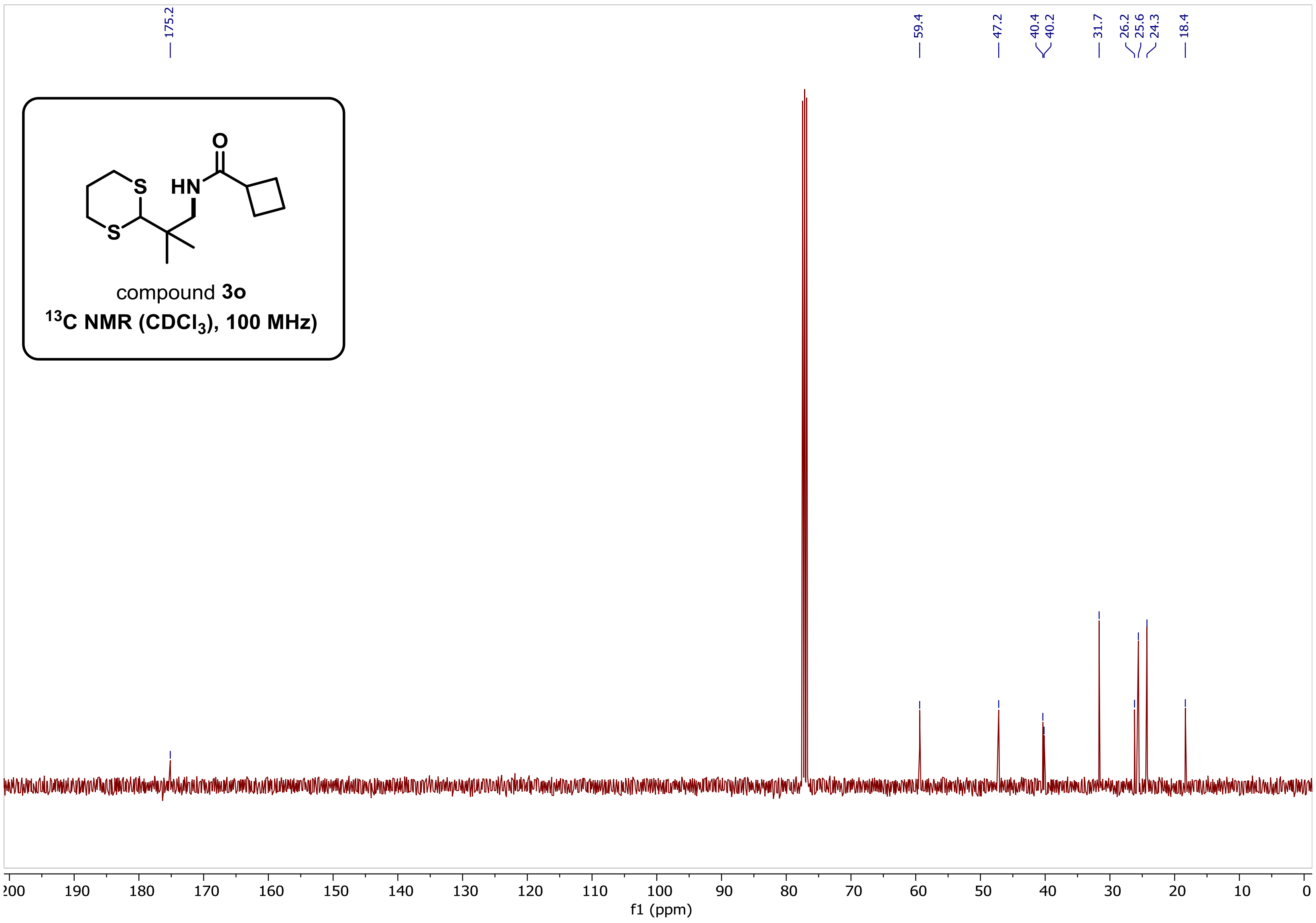
175.2
59.4
47.2
40.4
40.2
31.7
26.2
25.6
24.3
18.4
HN
O
S
S
compound 3o
13C NMR (CDCl3), 100 MHz)
200
190
180
170
160
150
140
130
120
110
100
90
80
70
60
50
40
30
20
10
0
f1 (ppm)

3.36. Compound 3p

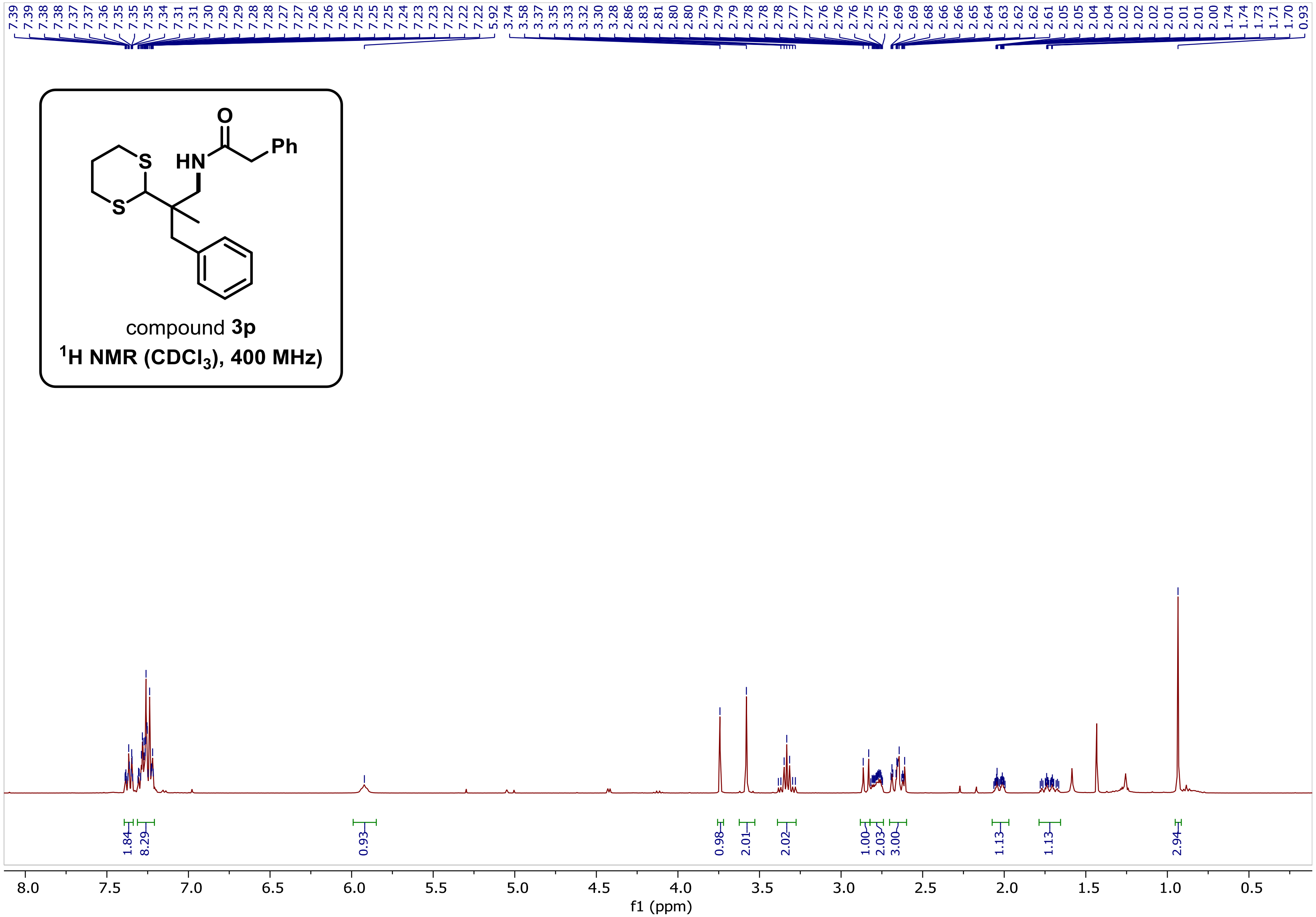

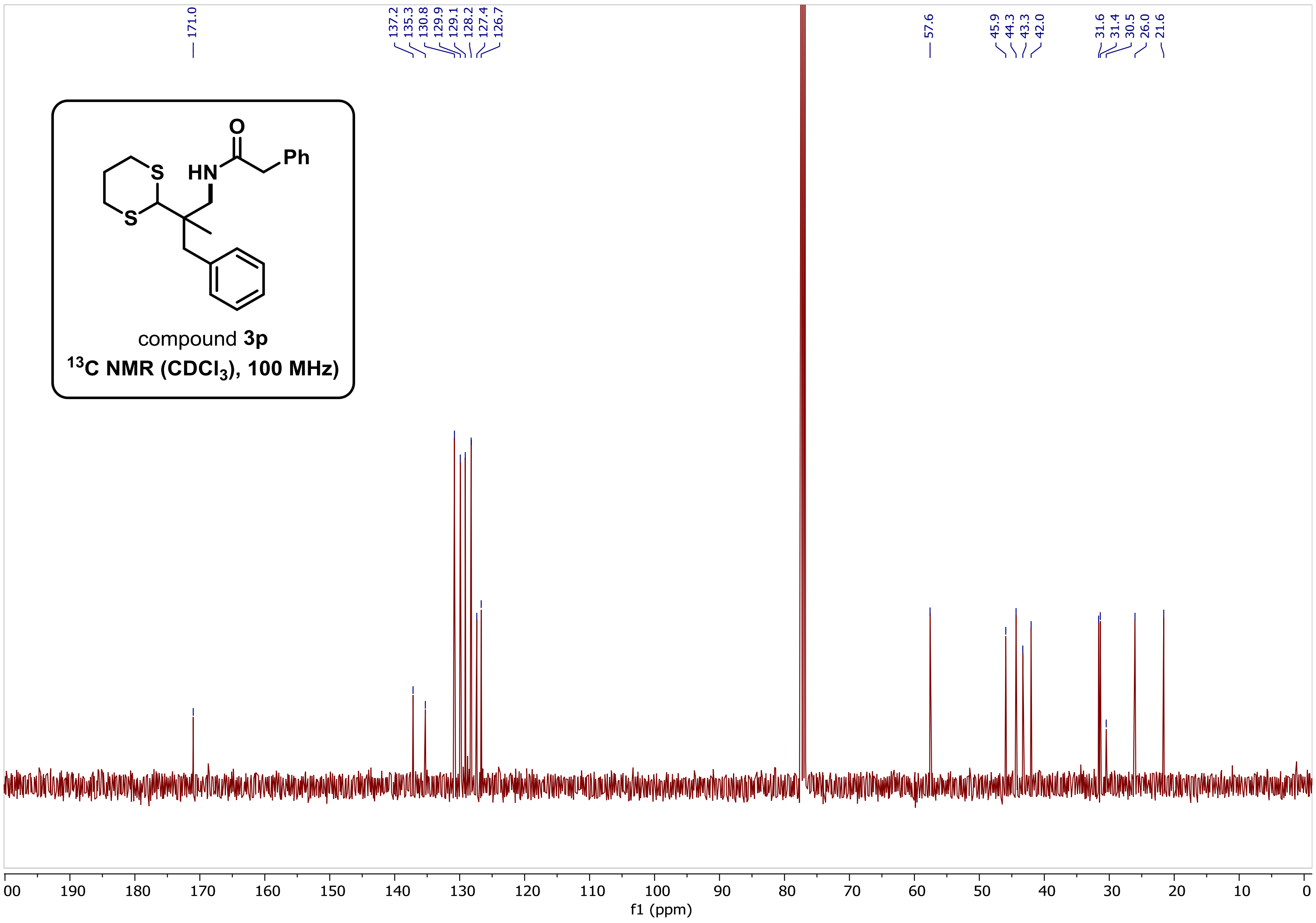
171.0
137.2
135.3
130.8
129.9
129.1
128.2
127.4
126.7
57.6
45.9
44.3
43.3
42.0
31.6
31.4
30.5
26.0
21.6
O
Ph
S
HN
S
compound 3p
13C NMR (CDCl3), 100 MHz)
f1 (ppm)

3.37. Compound 3q

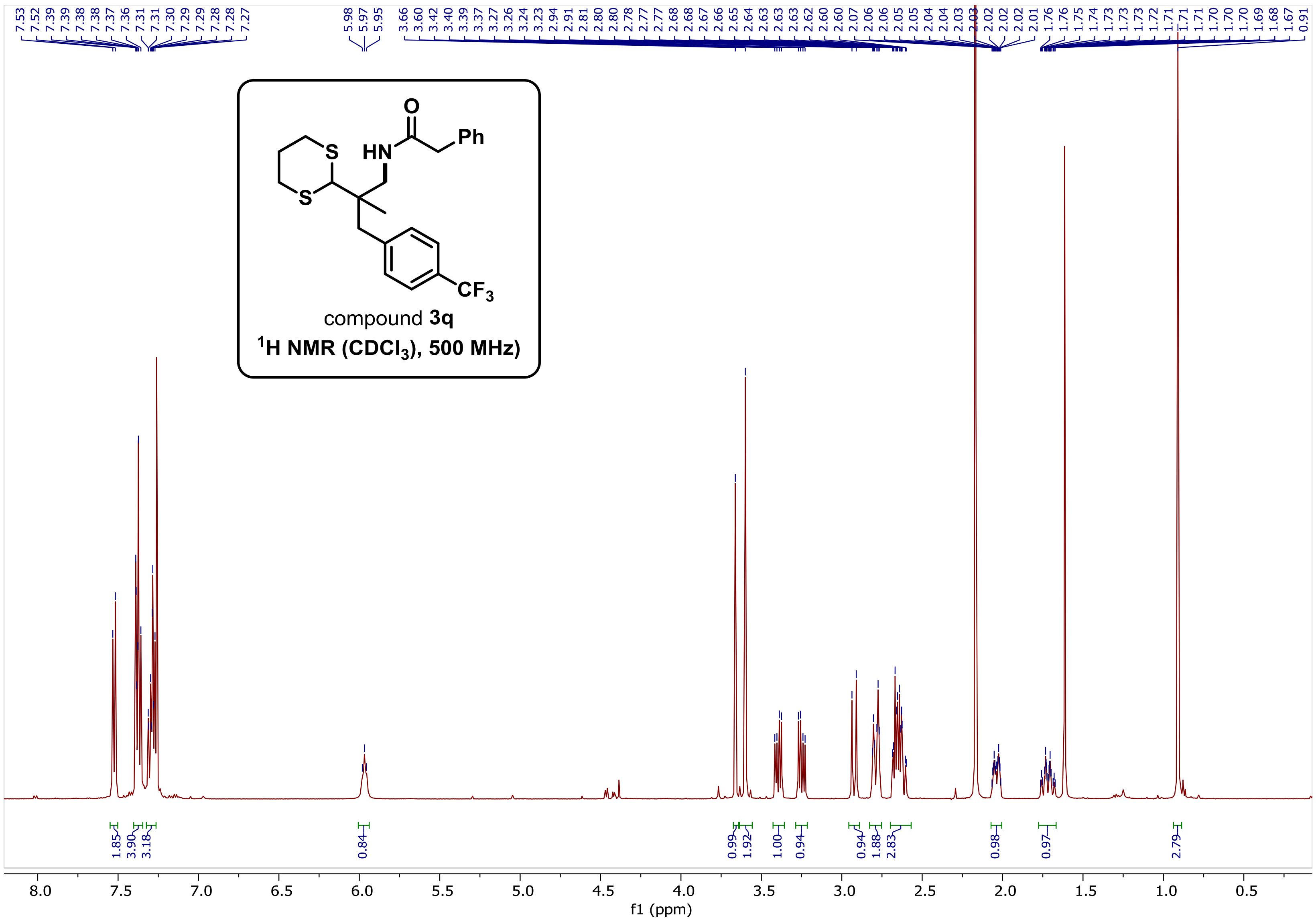

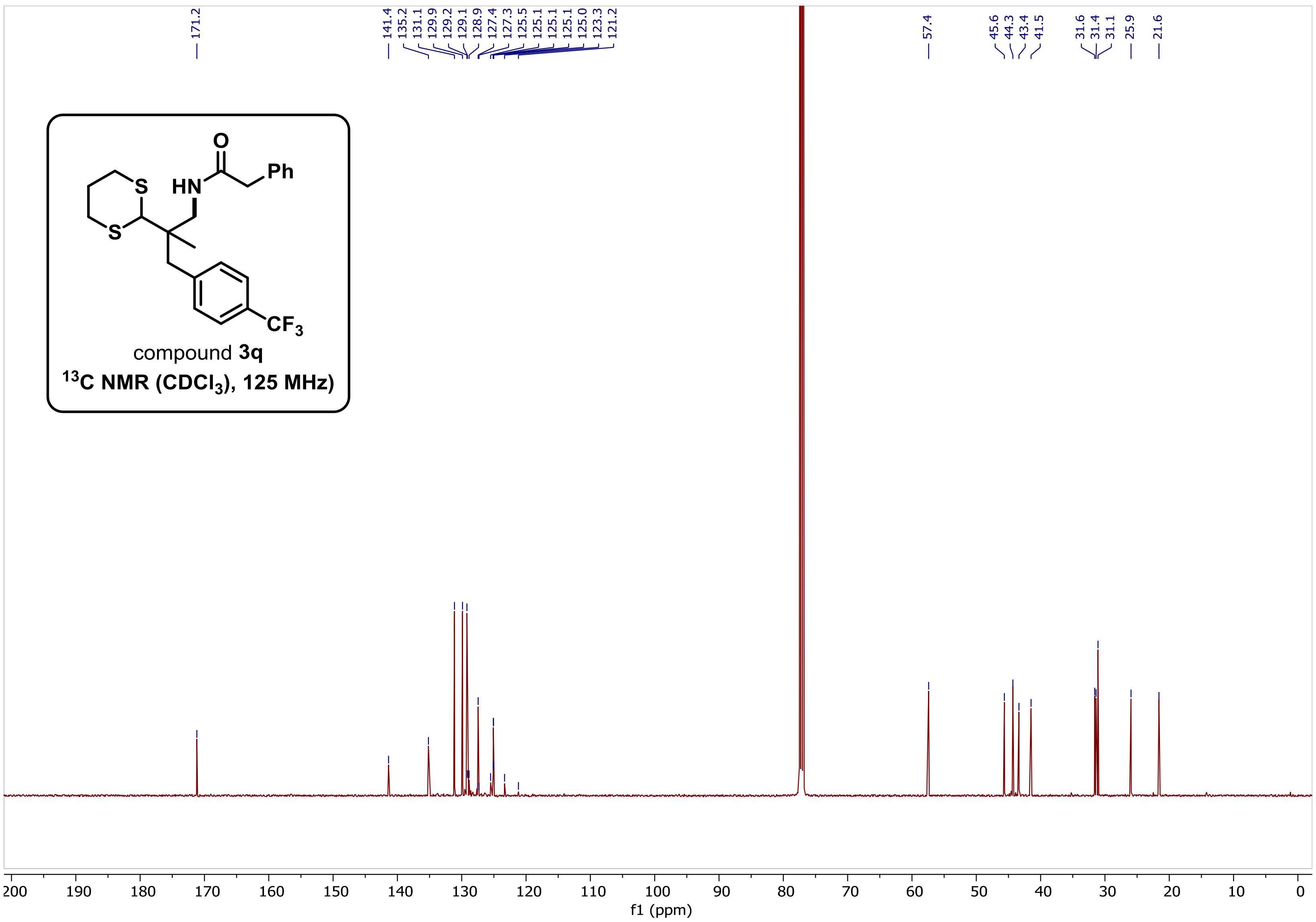

171.2
141.4
135.2
131.1
129.9
129.2
129.1
128.9
127.4
127.3
125.5
125.1
125.1
125.1
125.0
123.3
121.2
57.4
45.6
44.3
43.4
41.5
31.6
31.4
31.1
25.9
21.6
O
Ph
S
HN
S
CF3
compound 3q
13C NMR (CDCl3), 125 MHz)
200 190 180 170 160 150 140 130 120 110 100 90 80 70 60 50 40 30 20 10 0
f1 (ppm)

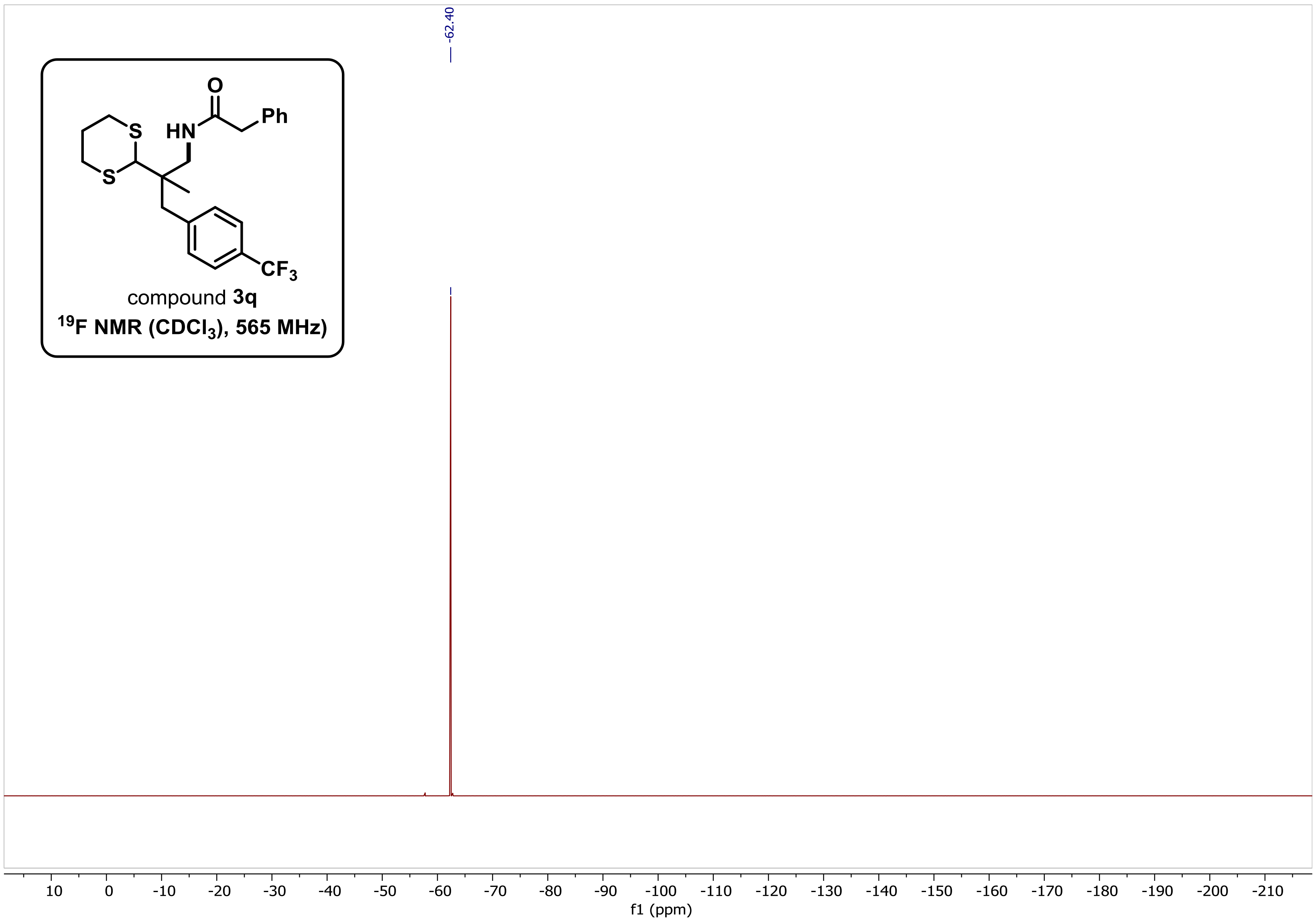
-62.40
O
Ph
S
HN
S
CF3
compound 3q
19F NMR (CDCl3), 565 MHz)
10 0 -10 -20 -30 -40 -50 -60 -70 -80 -90 -100 -110 -120 -130 -140 -150 -160 -170 -180 -190 -200 -210
f1 (ppm)

3.38. Compound 3r

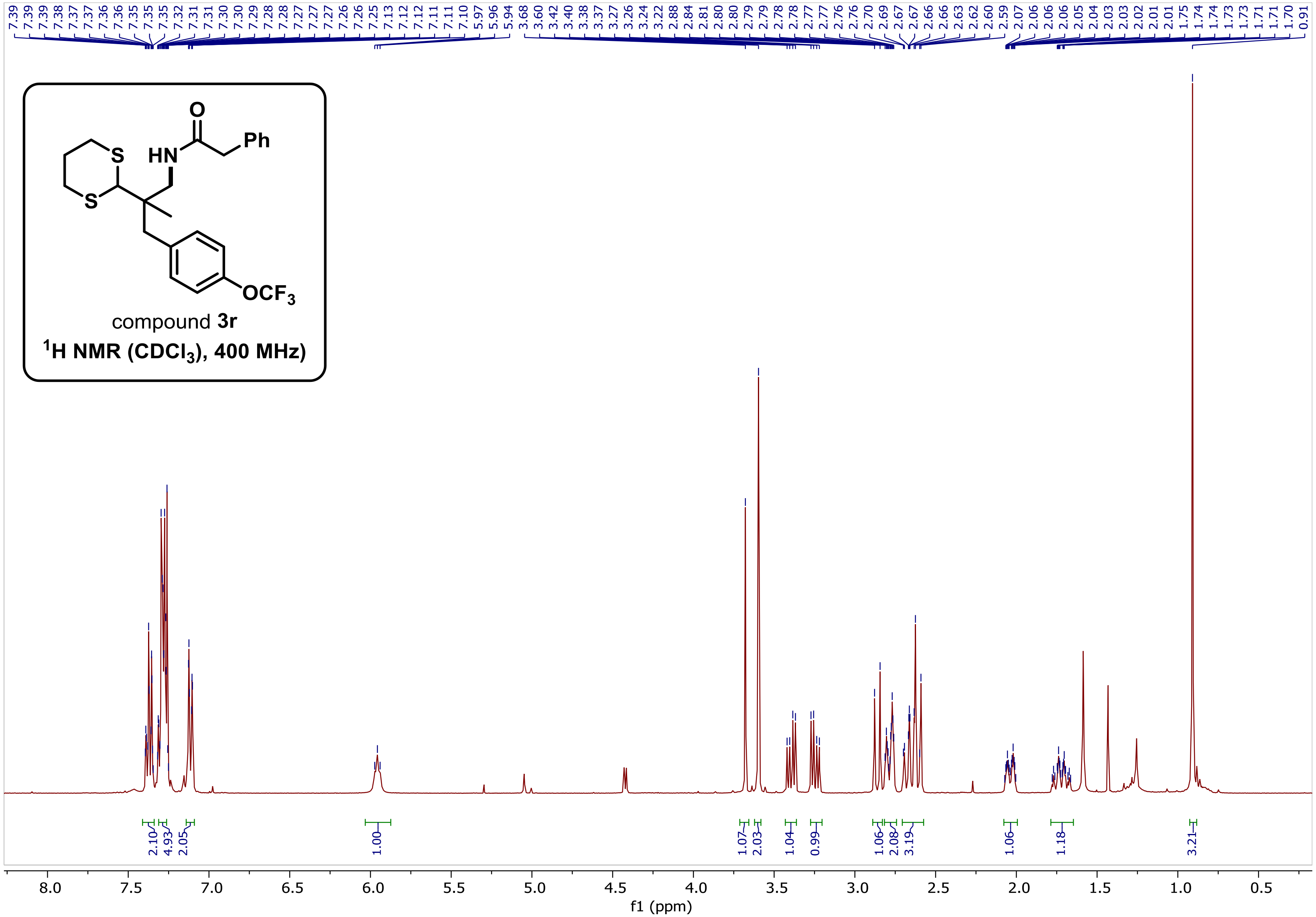

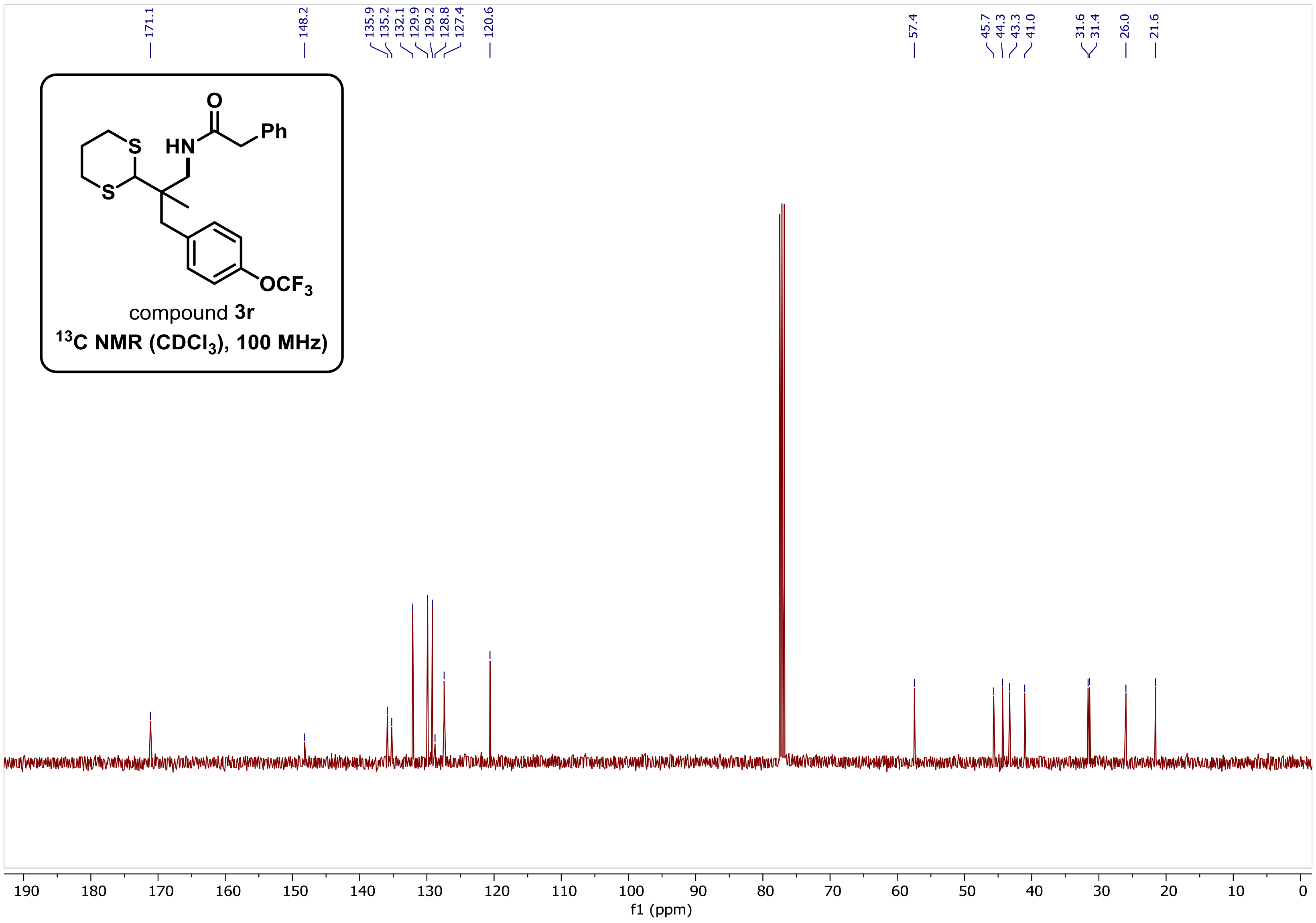

171.1
148.2
135.9
135.2
132.1
129.9
129.2
128.8
127.4
120.6
57.4
45.7
44.3
43.3
41.0
31.6
31.4
26.0
21.6
O
Ph
S
HN
S
OCF3
compound 3r
13C NMR (CDCl3), 100 MHz)
190
180
170
160
150
140
130
120
110
100
90
80
70
60
50
40
30
20
10
0
f1 (ppm)

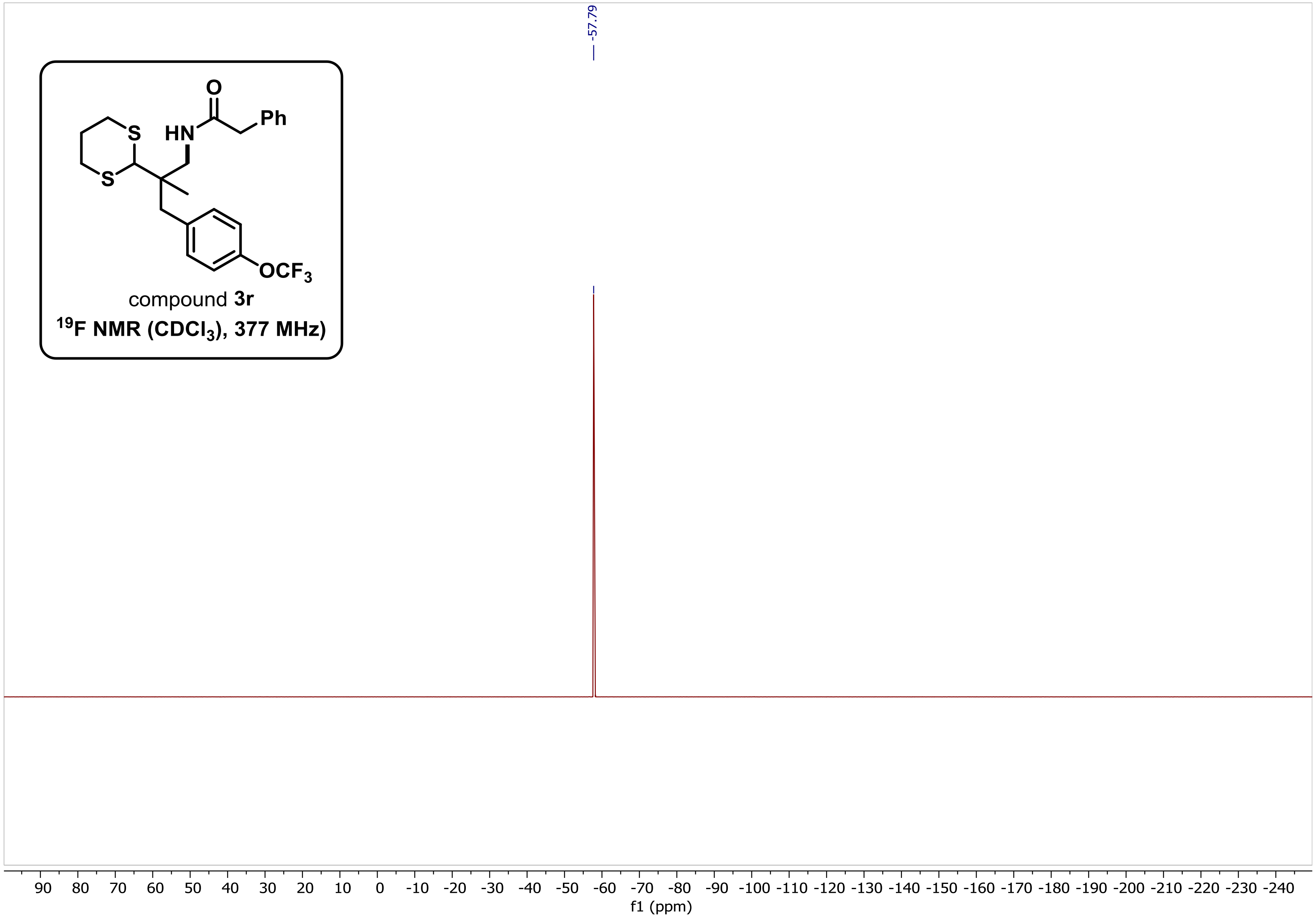
-57.79
O
Ph
S
HN
S
OCF3
compound 3r
19F NMR (CDCl3), 377 MHz)
90 80 70 60 50 40 30 20 10 0 -10 -20 -30 -40 -50 -60 -70 -80 -90 -100 -110 -120 -130 -140 -150 -160 -170 -180 -190 -200 -210 -220 -230 -240
f1 (ppm)

3.39. Compound 3s

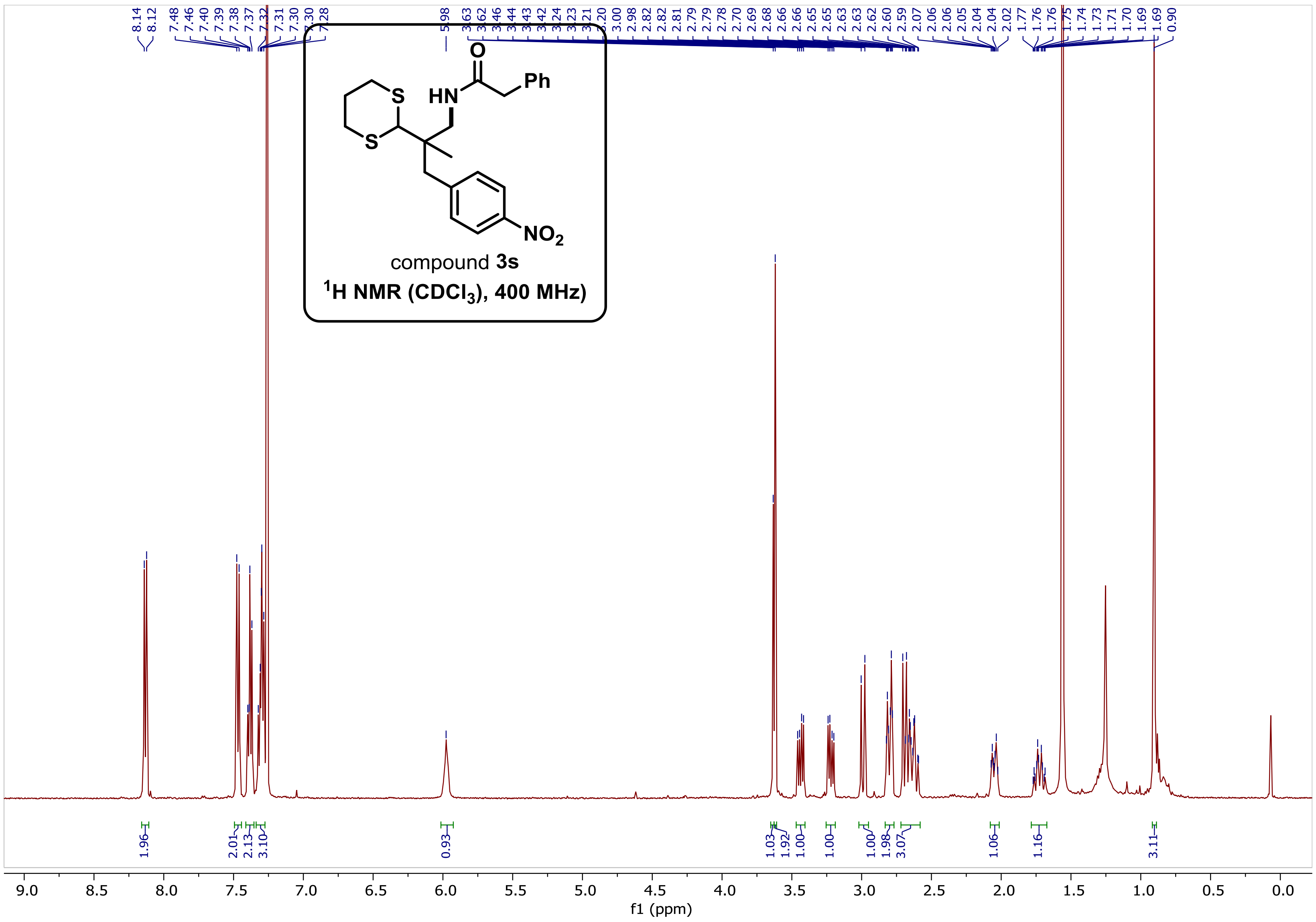

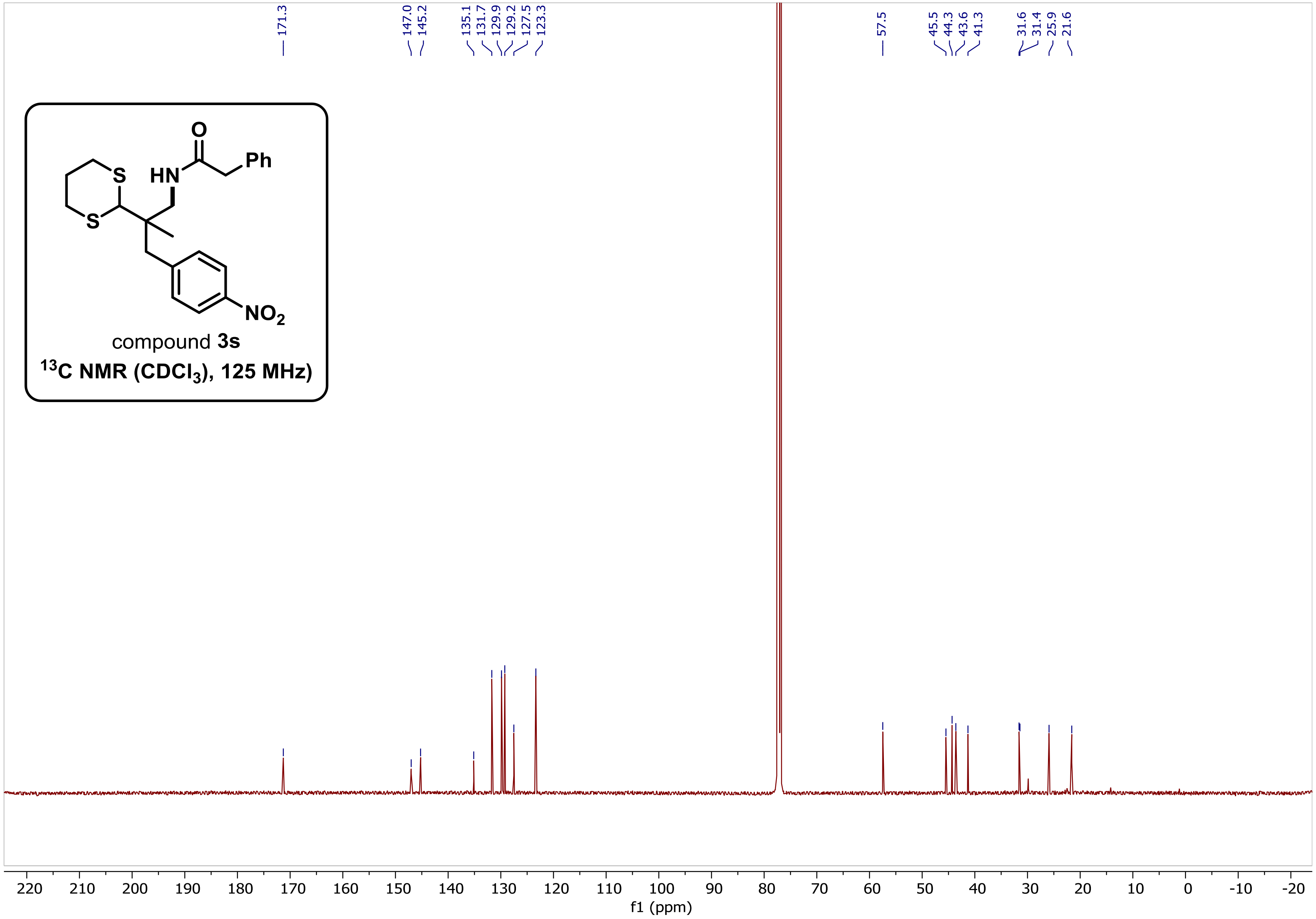
171.3
147.0
145.2
135.1
131.7
129.9
129.2
127.5
123.3
57.5
45.5
44.3
43.6
41.3
31.6
31.4
25.9
21.6
O
Ph
S
HN
S
NO2
compound 3s
13C NMR (CDCl3), 125 MHz)
220
210
200
190
180
170
160
150
140
130
120
110
100
90
80
70
60
50
40
30
20
10
0
-10
-20
f1 (ppm)

Compound 3t

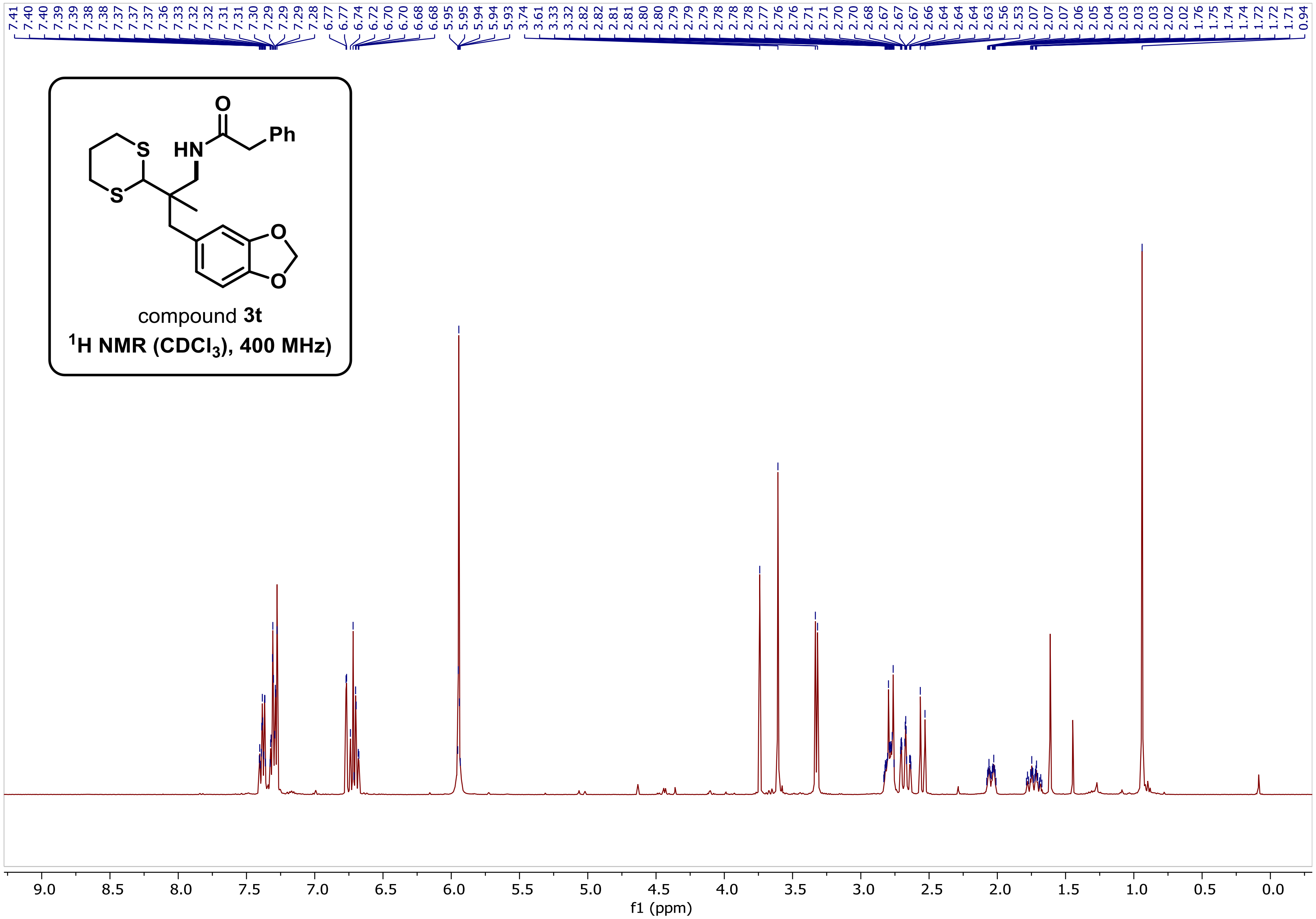

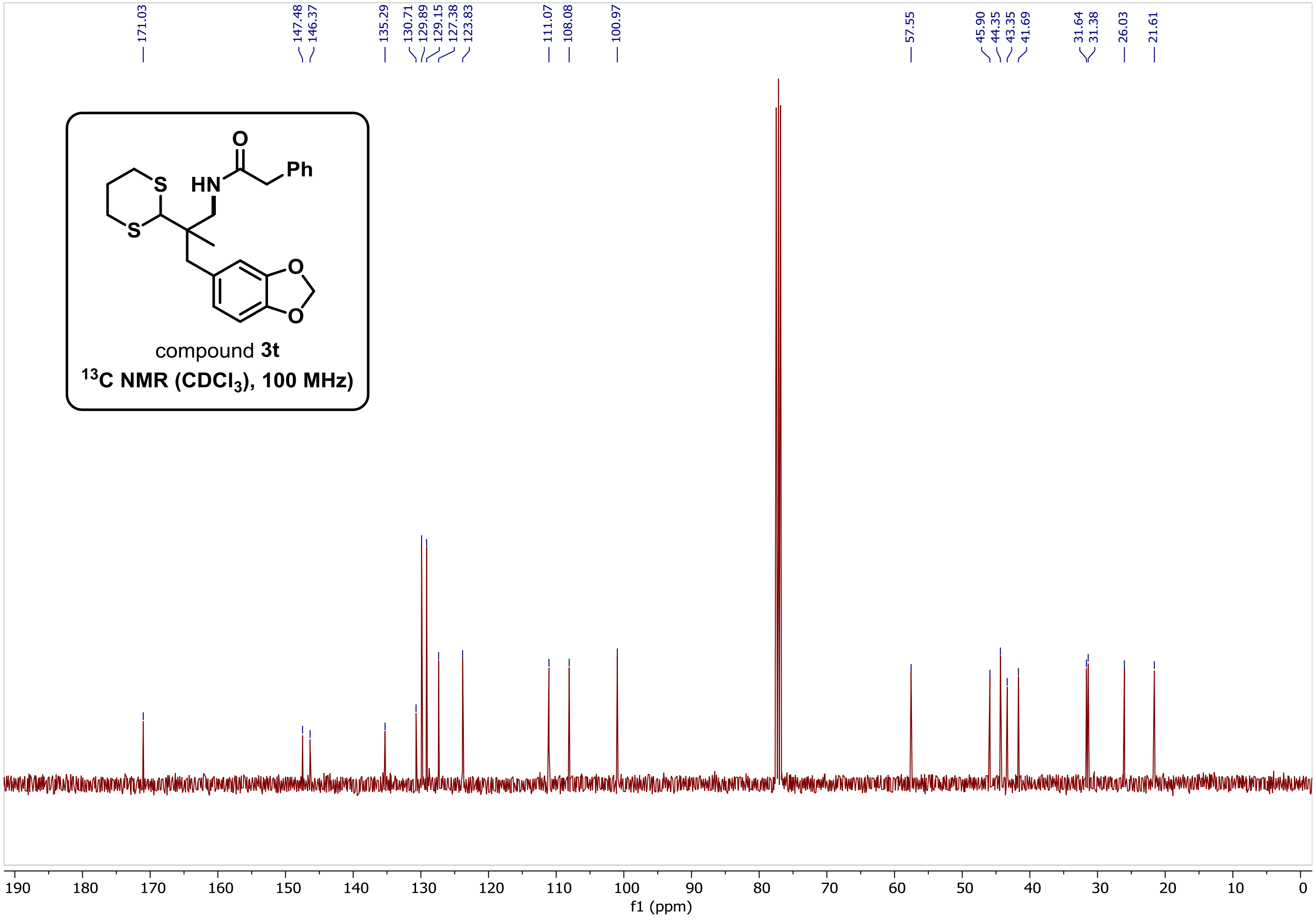
171.03
147.48
146.37
135.29
130.71
129.89
129.15
127.38
123.83
111.07
108.08
100.97
57.55
45.90
44.35
43.35
41.69
31.64
31.38
26.03
21.61
O
Ph
S
HN
S
O
O
compound 3t
^{13}C NMR (CDCl$_3$), 100 MHz)
190 180 170 160 150 140 130 120 110 100 90 80 70 60 50 40 30 20 10 0
f1 (ppm)

3.40. Compound 3u

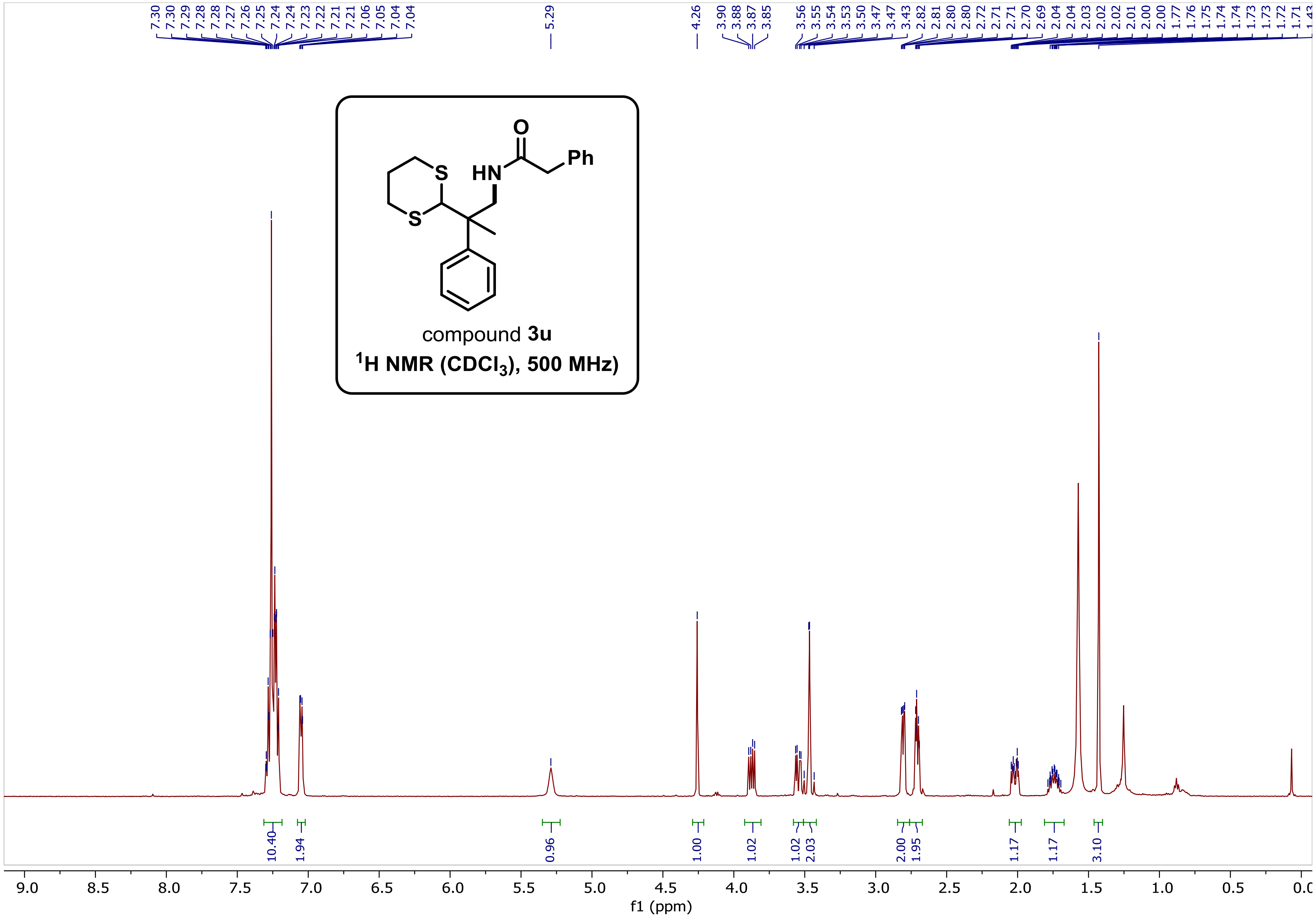

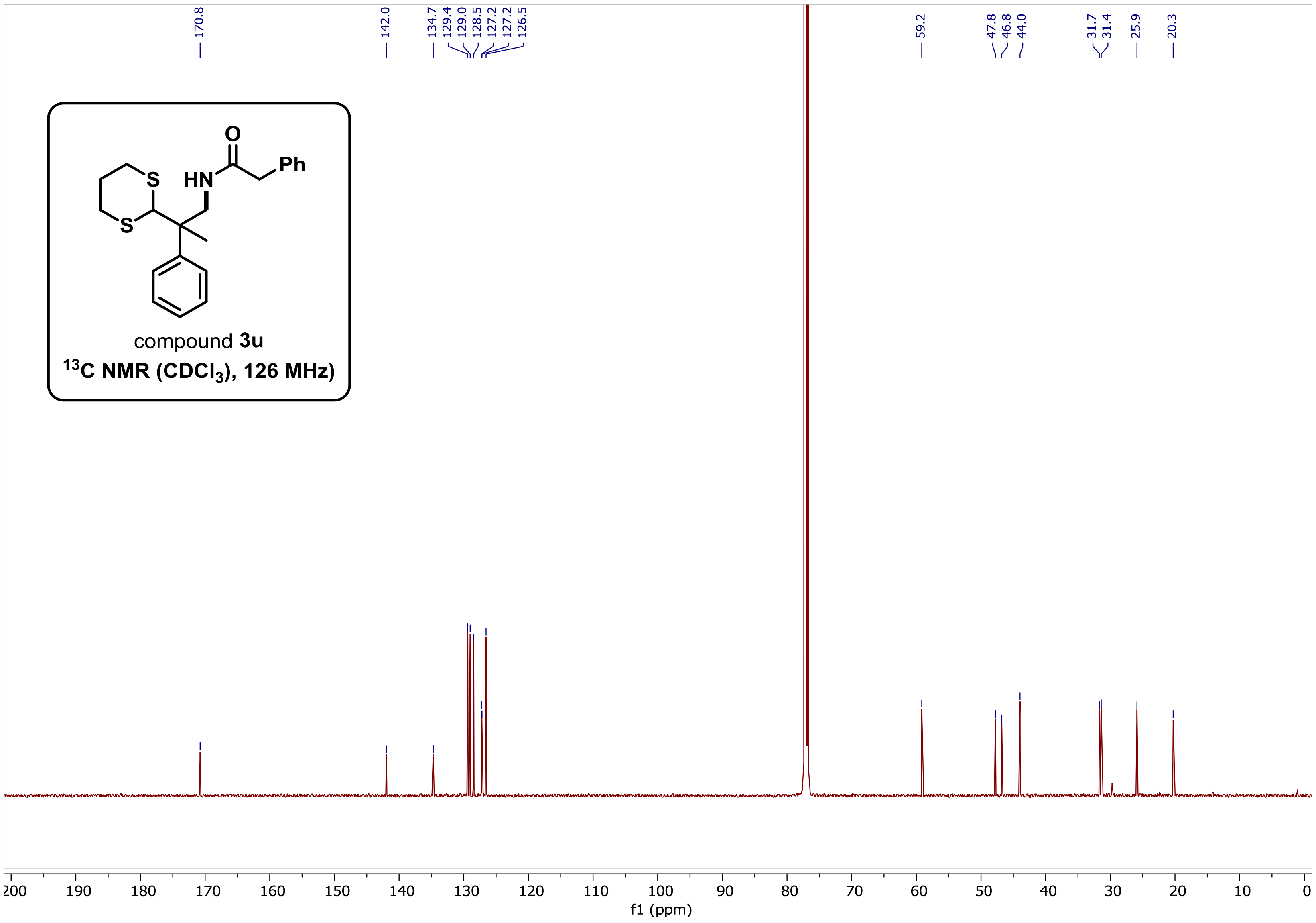
170.8
142.0
134.7
129.4
129.0
128.5
127.2
127.2
126.5
59.2
47.8
46.8
44.0
31.7
31.4
25.9
20.3
O
Ph
S
HN
S
compound 3u
^{13}C NMR ($CDCl_3$), 126 MHz)
200
190
180
170
160
150
140
130
120
110
100
90
80
70
60
50
40
30
20
10
0
f1 (ppm)

Compound 3v

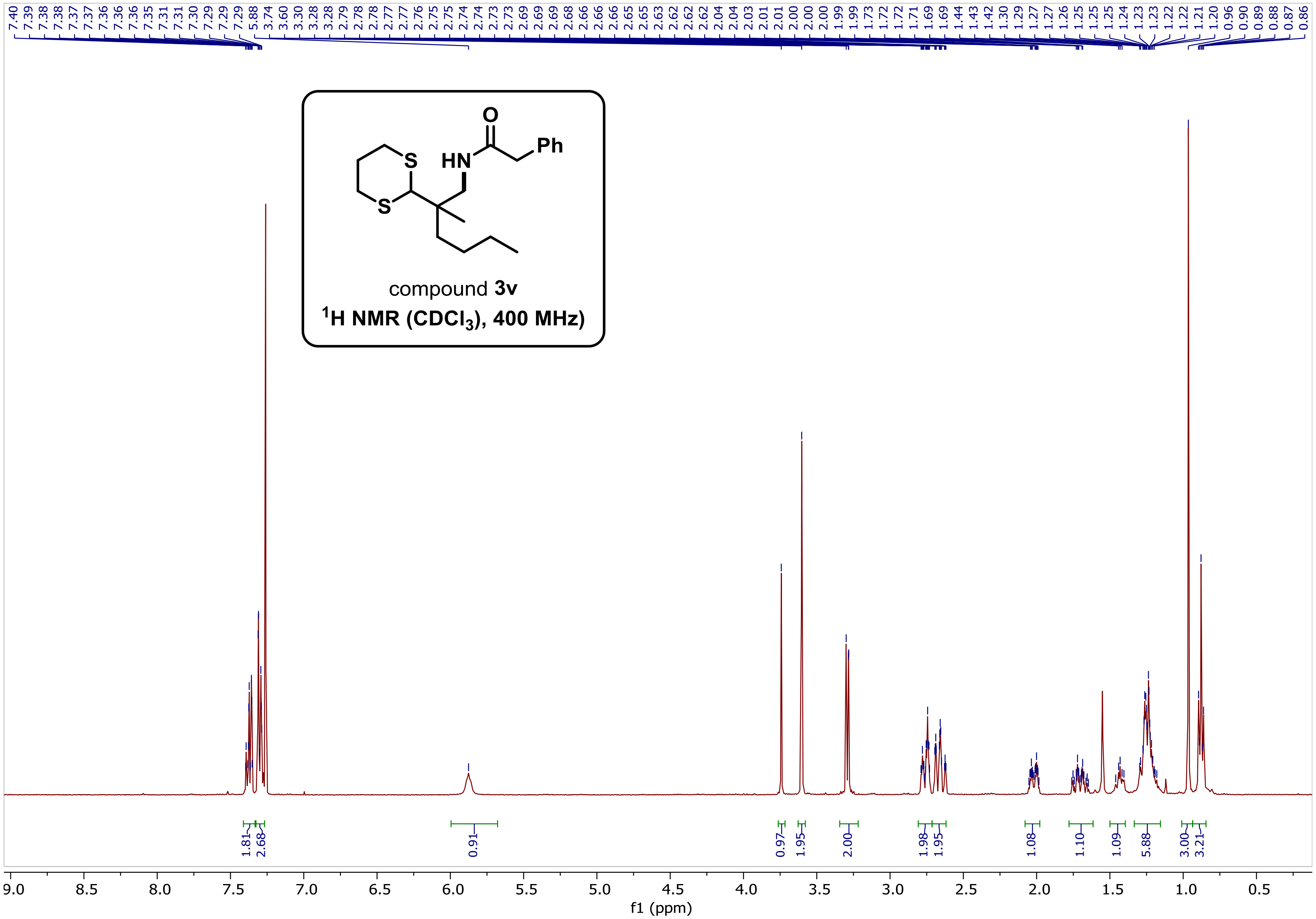

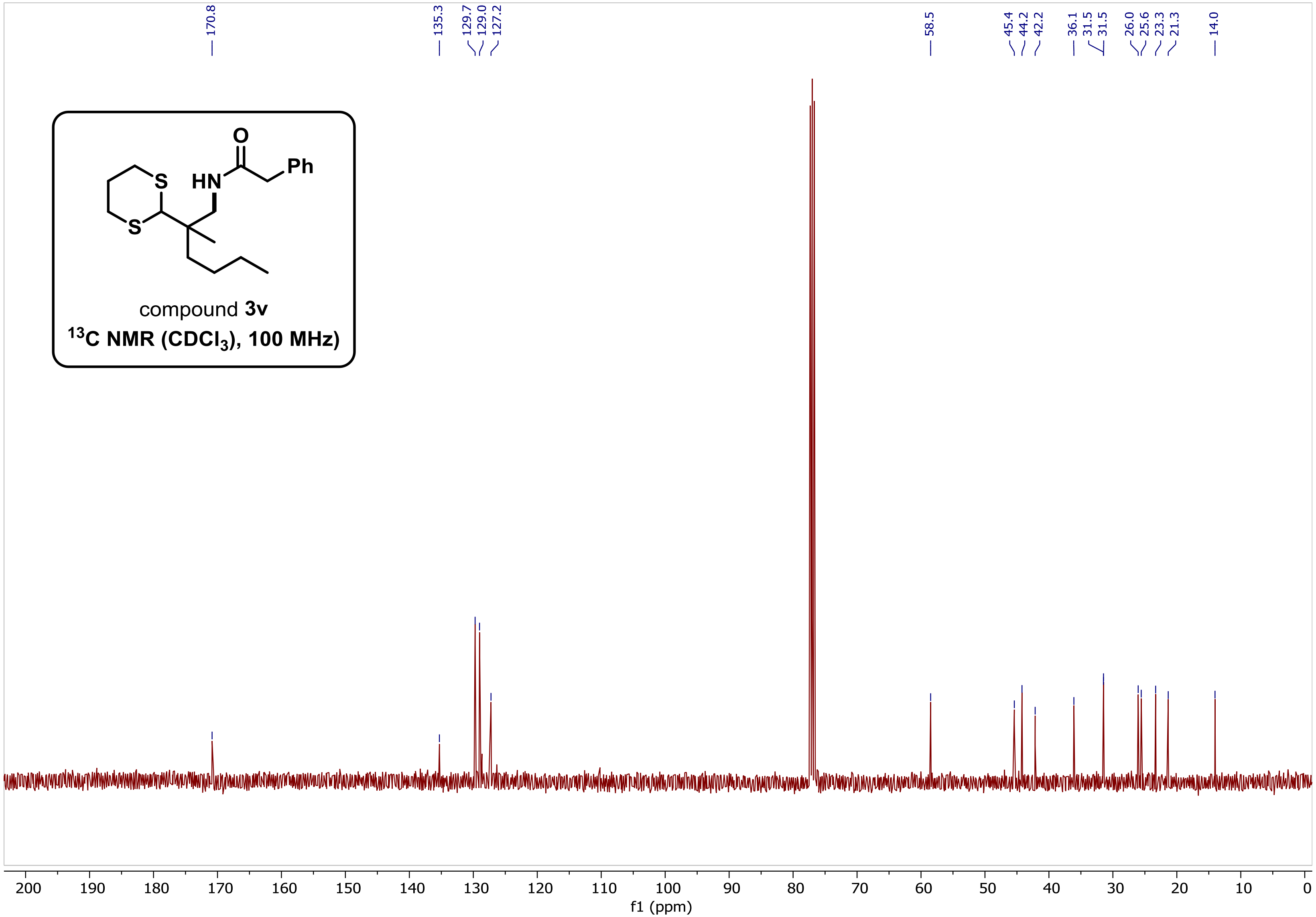

170.8
135.3
129.7
129.0
127.2
58.5
45.4
44.2
42.2
36.1
31.5
31.5
26.0
25.6
23.3
21.3
14.0
O
Ph
S
HN
S
compound 3v
13C NMR (CDCl3), 100 MHz)
200 190 180 170 160 150 140 130 120 110 100 90 80 70 60 50 40 30 20 10 0
f1 (ppm)

3.41. Compound 3w

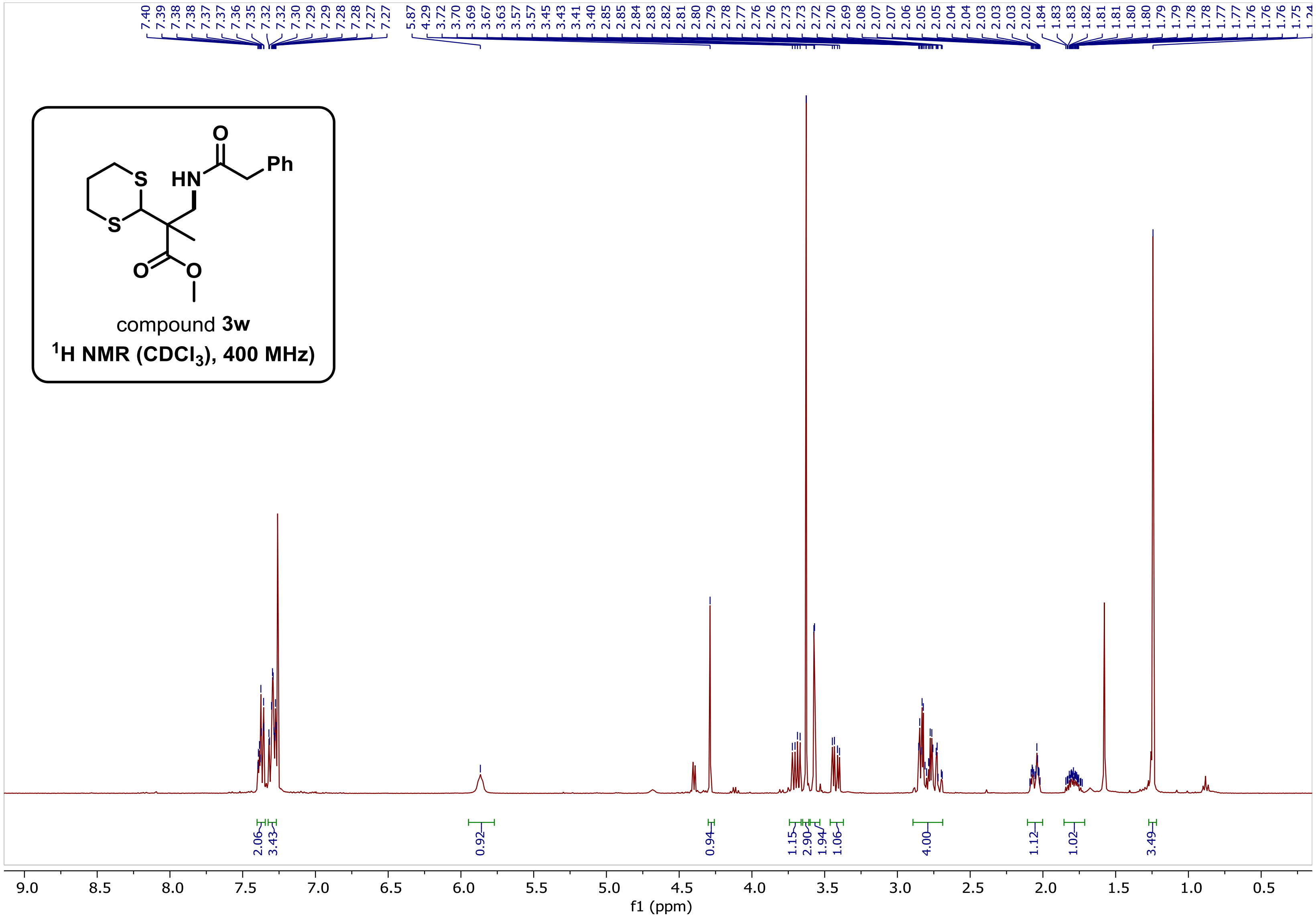

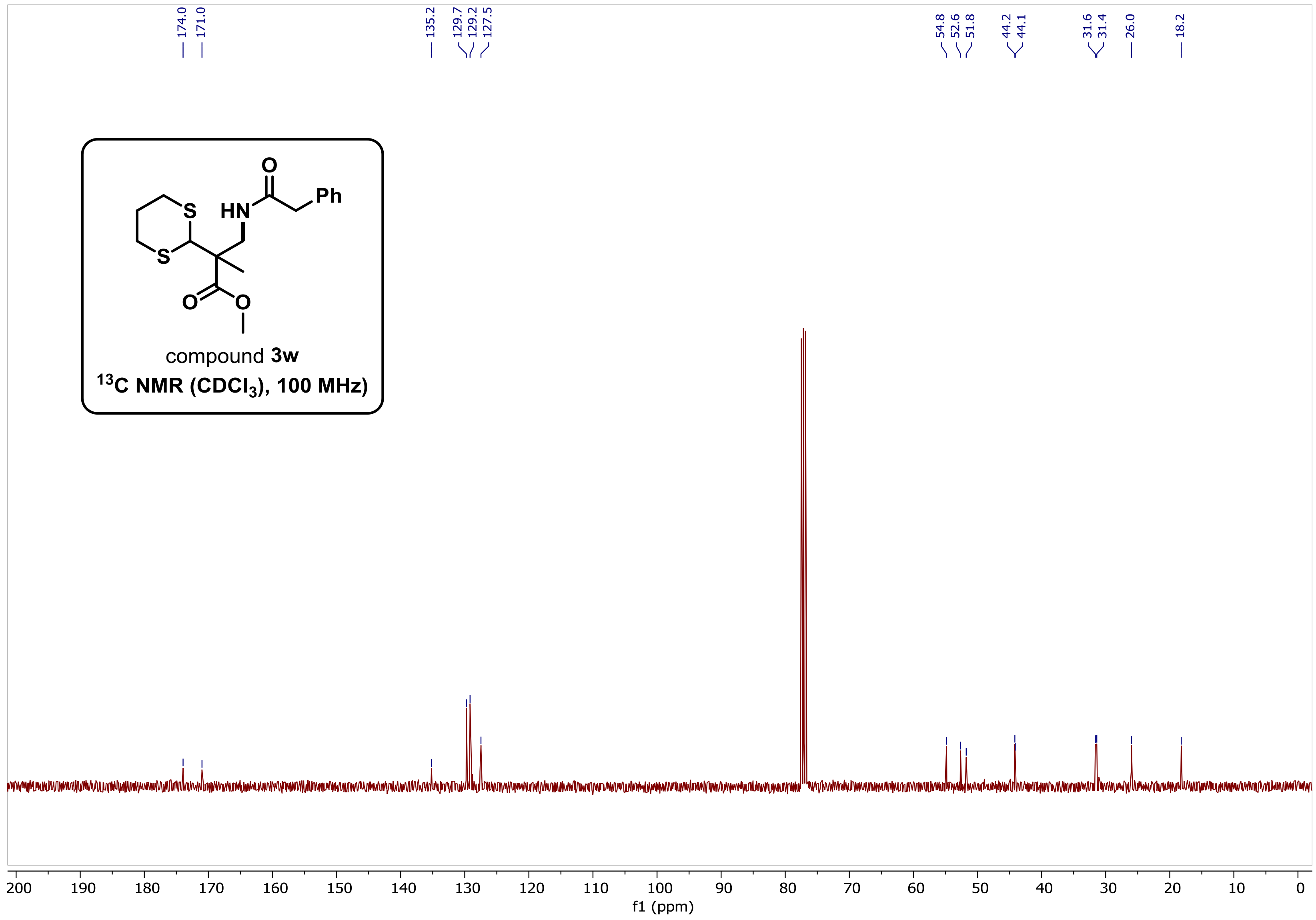

174.0
171.0
135.2
129.7
129.2
127.5
54.8
52.6
51.8
44.2
44.1
31.6
31.4
26.0
18.2
compound 3w
13C NMR (CDCl3), 100 MHz)
f1 (ppm)

3.42. Compound 3x

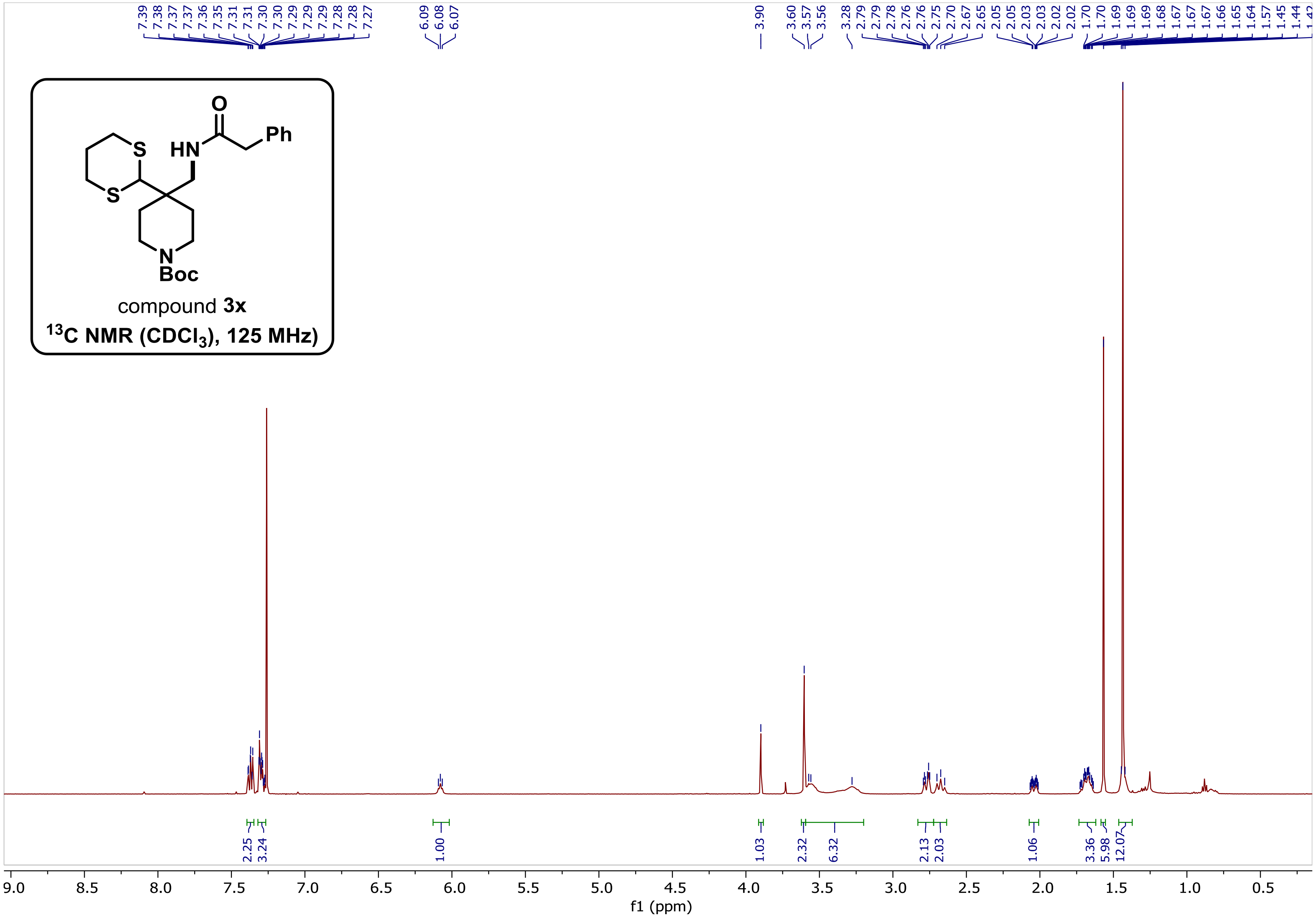

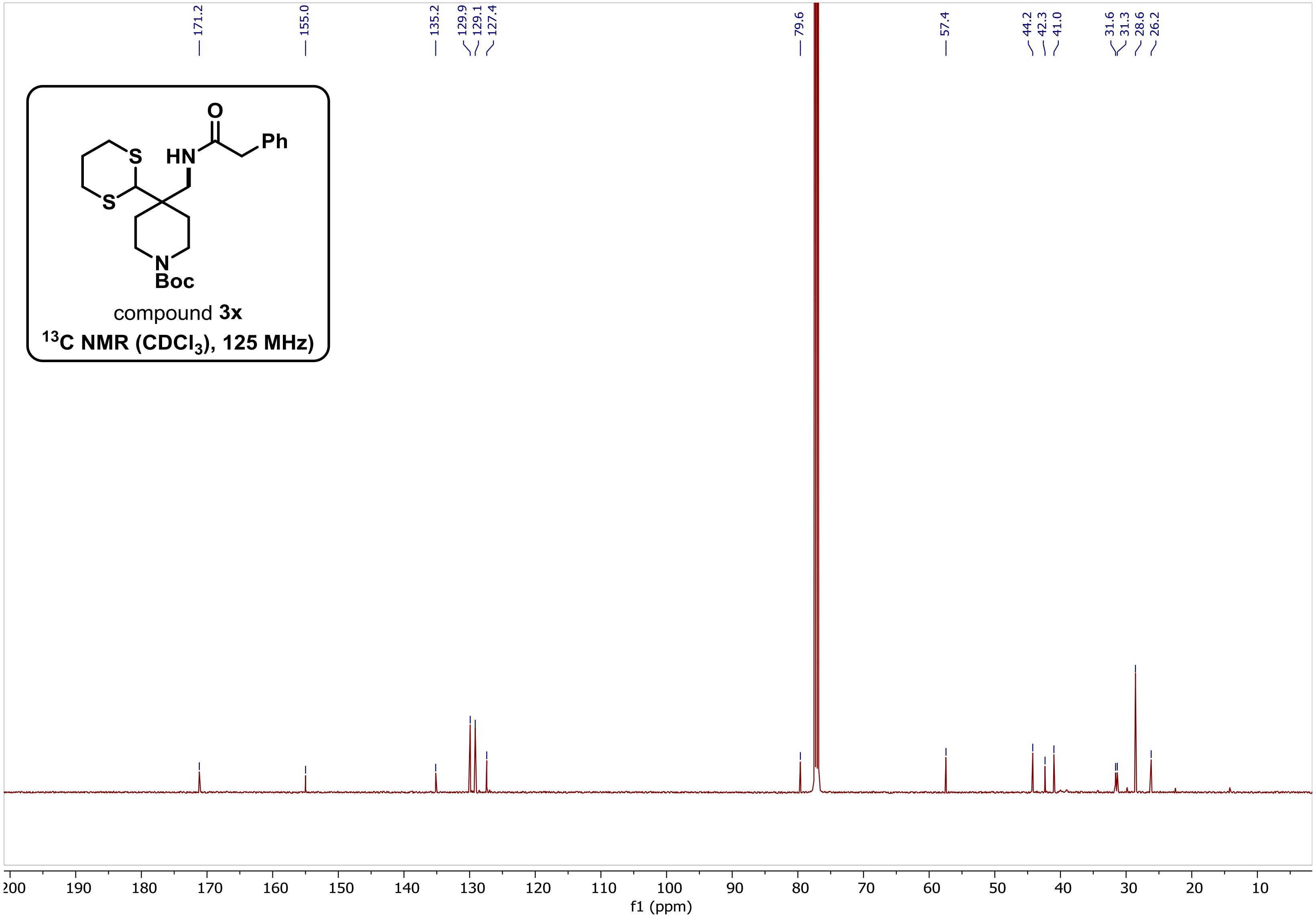
171.2
155.0
135.2
129.9
129.1
127.4
79.6
57.4
44.2
42.3
41.0
31.6
31.3
28.6
26.2
O
HN
Ph
S
S
N
Boc
compound 3x
^{13}C NMR ($CDCl_3$), 125 MHz)
200
190
180
170
160
150
140
130
120
110
100
90
80
70
60
50
40
30
20
10
f1 (ppm)

3.43. Compound 6

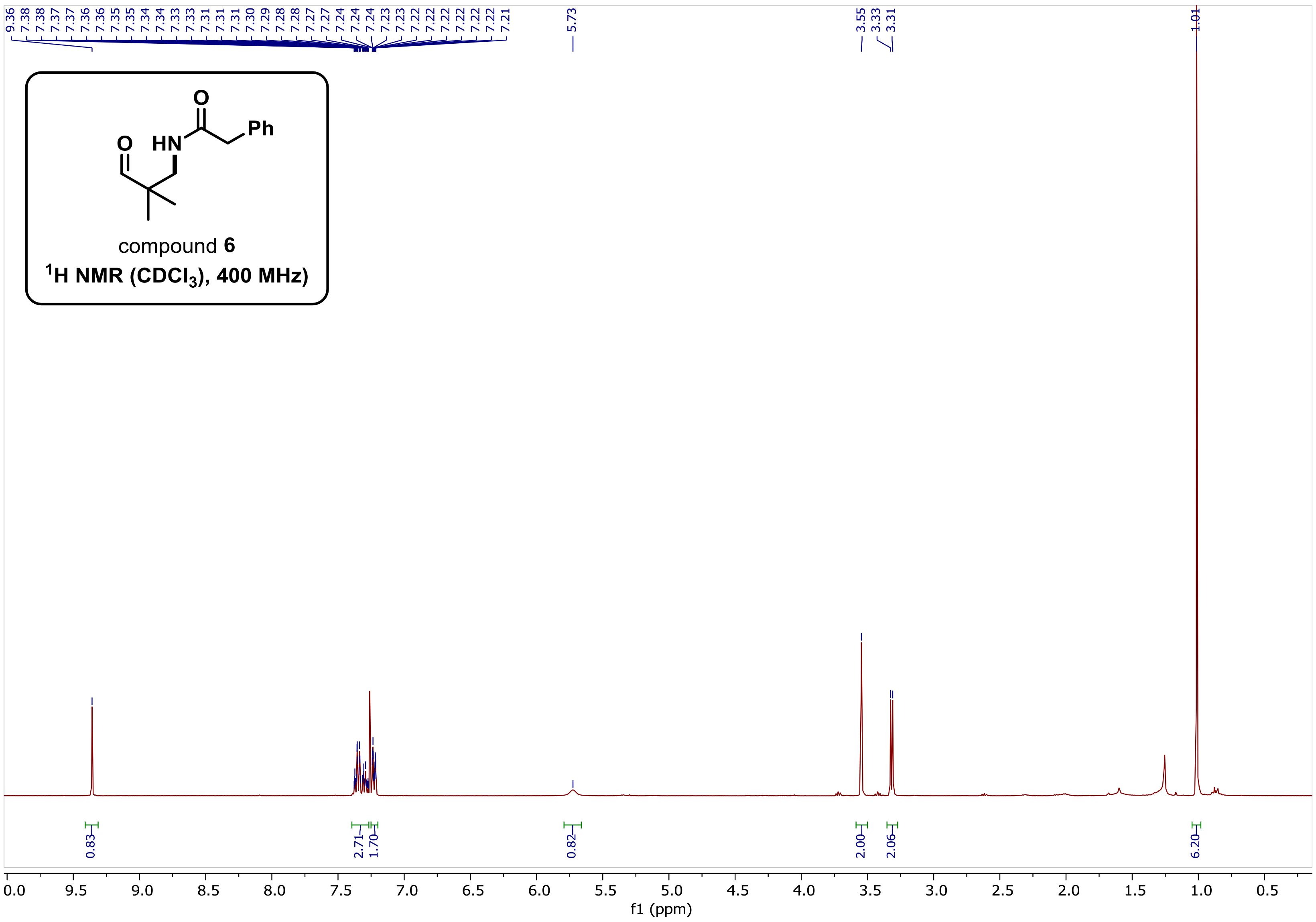

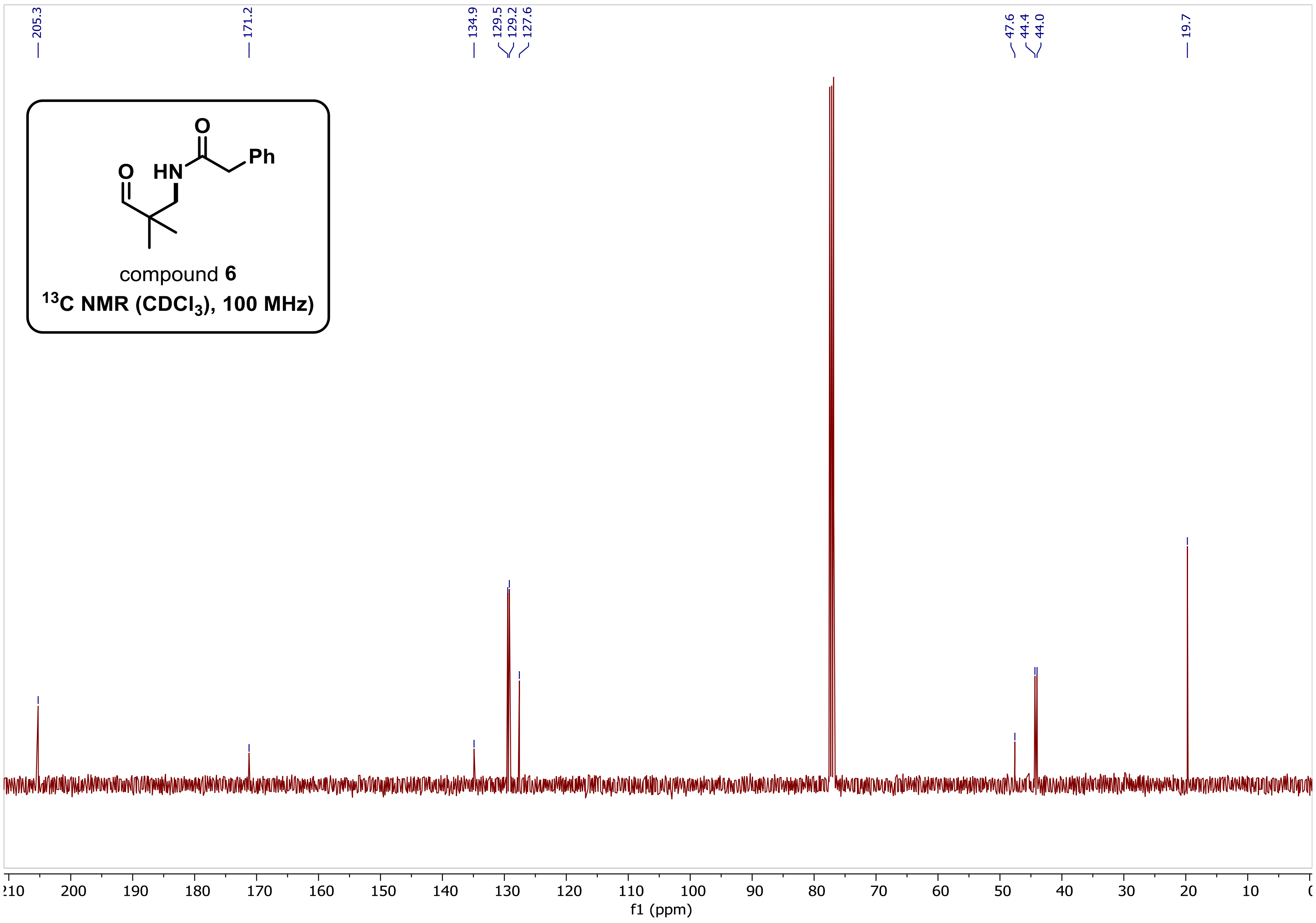

205.3
171.2
134.9
129.5
129.2
127.6
47.6
44.4
44.0
19.7
O
HN
O
Ph
compound 6
13C NMR (CDCl3), 100 MHz)
210
200
190
180
170
160
150
140
130
120
110
100
90
80
70
60
50
40
30
20
10
f1 (ppm)

3.44. Compound 7

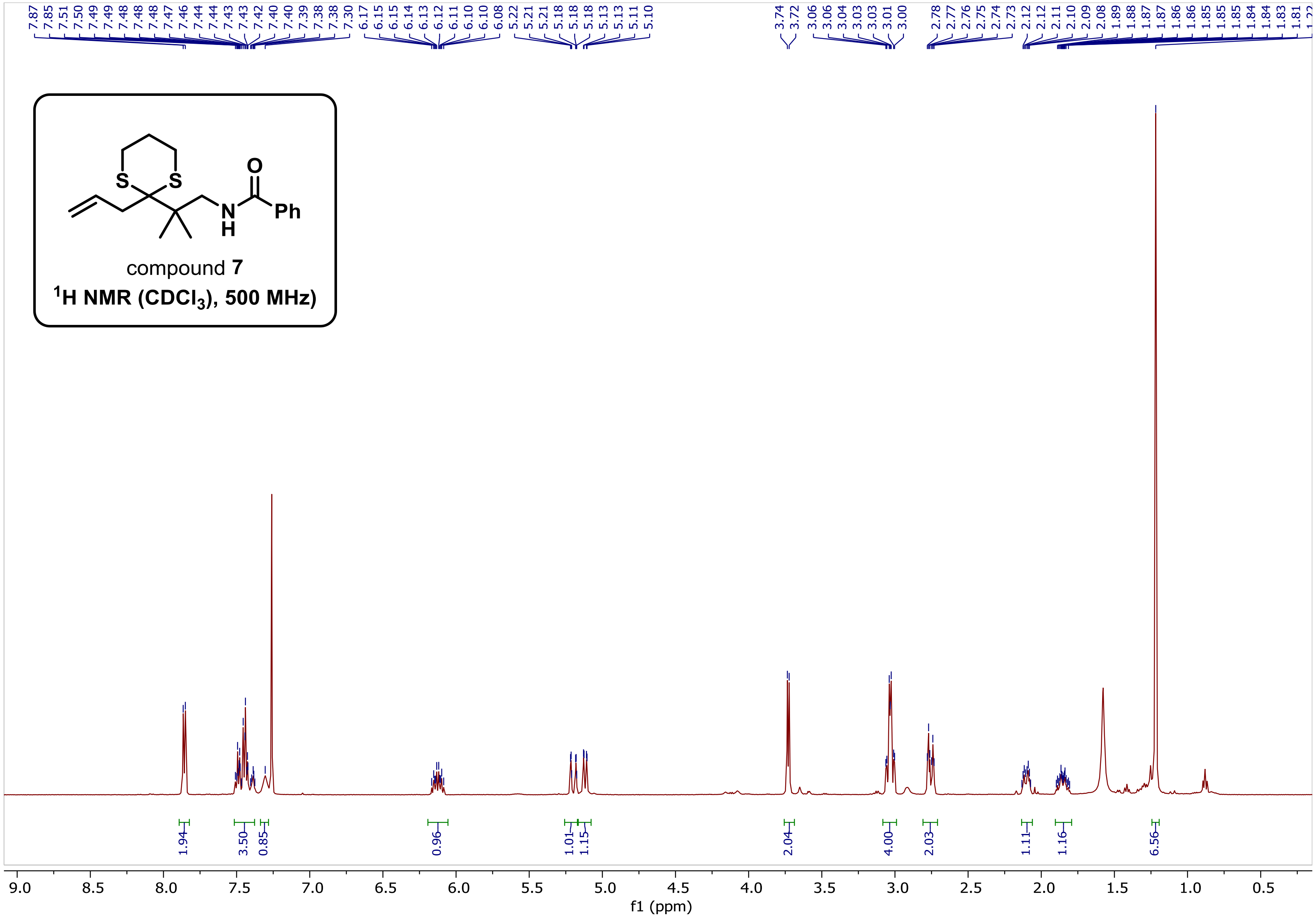

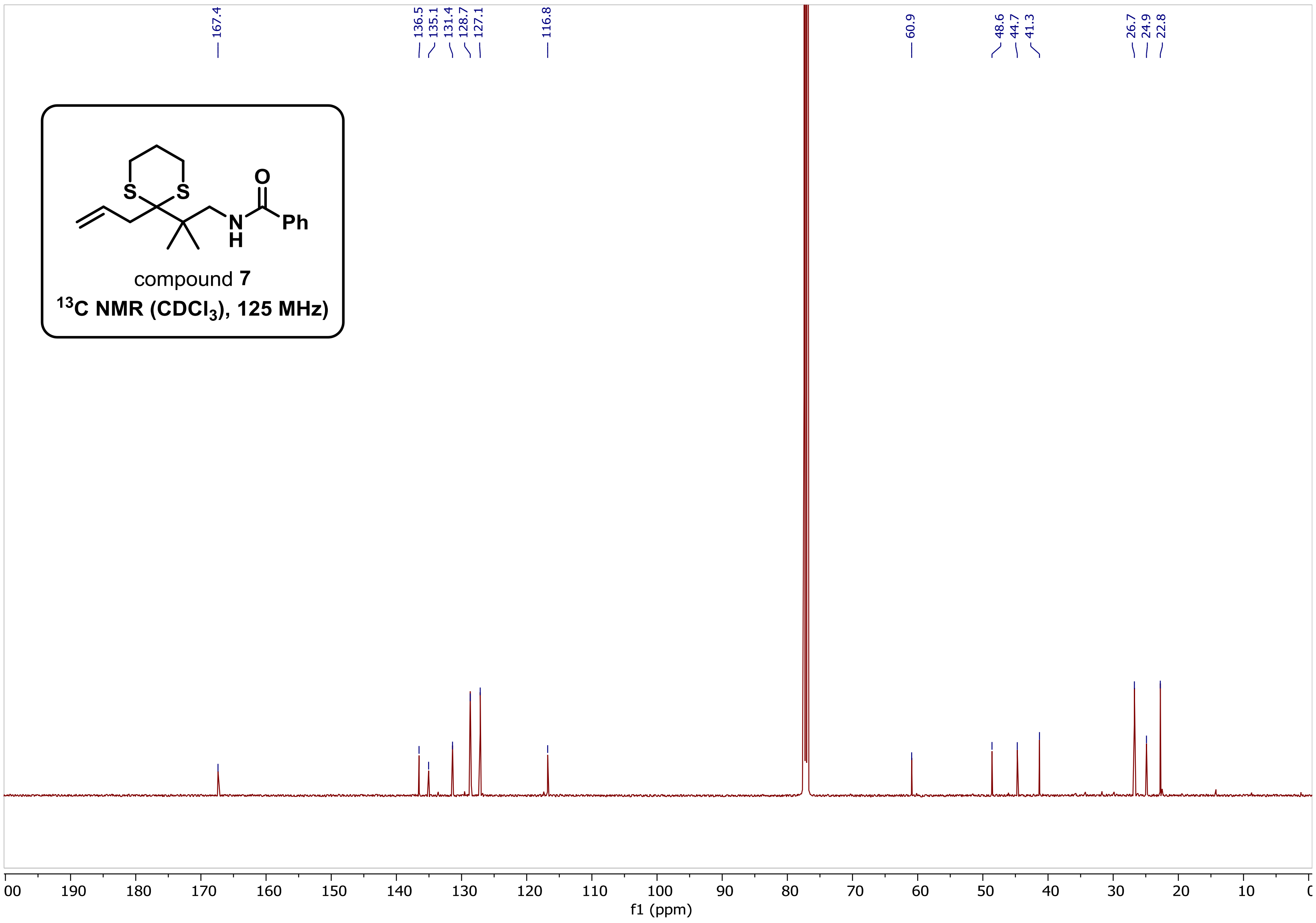

167.4
136.5
135.1
131.4
128.7
127.1
116.8
60.9
48.6
44.7
41.3
26.7
24.9
22.8
S
S
O
N
H
Ph
compound 7
13C NMR (CDCl3), 125 MHz)
f1 (ppm)

3.45. Compound 8

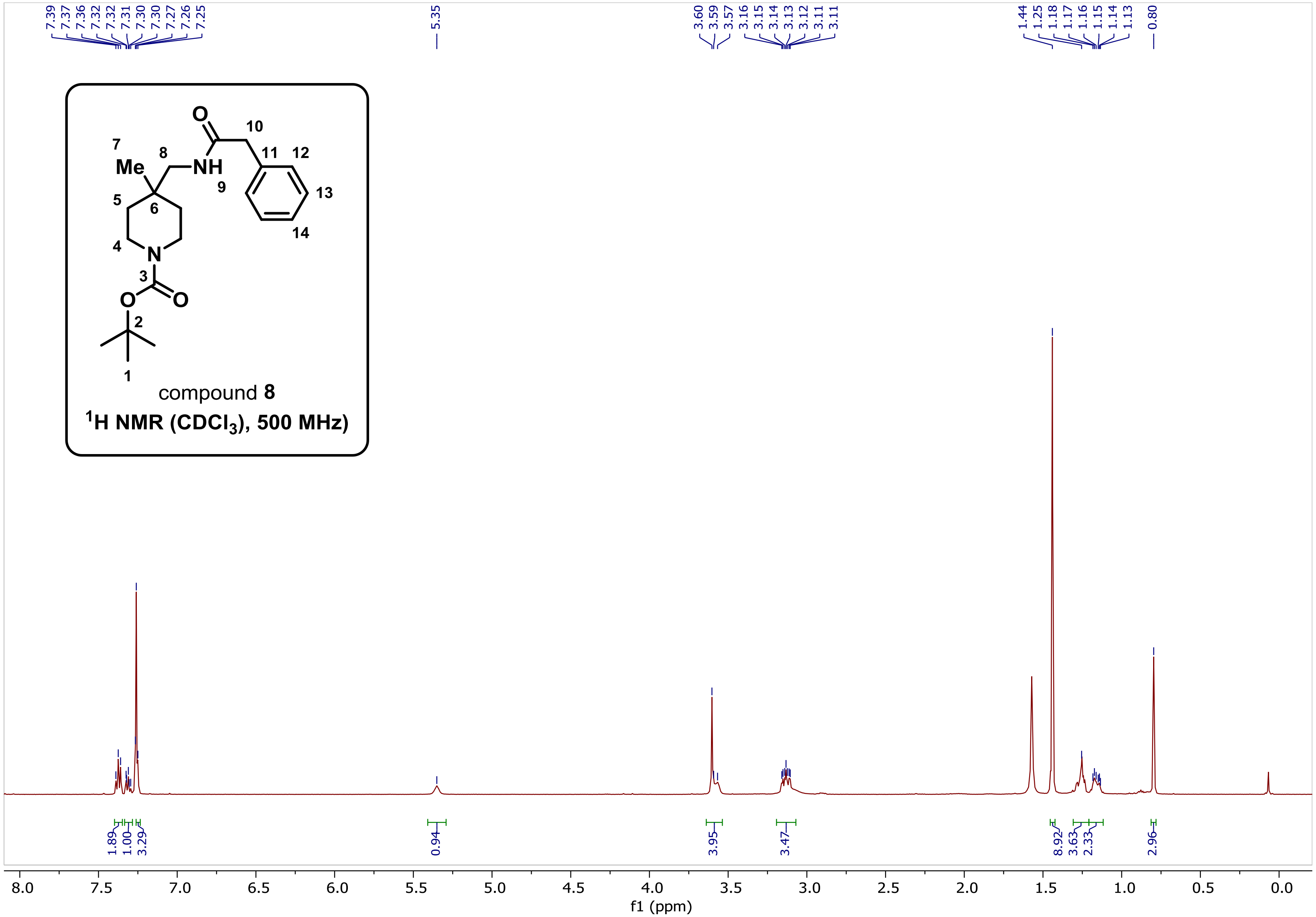

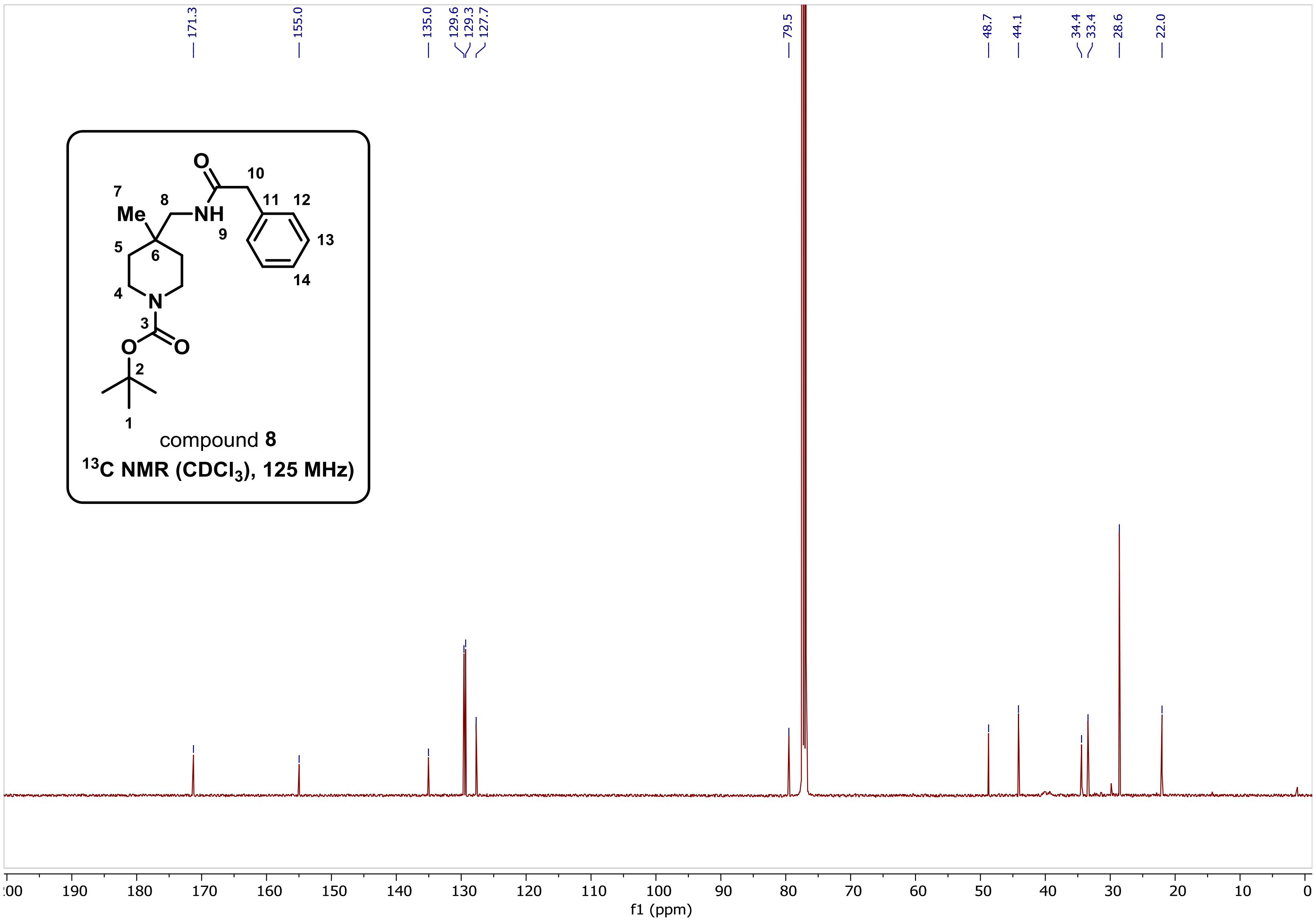

171.3
155.0
135.0
129.6
129.3
127.7
79.5
48.7
44.1
34.4
33.4
28.6
22.0
O
10
7
8
11
12
Me
NH
9
13
5
6
14
4
N
3
O
O
2
1
compound **8**
^{13}C NMR ($CDCl_3$), 125 MHz)
200
190
180
170
160
150
140
130
120
110
100
90
80
70
60
50
40
30
20
10
0
f1 (ppm)
